# Investigation of Antithrombotic Activity and In Vivo Effective Forms of Kaempferitrin Using FeCl_3_-Induced Rat Arterial Thrombosis and UHPLC-Q-Exactive Orbitrap MS

**DOI:** 10.3390/molecules30224434

**Published:** 2025-11-16

**Authors:** Jingjing Zhou, Ruixin Wang, Jingchen Hou, Yitong Qi, Yanglu Liu, Linying Niu, Xinyu Xia, Jinchen Shao, Yizhou Liu, Chunyan Liu, Hongfu Li

**Affiliations:** School of Pharmacy, North China University of Science and Technology, 21 Bohai Avenue, Caofeidian New Town, Tangshan 063210, China; zhoujingjing@ncst.edu.cn (J.Z.); wangruixin6231@163.com (R.W.); houjich452@163.com (J.H.); 15128980698@163.com (Y.Q.); 15128013049@163.com (Y.L.); nly01715@163.com (L.N.); xiaxinyu10051005@163.com (X.X.); shao_jinchen@163.com (J.S.); vicolyz@163.com (Y.L.)

**Keywords:** kaempferitrin, antithrombotic activity, in vivo effective forms, UHPLC-Q-Exactive Orbitrap MS, SRC/PI3K/AKT

## Abstract

Kaempferitrin (KAE) is a natural flavonol dirhamnopyranoside with various pharmacological activities, isolated from the antithrombotic fraction of *Celastrus orbiculatus* Thunb. This study aimed to investigate the antithrombotic activity and “effective forms” of KAE. The results showed that KAE significantly prolonged rabbit plasma recalcification time in vitro. In the FeCl_3_-induced rat arterial thrombosis model, KAE demonstrated antithrombotic effects by inhibiting coagulation, platelet aggregation, and fibrinolysis, with a lesser risk of bleeding compared to aspirin. KAE was orally administered to rats, and a total of 192 metabolites were characterized. These included 25 phase I metabolites, 8 hydroxylated and methylated metabolites, 57 sulfated metabolites, 74 glucuronidated metabolites, 26 sulfated and glucuronidated metabolites, and 2 glycosylated metabolites. Twenty-eight compounds were considered the in vivo “effective forms” of KAE for their antithrombotic activity. Network pharmacology, molecular docking, and molecular dynamics simulations collectively predict that these “effective forms” may exert antithrombotic effects by suppressing the SRC/PI3K/AKT pathway. This study provides a foundation for a better understanding of the in vivo “effective forms” and mechanisms underlying KAE’s antithrombotic activity, which is essential for understanding of “hexue” traditional efficacy of *C. orbiculatus*.

## 1. Introduction

Cardiovascular diseases (CVDs) are the leading cause of death worldwide, and their critical lethal factor is excessive thrombus formation [1]. Thrombus is typically triggered by the imbalanced coagulation processes, abnormal platelet aggregation, and dysfunctional fibrinolysis system [2,3,4]. At present, a series of antithrombotic drugs, such as aspirin, clopidogrel, and ticagrelor, have been used clinically for the treatment of thrombus formation. But the side effects of these antithrombotic drugs, including intracranial hemorrhage, gastric bleeding, and palpitation, result in many inconveniences and limitations in clinical use [5,6,7]. Therefore, it is imperative to study safe and effective antithrombotic drugs.

Traditional Chinese Medicines (TCMs) are employed for the treatment of CVDs due to their advantages of limited adverse effects, active-target diversity, and significant efficiency [8]. Pharmacological studies showed that flavonoid-enriched TCMs like *Ginkgo biloba* L. [9], *Carthamus tinctorius* L. [10], and *Citrus reticulata* Blanco [11] have been utilized clinically for the treatment of thrombotic diseases with minimal adverse effects, which have also demonstrated significant cardiovascular protective effects attributed to flavonoids [12,13,14]. For example, quercetin, catechin, and kaempferol can reduce platelet aggregation by reducing oxidative stress and blocking triphosphopyridine nucleotide-oxidase (NADPH-oxidase) [15,16]. Therefore, it has been an important research direction to search for safe and effective antithrombotic drugs from TCMs with flavonoids.

Kaempferitrin (Kaempferol-3, 7-*O*-*α*-L-dirhamnopyranoside, KAE) is a natural flavonol dirhamnopyranoside and is commonly found in many TCMs, such as *Rosa laevigata* Michx. [17], *Vicia amoena* var*. angusta* [18], and *Celastrus orbiculatus* Thunb. [19]. It is also found in medicinal and edible plants, including *Houttuynia cordata* Thunb. [20] and *Siraitia grosvenori* Swingle [21]. KAE possesses various pharmacological properties, including anti-inflammatory, anticonvulsant, immunostimulatory, antidepressant, and antitumor properties [22]. In clinical practice, KAE is widely employed for treating renal inflammation and fibrosis as well as regulating blood glucose levels [23]. In our previous study, the active fraction of *C. orbiculatus* fruit had significant antithrombotic activity and would cause a lower bleeding tendency [19]. The chemical analysis revealed that KAE is the primary component of the active fraction of *C. orbiculatus*. Therefore, it is essential to investigate whether KAE has antithrombotic activity and how it exerts an antithrombotic effect.

Flavonoid glucosides are usually metabolized to many metabolites after oral administration, and these produced metabolites are absorbed more easily than their parent compounds. So the blood concentrations of the metabolites are usually much higher than those of the parent compounds [24]. Moreover, some metabolites have various bioactivities similar to their parent compounds [25]. Therefore, the effective forms of flavonoids are not necessarily their natural phytochemical forms but also their metabolites, and more researchers have begun to pay attention to the bioactivity of flavonoid metabolites [26]. Because it is not known whether KAE acts as its original form or as its metabolites, it is necessary to investigate the effective forms of KAE for antithrombotic activity.

In this study, the antithrombotic effects of KAE were initially evaluated using a rat arterial thrombosis model by measuring thrombus wet weight, coagulation parameters, and platelet aggregation rates. Subsequently, the “effective forms” of KAE responsible for its antithrombotic activity were identified by profiling the metabolites of KAE using UHPLC-Q-Exactive Orbitrap MS technology. Finally, the mechanism of antithrombotic action was preliminarily studied using network pharmacology, molecular docking, and molecular dynamics simulation. The findings of this study will contribute to elucidating the antithrombotic effects, effective forms, and mechanism of KAE, and lay a foundation for developing KAE as a potential antithrombotic drug in future clinical therapy.

## 2. Results

### 2.1. Antithrombotic Activities of KAE

**Figure 1:**
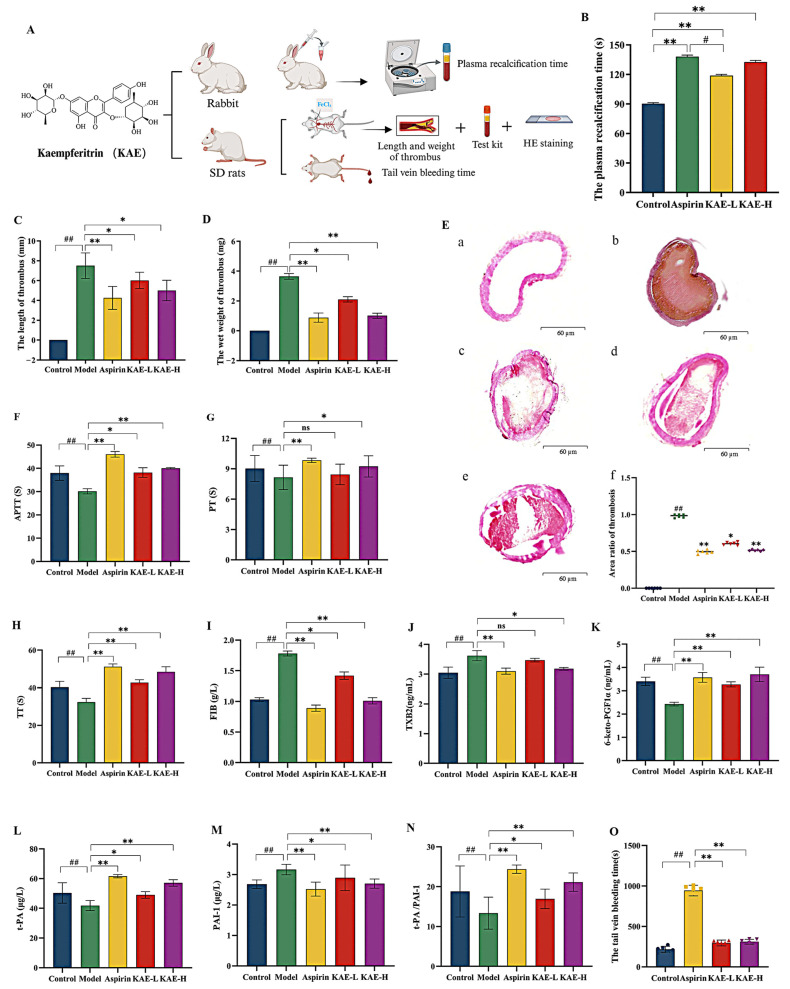
Antithrombotic activity of KAE. (**A**) Experimental design. (**B**) Anticoagulant activity of KAE on plasma recalcification time in rabbits. (**C**) Length of thrombus. (**D**) Wet weight of thrombus. (**E**) Pathological photomicrograph of thrombus (**a**: Control; **b**: Model; **c**: Aspirin; **d**: KAE-L; **e**: KAE-H; **f**: Area ratio of thrombosis). (**F**–**N**) APTT, PT, TT, FIB, TXB_2_, 6-keto-PGF1*α*, t-PA, PAI-1, and t-PA/PAI-1 levels. (**O**) The tail vein bleeding time. *n* = 6. Results are presented as mean ± SD. ^#^ *p* < 0.05, ^##^ *p* < 0.01 vs. control group, * *p* < 0.05, ** *p* < 0.01 vs. model group. ns: no significant. SD rats: Sprague-Dawley rats; KAE-L: low-dose of KAE; KAE-H: high-dose of KAE; APTT: activated partial thromboplastin time; PT: prothrombin time; TT: thrombin time; FIB: fibrinogen; TXB_2_: thromboxane B2; 6-keto-PGF1*α*: 6-keto-prostaglandin F1*α*; t-PA: tissue-type plasminogen activator; PAI-1: plasminogen activator inhibitor.

To assess the antithrombotic activities of KAE, the rabbit plasma recalcification test was first conducted, and PRT was measured. Then, the FeCl_3_-induced carotid arterial bypass thrombosis rat model was established, and thrombus length, thrombus wet weight, and four coagulation indices, including activated partial thromboplastin time (APTT), prothrombin time (PT), thrombin time (TT), and fibrinogen (FIB), were detected in plasma to manifest the KAE antithrombotic activity in vivo. Meanwhile, the levels of TXA_2_ and 6-keto-PGF1*α* were detected to evaluate the regulation of platelet function. The levels of t-PA and PAI-1 were examined to evaluate the fibrinolytic system function. Because the induction of bleeding tendency is the most common side effect of antithrombin agents, the effect of KAE on the tail vein bleeding time in rats was also investigated. The antithrombotic experiment design is shown in Figure 1A.

#### 2.1.1. Plasma Recalcification Time (PRT) Measurement

To study the activity of KAE on blood coagulation, the PRT was measured, which is an easy way of monitoring the general situation of coagulation. As shown in Figure 1B and Table S1, the PRT of KAE-L and KAE-H groups were 118.51 ± 1.75 s and 131.34 ± 2.50 s, respectively, which were significantly prolonged compared with the control group (90.19 ± 0.78 s) (*p* < 0.01). Moreover, the PRT of the KAE-H group was similar to the aspirin group (138.56 ± 2.41 s), and there was no significant difference observed (*p* > 0.01). The study indicates that KAE has certain anticoagulant effects in vitro.

#### 2.1.2. In Vitro Inhibitory Effect of KAE on Platelet Aggregation

The inhibitory effect of KAE on adenosine diphosphate (ADP)-induced platelet aggregation was shown in Table 1. KAE exerted dose-dependent inhibition on ADP-triggered platelet aggregation: the platelet aggregation rate of the KAE-L and KAE-H groups was 21.01 ± 0.75% and 15.34 ± 0.42%, respectively, which was significantly reduced compared with the control group (53.53 ± 1.30%) (*p* < 0.01). Moreover, the platelet aggregation inhibition rate of the KAE-H group (72.87 ± 0.25%) was similar to that of aspirin (74.66 ± 1.30%), and there was no significant difference observed. KAE exhibited an inhibitory effect on platelet aggregation induced by ADP.

#### 2.1.3. Evaluation of the In Vivo Antithrombotic Activity of KAE

(1) The length and wet weight of thrombus measurement: The FeCl_3_-induced rat carotid arterial thrombus model was used to investigate the effect of KAE on arterial thrombosis. As shown in Figure 1C,D and Table S2, the length and the wet weight of the thrombus in the rat model group were 7.50-fold and 3.65-fold compared to those of the control group (*p* < 0.01). The length of the thrombus in the KAE-L and KAE-H groups was 6.02 mm and 5.00 mm, which were obviously decreased compared with the model group (7.50 mm) (*p* < 0.05). The wet weight of the thrombus in the KAE-L and KAE-H groups was 2.10 mg and 1.01 mg, which were obviously decreased compared with the model group (3.65 mg) (*p* < 0.05). This result suggests that KAE has antithrombotic activity in vivo.

Moreover, the pathological examination results of the carotid artery with thrombosis for each group are shown in Figure 1E. The area ratio of thrombosis in different groups was calculated by Image Pro Plus (version 7.1). Six animals from each group were randomly chosen in this process. The area of each thrombosis section was the mean of at least 6 views at ×400 by using a microscope. The area ratio in each group was equal to the value of the area of the model group, which was divided by the area of the groups treated with aspirin or KAE. As a result, there was no occlusion of the carotid artery observed in the control group, Figure 1(Ea). On the contrary, the complete occlusion of the carotid artery was observed in the model group, Figure 1(Eb). Rats in the aspirin, KAE-L, and KAE-H groups exhibited a similar red mixed thrombus, Figure 1(Ec–Ee), and the area ratio of thrombosis was 0.50, 0.61, and 0.53, respectively, Figure 1(Ef). Therefore, KAE has the ability to reduce thrombus burden/size.

(2) Coagulation parameters detection: As shown in Figure 1F–H and Table S2, the APTT, PT, and TT of the model group rats were reduced compared with those of the control group (*p* < 0.01). These coagulation parameters were increased in the aspirin group compared with those of the control group (*p* < 0.01). FIB was increased in the model group but decreased in the aspirin group compared with that of the control group (Figure 1I and Table 1) (*p* < 0.01). KAE-L and KAE-H had prolonged APTT, PT, and TT, and reduced the FIB content compared with that of the model group (*p* < 0.05). The findings demonstrated that KAE exhibits antithrombotic properties through its ability to significantly prolong APTT, PT, and TT, while concurrently decreasing FIB levels, thus effectively modulating the coagulation system.

(3) TXB_2_ and 6-keto-PGF1*α* detection: As shown in Figure 1J,K and Table S2, KAE-H could effectively down-regulate the TXB_2_ content (3.18 ± 0.05 ng/mL) and up-regulate the 6-keto-PGF1*α* content (3.70 ± 0.31 ng/mL) compared with that of the model rats (3.62 ± 0.17 ng/mL and 2.43 ± 0.08 ng/mL) (*p* < 0.01). In addition, there was no obvious difference between the KAE-H group and the aspirin group with the contents of TXB_2_ and 6-keto-PGF1*α* (*p* > 0.05). This finding suggests that KAE demonstrates antithrombotic activity by modulating platelet function.

(4) t-PA and PAI-1 detection: As shown in Figure 1L–N and Table S2, the content of t-PA in model rats (41.83 ± 3.42 μg/L) was 0.83-fold that of the control group (50.33 ± 6.90 μg/L) (*p* < 0.01), whereas the content of PAI-1 in the model rats (3.16 ± 0.17 μg/L) was markedly higher than that of the control group (2.68 ± 0.14 μg/L) (*p* < 0.01). KAE-L and KAE-H significantly increased the content of t-PA and reduced the content of PAI-1. Indeed, KAE-H also increased the ratio of t-PA/PAI-1 compared with that of the model group (*p* < 0.01). The effect of KAE-H on t-PA and PAI-1 was close to that of the aspirin group (*p* > 0.05). This study suggests KAE exerts antithrombotic effects by activating the fibrinolysis system function.

#### 2.1.4. The Rat Tail Vein Bleeding Time Measurement

To investigate the side effects of KAE on bleeding, the tail vein bleeding time was measured on the FeCl_3_-induced rat arterial thrombosis model. As shown in Figure 1O and Table S3, the tail vein bleeding time was increased 4.34-fold in the aspirin group compared with that of the control group. KAE-L (295.75 ± 36.72 s) and KAE-H (311.50 ± 29.43 s) produced less bleeding time than that of the aspirin group (944.25 ± 65.52 s). The research indicated that KAE had a marked antithrombotic effect with less bleeding time compared with aspirin.

Additionally, to further control the multiple comparison error, the Tukey honestly significant difference (Tukey HSD) global correction analysis for all endpoints was conducted, and the results are shown in Table S4. The normality and homogeneity of variance for ANOVA have been verified through the Shapiro–Wilk and Brown–Forsythe tests, and the results are shown in Tables S5 and S6. Meanwhile, a post hoc power analysis for the primary endpoints (thrombus length, thrombus weight, APTT, PT, TT, and FIB) in the FeCl_3_-induced rat arterial thrombosis model was carried out, and the results are shown in Table S7.

### 2.2. Analysis of the Metabolites of KAE

A total of 192 metabolites of KAE were characterized in this study (Table 2 and Table S8). We detected 124, 77, and 42 metabolites in the rat urine, plasma, and feces samples, respectively. These 192 metabolites were identified as 56 potential new compounds that could not be found in the SciFinder database (Table 2) and 136 new metabolites of KAE. To facilitate the description of these metabolites, they were divided into five groups based on their metabolic reactions: phase I metabolites (25), hydroxylated and methylated metabolites (8), sulfated metabolites (57), glucuronidated metabolites (74), sulfated and glucuronidated metabolites (26), and glycosylated metabolites (2). The detailed metabolite information is shown in Table 2 and Table S8, and the metabolites’ extracted ion chromatograms (EICs) are presented in Figures S1–S71 in the Supplementary Materials.

#### 2.2.1. Phase I Metabolites Characterization

**Figure 2:**
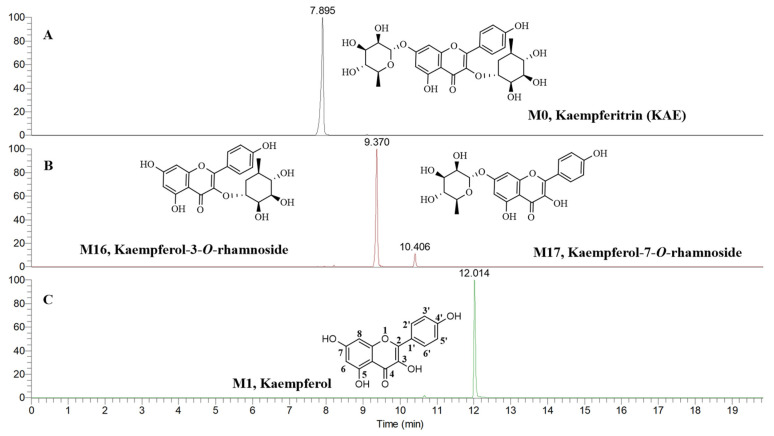
Extracted ion chromatograms (EICs) of kaempferitrin (KAE) and its deglycosylated metabolites. (**A**) M0, kaempferitrin. (**B**) M16, kaempferol-3-*O*-rhamnoside; M17, kaempferol-7-*O*-rhamnoside. (**C**) M1, kaempferol.

Twenty-five phase I metabolites (M1–M25) were characterized in the present study, which was composed of deglycosylated (3), hydroxylated (3), hydrogenated and dehydroxylated (18), and demethylated (1) metabolites. As shown in Figure 2, M0, M1, M16, and M17 were unambiguously identified as KAE, kaempferol, kaempferol-3-*O*-rhamnoside, and kaempferol-7-*O*-rhamnoside, respectively, by comparison with the standard references. Therefore, KAE (Figure 2A) firstly removed a rhamnose group to become kaempferol-3-*O*-rhamnoside or kaempferol-7-*O*-rhamnoside (Figure 2B), and then further removed another rhamnose group to become its aglycone of kaempferol (Figure 2C). It can be seen that the relative content of kaempferol-3-*O*-rhamnoside is more than kaempferol-7-*O*-rhamnoside (Figure 2B), so the deglycosylation of C-7 is easier than that of C-3.

**Figure 3:**
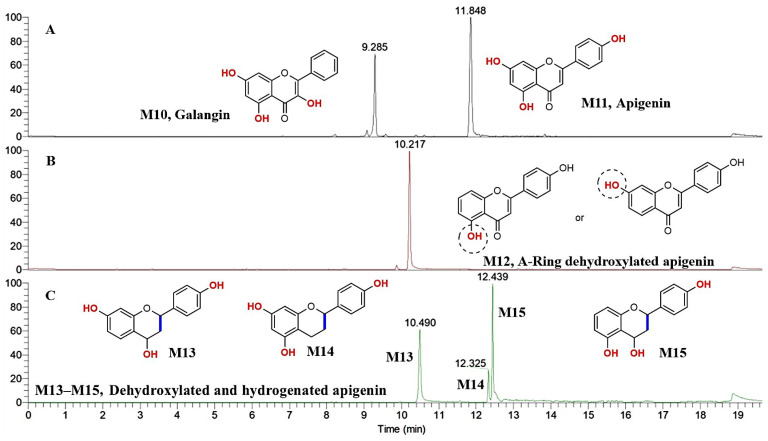
Extracted ion chromatograms (EICs) of M10–M15, red: probably varied hydroxyl group; blue: probably varied hydrogen bond; 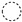: unchanged hydroxyl group. (**A**) M10, galangin; M11, apigenin. (**B**) M12, A-ring dehydroxylated apigenin. (**C**) M13–M15, dehydroxylated and hydrogenated apigenin.

Kaempferol can form galangin (M10) and apigenin (M11) by dehydroxylation metabolic reaction (Figure 3A). As shown in Figure S1A,B, M10 and M11 showed [M–H]^−^ at *m*/*z* 269.04575 and *m*/*z* 269.04568, and their molecular formula (MF) was predicted to be C_15_H_10_O_5_, molecular weight (MW) was 270 Da, and the double bond equivalents (DBE) were 11. Compared with kaempferol, the MF of M10 and M11 was reduced by one oxygen atom, the MW was decreased by 16 Da, and the DBE was unchanged. Therefore, M10 and M11 were tentatively identified to be the dehydroxylated metabolites of kaempferol. In their MS^2^ spectra, a pair of diagnostic fragment ions of *m*/*z* 151.00 (C_7_H_3_O_4_, ^1,3^A^−^) and *m*/*z* 117.03 (C_8_H_5_O, ^1,3^B^−^) were observed, which indicated the A-ring of kaempferol was unchanged. In other words, the dehydroxylation occurred in the B- or C-ring of kaempferol. Furthermore, with the help of the CLog *P* values, M10 and M11 were tentatively determined as galangin (CLog *P*: 2.76403) and apigenin (CLog *P*: 2.90529). Because the relative content of apigenin (M11) is more than that of galangin (M10), kaempferol is more easily to undergo dehydroxylation at C-3 to form apigenin.

Apigenin can also be metabolized to its dehydroxylated metabolites (Figure 3B,C). M12 showed [M–H]^−^ at *m*/*z* 253.05070, and its MF was predicted as C_15_H_10_O_4_, MW was 254 Da, and the DBE was 11. The fragment ions at *m*/*z* 224.04802, *m*/*z* 209.06064, *m*/*z* 180.05786, *m*/*z* 135.00900 (C_7_H_3_O_3_, ^1,3^A^−^), *m*/*z* 117.03467 (C_8_H_5_O, ^1,3^B^−^), and *m*/*z* 91.01903 were observed in the MS^2^ spectrum (Figure S1C). Therefore, it was determined that dehydroxylation occurred in the A-ring of apigenin.

**Figure 4:**
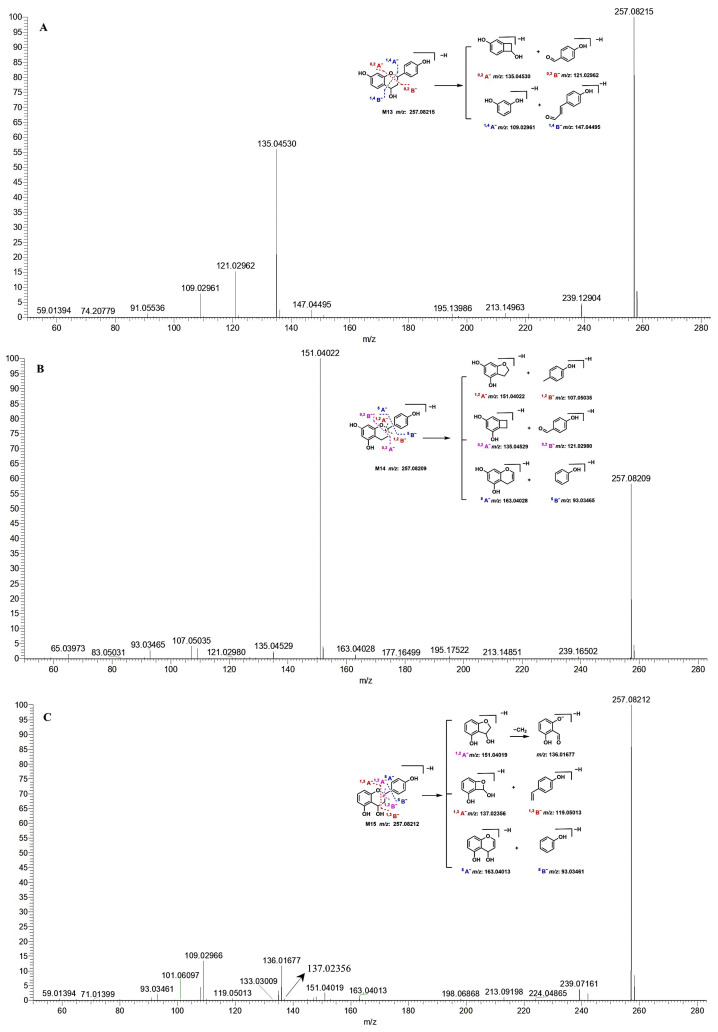
Secondary mass spectrometry (MS^2^) data of M13–M15 and their cleavage pathways in the negative ion model. (**A**) M13. (**B**) M14. (**C**) M15.

M13–M15 all exhibited [M–H]^−^ at *m*/*z* 257.082 (Figure 4), and their MF was predicted to be C_15_H_14_O_4_, MW was 258 Da, and the DBE was 9. Therefore, M13–M15 were tentatively identified to be the dehydroxylated and dihydrogenated metabolites of apigenin. In the structure of apigenin, the two double bonds of the C-ring were easy to reduce, so the probability of hydrogenation was high in the C-ring. M13 showed two pairs of characteristic fragment ions at *m*/*z* 135.04530 (C_8_H_7_O_2_, ^0,2^A^−^) and *m*/*z* 121.02962 (C_7_H_5_O_2_, ^0,2^B^−^); *m*/*z* 147.04495 (C_9_H_7_O_2_, ^1,4^B^−^) and *m*/*z* 109.02961 (C_6_H_5_O_2_, ^1,4^A^−^) in the MS^2^ spectrum (Figure 4A). Therefore, M13 was tentatively identified as 4, 7, 4′-trihydroxylated dihydrogenated flavone. In the MS^2^ spectrum of M14, the *m*/*z* 151.04022 (C_8_H_7_O_3_, ^1,2^A^−^) was the base peak ion, which indicated that it was generated from the parent ion of *m*/*z* 257.08209 easily. This ion was produced by the cleavage of bonds 1 and 2 as speculated, and its complementary ion of *m*/*z* 107.05035 (C_7_H_7_O, ^1,2^B^−^) was also observed. Additionally, the other pair of ions at *m*/*z* 163.04028 (C_9_H_7_O_3_, ^5^A^−^) and *m*/*z* 93.03465 (C_6_H_5_O, ^5^B^−^) were observed (Figure 4B). So M14 was tentatively determined to be 5, 7, 4′-trihydroxylated dihydrogenated flavone. M15 displayed the fragment ions of *m*/*z* 163.04013 (C_9_H_7_O_3_, ^5^A^−^), *m*/*z* 151.04019 (C_8_H_7_O_3_, ^1,2^A^−^), *m*/*z* 136.01677 (C_7_H_4_O_3_, ^1,2^A^−^ − CH_3_), *m*/*z* 137.02356 (C_7_H_5_O_3_, ^1,3^A^−^), *m*/*z* 133.03009 (C_7_H_7_O_2_, ^1,2^A^−^ − H_2_O), *m*/*z* 119.05013 (C_8_H_7_O, ^1,3^B^−^), *m*/*z* 109.02966 (C_6_H_5_O_2_, ^2,4^A^−^), *m*/*z* 101.06097 (C_8_H_5_, ^1,3^B^−^ − H_2_O), and *m*/*z* 93.03461 (C_6_H_5_O, ^5^B^−^) in the MS^2^ spectrum (Figure 4C). Therefore, M15 was tentatively identified as 4, 5, 4′-trihydroxylated dihydrogenated flavone. The retention time of M15 was 12.439 min, which was longer than that of M13 (t*_R_*: 10.490 min) and M14 (t*_R_*: 12.325 min), which suggests that the polarity of M15 was smaller than that of M13 and M14. This may be explained by the hydroxyl group substituted in the C-5 position could form an intramolecular hydrogen bond with the hydroxyl group in the C-4 position, which would decrease the polarity of M15. The cleavage pathways of M13–M15 in the negative ion model are presented in Figure 4.

#### 2.2.2. Phase II Metabolites Characterization

**Figure 5:**
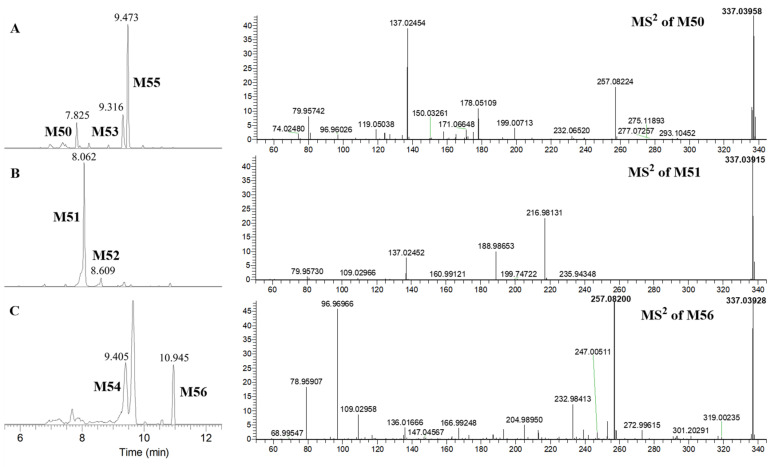
Extracted ion chromatograms (EICs) of M50–M56 in rat urine (**A**), feces (**B**), and plasma (**C**), and secondary mass spectrometry (MS^2^) data of M50, M51, and M56.

M50 presented [M–H]^−^ at *m*/*z* 337.03958 in the MS spectrum and the fragment ions of *m*/*z* 257.08224, *m*/*z* 199.00713 (C_8_H_7_SO_4_, ^1,3^B^−^ + SO_3_), *m*/*z* 137.02454 (C_7_H_5_O_3_, ^1,3^A^−^), *m*/*z* 119.05038 (C_8_H_7_O, ^1,3^B^−^), *m*/*z* 96.96026, and *m*/*z* 79.95742 (SO_3_) in the MS^2^ spectrum (Figure 5A). Therefore, M50 was tentatively identified as the sulfated metabolite of M15, and sulfation occurred in the B-ring, namely, 4, 5, 4′-trihydroxylated dihydrogenated flavone-4′-*O*-sulfate.

M51 had [M–H]^−^ at *m*/*z* 337.03915 in the MS spectrum and the fragment ions of *m*/*z* 216.98131 (C_7_H_5_SO_6_, ^1,3^A^−^ + SO_3_), *m*/*z* 137.02452 (C_7_H_5_O_3_, ^1,3^A^−^), *m*/*z* 188.98653 (C_6_H_5_SO_5_, ^1,3^A^−^ − CO + SO_3_), *m*/*z* 109.02966 (C_6_H_5_O_2_, ^1,3^A^−^ − CO), and *m*/*z* 79.95730 (SO_3_) in the MS^2^ spectrum (Figure 5B). M53 showed [M–H]^−^ at *m*/*z* 337.03903 in the MS spectrum and the fragment ions of *m*/*z* 257.08215, *m*/*z* 216.98151 (C_7_H_5_SO_6_, ^1,3^A^−^ + SO_3_), *m*/*z* 137.02457 (C_7_H_5_O_3_, ^1,3^A^−^), *m*/*z* 119.05041 (C_8_H_7_O, ^1,3^B^−^), *m*/*z* 96.96020, and *m*/*z* 79.95741 (SO_3_) in the MS^2^ spectrum (Figure S2A). M54 displayed [M–H]^−^ at *m*/*z* 337.03928 in the MS spectrum and the fragment ions of *m*/*z* 257.08209, *m*/*z* 216.98164 (C_7_H_5_SO_6_, ^1,3^A^−^ + SO_3_), *m*/*z* 137.02449 (C_7_H_5_O_3_, ^1,3^A^−^), *m*/*z* 119.05032 (C_8_H_7_O, ^1,3^B^−^), *m*/*z* 96.96004, and *m*/*z* 79.95739 (SO_3_) in the MS^2^ spectrum (Figure S2B). Therefore, M51, M53, and M54 were tentatively identified as the sulfated metabolites of M15, and sulfation is presumed to occur in the A- or C-ring. However, there are only two hydroxyl groups in the A- and C-rings of M15, and three sulfated metabolites were detected. This may be explained by the C-4 being a chiral carbon that leads to stereoisomer formation. In addition, the tR of M51, M53, and M54 was 8.062, 9.312, and 9.405 min, respectively, which suggested the polarity of M51 was greater than that of M53 and M54, and the polarity of M53 and M54 was similar. The polarity of the C-5 substituted sulfonate group in the compound is much larger than that of the C-4 substituted sulfonate group. Therefore, M51 was tentatively identified as 4, 5, 4′-trihydroxylated dihydrogenated flavone-5-*O*-sulfate, and M53 and M54 were tentatively identified as 4, 5, 4′-trihydroxylated dihydrogenated flavone-4-*O*-sulfate isomers.

M52 showed [M–H]^−^ at *m*/*z* 337.03943 in the MS spectrum and the fragment ions of *m*/*z* 257.08206, *m*/*z* 215.00218 (C_8_H_7_SO_5_, ^0,2^A^−^ + SO_3_), *m*/*z* 135.04532 (C_8_H_7_O_2_, ^0,2^A^−^), *m*/*z* 121.02961 (C_7_H_5_O_2_, ^0,2^B^−^), *m*/*z* 96.96017, and *m*/*z* 80.96252 in the MS^2^ spectrum (Figure S2C). M55 exhibited [M–H]^−^ at *m*/*z* 337.04041 in the MS spectrum and the fragment ions of *m*/*z* 257.08206, *m*/*z* 147.04529 (C_9_H_7_O_2_, ^1,4^B^−^), *m*/*z* 135.04532 (C_8_H_7_O_2_, ^0,2^A^−^), *m*/*z* 121.02966 (C_7_H_5_O_2_, ^0,2^B^−^), *m*/*z* 109.02956(C_6_H_5_O_2_, ^1,4^A^−^), *m*/*z* 96.96976, and *m*/*z* 79.95742 (SO_3_) in the MS^2^ spectrum (Figure S2D). According to the fragment information, the structure of the parent nucleus of M52 and M55 was determined as M13. Furthermore, M52 was tentatively identified as 4, 7, 4′-trihydroxylated dihydrogenated flavone-4/7-O-sulfate based on the characteristic ion of *m*/*z* 215.00218 (C_8_H_7_SO_5_, ^0,2^A^−^ + SO_3_), while the sulfonate group substituted position of M55 was not exactly identified.

M56 showed [M–H]^−^ at *m*/*z* 337.03928 in the MS spectrum and the fragment ions of *m*/*z* 257.08200, *m*/*z* 136.01666 (C_7_H_4_O_3_, ^1,2^A^−^ − CH_3_), *m*/*z* 109.02958 (C_6_H_5_O_2_, ^2,4^A^−^), *m*/*z* 96.96966, and *m*/*z* 79.95907 (SO_3_) in the MS^2^ spectrum (Figure 5C). Based on the fragment ions, M56 was tentatively identified as the sulfated metabolite of M15, and the precise sulfonate group substituted position was not confirmed.

Methylated dihydrogenated kaempferol sulfate (M72). The EICs of M72 are presented in Figure S28. In negative ion mode, the presented at *m*/*z* 383.04306 ([M–H]^−^) in the MS spectral, MF was predicted to be C_16_H_16_O_9_S, and the MS^2^ showed the fragment ions of *m*/*z* 303.08752, *m*/*z* 124.01672, *m*/*z* 151.04062 (C_8_H_7_O_4_, ^0,2^A^−^), *m*/*z* 193.03546, and *m*/*z* 79.95739, of which *m*/*z* 79.95739 was the sulfated characteristic fragment ion, compared with kaempferol, the MF increased a unit of CH_6_, DBE was 9, it was presumed that methylation and C-ring reduction occurs, and methylation occurs in the A-ring, the compound was identified as the A-ring methylated dihydrogenated kaempferol sulfate. Entering C_16_H_16_O_9_S into Scifinder retrieved 40 compounds with the same formula, and the presumed compound was not found; hence, it was presumed to be a new compound.

Methylated dihydrogenated apigenin sulfates (M73–M75). The EICs of M73–M75 are presented in Figure S29. In the negative ion mode, the presented at *m*/*z* 367.04846, *m*/*z* 367.04810, and *m*/*z* 367.04878 ([M–H]^−^) in the MS spectra, MF was predicted to be C_16_H_16_O_8_S. M73 fragment ions of *m*/*z* 287.09277, *m*/*z* 137.02457 (C_7_H_5_O_3_, ^0,3^A^−^), *m*/*z* 149.06104, *m*/*z* 119.05026 (C_8_H_7_O, ^1,3^B^−^), *m*/*z* 199.00748 (*m*/*z* 119.05026 + SO_3_), *m*/*z* 79.95742, and *m*/*z* 96.96022 were observed by MS^2^, where *m*/*z* 79.95742 and *m*/*z* 96.96022 were the characteristic sulfide fragment ions. The signals at *m*/*z* 199.00748, *m*/*z* 137.02457, and *m*/*z* 149.06104 suggested that both methylation and sulfation occurred in the B-ring, and identified the compound as B-ring methylated dihydrogenated apigenin sulfate. C_16_H_16_O_8_S was researched using Scifinder, and a total of 30 compounds with the same formula were found. But there was no conjectured compound, conjectured may be a new compound. M74 and M75 fragment ions of *m*/*z* 287.0928 and *m*/*z* 151.0401 (C_8_H_7_O_3_, ^0,2^A^−^), *m*/*z* 216.9813 (*m*/*z* 137.0246 + SO_3_), *m*/*z* 137.0246 (C_7_H_5_O_3_, ^0,3^A^−^), *m*/*z* 135.0452 (C_8_H_7_O_2_, ^0,3^B^−^), *m*/*z* 79.9574, and *m*/*z* 96.9601 could be seen by MS^2^, on the basis of *m*/*z* 137.0246, *m*/*z* 151.0401, and *m*/*z* 215.0022, methylation was presumed to occur in the A-ring, sulfation was presumed to occur in the B-ring, and the compounds were identified as A-ring methylated dihydrogenated apigenin sulfates. Input C_16_H_16_O_8_S into Scifinder, there were 30 compounds with the same general formula, but there were no speculated compounds, which may be new compounds.

The A, C-Rings of apigenin cracking sulfates (M76–M79). The EICs of M76–M79 are presented in Figure S30. In negative ion mode, the *m*/*z* 258.99152, *m*/*z* 258.99158, *m*/*z* 258.99158, and *m*/*z* 258.99165 ([M–H]^−^) peaks were visible in the MS spectra, and MF was predicted to be C_9_H_8_O_7_S. MS^2^ showed the fragment ions of *m*/*z* 179.0350, *m*/*z* 135.0452 (C_8_H_7_O_2_, ^0,2^A^−^), *m*/*z* 96.9601, where *m*/*z* 96.9601 was the sulfation characteristic fragment ion, and the compounds were identified as A- and C-rings of apigenin cracking sulfates. Inputting C_9_H_8_O_7_S into Scifinder provided 45 compounds with the same formula as its general formula, but there were none of the presumed compound; hence, it was assumed to be a new compound.

Trihydroxylated dihydrogenated flavone disulfates (M82–M84). The EICs of M82–M84 are presented in Figure S32. In the negative ion mode, the *m*/*z* 416.99478, *m*/*z* 416.99460, and *m*/*z* 416.99460 ([M–H]^−^) were visible at the MS spectral, MF was predicted to be C_15_H_14_O_10_S_2_, M82 showed the fragment ions of *m*/*z* 337.03815, *m*/*z* 257.08224, *m*/*z* 137.02463 (C_7_H_5_O_3_, ^0,3^A^−^), *m*/*z* 119.05075 (C_7_H_5_O_2_, ^0,2^B^−^), *m*/*z* 96.96031, and *m*/*z* 79.95741, in the MS^2^ spectrum, of which *m*/*z* 96.96031 and *m*/*z* 79.95741 were sulfated characteristic fragmentation ions, so the compound was identified as 4, 5, 4′-trihydroxylated dihydrogenated flavone disulfate. M83 and M84 showed the fragment ions of *m*/*z* 135.04530 (C_8_H_7_O_2_, ^0,2^A^−^) and *m*/*z* 121.02962 (C_7_H_5_O_2_, ^0,2^B^−^); *m*/*z* 147.04495 (C_9_H_7_O_2_, ^1,4^B^−^) and *m*/*z* 109.02961 (C_6_H_5_O_2_, ^1,4^A^−^) in the MS^2^ spectrum, so the compounds were identified as 4, 7, 4′-trihydroxylated dihydrogenated flavone disulfates. When C_15_H_14_O_10_S_2_ was entered into Scifinder, six compounds with the same general formula were found, but there were no presumed compounds; hence, it was speculated that it might be a new compound.

Methylated hydrogenated kaempferol glucuronide (M129). The EICs of M129 are presented in Figure S45. In the negative ion mode, the *m*/*z* 477.10306 ([M–H]^−^) was visible at the MS spectral, MF was predicted to be C_22_H_22_O_12_, and the MS^2^ showed *m*/*z* 301.07205, *m*/*z* 286.04861, *m*/*z* 175.02481, *m*/*z* 151.00386 (C_7_H_3_O_4_, ^1,3^A^−^), *m*/*z* 149.06097 (C_9_H_9_O_2_, ^1,4^B^−^), *m*/*z* 134.03764 (C_9_H_9_O_2_ − CH_2_, B^−^), *m*/*z* 113.02457 and *m*/*z* 85.02957, of which *m*/*z* 175.02481 was a characteristic fragment ion for glucuronidation, *m*/*z* 149.06097 with *m*/*z* 134.03764, presumably methylation occur in the B-ring, removal of the MF of C_6_H_8_O_6_ and an increase in a unit of CH_4_ in the MF, compared to kaempferol with a DBE of 10, it was presumed that C-ring reduction and methylation occurs, the compound was identified as B-ring methylated hydrogenated kaempferol glucuronide. When C_22_H_22_O_12_ was entered into Scifinder, 262 compounds with the same formula were found, but there was no presumed compound, so it might be a new compound.

Methylated dihydrogenated apigenin glucuronide (M144). The EICs of M144 are presented in Figure S52. In negative ion mode, the *m*/*z* 463.12418 ([M–H]^–^) was visible at the MS spectral, MF was predicted to be C_22_H_24_O_11_, with *m*/*z* 463.1246 as the parent ion, and the MS^2^ showed the fragment ions of *m*/*z* 287.09293, *m*/*z* 175.02489, *m*/*z* 149.02437 (C_8_H_5_O_3_, ^0,2^A^−^), *m*/*z* 113.02452 and *m*/*z* 85.02954, of which *m*/*z* 175.02489 was the characteristic fragment ion for glucuronidation, removing the C_6_H_8_O_6_ molecule, compared to kaempferol, the MF was reduced by 1 oxygen atom and increased by a unit of CH_6_, the DBE was 9, it was presumed that dehydroxylation and methylation occurs, and the C-ring was fully reduced, and it was presumed that the methylation occurs in the A-ring, the compound was identified as A-ring methylated dihydrogenated apigenin glucuronide, C_22_H_24_O_11_ was entered into Scifinder and 167 compounds with the same general formula were found, but not the presumed compound, which was presumed to be a new compound.

Methylated apigenin glucuronyl sulfate (M166). The EICs of M166 are presented in Figure S63. In the negative ion mode, the *m*/*z* 539.04901 ([M–H]^−^) was visible at the MS spectral, MF was predicted to be C_22_H_20_O_14_S, and the MS^2^ showed the fragment ions of *m*/*z* 363.01804, *m*/*z* 283.06125, *m*/*z* 268.03793, *m*/*z* 263.02322, *m*/*z* 242.99701, *m*/*z* 163.04015 (C_9_H_7_O_3_, ^0,2^A^−^) and *m*/*z* 79.95739, of which *m*/*z* 79.95739 was a sulfated characteristic fragment, *m*/*z* 363.01804 and *m*/*z* 539.04901 have a difference of 176.03097 relative molecular mass. The *m*/*z* 242.9970 and *m*/*z* 163.0401 were the characteristic fragment ions of glucuronidation, removing SO_3_ and C_6_H_8_O_6_, compared with kaempferol, MF increased a unit of CH_2_ and decreased 1 oxygen atom, DBE remained unchanged, and it was presumed that hydroxylation and methylation occurred, *m*/*z* 242.9970 and *m*/*z* 163.0401 presume that sulfation occurs in the A-ring, and methylation occurs in the A-ring. The compound was identified as A-ring methylated apigenin A-ring glucuronyl B-ring sulfate. When C_22_H_20_O_14_S was input into Scifinder, four compounds with the same formula were found, but there was no presumed compound, so it was presumed that it might be a new compound.

Dehydroxylated apigenin glucuronyl sulfates (M184–M186). The EICs of M184–M186 are presented in Figure S68. In the negative ion mode, the *m*/*z* 509.03869, *m*/*z* 509.03855 and *m*/*z* 509.03870 ([M–H]^−^) were visible at the MS spectral, MF was predicted to be C_21_H_18_O_13_S, and the MS^2^ showed the fragment ions of *m*/*z* 429.0846, *m*/*z* 333.0078, *m*/*z* 253.0507, *m*/*z* 242.9969, *m*/*z* 203.0024, *m*/*z* 163.0405, *m*/*z* 113.0245, *m*/*z* 79.9573, of which *m*/*z* 175.0249 was a glucuronidation characteristic fragment ion, *m*/*z* 79.9573 was a sulfation characteristic fragment ion, *m*/*z* 242.9969, *m*/*z* 203.0024, *m*/*z* 163.0405 presumed that the sulfation occurred in the A-ring and the compounds were identified as A-ring dehydroxylated apigenin glucuronyl sulfates. Entering C_21_H_18_O_13_S into Scifinder retrieved 5 compounds with the same formula as it, and the presumed compounds were not found and were presumed to be new compounds.

Methylated dehydroxylated apiferol glucuronyl sulfate (M191). The EICs of M191 are presented in Figure S70. In the negative ion mode, the *m*/*z* 527.08582 ([M–H]^−^) was visible at the MS spectral, MF was predicted as C_22_H_24_O_13_S, and the MS^2^ showed *m*/*z* 351.05438, *m*/*z* 271.09775, *m*/*z* 199.00732, *m*/*z* 175.02495, *m*/*z* 151.04025 (C_8_H_7_O_3_, ^0,2^A^−^), *m*/*z* 137.02455 (C_7_H_5_O_3_, ^0,3^A^−^), *m*/*z* 119.05038 (C_8_H_7_O, ^1^.^3^B^−^), *m*/*z* 113.02457 and *m*/*z* 85.02956, where *m*/*z* 175.02495 was the characteristic fragmentation ion of glucuronidation. The *m*/*z* 351.05455 and *m*/*z* 271.09775 difference of 79.95680 was the characteristic locking piece of sulfate acidification. Removing C_6_H_8_O_6_ and SO_3_, compared to kaempferol, the MF was decreased by 2 oxygen atoms and increased by a unit of CH_6_, and the DBE was decreased by 2, it was presumed that dehydroxylation, C-ring reduction, and methylation occurred, presumably methylation occurred in the A-ring and sulfation occurred in the B-ring. The compound was identified as an A-ring methylated dehydroxylated apiferol glucuronyl-4′-O-sulfate. Inputting C_22_H_24_O_13_S into Scifinder revealed 1 compound with the same formula, but not the presumed compound, which was presumed to be a new compound.

The characterizations of other metabolites are shown in Sections 2.2–2.6 of the Supplementary Materials.

#### 2.2.3. Potential Metabolic Pathway

**Figure 6:**
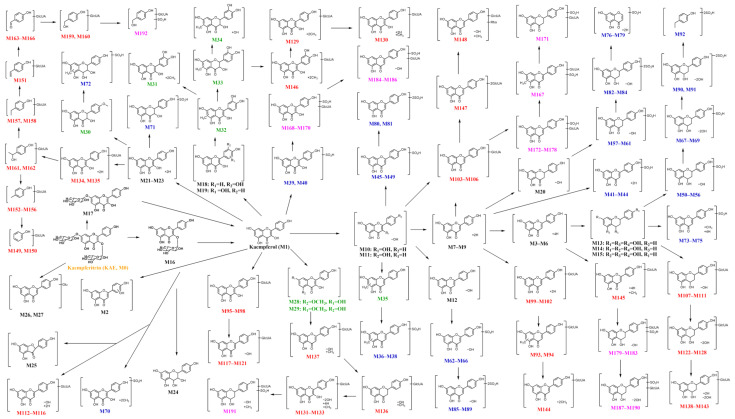
Proposed metabolic pathway of kaempferitrin (KAE) in rats. Yellow for kaempferitrin (KAE); Black for phase I metabolites, red for glucuronidated metabolites, blue for sulfated metabolites, purple for sulfated and glucuronidated metabolites, green for methylated metabolites.

In this study, the comprehensive metabolism of KAE was studied, and 192 metabolites were identified. According to the identified metabolites, the potential metabolic pathway of KAE in vivo was proposed (Figure 6). KAE was first hydrolyzed to form its deglycosylated metabolites, i.e., kaempferol-3-*O*-rhamnoside, kaempferol-7-*O*-rhamnoside, and kaempferol in the gastrointestinal tract after being ingested by rats. Kaempferol can undergo a dehydroxylation metabolic reaction and produce dehydroxylation metabolites, and dehydroxylation can occur one–three times. Kaempferol can also undergo a methylation metabolic reaction and produce methylation metabolites. Based on these dehydroxylated metabolites and methylated metabolites, sulfation and glucuronidation metabolic reactions took place because the dehydroxylation and methylation that occurred in the KAE provided more sites. The hydroxy group, the double bonds of the C-ring, and the carbonyl group of kaempferol are vulnerable positions for metabolism. The double bonds of the C-ring of kaempferol and apigenin can be reduced to their corresponding flavanones. Furthermore, the carbonyl group of these produced flavanones was subject to a reduction to form flavanols.

Sulfation and glucuronidation are considered to be the main phase II metabolic reactions of flavones. In the present study, 57 sulfated containing 13 disulfated metabolites, 74 glucuronidated metabolites, and 26 both sulfated and glucuronidated metabolites were detected. During the identification of the sulfated metabolites, the sulfonyl group conjugated sites of some sulfated metabolites could be assigned with the help of the sulfonyl group-conjugated fragments that appeared in the MS^2^ spectrum. Based on the identification results of the sulfated metabolites, the skeletal structures of the sulfated metabolites with the same MF values were very different. The diversity of the metabolites reflected the complexity of the in vivo metabolism process of KAE. However, the glucuronyl group conjugated sites were not determined because the glucuronyl group was easily cleaved from their parent compounds and could not be observed in the MS^2^ spectrum. As mentioned above, the metabolism of KAE in rats is complex, and most of the metabolites are formed by multiple steps of metabolic reactions. The main metabolic reactions of KAE contained deglycosylation, hydroxylation, methylation, hydrogenation, dehydroxylation, sulfation, and glucuronidation.

M1 (kaempferol), M7 (naringenin), M11 (apigenin), M16 (kaempferol-3-*O*-rhamnoside), M17 (kaempferol-7-*O*-rhamnoside), and M18 (quercetin) have anti-atherosclerotic, anti-thrombotic, and anti-inflammatory activities and are found in high levels in metabolites [27,28,29]. Thus, the bioactivity of KAE might be exerted by both/or KAE itself and/or any of its metabolites. Therefore, the metabolism of KAE plays an important role in producing pharmacological actions, and the produced metabolites with bioactivities can be considered as the “effective forms” of KAE in vivo. One important mechanism of action of KAE may be additive or/and synergistic effects, namely, these “effective forms” consisting of KAE and/or its many metabolites may act on the same targets and may exert efficacy through the addition of their blood concentrations because of their very similar structures. These findings enhance our understanding of the “effective forms” of KAE and its mechanisms of action.

### 2.3. Network Pharmacology of KAE and Its Metabolites

#### 2.3.1. Screening “Effective Forms” and Their Antithrombotic Targets and Construction of the “Compound-Target” Network

**Figure 7:**
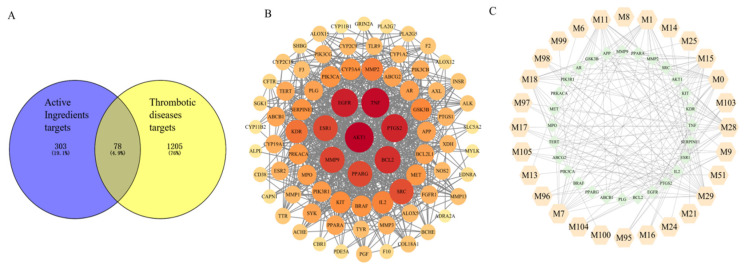
Effective forms of kaempferitrin and their antithrombotic targets with network pharmacology. (**A**) Venn diagram of compound targets and thrombotic disease targets. (**B**) Protein–protein interaction (PPI) network. (**C**) Component–target network. (28 green nodes represent antithrombotic targets, and 28 orange nodes represent effective forms).

**Figure 8:**
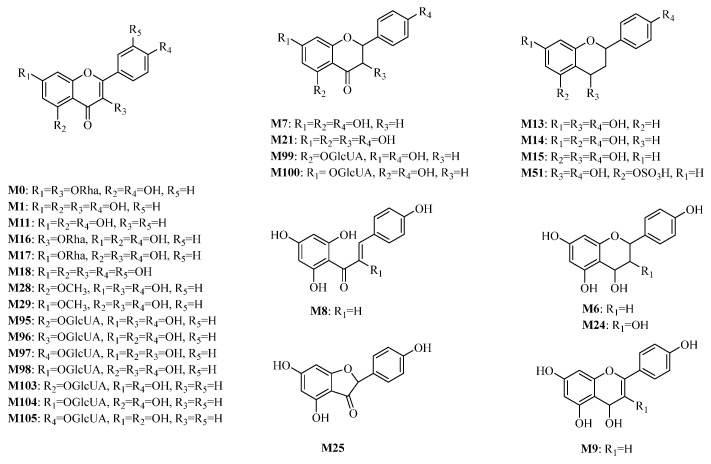
Twenty-eight effective forms (kaempferitrin and its 27 metabolites).

Thirty compounds (KAE and 29 metabolites) were confirmed to have a probability value > 0.1, and 303 targets were obtained based on SwissTarget Prediction. A total of 1283 targets for thrombotic diseases were obtained based on the GeneCards and OMIM databases. As a result, 78 antithrombotic targets were obtained through cross-tabulation analysis with compound targets and thrombotic disease targets (Figure 7A). The interaction of the selected antithrombotic targets was analyzed based on the STRING database, and a PPI network was constructed (Figure 7B). The PPI network included 78 nodes, and AKT1, TNF, EGFR, PTGS2, BCL2, MMP9, ESR1, PPARG, SRC, KDR, MMP2, IL2, GSK3B, KIT, BRAF, SERPINE1, PIK3CA, MET, PIK3R1, PRKACA, MPO, AR, ABCG2, APP, PLG, ABCB1, PPARA, and TERT were the top 28 nodes with the largest degree values (degree ≥ 21) (Table S9), which are the core targets. The antithrombotic component–target network of KAE was drawn (Figure 7C). Therefore, the 28 “effective forms” including M0, M1, M6, M7, M8, M9, M11, M13, M14, M15, M16, M17, M18, M21, M24, M25, M28, M29, M51, M95, M96, M97, M98, M99, M100, M103, M104, and M105 (Figure 8) that interact with these core targets may play an important role in the antithrombotic process.

#### 2.3.2. Signaling Pathway Enrichment Analysis with GO and KEGG

**Figure 9:**
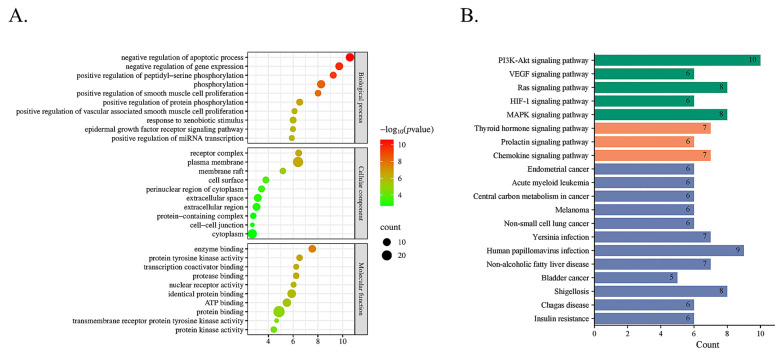
Results of GO (**A**), and KEGG (**B**) enrichment analysis, green: environmental information processing, orange: organismal systems, blue: human diseases. GO: Gene Ontology; KEGG: Kyoto Encyclopedia of Genes and Genomes.

To investigate the mechanism of effective forms in the treatment of thrombotic disease, 28 core targets were enriched by GO and KEGG analysis. The GO analysis gave 335 terms, including 249 terms for biological processes (BP), 29 terms for cell composition (CC), and 57 terms for molecular function (MF) (Tables S10–S12). The top 10 enriched BP, CC, and MF terms are shown in Figure 9A and Figure S72. BP was mainly associated with negative regulation of apoptotic process and gene expression, positive regulation of peptidyl-serine phosphorylation, vascular-associated smooth muscle cell proliferation, and miRNA transcription, the influence of the response to xenobiotic stimulus, and epidermal growth factor receptor signaling pathway. According to the results of the CC, the major targets were located on the receptor complex, plasma membrane, membrane raft, cell surface, perinuclear region of cytoplasm, extracellular space, extracellular region, protein-containing complex, cell–cell junction, and cytoplasm. MF was mainly related to the activity and binding of proteins.

KEGG pathway analysis was used to determine the pathways that were significantly altered by effective forms in thrombotic disease treatment. A total of 107 pathways were significantly enriched (*p* < 0.05, Table S13), and the top of 20 highly enriched pathways are shown in Figure 9B. Then, a C-T-P network with 431 nodes and 1794 edges was built using Cytoscape 3.10 based on the top of 20 pathways (Supplementary Figure S73). The results showed that the mechanism of effective forms in thrombotic disease therapy may be mostly associated with PI3K-AKT, VEGF, thyroid hormone, HIF-1, and MARK signaling pathways. The visualized PI3K-AKT signaling pathway was established utilizing “pathview” in R 4.0.0 (Supplementary Figure S74), which presented the role of effective forms in targeting this pathway. These findings suggest that effective forms may exert anti-thrombotic effects through various mechanisms, and the PI3K-AKT signaling pathway can play a crucial role in their action.

### 2.4. Molecular Docking Validation Analysis

**Figure 10:**
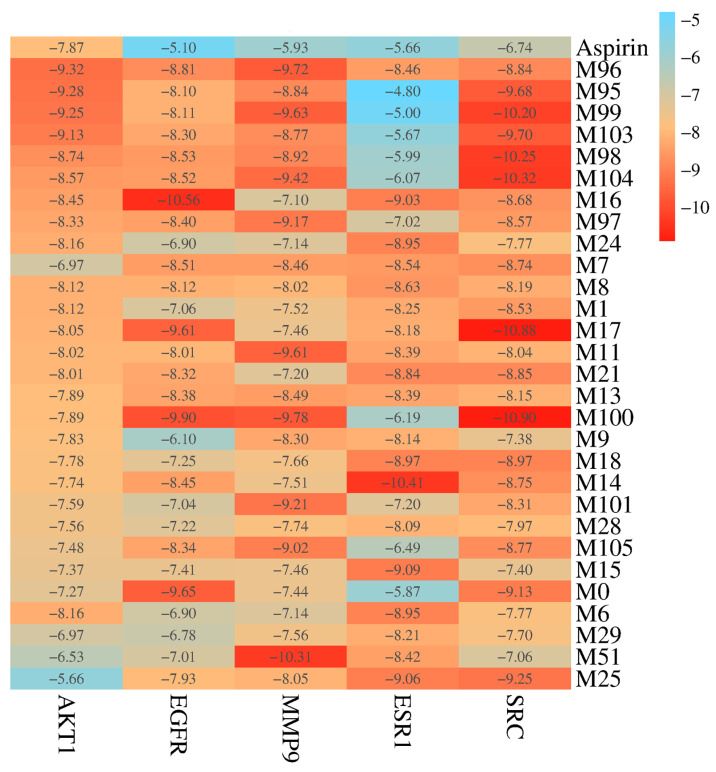
Heat map of molecular docking scoring.

**Figure 11:**
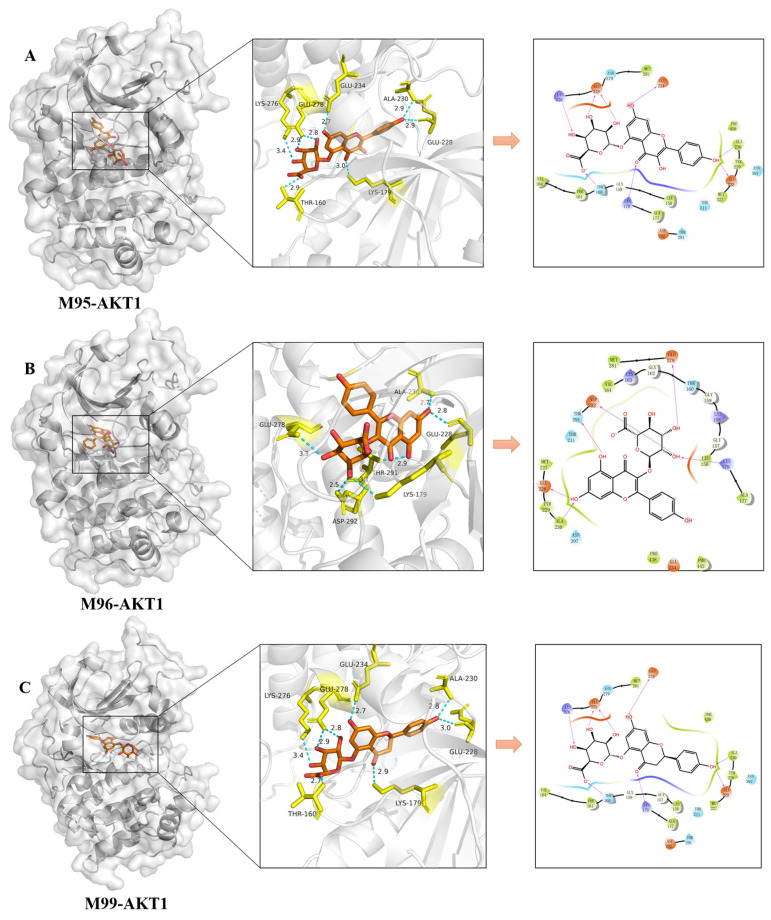
Molecular docking results of M95 (**A**), M96 (**B**), and M99 (**C**) with AKT1. AKT1: serine/threonine kinase 1.

**Figure 12:**
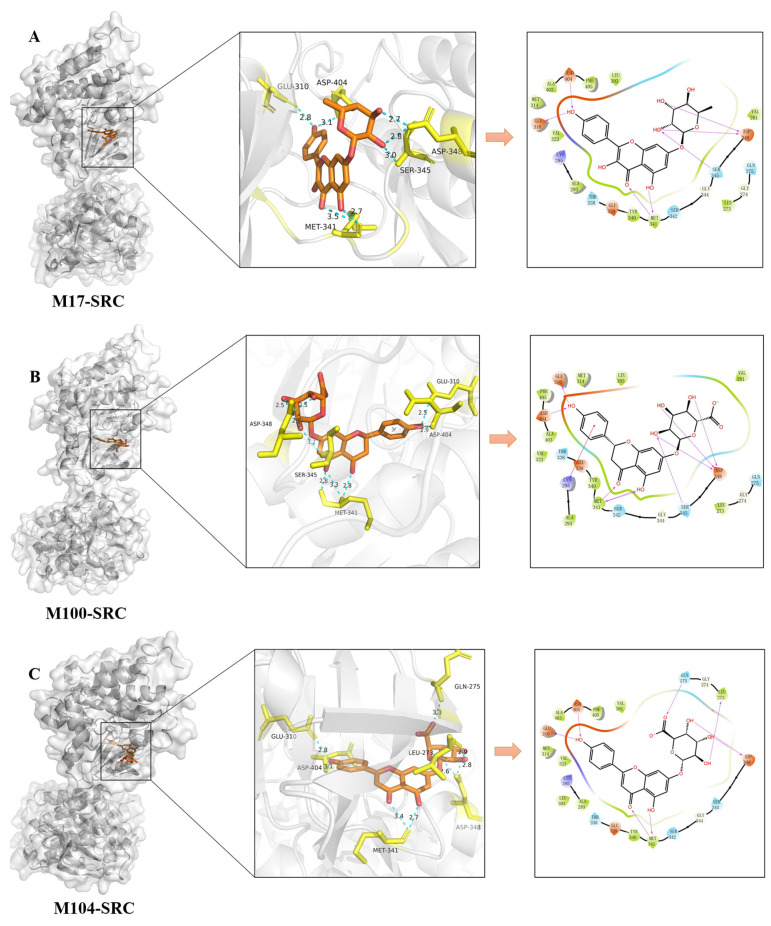
Molecular docking results of M17 (**A**), M100 (**B**), and M104 (**C**) with SRC. SRC: steroid receptor coactivator.

In order to mimic the interactions of compounds and targets, the molecular docking of 28 effective forms with five antithrombotic core targets (AKT1, EGFR, MMP9, ESR1, and SRC) was performed (Figure 10, Figure 11 and Figure 12 and Figures S75–S79 and Table S14). The scores of binding energies of aspirin and effective forms with targets are shown in Figure 10. It is usually believed that the smaller docking binding energy leads to a more stable conformation (the deeper red color with a lower score in the heat map). Aspirin is a clinically recognized antithrombotic positive control, exhibiting moderate binding affinities to the five core targets (AKT1, EGFR, MMP9, ESR1, and SRC) with docking scores of −7.87, −5.10, −5.93, −5.66, and −6.47 Kcal/mol, respectively. The study shows that KAE (M0), phase I metabolites (M1, M6, M7, M8, M9, M11, M13, M14, M15, M16, M17, M18, M21, M24, and M25) and phase II metabolites (M28, M29, M51, M95, M96, M97, M98, M99, M100, M103, M104, and M105) can bind to multiple antithrombotic targets though the binding ability varies. Furthermore, this result was verified using AutoDock Vina (version 1.1.2) software, and the results are shown in Figure S80 and Table S15.

The detailed binding poses and binding sites of effective forms with AKT1 are present in Figure 11A–C. M95 (kaempferol-5-*O*-glucuronide) forms eight hydrogen bonds with the amino acid residues of LYS276, LYS179, GLU228, GLU278, GLU234, ALA230, and THR160 (Figure 11A). M96 (kaempferol-3-*O*-glucuronide) forms six hydrogen bonds with the amino acid residues of LYS179, GLU228, GLU278, ASP292, ALA230, and THR291 (Figure 11B). M99 (naringin-5-*O*-glucuronide) forms eight hydrogen bonds with the amino acid residues of LYS179, LYS276, GLU228, GLU278, GLU234, ALA230, and THR160 (Figure 11C). Therefore, these three effective forms exhibit good binding ability with AKT1 (the molecular docking score was −9.28, −9.32, and −9.25, respectively), indicating higher binding affinity between the compound and the target. Similarly, multiple binding sites are also observed for other effective forms (Figure S75).

Figure 12A–C presents the binding poses and binding sites of effective forms with SRC. M17 (kaempferol-7-*O*-rhamnoside) forms seven hydrogen bonds with the amino acid residues of GLU310, ASH404, MET341, SER345, and ASP348 (Figure 12A). M100 (naringin-7-*O*-glucuronide) forms eight hydrogen bonds with the amino acid residues of GLU310, ASH404, MET341, SER345, and ASP348, and exhibits pi-cation interaction with LYS295 (Figure 12B). M104 (apigenin-7-*O*-glucuronide) forms eight hydrogen bonds with the amino acid residues of GLU310, ASH404, MET341, ASP348, LEU273, and GLN275 (Figure 12C). Therefore, these three effective forms exhibit good binding ability with SRC (the molecular docking scores were −10.88, −10.99, and −10.82, respectively), indicating higher binding affinity between the compound and the target. Similar multiple binding sites are also observed for other effective forms (Figure S76).

SRC plays an important role in mediating the rapid response of platelets to vascular injury [30]. SRC kinase-transmitted signaling can activate platelets via G protein-coupled receptors [31]. Furthermore, SRC kinase activation was also important for platelet spreading, an action of platelets standing for outside-in signaling triggered by the interaction between GPIIb/IIIa and fibrinogen [32]. The downstream effectors of SRC kinase include adaptors, enzymes, and cytoskeletal proteins. Among them, PI3K is the most crucial one, which can completely activate AKT1 by phosphatidylinositol-4, 5-bisphosphate (PIP2). AKT1 was also known as protein kinase B (PKB), which is the most well-known activation marker of PI3K. The PI3K/AKT pathway plays a vital role in the regulation of platelet function, including platelet aggregation and the secretion of platelet granules [33]. Several studies have revealed that PI3K/AKT is an important mediator in *α*IIb/β3 activation, an essential step for platelet aggregation [34,35]. Therefore, we can speculate that the inhibition effect of effective forms on platelet aggregation may be attributed to the suppression of the SRC/PI3K/AKT pathway.

### 2.5. Molecular Dynamic Simulations

To further understand the interaction mechanisms of the effective forms, we conducted molecular dynamic simulations for M96 with AKT1 and M100 with SRC, which exhibited the most favorable molecular docking performance.

**Figure 13:**
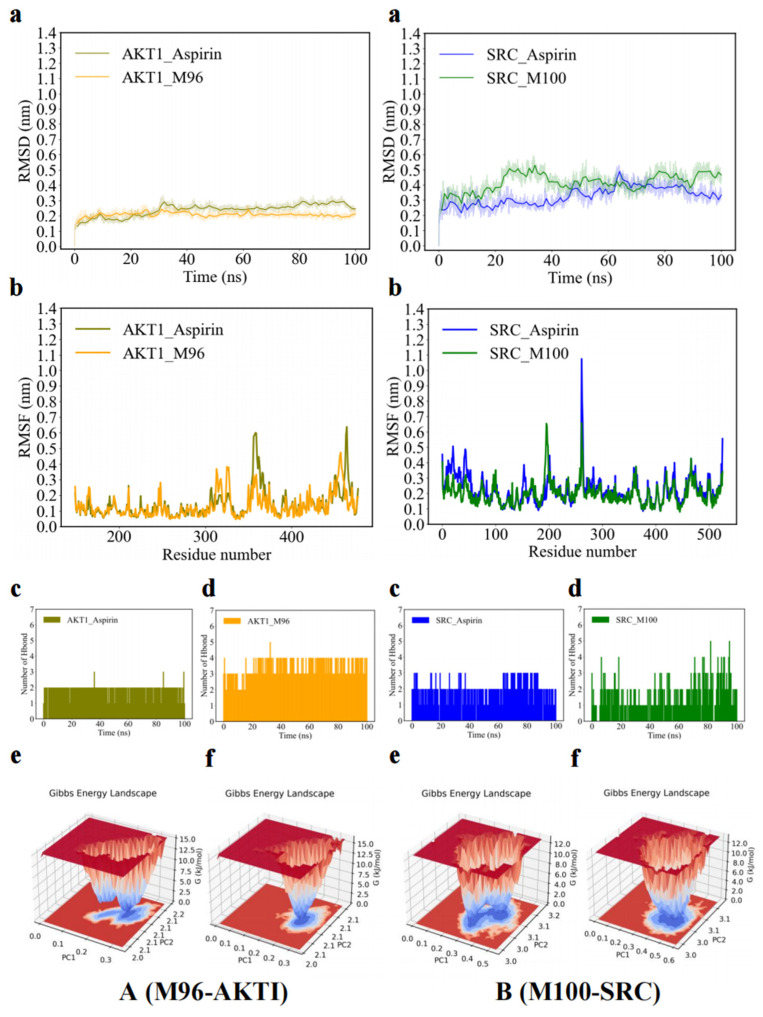
Molecular dynamic simulations of M96-AKT1 (**A**) and M100-SRC (**B**). (**a**: RMSD, **b**: RMSF, **c**,**d**: Number of hydrogen bonding, **e**,**f**: Gibbs energy landscape). AKT1: serine/threonine kinase 1; SRC: steroid receptor coactivator; RMSF: root-mean-square fluctuation.

MSD is commonly used to evaluate the structural changes in proteins [36]. Generally, the RMSD values remained within a narrow range, suggesting that the binding of proteins to small molecules was relatively stable. As shown in Figure 13(Aa), the RMSD value of M96-AKT1 varied slightly since 40 ns, which demonstrates that this complex reached a steady state after 40 ns. The RMSD value of M100-SRC was steady at the time of 20–60 ns, Figure 13(Ba), which suggests this complex reaches a stable state at this period. RMSF analysis is a crucial method for identifying rigid and flexible regions within the protein structure. High fluctuations usually indicate a higher degree of structural flexibility in the region. It can be observed that the residue fragments of 10–20, 42–50, and 75–85 have high fluctuations after M96 binds to AKT1, Figure 13(Ab). The residue fragments of 12–18, and 30–40 showed high fluctuations after M100 binds to SRC, Figure 13(Bb). In addition, the covariance matrix color and the positive correlation movement for the residue fragments of 48–53 and 68–75 in M96-AKT1, Figure 13(Ac), and 12–18 and 30–40 in M100-SRC, Figure 13(Bc) are significantly increased, which indicates the movement between these regions is more synchronized and may have higher structural stability.

The formation or distortion of hydrogen bonds plays a pivotal role in molecular dynamic simulation, directly influencing the binding affinity. As shown in Figure 13(Ad), there are a large number of hydrogen bonds, with a maximum of five, during the entire molecular dynamics simulation of M96-AKT1. The binding free energy of M96-AKT1 is −15.48 Kcal/mol, Figure 13(Ae), which demonstrates M96 has a good affinity to the AKT1. Similarly, there are a large number of hydrogen bonds, with a maximum of five, during the entire molecular dynamics simulation of M100-SRC, Figure 13(Bd). M100 also has a good affinity with SRC, and the binding free energy is −30.87 Kcal/mol, Figure 13(Be). These results suggest the establishment of hydrogen bonds between M96 and AKT1, and M100 and SRC, consistent with the outcomes of molecular docking. Therefore, M96 and M100 could enter the AKT1 and SRC receptor protein, to form a stable complex through intermolecular interaction forces. Together, the result strongly supports the validity of the docking results.

## 3. Discussion

Thrombosis is a serious CVD that significantly impacts human health and life. In recent years, the incidence of thrombosis has been rising, leading to increased morbidity and mortality risks [37]. Currently, there is no specific medication available for treating thrombosis, making it crucial to discover drugs that can effectively manage the condition with fewer side effects. This study identified KAE, a natural flavonol dirhamnopyranoside extracted from the active fraction of *C. orbiculatus*, which demonstrated a notable antithrombotic effect with a reduced risk of bleeding. In the rabbit plasma recalcification test, PRT was significantly prolonged in the KAE-treated group, similar to the effects of aspirin. Additionally, in a rat model of arterial thrombosis induced by FeCl_3_, both the length and wet weight of the thrombus were significantly decreased with KAE treatment, comparable to aspirin, while the tail vein bleeding time was shorter than that observed with aspirin.

The formation of thrombosis is a complex process that is closely linked to coagulation function, platelet and vascular activity, and the performance of the fibrinolytic system [38]. Four key indicators of coagulation function are APTT, PT, TT, and FIB. APTT primarily reflects the levels of endogenous coagulation factors, while PT indicates the levels of exogenous factors such as prothrombin and factors V, VII, and X in the bloodstream. TT measures the duration it takes for fibrinogen to convert into fibrin, with a prolonged TT suggesting an increase in anticoagulant substances like heparin [39]. FIB, also known as coagulation factor I, is the main protein involved in the coagulation process, and elevated levels of FIB suggest that the common coagulation pathway is being expedited [40]. The experimental results demonstrated that KAE significantly prolonged APTT, PT, and TT, while simultaneously reducing FIB compared to the control group (with APTT and TT in the KAE-H group showing *p* < 0.01; PT in the KAE-H group showing *p* < 0.05; and FIB in the KAE-H group showing *p* < 0.01). Thus, KAE may exert an anticoagulant effect by influencing the activation of relevant coagulation factors and fibrinolysis in both endogenous and exogenous coagulation pathways.

The formation of thrombosis is linked to the activation and clumping of platelets. Factors that inhibit platelets help prevent their adhesion to one another and endothelial tissue [41]. When activated, platelets can attach to fibrinogen and collagen, leading to aggregation [42]. TXA_2_ and PGI2 are two opposing substances that play a crucial role in regulating platelet activity. Maintaining a balance between TXA_2_ and PGI2 is essential for the integrity of blood vessel walls and proper regional blood flow. An imbalance can lead to conditions such as vasospasm and thrombosis, which may result in myocardial infarction, angina, and strokes [43]. The levels of TXA_2_ and PGI2 can be directly indicated by the levels of TXB_2_ and 6-keto-PGF1*α*. A higher ratio of 6-keto-PGF1*α* to TXB_2_ suggests stronger antithrombotic effects of a drug [44]. In this study, we discovered that KAE significantly reduced plasma TXB_2_ levels while increasing plasma 6-keto-PGF1*α* levels, thus enhancing the 6-keto-PGF1*α*/TXB_2_ ratio. This finding suggests that KAE has anti-thrombotic properties as a platelet inhibitor.

t-PA and PAI-1 are key markers of the fibrinolytic system, maintaining a dynamic balance. t-PA facilitates the conversion of plasminogen into fibrinolysin, which plays a crucial role in the intrinsic fibrinolytic system by breaking down insoluble fibrin into soluble products, thereby dissolving clots. PAI-1 serves as an indicator of the fibrinolytic system’s activity. Consequently, higher levels of t-PA and lower levels of PAI-1 indicate a stronger antithrombotic effect of a drug. Both t-PA and PAI-1 regulate fibrinolytic activity, ensuring proper blood flow and vessel openness. If the balance between t-PA and PAI-1 is disrupted, the breakdown of local fibrin in blood vessels decreases, leading to fibrin accumulation and potential thrombosis [45]. This study shows that KAE can significantly boost t-PA levels while reducing PAI-1 levels in plasma, suggesting that KAE has an antithrombotic effect and may enhance the fibrinolytic system’s activity.

In the study of the antithrombotic “effective forms” of KAE, a detailed metabolic profile was conducted using UHPLC-Q Exactive-Orbitrap-MS technology. This analysis identified a total of 192 metabolites after KAE was administered to rats. The identification process indicated that various complex phase I and phase II metabolic reactions took place in vivo, including deglycosylation, hydrogenation, hydroxylation, dehydroxylation, methylation, demethylation, as well as related sulfation and glucuronidation, with some reactions occurring up to two times. Calderon-Montano et al. noted that after kaempferol absorption, metabolites such as glucuronidated, methylated, and sulfated forms were produced, with kaempferol-3-glucuronic acid being the primary metabolite found in blood and urine, alongside mono- and di-sulfated metabolites [46]. Twenty-eight compounds were identified as “effective forms” according to network pharmacology, which included KAE, 15 phase I metabolites, 2 methylated metabolites, 1 sulfated metabolite, and 9 glucuronidated metabolites. Additionally, metabolites showing disulfide formation, diglucuronide structures, glucose binding characteristics, and C-ring cleavage were also found, suggesting they may represent effective forms of KAE. It is proposed that both phase I and phase II metabolites play a significant role in the in vivo efficacy of KAE.

To investigate the antithrombotic mechanism, KAE and its “effective forms” were analyzed through network pharmacology, molecular docking, and molecular dynamics simulations. KAE and 27 metabolites primarily interacted with 28 core targets, with mechanisms largely linked to the PI3K-AKT, VEGF, thyroid hormone, HIF-1, and MARK signaling pathways. This aligns with earlier findings that kaempferol’s anti-atherosclerotic effects are associated with genes such as TNF-*α*, HIF-1*α*, and the PI3K-Akt signaling pathways. Choi JH et al. noted that kaempferol inhibits collagen and thrombin stimulation, reducing the phosphorylation of PI3K, Akt, ERK, and p38 induced by thrombin, thereby decreasing thrombus formation in mice [47]. PI3K can fully activate AKT through phosphatidylinositol-4, 5-bisphosphate (PIP2), which allows AKT to release NF-*κ*B in the cytoplasm. The PI3K/AKT pathway is a well-known antithrombotic signaling pathway crucial for regulating platelet functions, including aggregation and granule secretion. Multiple studies have shown that PI3K/AKT is a significant mediator in *α*IIb/β3 activation, a critical step in platelet aggregation [48,49,50]. Thus, it can be inferred that KAE’s inhibitory effect on platelet aggregation may stem from the suppression of the SRC/PI3K/AKT pathway. Molecular docking results confirmed that the core targets exhibit strong binding affinity with the active compounds, and molecular dynamics simulations indicated that the **M96**-AKT1 and **M100**-SRC complexes are quite stable. However, despite these important preliminary findings, there are limitations. The antithrombotic “effective forms” of KAE should be prepared using various chromatographic separation techniques based on UHPLC-Q-Exactive Orbitrap MS technology for identification. The antithrombotic mechanism was explored solely through advanced bioinformatics and computational methods, so further validation through cell and animal studies is necessary to ensure the reliability and accuracy of the predictions.

## 4. Materials and Methods

### 4.1. Chemicals and Reagents

KAE, kaempferol, kaempferol-3-*O*-rhamnoside, and kaempferol-7-*O*-rhamnoside were isolated from the fruit of *C. orbiculatus* in our laboratory, and their purities are more than 98%, as determined by high-performance liquid chromatography (HPLC) analysis. Apigenin (batch no. CFS202402) was purchased from Wuhan Zhongbiao Technology Co., Ltd. (Wuhan, China), and its purity was 98.3%. Aspirin (batch no. 23041203) enteric-coated tablets were purchased from Hui-Kang Pharmacy (Tangshan, China). activated partial thromboplastin time (APTT) (batch no. B4218-2), thrombin time (TT) (batch no. OWHM13), prothrombin time (PT) (batch no. OUHP49), and fibrinogen (FIB) (batch no. B4233-15) assay kits were purchased from Siemens Healthcare Diagnostics Products GmbH (Shanghai, China). Thromboxane B2 (TXB_2_) (batch no. YM-S2052), 6-keto-prostaglandin F1*α* (6-keto-PGF1*α*) (batch no.YM-S1804), tissue-type plasminogen activator (t-PA) (batch no. YM-S2059), and plasminogen activator inhibitor (PAI-1) (batch no. YM-S1608) assay kits were purchased from Yuanmu Biological (Shanghai, China). Normal saline (batch no. C1230911A1) was purchased from Beyotime Biotech Inc. (Shanghai, China). Acetonitrile (batch no. P2937380) and formic acid (batch no. C14569049) were purchased from Titan Technology Co., Ltd. (Shanghai, China) and are chromatographic grade. Sodium citrate (batch no. 20231013), ethyl carbamate (batch no. EK231220), dimethyl sulfoxide (DMSO) (batch no. 01912114), sodium carboxymethyl cellulose (CMC-Na) (batch no. 20240101), calcium chloride (CaCl_2_) (batch no. 20231226), and ferric chloride (FeCl_3_) (batch no. 20240113) were purchased from Tianjin Jindong Tianzheng Fine Chemical Reagent Factory (Tianjin, China) and analytic grade.

### 4.2. Experimental Animals

Rabbits (body weight: 1.5–3.0 kg) and male Sprague-Dawley rats (body weight: 250–300 g) were acquired from the Experimental Animal Center of North China University of Science and Technology (Tangshan, China). All animals were maintained under controlled environmental conditions (constant temperature of 22 ± 2 °C with a relative humidity of 50 ± 5%). All experimental procedures were performed in accordance with the Biomedical Ethical Committee of North China University of Science and Technology (Approval No. 2024SY3046).

### 4.3. Antithrombotic Activity of KAE In Vitro

The plasma recalcification time (PRT) of KAE was measured following a previously established protocol [51]. Fresh rabbit blood was obtained from the ear vein and combined with sodium citrate (38 mg/mL) in a volume ratio of 9:1, then centrifuged for 10 min at 1000 rpm. The supernatant was collected as the platelet-rich plasma (PRP) and incubated at 37 °C prior to use. KAE was dissolved in 5% DMSO to obtain a KAE solution with a concentration of 20 mg/mL. In low dose of KAE (KAE-L) and high dose of KAE (KAE-H) groups, 0.05 and 0.1 mL of KAE solution was added to 0.1 mL of PRP and incubated for 1 min at 37 °C, respectively, and then 0.1 mL of CaCl_2_ solution (2.8 g/L) was added. The final concentrations of KAE-L and KAE-H were 3330 and 6670 µg/mL. The PRT was noted as the duration until a clot formed. In the control and positive groups, normal saline and aspirin (6670 µg/mL) were used instead of KAE.

### 4.4. Platelet Aggregation Assay In Vitro

Platelet aggregation was determined using the CHRONO-LOG^®^ Model 700 Whole Blood/Optical Lumi-Aggregometers (Chrono-Log Corporation, Havertown, PA, USA). Rabbit blood was collected from the abdominal aorta and mixed with sodium citrate (38 mg/mL) at a ratio of 9:1, then the mixture was centrifuged at 1000 and 3000 rpm/min to obtain platelet-rich plasma (PRP) and platelet-poor plasma (PPP). The platelet density of the obtained PRP was adjusted to 2 × 10^8^/mL by adding PPP. The 20 μL of saline, aspirin (54.05 µg/mL), KAE-L (54.05 µg/mL), and KAE-H (108.10 µg/mL) were added to 274 μL of PRP, respectively, and the mixed PRP was incubated for 20 min. The baseline was automatically set and adjusted, and the mixed PRP was continuously incubated for 10 min. The 6 μL of ADP working solution was added to start the assay, and the aggregation rate was detected.

### 4.5. Antithrombotic Activity of KAE In Vivo

#### 4.5.1. Rats Administration, Thrombus Formation, and Plasma Collection

Rat’s thrombosis was induced by FeCl_3_ according to a previously established method [52]. KAE-L (30 mg/kg), KAE-H (60 mg/kg), normal saline (0.1 mL/kg), and aspirin (30 mg/kg) were orally administered to rats for seven days prior to the FeCl_3_ injury. On the seventh day, rats were anesthetized via intraperitoneal injection of 20% ethyl carbamate (0.8 mL/kg) two hours after the last oral administration. An incision was made from the mandible to the suprasternal notch to expose a segment of the right carotid artery. Thrombus formation was initiated by placing pieces of filter paper (1.5 × 2 cm) around the vessel for 10 min, and the filter paper was soaked in 10 μL of FeCl_3_ solution at a concentration of 2.2 mol/L. The right arterial thrombus was carefully dissected and fixed in 10% formaldehyde overnight at 4 °C before being stained with hematoxylin and eosin (H&E) for histopathological examination. Blood samples were collected from the left abdominal aorta and transferred into tubes containing sodium citrate (38 mg/mL, the volume ratio of citrate to blood was 1:9), then centrifuged at 3000 rpm for 15 min to obtain the plasma.

#### 4.5.2. Evaluation of Antithrombotic Activity

The plasma was obtained as described in the above method. The coagulation parameters of APTT, PT, TT, and FIB were determined using an automatic coagulation analyzer (CA-1500, Instrumentation Laboratory, Kirchheim, Germany). The contents of TXB_2_ and 6-keto-PGF1*α* were determined with an enzyme-linked immunosorbent assay (ELISA) to evaluate platelet aggregation function. The contents of t-PA and PAI-1 were determined with ELISA to evaluate fibrinolysis function.

The tail vein bleeding time was measured to assess hemostasis in vivo. On the seventh day, the rats were anesthetized two hours following the final oral dose. The tail was cut 5 mm from the tips and immersed in normal saline at 37 °C immediately to measure the bleeding time. The bleeding time was defined as the duration until hemorrhage ceased, and it exceeded 15 min to be recorded as 15 min.

### 4.6. Study on the “Effective Forms” of KAE for Antithrombotic Activity

#### 4.6.1. Metabolism Experiment of KAE

Six rats were randomly divided into the KAE and blank groups, and each group contained three rats. The rats in each group were individually housed in metabolic cages (Suzhou Fengshi Laboratory Animal Equipment Co., Suzhou, Jiangsu province, China). The rats were kept for 5 days to acclimate to the facilities. The KAE group rats were orally administered to KAE solution (KAE was suspended in 0.1% CMC-Na solution at a concentration of 10 mg/mL) with a dose of 50 mg/kg. The blank group rats were orally administered to 0.1% CMC-Na solution with the same volume as that of the KAE group. The drug administration lasted for 7 days at a frequency of once a day (at 8:30 a.m.).

#### 4.6.2. Biosamples Collection and Preparation

Urine sample: During the period of drug administration, urine samples from the rats in the KAE and blank groups were collected separately every day. All the urine collection tubes were completely cleaned and then filled with 1 mL of absolute ethanol as a preservative before the collection was performed. Urine samples were collected at three time points (8:00 a.m., 2:00 p.m., and 10:00 p.m.) each day during the period of drug administration. The collected urine samples from the same group of rats were merged and evaporated to dry under reduced pressure at 45 °C using a RE52CS rotary evaporator (Shanghai Yarong Biochemical Instrument Factory, Shanghai, China). The urine samples that cannot be treated immediately were temporarily stored at −20 °C in a fridge. The dried urine samples were supplemented with 10-fold (*v*/*w*) methanol to precipitate the endogenous substances, and the supernatant was condensed to obtain the urine residue. As a result, a total of 12 g of the urine residue (yellow viscous solid) was obtained from each group. A total of 1 g of the urine residue for each group was supplemented with 10-fold methanol (*v*/*w*) and ultrasonically extracted for 30 min. The extract was centrifuged at 5000 rpm for 20 min, and the supernatant was dried in a vacuum at 45 °C and resolved with 2 mL of methanol. Finally, the prepared urine samples were filtered through a 0.22 μm membrane for UHPLC-MS analysis.

Feces sample: The feces samples of the two groups were collected separately at 8:30 a.m. each day, starting on the second day, also for 7 days. The feces samples were transferred from the feces collection tubes into two prepared cartons and immediately dried at 45 °C using an electro-thermostatic blast oven. A total of 110 g of dried feces samples were obtained for each group. 1 g of powdered feces samples for each group was mixed with 10 mL of methanol and then ultrasonically extracted for 30 min. The extract was centrifuged at 5000 rpm for 20 min, and the supernatant was dried in a vacuum at 45 °C to obtain the feces residues. Subsequently, the feces residues for the two groups were dissolved in 2 mL of methanol. Finally, the prepared feces samples were filtered through a 0.22 μm membrane for UHPLC-MS analysis.

Plasma sample: On the seventh day, the rats were anesthetized after 0.5 h at the last administration. Then, the blood samples were collected from the heart into heparinized tubes using the puncture method. The collected blood samples from rats within the same group were mixed and immediately centrifuged at 5000 rpm for 20 min to obtain the plasma samples. A total of 17 mL of the plasma was supplemented with a 10-fold volume of methanol and ultrasonically extracted for 30 min to precipitate the protein. The extract was then centrifuged at 10,000 rpm for 10 min, and the supernatant was dried in a vacuum at 45 °C. Subsequently, the dried plasma samples were dissolved in 2 mL of methanol. Finally, the prepared plasma samples were filtered through a 0.22 μm membrane for UHPLC-MS analysis.

#### 4.6.3. Instruments and Conditions

The experiments were performed using a Thermo Fisher Scientific UHPLC system (Dionex UltiMate 3000, Thermo Fisher Scientific, Waltham, MA, USA) coupled with a high-resolution Q Exactive orbitrap mass spectrometer (Thermo Fisher Scientific, Waltham, USA). Biosamples were separated on a Waters Atlantis T3 (2.1 × 100 mm, 1.8 μm) (Waters Corporation, Milford, CT, USA). The mobile phase consisted of 0.1% formic acid (A) and acetonitrile (B). The gradient elution program was set as follows: 0.01–1 min, 5% B; 1–10 min, 5–40% B; 10–13 min, 40–95% B; 13–17 min, 95–5% B. The injection volume was 5 μL; the flow rate was 0.3 mL/min, and the column temperature was maintained at 35 °C. The mass spectra analysis conditions: negative ion and positive ion detection modes were simultaneously applied; the full MS scan range was *m*/*z* 250–1200 Da, and the resolution was 70,000. The sheath gas flow rate is 45 arbitrary units, the aux gas flow rate is 10 arbitrary units, the spray voltage is 3.5 kV, the capillary temperature is 275 °C, and the aux gas heater temperature is 450 °C.

#### 4.6.4. Metabolites Characterization

The metabolites of KAE in rats were thoroughly screened based on the strategy proposed in our previous study. The structural elucidation of the metabolites was conducted by careful interpretation of their UHPLC-MS data. The first step was to confirm the types of metabolic reactions according to the characteristic mass differences in MS data. For example, −15.023 Da (−CH_3_), +2.016 Da (+2H), +15.023 Da (+CH_3_), +15.995 Da (+OH), +79.957 Da (+SO_3_), and +176.031 Da (+C_6_H_8_O_6_) are generally considered to represent the demethylation, hydrogenation, methylation, hydroxylation, sulfation, and glucuronidation metabolic reactions, respectively. The second step was to identify the skeletal structures of the metabolites by comparing the MS^2^ data with that of KAE and its aglycone kaempferol. The final step was to determine the exact conjugated sites based on the diagnostic fragment ions that arose from the Retro-Diels–Alder (RDA) cleavage of flavonoids. In addition, the calculated n-octanol/water partition coefficient (CLog *P*) value was also applied to discriminate the metabolites.

To clearly describe these metabolites, the confidence levels (CL) for all metabolites were established. Confidence level 1 (CL 1): metabolites were clearly identified by comparison with the reference compounds, and retention time (t*_R_*) was aligned. Confidence level 2 (CL 2): metabolites were tentatively identified by interpretation of their MS^n^ data. For these metabolites, the metabolic reaction sites can be speculated by the key diagnostic fragments. Confidence level 3 (CL 3): metabolites were tentatively identified by interpretation of their MS^n^ data. For these metabolites, the type of metabolic reaction can be determined, but the metabolic reaction sites can not be speculated due to the absence of the key diagnostic fragments. Therefore, the new metabolites with CL 1, 2 are presented in the main text, and the other metabolites with CL 3 were placed in the Supplementary Materials.

### 4.7. Network Pharmacology

#### 4.7.1. Targets Collection for KAE Metabolites

The structures of the obtained metabolites of KAE were researched using the PubChem (https://www.ncbi.nlm.nih.gov/pubmed), (accessed on 29 September 2024) [53] platform and saved in standard SMILES format (Canonical SMILES). The potential targets of selected metabolites were obtained by importing those metabolites into the Swiss Target Prediction (http://www.swisstargetprediction.ch/), (accessed on 29 September 2024) [54] and SEA Search Server (https://sea16.docking.org/), (accessed on 29 September 2024) [55] database online analysis platform. Duplicate drug targets were removed and converted to the standard gene name using the Uniprot database (https://www.uniprot.org/), (accessed on 29 September 2024) [56] with a limit on the species of “human”.

#### 4.7.2. Antithrombotic Targets Collection for KAE Metabolites

The keywords “Thrombus” and “Thrombosis” were used as medical subject headings (MeSH) for thrombosis in GenCLiP 3 (http://cismu.net/genclip3/analysis.php), (accessed on 30 September 2024) [57] to screen the disease targets of thrombus. Antithrombotic targets of metabolites were obtained by cross-analysis of metabolite targets (Section 4.6.1) and thrombosis disease targets.

#### 4.7.3. Key Antithrombotic Targets Analysis for KAE Metabolites

The obtained antithrombotic targets of metabolites (Section 4.6.2) were imported into the STRING database (https://string-db.org/), (accessed on 30 September 2024) [58], and the interaction network of antithrombotic targets was constructed by selecting the human-derived interaction protein as the background with medium confidence. Then the interaction data were imported into Cytoscape 3.10.0 (https://www.cytoscape.org/), (accessed on 30 September 2024) [59] to construct the protein–protein interaction (PPI) network. The core targets of metabolites for antithrombotic activity were obtained using the key node analysis tool cytoHubba [60] based on the target degree value in the interaction network.

#### 4.7.4. GO and KEGG Pathway Enrichment Analysis

The biological processes and metabolic pathways of antithrombotic targets were analyzed using Gene Ontology (GO) and Kyoto Encyclopedia of Genes and Genomes (KEGG) pathway enrichment analysis based on DAVID (https://davidbioinformatics.nih.gov, Version 6.8), (accessed on 30 September 2024) [59] database to elucidate antithrombotic mechanisms of metabolites of KAE. The gene names of antithrombotic targets were input into the DAVID database to enrich the biological process and pathway.

### 4.8. Molecular Docking

#### 4.8.1. Pretreatment of Receptor Proteins

The crystal structure of the target protein was downloaded from the protein data bank (PDB) database (https://www.rcsb.org/), (accessed on 3 October 2024) and subsequently processed using the protein preparation wizard in Schrodinger Suite 2021. Unnecessary components, such as redundant water molecules and small ligand molecules, were removed [61]. Missing amino acid side chains and flexible ring regions were repaired according to a pH range of 7.0 ± 0.5. The optimized potentials for liquid simulations 4 (OPLS4) force field were employed to minimize the energy, with root mean square deviation (RMSD) convergence threshold set at 0.3 Å. Only hydrogen atom conformations and side chains were optimized to maintain the stability of the protein backbone. The processed structure was saved in Maestro format (.MAE) for subsequent docking.

#### 4.8.2. Pretreatment of Ligand Small Molecules

The ligand small molecule structures were standardized using the LigPrep module, which automatically generated tautomers, ionized states, and stereoisomers. The enhanced torsional kinetic distance geometry (ETKDG) algorithm was utilized for conformation searching, retaining only the 30 lowest energy conformations as initial candidates. Based on the OPLS4 force field, ligand structures underwent optimization, and a multi-conformational ligand library was obtained.

#### 4.8.3. Generation of Docking Grid Interface

The docking active pocket was defined utilizing the receptor grid generation module. The centering on the primary ligands within the crystal structure, a grid box size measuring 20 × 20 × 20 Å (along x, y, z axes) was established to ensure comprehensive coverage of both the entire active pocket and surrounding flexible areas; redundant protein regions outside this active pocket were eliminated while retaining only those residues likely to interact with ligands for generating a docking mesh file.

#### 4.8.4. Molecular Docking Parameter Setting

The glide module was employed for docking calculations. The “Standard Precision Mode (SP)” was utilized, with the number of docking conformations set to 50, retaining the top 5 conformations for each ligand. All other parameters were maintained at their default settings. The binding energy was computed using the OPLS4 force field, which accounts for van der Waals interactions, electrostatic interactions, and solvation effects between ligands and proteins. The docking results were ranked according to GlideScore (Kcal/mol).

After molecular docking, the total score of the active ingredient and the key target was obtained. A score smaller than −5.0 indicates good binding activity, while a docking score smaller than −7.0 indicates high binding activity.

### 4.9. Molecular Dynamics Simulation

Based on the molecular docking results, the molecules were protonated at pH 7.4 using Open Babel and subjected to energy minimization with the Merck molecular force field 1994 (MMFF94) force field. The minimization was performed for a max of 500 steps, with a convergence criterion of an energy change below 0.0001 Kcal/mol, to obtain energetically stable molecular conformations. The protein receptor structures were prepared using SPDBV 4.10 to repair missing residues and loops. The ligand topology was generated using the AnteChamber PYthon Parser interface (ACPYPE) web server for subsequent classical molecular dynamics simulations.

Classical molecular dynamics (MD) simulations were performed using GROMACS software (version 2023-plumed_2.9.1) to confirm the molecular docking results [62,63]. The PDB files of proteins were processed to construct the relevant topology structure. In addition, the topology structure of the ligand was processed using the ACPYPE web server (https://www.bio2byte.be/acpype/submit/), (accessed on 16 October 2024). The AMBER99SB-ILDN force field and three-point transferable intermolecular potential (TIP3P) water model were utilized for calculations, and the system was confined within a cubic box with dimensions of 1.0, with SPC216 aqueous solvent and Na^+^/Cl^−^ ions added to neutralize charges and prevent protein-ligand collisions. Energy minimization of the protein-ligand complexes was conducted at 300 K for 1000 steps. Subsequently, the system underwent NVT equilibration at 300 K, followed by NPT equilibration using the Parrinello-Rahman barostat method at 1 bar for 100 ps, with a time step of 2 fs. The coordinates and energies of the systems were recorded every 10 ps. Each system was then simulated for 100 ns, during which the molecular trajectories were corrected for subsequent evaluation and analysis. Long-range electrostatic interactions were calculated using the LINCS method to constrain hydrogen bonds, while short-range electrostatic and van der Waals interactions were calculated using the PME method. For binding free energy calculations (MM-PBSA), the binding free energy of the system was determined using the Molecular Mechanics/Poisson-Boltzmann (Generalized Born) method, specifically through the gmx_MMPBSA program [64]. The calculation Formula (1) is shown below.

ΔGbind = ΔGcomplex − (ΔGreceptor + ΔGligand) (1)

where ΔGbind is the total binding energy, ΔGcomplex is the binding energy of the free complex, ΔGreceptor is the binding energy of the free receptor, and ΔGligand is the binding free energy of the unbound ligand.

### 4.10. Statistical Analysis

The data was expressed as the mean ± standard deviation ($\bar{x}$ ± SD). Normality and homogeneity of data were verified by Shapiro–Wilk and Brown–Forsythe tests. Statistical significance was assessed by the SPSS package (version 27.0, SPSS Inc., New York, NY, USA) and was performed by one-way analysis of variance (ANOVA) Tukey honestly significant difference (Tukey HSD), and least significant difference (LSD) test for multiple group comparisons, and p < 0.05 was considered statistically significant.

## 5. Conclusions

Kaempferitrin (KAE) is the primary active component in the extract of *C. orbiculatus* that exhibits antithrombotic properties. It demonstrates significant antithrombotic effects, such as extended PRT in vitro and a decrease in both the length and wet weight of the thrombus in vivo, without causing bleeding. KAE’s antithrombotic activity is attributed to its influence on both endogenous and exogenous coagulation pathways, its ability to inhibit platelet aggregation, and its enhancement of fibrinolytic system activity. The “effective forms” of KAE include both the compound itself and its partial metabolites, which may interact with the SRC/PI3K/AKT pathway to exert their antithrombotic effects. This research lays the groundwork for a better understanding of the “effective forms” and the mechanisms behind KAE’s antithrombotic activity. Future studies will focus on isolating the antithrombotic “effective forms” of KAE from biological samples or through chemical synthesis and will investigate the mechanisms involved through cell and animal experiments.

## Figures and Tables

**Table 1:** Inhibitory effect of KAE on platelet aggregation induced by ADP.

Group	Dose (µg/mL)	Platelet Aggregation Rate (%)	Platelet Aggregation Inhibition Rate (%)
Control	—	56.53 ± 1.30	—
Aspirin	54.05	14.33 ± 0.53 **	74.66 ± 1.30
KAE-L	54.05	21.01 ± 0.75 **^##^	62.82 ± 1.44 ^##^
KAE-H	108.10	15.34 ± 0.42 **	72.87 ± 0.25

Note: Values are presented as mean ± SD (*n* = 3). One-way ANOVA followed by an LSD test was used to investigate significant differences between the groups. ** *p* < 0.01 vs. Control group, ## *p* < 0.01 vs. aspirin group. —: 20 µL of normal saline; ADP: adenosine diphosphate; KAE-L: low-dose of kaempferitrin; KAE-H: high-dose of kaempferitrin.

**Table 2:** Detailed information of kaempferitrin (KAE) and its new metabolites.

No.	Name	MF	Meas. (Da)	Pred. (Da)	Diff (ppm)	DBE	t*_R_* (min)	P	U	F	CL
M0	Kaempferitrin (KAE)	C_27_H_30_O_14_	577.15509	577.15628	−2.06	13	7.946	▲	▲	▲	1
M1	Kaempferol	C_15_H_10_O_6_	285.04010	285.04046	−1.26	11	12.078		▲	▲	1
M11	Apigenin	C_15_H_10_O_5_	269.04568	269.04555	0.48	11	11.927	▲	▲	▲	1
M16	Kaempferol-3-*O*-rhamnoside	C_21_H_20_O_10_	431.09613	431.09837	−5.20	12	9.370			▲	1
M17	Kaempferol-7-*O*-rhamnoside	C_21_H_20_O_10_	431.09766	431.09837	−1.65	12	10.406			▲	1
* M50	4, 5, 4′-Trihydroxylated dihydrogenated flavone-4′-*O*-sulfate	C_15_H_14_O_7_S	337.03958	337.03875	2.46	9	7.829		▲		2
* M51	4, 5, 4′-Trihydroxylated dihydrogenated flavone-5-*O*-sulfate	C_15_H_14_O_7_S	337.03915	337.03875	1.19	9	8.062			▲	2
* M53	4, 5, 4′-Trihydroxylated dihydrogenated flavone-4-*O*-sulfate isomer 1	C_15_H_14_O_7_S	337.03903	337.03875	0.83	9	9.312		▲		2
* M54	4, 5, 4′-Trihydroxylated dihydrogenated flavone-4-*O*-sulfate isomer 2	C_15_H_14_O_7_S	337.03928	337.03875	1.57	9	9.405	▲			2
* M56	4, 5, 4′-Trihydroxylated dihydrogenated flavone sulfate	C_15_H_14_O_7_S	337.03928	337.03875	1.57	9	10.945	▲			2
* M67	Dihydroxylated flavane sulfate 1	C_15_H_14_O_6_S	321.04330	321.04383	−1.65	9	9.125	▲			3
* M68	Dihydroxylated flavane sulfate 2	C_15_H_14_O_6_S	321.04337	321.04383	−1.43	9	9.408		▲		3
* M69	Dihydroxylated flavane sulfate 3	C_15_H_14_O_6_S	321.04345	321.04383	−1.18	9	9.577	▲		▲	3
* M72	A-Ring methylated dihydrogenated kaempferol sulfate	C_16_H_16_O_9_S	383.04306	383.04423	−3.05	9	9.430		▲		2
* M73	B-Ring methylated dihydrogenated apigenin B-ring sulfate	C_16_H_16_O_8_S	367.04846	367.04931	−2.32	9	7.782		▲		2
* M74	A-Ring methylated dihydrogenated apigenin B-ring sulfate 1	C_16_H_16_O_8_S	367.04810	367.04931	−3.30	9	9.518		▲		2
* M75	A-Ring methylated dihydrogenated apigenin B-ring sulfate 2	C_16_H_16_O_8_S	367.04867	367.04931	−1.74	9	9.616		▲		2
* M76	A, C-Rings of apigenin cracking sulfate 1	C_9_H_8_O_7_S	258.99152	258.99180	−1.08	6	4.063		▲		2
* M77	A, C-Rings of apigenin cracking sulfate 2	C_9_H_8_O_7_S	258.99158	258.99180	−0.85	6	4.533		▲		2
* M78	A, C-Rings of apigenin cracking sulfate 3	C_9_H_8_O_7_S	258.99158	258.99180	−0.85	6	4.570		▲		2
* M79	A, C-Rings of apigenin cracking sulfate 4	C_9_H_8_O_7_S	258.99155	258.99180	−0.97	6	4.760		▲		2
* M82	4, 5, 4′-Trihydroxylated dihydrogenated flavone disulfate	C_15_H_14_O_10_S_2_	416.99478	416.99556	−1.87	9	6.963		▲		2
* M83	4, 7, 4′-Trihydroxylated dihydrogenated flavone disulfate 1	C_15_H_14_O_10_S_2_	416.99460	416.99556	−2.30	9	7.378		▲		2
* M84	4, 7, 4′-Trihydroxylated dihydrogenated flavone disulfate 2	C_15_H_14_O_10_S_2_	416.99460	416.99556	−2.30	9	7.477		▲		2
* M90	Dihydroxylated flavane disulfate 1	C_15_H_14_O_9_S_2_	400.99979	401.00065	−2.14	9	7.203	▲			3
* M91	Dihydroxylated flavane disulfate 2	C_15_H_14_O_9_S_2_	401.00009	401.00065	−1.40	9	7.724	▲			3
* M121	Dihydroxylated flavane glucuronide 1	C_21_H_22_O_9_	417.11819	417.11911	−2.21	11	8.534	▲			3
* M122	Dihydroxylated flavane glucuronide 2	C_21_H_22_O_9_	417.11807	417.11911	−2.49	11	8.595	▲			3
* M123	Dihydroxylated flavane glucuronide 3	C_21_H_22_O_9_	417.11801	417.11911	−2.64	11	8.877	▲			3
* M124	Dihydroxylated flavane glucuronide 4	C_21_H_22_O_9_	417.11816	417.11911	−2.28	11	8.904	▲	▲		3
* M125	Dihydroxylated flavane glucuronide 5	C_21_H_22_O_9_	417.11810	417.11911	−2.42	11	9.035	▲	▲		3
* M126	Dihydroxylated flavane glucuronide 6	C_21_H_22_O_9_	417.11819	417.11911	−2.21	11	9.499		▲		3
* M127	Dihydroxylated flavane glucuronide 7	C_21_H_22_O_9_	417.11821	417.11911	−2.16	11	9.642		▲		3
* M129	B-Ring methylated hydrogenated kaempferol glucuronide	C_22_H_22_O_12_	477.10306	477.10385	−1.66	12	9.201		▲		2
* M130	Methylated dedihydroxylated dihydrogenated kaempferol glucuronide 1	C_22_H_24_O_10_	447.12900	447.12967	−1.50	11	9.285	▲	▲		3
* M131	Methylated dedihydroxylated dihydrogenated kaempferol glucuronide 2	C_22_H_24_O_10_	447.12930	447.12967	−0.83	11	9.430	▲	▲		3
* M132	Methylated dedihydroxylated dihydrogenated kaempferol glucuronide 3	C_22_H_24_O_10_	447.12894	447.12967	−1.63	11	9.665	▲	▲		3
* M135	Methylated dehydroxylated naringenin glucuronide	C_22_H_22_O_10_	445.11316	445.11402	−1.93	12	11.017		▲		3
* M137	Dihydroxylated dihydrogenated flavane glucuronide 1	C_21_H_24_O_9_	419.13397	419.13476	−1.88	10	7.651		▲		3
* M138	Dihydroxylated dihydrogenated flavane glucuronide 2	C_21_H_24_O_9_	419.13403	419.13476	−1.74	10	7.731		▲		3
* M139	Dihydroxylated dihydrogenated flavane glucuronide 3	C_21_H_24_O_9_	419.13397	419.13476	−1.88	10	9.201	▲	▲		3
* M140	Dihydroxylated dihydrogenated flavane glucuronide 4	C_21_H_24_O_9_	419.13336	419.13476	−3.34	10	9.459	▲	▲		3
* M141	Dihydroxylated dihydrogenated flavane glucuronide 5	C_21_H_24_O_9_	419.13370	419.13476	−2.53	10	9.748	▲	▲		3
* M142	Dihydroxylated dihydrogenated flavane glucuronide 6	C_21_H_24_O_9_	419.13310	419.13476	−3.96	10	10.145		▲		3
* M143	Dimethylated apigenin glucuronide	C_23_H_22_O_11_	473.10779	473.10893	−2.41	13	10.299		▲		3
* M144	A-Ring methylated dihydrogenated apigenin glucuronide	C_22_H_24_O_11_	463.12418	463.12458	−0.86	11	9.962	▲			2
* M166	A-Ring methylated apigenin A-ring glucuronyl B-ring sulfate	C_22_H_20_O_14_S	539.04901	539.05010	−2.02	13	6.055		▲		2
* M178	Trihydroxylated dihydrogenated flavone glucuronyl sulfate 1	C_21_H_22_O_13_S	513.07007	513.07083	−1.48	11	7.048		▲		3
* M179	Trihydroxylated dihydrogenated flavone glucuronyl sulfate 2	C_21_H_22_O_13_S	513.07000	513.07083	−1.62	11	7.183		▲		3
* M180	Trihydroxylated dihydrogenated flavone glucuronyl sulfate 3	C_21_H_22_O_13_S	513.07007	513.07083	−1.48	11	7.408		▲		3
* M181	Trihydroxylated dihydrogenated flavone glucuronyl sulfate 4	C_21_H_22_O_13_S	513.06995	513.07083	−1.72	11	7.681		▲		3
* M182	Trihydroxylated dihydrogenated flavone glucuronyl sulfate 5	C_21_H_22_O_13_S	513.06909	513.07083	−3.39	11	8.522		▲		3
* M183	Trihydroxylated dihydrogenated flavone glucuronyl sulfate 6	C_21_H_22_O_13_S	513.07007	513.07083	−1.48	11	8.857		▲		3
* M184	A-Ring dehydroxylated apigenin glucuronyl sulfate 1	C_21_H_18_O_13_S	509.03869	509.03953	−1.65	13	5.417		▲		2
* M185	A-Ring dehydroxylated apigenin glucuronyl sulfate 2	C_21_H_18_O_13_S	509.03855	509.03953	−1.93	13	5.669		▲		2
* M186	A-Ring dehydroxylated apigenin glucuronyl sulfate 3	C_21_H_18_O_13_S	509.03870	509.03953	−1.63	13	5.800		▲		2
* M187	Dihydroxylated flavane glucuronyl sulfate 1	C_21_H_22_O_12_S	497.07629	497.07592	0.74	11	6.761	▲			3
* M188	Dihydroxylated flavane glucuronyl sulfate 2	C_21_H_22_O_12_S	497.07455	497.07592	−2.76	11	7.401	▲			3
* M189	Dihydroxylated flavane glucuronyl sulfate 3	C_21_H_22_O_12_S	497.07489	497.07592	−2.07	11	7.216		▲		3
* M190	Dihydroxylated flavane glucuronyl sulfate 4	C_21_H_22_O_12_S	497.07413	497.07592	−3.60	11	7.317		▲		3
* M191	A-Ring methylated dehydroxylated apiferol glucuronyl-4′-*O*-sulfate	C_22_H_24_O_13_S	527.08582	527.08648	−1.25	11	7.862		▲		2

Note: No.: number; MF: molecular formula; Meas.: measured; Pred.: predicted; Diff: difference; DBE: double bone equivalents; t*_R_*: retention time; P: plasma; U: urine; F: feces; CL: confidence levels; *: potential new compounds by retrieving information from the SciFinder database; ▲: detected.

## Data Availability

The original contributions presented in this study are included in the article. Further inquiries can be directed to corresponding authors.

## Supplementary Materials

The following supporting information can be downloaded at: https://www.mdpi.com/article/10.3390/molecules30224434/s1. Figure S1: Secondary mass spectrometry (MS^2^) data of M10–M12. Figure S2: Secondary mass spectrometry (MS^2^) data of M52–M55. Figures S3–S10: Characterization of phase I metabolites (M2–M9 and M18–M25). Figure S11: Characterization of glucose metabolites (M26 and M27). Figures S12–S18: Characterization of phase II metabolites: hydroxylated and methylated metabolites (containing M28–M35). Figures S19–S35: Characterization of phase II metabolites: sulfated metabolites (M36–M49, and M57–M92). Figures S36–S62: Characterization of phase II metabolites: glucuronidated metabolites (M93–M165). Figures S63–S71: Characterization of phase II metabolites: sulfated and glucuronidated metabolites (M166–M192). Figure S72: Results of GO enrichment analysis. Figure S73: The compound-target-pathway (C-T-P) network (Green nodes represent the targets; orange nodes represent the pathways, yellow nodes represent the compounds). Figure S74: Distribution of key targets in the PI3K/AKT signaling pathway. Figure S75: Molecular docking results of active ingredients (M98, M103, M104) with AKT1. Figure S76: Molecular docking results of active ingredients (M98, M103) with SRC. Figure S77: Molecular docking results of active ingredients (M51, M96, M100) with MMP9. Figure S78: Molecular docking results of active ingredients (M0, M16, M100) with EGFR. Figure S79: Molecular docking results of active ingredients (M13, M15, M25) with ESR1. Figure S80: Heat map of molecular docking scoring (AutoDock Vina). Table S1: Anticoagulant activity of kaempferitrin (KAE) on rabbit plasma recalcification time (PRT). Table S2: Antithrombotic activity of kaempferitrin (KAE) in vivo. Table S3: Tail vein bleeding time in rats treated with kaempferitrin (KAE). Table S4: The *p*-values and effect size of Cohen’s d for each endpoint. Table S5: The *p*-values of the Shapiro–Wilk test for each endpoint. Table S6: The *p*-values of Brown–Forsy test for different endpoints. Table S7: Power analysis results for each endpoint. Table S8: Detailed information of kaempferitrin (KAE) metabolites. Table S9: Core target information of kaempferitrin (KAE) and its metabolites. Table S10: Results of the biological process (BP) category terms from GO enrichment analysis. Table S11: Results of the cellular component (CC) category terms from GO enrichment analysis. Table S12: Results of the molecular function (MF) category terms from GO enrichment analysis. Table S13: Results of the pathways from KEGG enrichment analysis. Table S14: Details of each ligand–protein interaction molecular docking results. Table S15: Intersection of the top 6 “effective forms” scored by two molecular docking methods.

## Abbreviations

The following abbreviations are used in this manuscript:

ABCB1	Adenosine triphosphate binding cassette subfamily B member 1 gene
ABCG2	Breast cancer resistance protein
AKT1	Serine/threonine-protein kinase 1 gene
ANOVA	One-way analysis of variance
APP	Amyloid precursor protein
APTT	activated partial thromboplastin time
AR	Androgen receptor
BCL2	B-cell lymphoma-2
BP	Biological processes
BRAF	B-raf proto oncogene serine/threonine protein kinase
CaCl2	Calcium chloride
*C. orbiculatus*	*Celastrus orbiculatus* thunb.
CC	Cell composition
CLog P	Calculated n-octanol/water partition coefficient
CMC-Na	Sodium carboxymethyl cellulose
C-T-P	Compound-target-pathway
CVDs	Cardiovascular diseases
DAVID	Database for annotation, visualization and integrated discovery
DBE	Double bond equivalents
DMSO	Dimethyl sulfoxide
EGFR	Epidermal growth factor receptor
EICs	Extracted ion chromatograms
ELISA	Enzyme-linked immunosorbent assay
ESR1	Estrogen receptor 1
FeCl3	Ferric chloride
FIB	Fibrinogen
GAFF	General amber force field
GO	Gene ontology
GSK3B	Glycogen synthase kinase 3 beta gene
HIF-1	Hypoxia-inducible factor
HPLC	High-performance liquid chromatography
6-keto-PGF1α	6-keto-prostaglandin F1α
IL2	Interleukin-2
KAE	Kaempferitrin
KAE-H	Kaempferitrin high dose group
KAE-L	Kaempferitrin low dose group
KDR	Kinase domain-containing receptor
KEGG	Kyoto encyclopedia of genes and genomes
KIT	Kinase inserts domain receptor
LC-MS	Liquid chromatograph mass spectrometer
LSD	Least significant difference
MARK	Mitogen-activated protein kinase
MD	Molecular dynamics simulation
MeSH	Medical subject headings
MET	Mesenchymal to epithelial transition factor
MF	Molecular function
MMP2	Matrix metallopeptidase 2
MMP9	Matrix metallopeptidase 9
MPO	Myeloperoxidase
MF	Molecule formula
MS2	Secondary mass spectrometry
MW	Molecular weight
NADPH-oxidase	Nicotinamide adenine dinucleotide phosphate-oxidase
NF-κB	Nuclear factor kappa-B
NPT	Isothermal isobaric ensemble
NVT	Isothermal isovolumic ensemble
OMIM	Online mendelian inheritance in man
PAI-1	Plasminogen activator inhibitor
PDB	Protein data bank
PGI2	Prostacyclin
PIK3CA	Phosphatidylinositol-4, 5-bisphosphate-3-kinase catalytic subunit α
PIK3R1	Phosphoinositide-3-kinase regulatory subunit 1 gene
PIP2	Phosphatidylinositol-4, 5-bisphosphate
PKB	Protein kinase B
PLG	Plasminogen gene
PPARA	Peroxisome proliferator-activated receptor α
PPARG	Peroxisome proliferator activated receptor gamma gene
PPI	Protein–protein interaction
PRKACA	Cyclic adenosine monophosphate-dependent protein kinase catalytic subunit α
PRP	Platelet rich plasma
PRT	Plasma recalcification time
PT	Prothrombin time
PTGS2	Prostaglandin-endoperoxide synthase 2
RDA	Retro-Diels–Alder
RMSD	Root-mean-square deviation
RMSF	Root-mean-square fluctuation
SD	Standard deviation
SERPINE1	Plasminogen activator inhibitor 1
SMILES	Simplified molecular input line entry system
SO3	Sulfuric acid
SPSS	Statistical product and service solutions
SRC	Non-receptor tyrosine kinase gene
STRING	Search Tool for the Retrieval of Interacting Genes/Proteins
TCMs	Traditional Chinese Medicines
TERT	Telomerase reverse transcriptase
TNF	Tumor necrosis factor
t-PA	Tissue-type plasminogen activator
tR	Retention time
TT	Thrombin time
TXA2	Thromboxane A2
TXB2	Thromboxane B2
UHPLC	Ultra-high-performance liquid chromatography
VEGF	Vascular endothelial growth factor

## References

[1] Alkarithi G., Duval C., Shi Y., Macrae F.L., Ariens R.A.S. (2021). Thrombus Structural Composition in Cardiovascular Disease. Arterioscl. Throm. Vas..

[2] Schmaier A.A., Anderson P.F., Chen S.M., EI-Darzi E., Aivasovsky I., Kaushik M.P., Sack K.D., Hartzell H.C., Parikh S.M., Flaumenhaft R. (2023). TMEM16E regulates endothelial cell procoagulant activity and thrombosis. J. Clin. Investig..

[3] Hosokawa K., Ohnishi-Wada T., Nagasato T., Sameshima-Kaneko H., Oyamada C., Dahlen J. (2020). New methodological approaches for assessing thrombus formation in cardiovascular disease. Kardiol. Pol..

[4] Ząbczyk M., Ariëns R.A.S., Undas A. (2023). Fibrin clot properties in cardiovascular disease: From basic mechanisms to clinical practice. Cardiovasc. Res..

[5] Yang Y.W., Huang Z.T., Zhang X.J. (2021). Efficacy and safety of clopidogrel and/or aspirin for ischemic stroke/transient ischemic attack. Medicine.

[6] Yamamoto T., Abe K., Kodashima S. (2024). Gastrointestinal bleeding and mucosal injury induced by antithrombotic drugs. J. Gastroenterol..

[7] Bhatia K., Ladd L.M., Carr K.H., Di Napoli M., Saver J.L., McCullough L.D., Farahabadi M.H., Alsbrook D.L., Hinduja A., Ortiz Garcia J.G. (2023). Contemporary Antiplatelet and Anticoagulant Therapies for Secondary Stroke Prevention: A Narrative Review of Current Literature and Guidelines. Curr. Neurol. Neurosci. Rep..

[8] Kim T.-H., Lee K.M., Hong N.D., Jung Y.-S. (2016). Anti-platelet and anti-thrombotic effect of a traditional herbal medicine Kyung-Ok-Ko. J. Ethnopharmacol..

[9] Ye W.L., Wang J.J., Little P.J., Zou J.M., Zheng Z.H., Lu J., Yin Y.J., Liu H., Zhang D.M., Liu P.Q. (2024). Anti-atherosclerotic effects and molecular targets of ginkgolide B from Ginkgo biloba. Acta Pharm. Sin. B..

[10] Orgah J.O., He S., Wang Y., Jiang M.M., Wang Y.F., Orgah E.A., Duan Y.J., Zhao B.C., Han J.H., Zhu Y. (2020). Pharmacological potential of the combination of *Salvia miltiorrhiza* (Danshen) and *Carthamus tinctorius* (Honghua) for diabetes mellitus and its cardiovascular complications. Pharmacol. Res..

[11] Wang Y.H., Zhang X.T., Zhou C.X., Khan H., Fu M.Q., Cheang W.S. (2022). *Citri Reticulatae Pericarpium* (Chenpi) Protects against Endothelial Dysfunction and Vascular Inflammation in Diabetic Rats. Nutrients.

[12] Sánchez M., Romero M., Gómez-Guzmán M., Tamargo J., Pérez-Vizcaino F., Duarte J. (2019). Cardiovascular Effects of Flavonoids. Curr. Med. Chem..

[13] Wang L., Wu H.Y., Yang F., Dong W.B. (2019). The Protective Effects of Myricetin against Cardiovascular Disease. J. Nutr. Sci. Vitaminol..

[14] Chen Z., Zhang S.-L. (2021). The role of flavonoids in the prevention and management of cardiovascular complications: A narrative review. Ann. Palliat. Med..

[15] Rolnik A., Żuchowski J., Stochmal A., Olas B. (2020). Quercetin and kaempferol derivatives isolated from aerial parts of *Lens culinaris* Medik as modulators of blood platelet functions. Ind. Crops Prod..

[16] Wang S.B., Chae Y.H., Jang J.Y., Min J.H., Baek J.Y., Kim M., Park Y., Hwang G.S., Ryu J.S., Chang T.S. (2015). Kaempferol suppresses collagen-induced platelet activation by inhibiting NADPH oxidase and protecting SHP-2 from oxidative inactivation. Free Radic Biol Med..

[17] Feng Y., Chen Y., Xin H. (2017). Analysis of flavonoids in rosae laevigatae fructus by UPLC-Q-TOF-MS. Chin. J. Exp. Formulaics..

[18] Guo F.Y., Wang Y., Hao Y.J., Lu X.Y., Sang Y.L. (2017). Determination of four flavonoids in *Vicia amoena* Fisch. and Vicia amoena Fisch. var. *angusta* Freyn. by HPLC. Chin. J. Pharm. Anal..

[19] Zhou J.J., Zhai J.X., Zheng W.L., Han N., Liu Z.H., Lv G.H., Zheng X.J., Chang S., Yin J. (2019). The antithrombotic activity of the active fractions from the fruits of *Celastrus orbiculatus* Thunb through the anti-coagulation, anti-platelet activation and anti-fibrinolysis pathways. J. Ethnopharmacol..

[20] Wei P.P., Luo Q., Hou Y., Zhao F.L., Li F., Meng Q.G. (2024). *Houttuynia Cordata* Thunb.: A comprehensive review of traditional applications, phytochemistry, pharmacology and safety. Phytomedicine.

[21] Tang Y.T., Hou X.T., Du Z.C., Hao E.-W. (2021). Research progress on chemical constituents and pharmacological effects of Siraitiae Fructus and predictive analysis on quality markers. Chin. Tradit. Herbal. Drugs..

[22] Patel D.K. (2021). Pharmacological Activities and Therapeutic Potential of Kaempferitrin in Medicine for the Treatment of Human Disorders: A Review of Medicinal Importance and Health Benefits. Cardiovasc. Hematol. Disord. Drug Targets..

[23] Sousa E.D., Zanatta L., Seifriz I., Creczynski-Pasa T.B., Pizzolatti M.G., Szpoganicz B., Mena Barreto Silva F.R. (2004). Hypoglycemic effect and antioxidant potential of kaempferol-3,7-O-(alpha)-dirhamnoside from *Bauhinia forficata* leaves. J. Nat. Prod..

[24] Shi J., Zheng L., Lin Z.F., Hou C.Q., Liu W.Q., Yan T.M., Zhu L.J., Wang Y., Lu L.L., Liu Z.Q. (2015). Study of pharmacokinetic profiles and characteristics of active components and their metabolites in rat plasma following oral administration of the water extract of *Astragali radix* using UPLC–MS/MS. J. Ethnopharmacol..

[25] Li H.F., Xu F., Yang P., Liu G.X., Shang M.Y., Wang X., Yin J., Cai S.Q. (2017). Systematic screening and characterization of prototype constituents and metabolites of total astragalosides using HPLC-ESI-IT-TOF-MSn after oral administration to rats. J. Pharm. Biomed. Anal..

[26] Cai S.Q., Wang X., Shang M.Y., Xu F., Liu G.X. (2015). “Efficacy theory” may help to explain characteristic advantages of traditional Chinese medicines. Chin. J. Chin. Mater. Med..

[27] Chen M.J., Xiao J.B., El-Seedi H.R., Woźniak K.S., Daglia M., Little P.J., Weng J.P., Xu S.W. (2024). Kaempferol and atherosclerosis: From mechanism to medicine. Crit. Rev. Food Sci. Nutr..

[28] Bangar S.P., Chaudhary V., Sharma N., Bansal V., Ozogul F., Lorenzo J.M. (2023). Kaempferol: A flavonoid with wider biological activities and its applications. Crit. Rev. Food Sci. Nutr..

[29] Gogoi D., Chattopadhyay P., Dolui S.K., Khan M.R., Mukherjee A.K. (2024). Studies on in vivo antithrombotic activity of quercetin, a natural flavonoid isolated from a traditional medicinal plant, African eggplant (*Solanum indicum*). J. Ethnopharmacol..

[30] Senis Y.A., Mazharian A., Mori J. (2014). Src family kinases: At the forefront of platelet activation. Blood.

[31] Zhai Y.H., Yang J., Zhang J., Yang J., Li Q., Zheng T. (2021). Src-family Protein Tyrosine Kinases: A promising target for treating Cardiovascular Diseases. Int. J. Med. Sci..

[32] Nigam A., Manjuprasanna V.N., Naik M.U., Naik U.P. (2024). Platelet Spreading and Clot Retraction are Regulated by two Distinct *α*IIbβ3 Outside-in Signaling Pathways. J. Pharmacol. Exp. Ther..

[33] Song H.D., Yang Y.J., Li B. (2022). Tripeptide Hyp–Asp–Gly from collagen peptides inhibited platelet activation via regulation of PI3K/Akt–MAPK/ERK1/2 signaling pathway. J. Food Sci..

[34] Kwon H.W. (2019). Inhibitory Effects of Ginsenoside Ro on Clot Retraction through Suppressing PI3K/Akt Signaling Pathway in Human Platelets. Prev. Nutr. Food Sci..

[35] Xie Z.T., Liu B., Xiong Y.Y., Yang Y.F. (2020). Study of Components and Mechanism of Juechuang Against Platelet Aggregation Based on Network Pharmacology. Nat. Prod. Commun..

[36] Zakaria N.H., Tap F.M., Aljohani G.F., Abdul Majid F.A. (2024). Molecular docking and dynamics simulations revealed the potential inhibitory activity of honey-iQfood ingredients against GSK-3β and CDK5 protein targets for brain health. J. Biomol. Struct. Dyn..

[37] Liu Y., Xiong B.B., Qiu X., Hao H.Y., Sha A. (2022). Study on the antithrombotic effect and physiological mechanism of okanin. Biomed. Pharmacother..

[38] Chu L.X., Qin Y.Q., Zhou S.X., Yang F., He L.P., Liang Z.S., Mo C.G., Wang X.D. (2016). The effect of pravastatin on carotid artery thrombosis in rats under the stimulus of C-reactive protein. Thromb. Res..

[39] Wang S., Yao W., Zhu X.D., Wang J.J., Lu L.H., Zhu N., Lan T., Kuang Y.X., Zhu W.F., Liu R.H. (2023). Exploring the mechanism of the antithrombotic effects of *Pueraria lobata* and *Pueraria lobata var. thomsonii* based on network pharmacology. J. Ethnopharmacol..

[40] Chen G.L., Zeng R., Wang X., Cai H.Y., Chen J.J., Zhong Y.X., Zhong S.Y., Jia X.J. (2022). Antithrombotic Activity of Heparinoid G2 and Its Derivatives from the Clam *Coelomactra antiquata*. Mar. Drugs.

[41] Mustapha M., Nassir C.M.N.C.M., Aminuddin N., Safri A.A., Ghazali M.M. (2019). Cerebral Small Vessel Disease (CSVD)—Lessons From the Animal Models. Front. Physiol..

[42] Van den Kerkhof D.L., Nagy M., Wichapong K., Brouns S.L.N., Heemskerk J.W.M., Hackeng T.M., Dijkgraaf I. (2021). Inhibition of platelet adhesion, thrombus formation, and fibrin formation by a potent *α*IIbβ3 integrin inhibitor from ticks. Res. Pract. Thromb Hae..

[43] Ruan D.T., Lu R., Ruan K.-H. (2021). Redirecting thromboxane A2 and prostacyclin biosyntheses from thrombotic to antithrombotic property by an Enzymelink. Future Med. Chem..

[44] Zhang Y.Y., Jiang M., Wang J., Gao J.Y., Guo M., Liu J.X., Chen X.Y., Lang H.Y. (2021). The hemostatic mechanism of “Treated the Spleen” therapy on immune thrombocytopenia based on the characteristics of vasoactive factors. Ann. Palliat. Med..

[45] Zhou W.T., Abdurahman A., Umar A., Iskander G., Abdusalam E., Berke B., Begaud B., Moore N. (2014). Effects of *Cydonia oblonga* Miller extracts on blood hemostasis, coagulation and fibrinolysis in mice, and experimental thrombosis in rats. J. Ethnopharmacol..

[46] Calderón-Montaño J.M., Burgos-Morón E., Pérez-Guerrero C., López-Lázaro M. (2011). A review on the dietary flavonoid kaempferol. Mini Rev. Med. Chem..

[47] Choi J.H., Park S.E., Kim S.J., Kim S. (2015). Kaempferol inhibits thrombosis and platelet activation. Biochimie.

[48] Ding Y.H., Xiang Q., Zhu P.Y., Fan M.L., Tong H.Q., Wang M.X., Cheng S.Y., Yu P., Shi H.B., Zhang H.W. (2024). Qihuang Zhuyu formula alleviates coronary microthrombosis by inhibiting PI3K/Akt/*α*IIbβ3-mediated platelet activation. Phytomedicine.

[49] Lee D.H. (2020). Inhibitory effects of scoparone through regulation of PI3K/Akt and MAPK on collagen-induced human platelets. J. Appl. Biol. Chem..

[50] Su W., Chen Y., Wang C.H., Ding X., Rwibasira G., Kong Y. (2016). Human cathelicidin LL-37 inhibits platelet aggregation and thrombosis via Src/PI3K/Akt signaling. Biochem. Biophys. Res. Commun..

[51] Dhahri M., Rodriguez-Ruiz V., Aid-Launais R., Ollivier V., Pavon-Djavid G., Journe C., Louedec L., Chaubet F., Letourneur D., Maaroufi R.M. (2017). In vitro and in vivo hemocompatibility evaluation of a new dermatan sulfate-modified PET patch for vascular repair surgery. J. Biomed. Mater. Res. Part B Appl. Biomater..

[52] Lee K.O., Kwon I., Nam H.S., Park Y., Kim J., Shim Y., Erdenebileg Z., Cha M.J., Choi H.J., Choi H.Y. (2021). Effect of leukopenia induced by cyclophosphamide on the initial stage of arterial thrombosis in mice. Thromb. Res..

[53] Kim S., Chen J., Cheng T.J., Gindulyte A., He J., He S.Q., Li Q.L., Shoemaker B.A., Thiessen P.A., Yu B. (2021). PubChem in 2021: New data content and improved web interfaces. Nucleic Acids Res..

[54] Wu J.S., Zhang F.Q., Ruan H.N., Chang X.Y., Wang J.X., Li Z.Z., Jin W.Y., Shi Y. (2021). Integrating Network Pharmacology and RT-qPCR Analysis to Investigate the Mechanisms Underlying ZeXie Decoction-Mediated Treatment of Non-alcoholic Fatty Liver Disease. Front. Pharmacol..

[55] Huang D.W., Sherman B.T., Lempicki R.A. (2009). Systematic and integrative analysis of large gene lists using DAVID bioinformatics resources. Nat. Protoc..

[56] Zhang H.H., Yao S., Zhang Z.G., Zhou C.C., Fu F.D., Bian Y.S., Luo H., Li Y., Yan S.X., Ge Y.Y. (2021). Network Pharmacology and Experimental Validation to Reveal the Pharmacological Mechanisms of Liuwei Dihuang Decoction Against Intervertebral Disc Degeneration. Drug Des. Dev. Ther..

[57] Wang J.H., Zhao L.F., Wang H.-F., Wen Y.T., Jiang K.K., Mao X.M., Zhou Z.Y., Yao K.T., Geng Q.S., Guo D. (2019). GenCLiP 3: Mining human genes’ functions and regulatory networks from PubMed based on co-occurrences and natural language processing. Bioinformatics.

[58] Nogales C., Mamdouh Z.M., List M., Kiel C., Casas A., Schmidt H.H. (2022). Network pharmacology: Curing causal mechanisms instead of treating symptoms. Trends Pharmacol. Sci..

[59] Wang Y., Xu T.F., Chen X.Y., Ye Y., Liu L.Q., Wang Y.F., Zhang P. (2024). Network pharmacology and molecular docking approach to investigate the mechanism of a Chinese herbal formulation Yougui pills against steroid-related osteonecrosis of the femoral head. Arab. J. Chem..

[60] Fu F.Y., Huang Z.Q., Ye H.L., Tan B., Wang R.T., Chen W.H. (2020). Mechanisms and Molecular Targets of the Tao-Hong-Si-Wu-Tang Formula for Treatment of Osteonecrosis of Femoral Head: A Network Pharmacology Study. Evid. Based Complement. Alternat. Med..

[61] Jiang C.N., Meng A.G., Shi X.Y., Fu Z.P., Wang Y.L., Zhou J.J., Zhang X.W., Liu C.Y. (2024). Preparation of antioxidant peptides from yak skin gelatin and their protective effect on myocardial ischemia reperfusion injury. Food Funct..

[62] Abraham M.J., Murtola T., Schulz R., Pall S., Smith J.C., Hess B., Lindahl E. (2015). GROMACS: High performance molecular simulations through multi-level parallelism from laptops to supercomputers. SoftwareX.

[63] Van Der Spoel D., Lindahl E., Hess B., Groenhof G., Mark A.E., Berendsen H.J.C. (2005). GROMACS: Fast, flexible, and free. J. Comput. Chem..

[64] Collier T.A., Piggot T.J., Allison J.R. (2020). Molecular Dynamics Simulation of Proteins. Methods Mol. Biol..

