# Supplementary material for: Investigation of Antithrombotic Activity and In Vivo Effective Forms of Kaempferitrin Using FeCl3-Induced Rat Arterial Thrombosis and UHPLC-Q-Exactive Orbitrap MS
Source: Molecules. 2025 Nov 16;30(22):4434. doi: 10.3390/molecules30224434 (PMC12655163; doi:10.3390/molecules30224434)
Supplement: Supplementary file 1 [file molecules-30-04434-s001.zip › molecules-3896395-supplementary.pdf]

Supplementary Materials

Investigation of Antithrombotic Activity and In Vivo Effective Forms of Kaempferitrin Using $FeCl_3$-Induced Rat Arterial Thrombosis and UHPLC-Q-Exactive Orbitrap MS

Jingjing Zhou[1], Ruixin Wang[1], Jingchen Hou[1], Yitong Qi[1], Linying Niu[1], Xinyu Xia[1], Jinchen Shao[1], Yizhou Liu[1], Yanglu Liu[1], Chunyan Liu[1]*, Hongfu Li[1]*

[1] *School of Pharmacy, North China University of Science and Technology, 21 Bohai Avenue, Caofeidian New Town, Tangshan, 063210 Hebei Province, China.*

* Corresponding authors:

E-mail address: chunyanliu@ncst.edu.cn (C.Y. Liu); lihongfu6688@163.com (H.F. Li)

Content

1. Anticoagulant activity of kaempferitrin (KAE) on rabbit plasma recalcification time (PRT) with **Table S1**……(Page 1)

2. Antithrombotic activity of kaempferitrin (KAE) in vivo with **Table S2**……(Page 2)

3. Tail vein bleeding time in rats treated with kaempferitrin (KAE) with **Table S3**……(Page 3)

4. The *p*-values and effect size of Cohen's d for each endpoint with **Table S4**……(Pages 3-5)

5. The *p*-values of the Shapiro–Wilk test for each endpoint with **Table S5**……(Pages 5-6)

6. The *p*-values of Brown–Forsy test for different endpoints with **Table S6**……(Pages 6-7)

7. Power analysis results for each endpoint with **Table S7**……(Page 7)

8. Detailed information of kaempferitrin (KAE) metabolites with **Table S8**……(Pages 8-15)

9. Secondary mass spectrometry (MS^2) data of **M10–M12** with **Figure S1**……(Page 16)

10. Secondary mass spectrometry (MS^2) data of **M52**–**M55** with **Figure S2**……(Pages 17–18)

11. Characterization of phase I metabolites (**M2**–**M9** and **M18**–**M25**) with **Figures S3–10**……(Pages 18–24)

12. Characterization of glucose metabolites (**M26** and **M27**) with **Figure S11**……(Page 24)

13. Characterization of phase II metabolites: hydroxylated and methylated metabolites (containing **M28**–**M35**) with **Figures S12**–**18**……(Pages 24–28)

14. Characterization of phase II metabolites: sulfated metabolites (**M36–M49,** and **M57–M92**) with **Figures S19**–**35**……(Pages 28–39)

15. Characterization of phase II metabolites: glucuronidated metabolites (**M93**–**M165**) with **Figures S36–62**……(Pages 39–55)

16. Characterization of phase II metabolites: sulfated and glucuronidated metabolites (**M166**–**M192**) with **Figures S63**–**71**……(Pages 55–60)

17. Core target information of kaempferitrin (KAE) and its metabolites with **Table S9**……(Pages 60–61)

18. Results of the biological process (BP) category terms from GO enrichment analysis with **Table S10**……(Pages 62–76)

19. Results of the cellular component (CC) category terms from GO enrichment analysis with **Table S11**……(Pages 77–79)

20. Results of the molecular function (MF) category terms from GO enrichment analysis with **Table S12**……(Pages 80–83)

21. Results of GO enrichment analysis with **Figure S72**……(Page 84)

22. Results of the pathways from KEGG enrichment analysis with **Table S13**……(Pages 85–89)

23. Compound -target-pathway (C-T-P) network with **Figure S73**……(Page 89)

24. Distribution of key targets in the PI3K/AKT signaling pathway with **Figure S74**……(Page 90)

25. Details of each ligand–protein interaction molecular docking results with **Table S14**……(Pages 91–92)

26. Molecular docking results of active ingredients (**M98**, **M103**, **M104**) with AKT1 in **Figure S75**……(Page 92)

27. Molecular docking results of active ingredients (**M98**, **M103**) with SRC in **Figure S76**……(Page 93)

28. Molecular docking results of active ingredients (**M51**, **M96**, **M100**) with MMP9 in **Figure S77**……(Page 94)

29. Molecular docking results of active ingredients (**M0**, **M16**, **M100**) with EGFR in **Figure S78**……(Page 95)

30. Molecular docking results of active ingredients (**M13**, **M15**, **M25**) with ESR1 in **Figure S79**……(Page 96)

31. Molecular docking using AutoDock Vina……(Pages 96–98)

32. Heat map of molecular docking scoring (AutoDock Vina) with **Figure S80** ……(Page 99)

33. Intersection of the top 6 “effective forms” scored by two molecular docking methods with **Table S15**……(Pages 99–100)

1. Antithrombotic activities of KAE

Table S1

Anticoagulant activity of kaempferitrin (KAE) on rabbit plasma recalcification time (PRT).

Group	Dose (μg/mL, ×10^3)	Time (s)
Control	—	90.19 ± 0.78
Aspirin	6.67	138.56 ± 2.41**
KAE-L	3.33	118.51 ± 1.75$^{**\#}$
KAE-H	6.67	131.34 ± 2.50**

Note: Values are presented as mean ± SD (n = 6). One-way ANOVA followed by an LSD test was used to investigate significant differences between the groups. $^{**}p < 0.01$ vs. Control group, $^{\#}p < 0.05$, $^{\#\#}p < 0.01$ vs. Aspirin group. KAE-L: low-dose of kaempferitrin; KAE-H: high-dose of kaempferitrin.

Table S2

Antithrombotic activity of kaempferitrin (KAE) in vivo.

Group	Thrombus		Coagulation indices				Platelet function factors		Fibrinolytic system markers		
	Length (mm)	Wet weight (mg)	APTT (s)	PT (s)	TT (s)	FIB (g/L)	TXB_2 (ng/mL)	6-keto-PGF1α (ng/mL)	t-PA (μg/L)	PAI-1 (μg/L)	t-PA/PAI-1
Control	0 ± 0	0 ± 0	37.97 ± 3.11	9.03 ± 1.28	40.29 ± 3.07	1.03 ± 0.03	3.05 ± 0.19	3.40 ± 0.18	50.33 ± 6.90	2.68 ± 0.14	18.80± 6.42
Model	7.50 ± 1.29$^{##}$	3.65 ± 0.19$^{##}$	30.13 ± 1.11$^{##}$	8.15 ± 1.21$^{##}$	32.35 ± 2.05$^{##}$	1.78 ± 0.04$^{##}$	3.62 ± 0.17$^{##}$	2.43 ± 0.08$^{##}$	41.83 ± 3.42$^{##}$	3.16 ± 0.17$^{##}$	13.35± 4.02$^{##}$
Aspirin	4.25 ± 1.15**	0.88 ± 0.31**	46.03 ± 1.21**	9.83 ± 0.21**	51.17 ± 1.46**	0.89 ± 0.05**	3.10 ± 0.10**	3.57 ± 0.21**	61.58 ± 1.11**	2.52 ± 0.23**	24.40 ± 1.05**
KAE-L	6.02 ± 0.82*	2.10 ± 0.18*	38.20 ± 2.05*	8.44 ± 1.02	42.73 ± 1.56**	1.42 ± 0.06*	3.47 ± 0.06	3.27 ± 0.11**	49.02 ± 2.24*	2.89 ± 0.42*	16.94 ± 2.45*
KAE-H	5.00 ± 1.03*	1.01 ± 0.17**	40.03 ± 0.32**	9.23 ± 1.05*	48.42 ± 2.68**	1.01 ± 0.05**	3.18 ± 0.05*	3.70 ± 0.31**	57.08 ± 2.15**	2.70 ± 0.15**	21.14 ± 2.31**

Note: Values are presented as mean ± SD (n = 6). One-way ANOVA followed by an LSD test was used to investigate significant differences between the groups. $^{##}p < 0.01$ vs. Control group; $^{*}p < 0.05$, $^{**}p < 0.01$ vs. Model group. APTT: activated partial thromboplastin time; FIB: fibrinogen; KAE-H: high-dose of kaempferitrin (60 mg/kg); KAE-L: low-dose of kaempferitrin (30 mg/kg); 6-keto-PGF1α: 6-keto-prostaglandin F1α; PAI-1: plasminogen activator inhibitor; PT: prothrombin time; t-PA: tissue-type plasminogen activator; TT: thrombin time; TXB_2: Thromboxane B2.

Table S3

Tail vein bleeding time in rats treated with kaempferitrin (KAE).

Groups	Bleeding time (s)						
	Rat 1	Rat 2	Rat 3	Rat 4	Rat 5	Rat 6	Average
Control	167.22	194.02	273.45	208.29	237.71	224.31	217.5±36.74
Aspirin	967.26	960.43	1010.95	987.58	829.59	909.66	944.25±65.51$^{\#\#}$
KAE-L	320.07	297.55	328.52	295.32	307.55	225.51	295.75±36.72**
KAE-H	345.18	288.85	342.15	272.93	321.11	298.75	311.50±29.43**

Note: Values are presented as mean ± SD (n = 6). One-way ANOVA followed by an LSD test was used to investigate significant differences between the groups. $^{\#\#}p < 0.01$ vs. Control group; $^{**}p < 0.01$ vs. Aspirin group. KAE-H: high-dose of kaempferitrin (60 mg/kg); KAE-L: low-dose of kaempferitrin (30 mg/kg).

Table S4

The *p*-values and effect size of Cohen's d for each endpoint.

Endpoints	Comparison group	*p* (LSD)	*p* (Tukey)	Cohen's d
PRT	Control vs Aspirin	< 0.0001	< 0.0001	0.82
	Control vs KAE-L	< 0.0001	0.0025	
	Aspirin vs KAE-L	0.0180	0.0225	
	Control vs KAE-H	0.0045	0.0034	
	Aspirin vs KAE-H	0.0724	0.0658	
Thrombus length	Control vs Model	< 0.0001	< 0.0001	1.00

	Model vs Aspirin	< 0.0001	< 0.0001	
	Model vs KAE-L	0.0223	0.0325	
	Model vs KAE-H	0.0113	0.0122	
Thrombus wet weight	Control vs Model	< 0.0001	< 0.0001	1.00
	Model vs Aspirin	< 0.0001	0.0021	
	Model vs KAE-L	0.0150	0.0125	
	Model vs KAE-H	< 0.0001	0.0026	
APTT	Control vs Model	0.0091	0.0012	1.00
	Model vs Aspirin	< 0.0001	< 0.0001	
	Model vs KAE-L	0.0142	0.0189	
	Model vs KAE-H	< 0.0001	0.0035	
PT	Control vs Model	0.0088	0.0065	0.82
	Model vs Aspirin	0.0075	0.0066	
	Model vs KAE-L	0.1052	0.1165	
	Model vs KAE-H	0.0402	0.0365	
TT	Control vs Model	< 0.0001	< 0.0001	1.00
	Model vs Aspirin	< 0.0001	< 0.0001	
	Model vs KAE-L	< 0.0001	0.0011	
	Model vs KAE-H	< 0.0001	0.0006	
FIB	Control vs Model	< 0.0001	< 0.0001	0.81
	Model vs Aspirin	< 0.0001	< 0.0001	
	Model vs KAE-L	0.0105	0.0113	
	Model vs KAE-H	< 0.0001	< 0.0001	

TXB_2	Control vs Model	< 0.0001	< 0.0001	0.95
	Model vs Aspirin	< 0.0001	< 0.0001	
	Model vs KAE-L	0.2779	0.0847	
	Model vs KAE-H	0.0201	0.0151	
6-keto-PGFα	Control vs Model	< 0.0001	< 0.0001	0.97
	Model vs Aspirin	< 0.0001	< 0.0001	
	Model vs KAE-L	< 0.0001	< 0.0001	
	Model vs KAE-H	< 0.0001	< 0.0001	
t-PA	Control vs Model	0.0050	0.0025	1.0
	Model vs Aspirin	< 0.0001	0.0050	
	Model vs KAE-L	0.0211	0.0175	
	Model vs KAE-H	< 0.0001	< 0.0001	
PAI-1	Control vs Model	0.0184	0.0204	1.0
	Model vs Aspirin	0.0012	0.0035	
	Model vs KAE-L	0.0350	0.0426	
	Model vs KAE-H	0.0026	0.0035	
t-PA/PAI-1	Control vs Model	0.0011	< 0.0001	0.89
	Model vs Aspirin	0.0002	< 0.0001	
	Model vs KAE-L	0.0106	0.0125	
	Model vs KAE-H	0.0021	0.0054	
Bleeding time	Control vs Aspirin	< 0.0001	< 0.0001	0.96
	Aspirin vs KAE-L	< 0.0001	0.0025	
	Aspirin vs KAE-H	< 0.0001	0.0034	

Note: LSD: Least significant difference; PRT: plasma recalcification time; APTT: activated partial thromboplastin time; FIB: fibrinogen; KAE-H: high-dose of kaempferitrin (60 mg/kg); KAE-L: low-dose of kaempferitrin (30 mg/kg); 6-keto-PGF1α: 6-keto-prostaglandin F1α; PAI-1: plasminogen activator inhibitor; PT: prothrombin time; t-PA: tissue-type plasminogen activator; TT: thrombin time; TXB_2: Thromboxane B2.

Table S5

The *p*-values of the Shapiro–Wilk test for each endpoint.

Endpoints	Control	Model	Aspirin	KAE-L	KAE-H
PRT	0.4703	—	0.0527	0.5727	0.1465
Thrombus length	0.0783	0.5692	0.2104	0.5506	0.0693
Thrombus wet weight	0.1042	0.4821	0.0618	0.3590	0.3218
APTT	0.0543	0.1615	0.4605	0.8160	0.4006
PT	0.0546	0.0558	0.0741	0.1144	0.0845
TT	0.2023	0.0660	0.0519	0.4803	0.9538
FIB	0.1483	0.8165	0.4815	0.5790	0.6412
TXB_2	0.1512	0.9766	0.3465	0.7095	0.8222
6-keto-PGFα	0.3234	0.7943	0.1096	0.7117	0.0522
t-PA	0.0602	0.1555	0.5089	0.9602	0.3928
PAI-1	0.4674	0.1190	0.9490	0.0548	0.3510
t-PA/PAI-1	0.1865	0.0548	0.3006	0.4015	0.0860
Bleeding time	0.9985	—	0.3822	0.0777	0.5659

Note: APTT: activated partial thromboplastin time; PRT: plasma recalcification time; FIB: fibrinogen; KAE-H: high-dose of kaempferitrin (60 mg/kg); KAE-L: low-dose of kaempferitrin (30 mg/kg); 6-keto-PGF1α: 6-keto-prostaglandin F1α; PAI-1: plasminogen activator inhibitor; PT: prothrombin time; t-PA: tissue-type plasminogen activator; TT: thrombin time; TXB_2: Thromboxane B2.

Table S6

The *p*-values of the Brown–Forsy test for different endpoints.

**Endpoints**	***p*-values of Brown–Forsy test**
PRT	0.0681
Platelet aggregation rate	0.5971
Thrombus length	0.0503
Thrombus wet weight	0.0545
APTT	0.2122
PT	0.0615
TT	0.1918
FIB	0.3619
TXB_2	0.1041
6-keto-PGFα	0.4107
t-PA	0.0735
PAI-1	0.5919
t-PA/PAI-1	0.0814

Bleeding time	0.6103

Note: PRT: plasma recalcification time; APTT: activated partial thromboplastin time; PT: prothrombin time; TT: thrombin time; FIB: fibrinogen; TXB_2: Thromboxane B2; 6-keto-PGF1α: 6-keto-prostaglandin F1α; t-PA: tissue-type plasminogen activator; PAI-1: plasminogen activator inhibitor.

Table S7

Power analysis results for each endpoint.

**Endpoints**	**Significance level (*α*)**	**Effect size (f)**	**Sample size /group (n)**	**Power (1-*β*)**
Thrombus length	0.05	2.52	6	1.00
Thrombus weight	0.05	1.26	6	1.00
APTT	0.05	5.09	6	1.00
PT	0.05	3.26	6	0.82
TT	0.05	6.59	6	1.00
FIB	0.05	4.25	6	0.81

Note: APTT: activated partial thromboplastin time; PT: prothrombin time; TT: thrombin time; FIB: fibrinogen

2. Analysis of the metabolites of KAE

Table S8

Detailed information of kaempferitrin (KAE) and its metabolites.

No.	Name	MF	Meas. (Da)	Pred. (Da)	Diff (ppm)	DBE	t_R (min)	P	U	F	CL
**M2**	B-Ring hydroxylated apigenin	$C_{15}H_{10}O_6$	285.04041	285.04046	–0.18	11	10.535		▲		2
**M3**	Apiforol isomer 1	$C_{15}H_{14}O_5$	273.07678	273.07685	–0.26	9	9.636			▲	3
**M4**	Apiforol isomer 2	$C_{15}H_{14}O_5$	273.07684	273.07685	–0.04	9	11.185			▲	3
**M5**	Apiforol isomer 3	$C_{15}H_{14}O_5$	273.07693	273.07685	0.29	9	11.379			▲	3
**M6**	Apiforol	$C_{15}H_{14}O_5$	273.07648	273.07685	–1.35	9	11.712			▲	2
**M7**	Naringenin	$C_{15}H_{12}O_5$	271.06107	271.06120	–0.48	10	11.871		▲	▲	2
**M8**	Naringenin isomer 1	$C_{15}H_{12}O_5$	271.06100	271.06120	–0.74	10	11.478		▲	▲	3
**M9**	Naringenin isomer 2	$C_{15}H_{12}O_5$	271.06116	271.06120	–0.15	10	9.079			▲	3
**M10**	Galangin	$C_{15}H_{10}O_5$	269.04575	269.04555	0.74	11	9.285			▲	2

**M11**	Apigenin	$C_{15}H_{10}O_5$	269.04568	269.04555	0.48	11	11.927	▲	▲	▲	1
**M12**	A-Ring dehydroxylated apigenin	$C_{15}H_{10}O_4$	253.05070	253.05063	0.28	11	10.276	▲	▲		2
**M13**	4, 7, 4'-Trihydroxylated dihydrogenated flavone	$C_{15}H_{14}O_4$	257.08182	257.08193	–0.43	9	10.490			▲	2
**M14**	5, 7, 4'-Trihydroxylated dihydrogenated flavone	$C_{15}H_{14}O_4$	257.08173	257.08193	–0.78	9	12.325			▲	2
**M15**	4, 5, 4'-Trihydroxylated dihydrogenated flavone	$C_{15}H_{14}O_4$	257.08185	257.08193	–0.31	9	12.439			▲	2
**M18**	Quercetin	$C_{15}H_{10}O_7$	301.03525	301.03538	–0.43	11	10.846			▲	2
**M19**	B-Ring hydroxylated kaempferol	$C_{15}H_{10}O_7$	301.03522	301.03538	–0.53	11	9.595			▲	2
**M20**	A-Ring dehydroxylated naringenin	$C_{15}H_{12}O_4$	255.06622	255.06628	–0.24	10	10.340			▲	2
**M21**	Hydrogenated kaempferol	$C_{15}H_{12}O_6$	287.05585	287.05611	–0.91	10	9.440			▲	3
**M22**	Hydrogenated kaempferol isomer 1	$C_{15}H_{12}O_6$	287.05609	287.05611	–0.07	10	10.015			▲	3
**M23**	Hydrogenated kaempferol isomer 2	$C_{15}H_{12}O_6$	287.05597	287.05611	–0.49	10	10.376			▲	3
**M24**	Dihydrogenated kaempferol	$C_{15}H_{14}O_6$	289.07156	289.07176	–0.69	9	9.348			▲	3
**M25**	2-Aryl-3-aminobenzofurans, and 2-aryl-3(2H)-benzofuranones	$C_{14}H_{10}O_5$	257.04550	257.04555	–0.19	10	11.077			▲	2
**M26**	Apigenin glucoside 1	$C_{22}H_{22}O_{12}$	477.10318	477.10385	–1.40	13	8.137			▲	3

**M27**	Apigenin glucoside 2	$C_{22}H_{22}O_{12}$	477.10303	477.10385	–1.72	13	8.208			▲	3
**M28**	5-*O*-Methylated kaempferol	$C_{16}H_{12}O_6$	299.05569	299.05611	–1.40	11	12.082		▲		2
**M29**	7-*O*-Methylated kaempferol	$C_{16}H_{12}O_6$	299.05621	299.05611	0.33	11	10.089		▲		2
**M30**	4'-*O*-Methylated hydrogenated kaempferol	$C_{16}H_{14}O_6$	301.07138	301.07176	–1.26	10	10.098		▲		2
**M31**	Dimethylated quercetin	$C_{17}H_{14}O_7$	329.06644	329.06668	–0.73	11	11.993		▲	▲	3
**M32**	A-Ring methylated quercetin	$C_{16}H_{12}O_7$	315.05099	315.05103	–0.13	11	10.724			▲	2
**M33**	A-Ring methylated hydrogenated quercetin	$C_{16}H_{14}O_7$	317.06662	317.06668	–0.19	10	12.296			▲	2
**M34**	A-Ring methylated hydroxylated hydrogenated quercetin	$C_{16}H_{14}O_8$	333.06152	333.06159	–0.21	10	10.413			▲	2
**M35**	A-Ring methylated apigenin	$C_{16}H_{12}O_5$	283.06100	283.06120	–0.71	11	10.456			▲	2
**M36**	A-Ring methylated apigenin-4'-*O*-sulfate	$C_{16}H_{12}O_8S$	363.01724	363.01801	–2.12	11	8.621		▲	▲	2
**M37**	A-Ring methylated apigenin sulfate 1	$C_{16}H_{12}O_8S$	363.01685	363.01801	–3.20	11	8.400	▲			2
**M38**	A-Ring methylated apigenin sulfate 2	$C_{16}H_{12}O_8S$	363.01758	363.01801	–1.18	11	8.728	▲			2
**M39**	Kaempferol sulfate 1	$C_{15}H_{10}O_9S$	364.99634	364.99728	–2.58	11	9.692		▲		3
**M40**	Kaempferol sulfate 2	$C_{15}H_{10}O_9S$	364.99603	364.99728	–3.42	11	9.998	▲	▲	▲	3

**M41**	Naringenin-7-*O*-sulfate	$C_{15}H_{12}O_8S$	351.01761	351.01801	–1.14	10	8.218		▲		2
**M42**	Naringenin isomer sulfate	$C_{15}H_{12}O_8S$	351.01709	351.01801	–2.62	10	9.362		▲		3
**M43**	Naringenin-4'-*O*-sulfate	$C_{15}H_{12}O_8S$	351.01727	351.01801	–2.11	10	9.903		▲		2
**M44**	Naringenin-5-*O*-sulfate	$C_{15}H_{12}O_8S$	351.01764	351.01801	–1.05	10	6.385		▲	▲	2
**M45**	Apigenin sulfate 1	$C_{15}H_{10}O_8S$	349.00110	349.00236	–3.61	11	7.086	▲		▲	3
**M46**	Apigenin sulfate 2	$C_{15}H_{10}O_8S$	349.00116	349.00236	–3.44	11	9.444		▲		3
**M47**	Apigenin sulfate 3	$C_{15}H_{10}O_8S$	349.00137	349.00236	–2.84	11	8.227		▲		3
**M48**	Apigenin sulfate 4	$C_{15}H_{10}O_8S$	349.00112	349.00236	–3.55	11	9.612	▲	▲	▲	3
**M49**	Apigenin sulfate 5	$C_{15}H_{10}O_8S$	349.00121	349.00236	–3.30	11	9.786	▲			3
**M52**	4, 7, 4'-Trihydroxylated dihydrogenated flavone-4/7-*O*-sulfate	$C_{15}H_{14}O_7S$	337.03943	337.03875	2.02	9	8.609			▲	2
**M55**	4, 7, 4'-Trihydroxylated dihydrogenated flavone sulfate	$C_{15}H_{14}O_7S$	337.04041	337.03875	4.93	9	9.473	▲	▲		2
**M57**	Dihydroxylated flavanones sulfate 1	$C_{15}H_{12}O_7S$	335.02246	335.02310	–1.91	10	7.832		▲		3
**M58**	Dihydroxylated flavanones-4'-*O*-sulfate	$C_{15}H_{12}O_7S$	335.02274	335.02310	–1.07	10	9.430		▲		2

**M59**	Dihydroxylated flavanones sulfate 2	$C_{15}H_{12}O_7S$	335.02271	335.02310	–1.16	10	8.724	▲		▲	3
**M60**	Dihydroxylated flavanones sulfate 3	$C_{15}H_{12}O_7S$	335.02298	335.02310	–0.36	10	9.577	▲			3
**M61**	Dihydroxylated flavanones sulfate 4	$C_{15}H_{12}O_7S$	335.02287	335.02310	–0.69	10	8.536			▲	3
**M62**	A-Ring dehydroxylated apigenin sulfate 1	$C_{15}H_{10}O_7S$	333.00708	333.00745	–1.11	11	8.177	▲	▲		2
**M63**	A-Ring dehydroxylated apigenin sulfate 2	$C_{15}H_{10}O_7S$	333.00770	333.00745	0.75	11	8.225		▲		2
**M64**	A-Ring dehydroxylated apigenin sulfate 3	$C_{15}H_{10}O_7S$	333.00699	333.00745	–1.38	11	8.386		▲		2
**M65**	A-Ring dehydroxylated apigenin sulfate 4	$C_{15}H_{10}O_7S$	333.00772	333.00745	0.81	11	8.490	▲		▲	2
**M66**	A-Ring dehydroxylated apigenin sulfate 5	$C_{15}H_{10}O_7S$	333.00721	333.00745	–0.72	11	8.522	▲		▲	2
**M70**	Dimethylated kaempferol sulfate	$C_{17}H_{14}O_9S$	393.02817	393.02858	–1.04	11	8.853		▲		3
**M71**	Hydrogenated kaempferol sulfate	$C_{15}H_{12}O_9S$	367.01160	367.01293	–3.62	10	6.897		▲		3
**M80**	A, B-Rings of apigenin disulfate 1	$C_{15}H_{10}O_{11}S_2$	428.95834	428.95918	–1.96	11	7.622	▲	▲		2
**M81**	A, B-Rings of apigenin disulfate 2	$C_{15}H_{10}O_{11}S_2$	428.95844	428.95918	–1.73	11	8.013	▲			2
**M85**	Dehydroxylated apigenin A, B-rings disulfate 1	$C_{15}H_{10}O_{10}S_2$	412.96323	412.96426	–2.49	11	6.623		▲		2
**M86**	Dehydroxylated apigenin A, B-rings disulfate 2	$C_{15}H_{10}O_{10}S_2$	412.96338	412.96426	–2.13	11	6.744		▲		2

**M87**	Dehydroxylated apigenin A, B-rings disulfate 3	$C_{15}H_{10}O_{10}S_2$	412.96359	412.96426	–1.62	11	6.801	▲	▲	2
**M88**	Dehydroxylated apigenin A, B-rings disulfate 4	$C_{15}H_{10}O_{10}S_2$	412.96328	412.96426	–2.37	11	6.415	▲		2
**M89**	Dehydroxylated apigenin A, B-rings disulfate 5	$C_{15}H_{10}O_{10}S_2$	412.96367	412.96426	–1.43	11	7.146	▲		2
**M92**	Ethyl-phenol disulfate	$C_8H_{10}O_7S_2$	280.97919	280.97952	–1.17	4	8.992	▲		2
**M93**	Methylated apigenin glucuronide 1	$C_{22}H_{20}O_{11}$	459.09210	459.09328	–2.57	13	7.263	▲	▲	3
**M94**	Methylated apigenin glucuronide 2	$C_{22}H_{20}O_{11}$	459.09250	459.09328	–1.70	13	6.943	▲		3
**M95**	Kaempferol-5-*O*-glucuronide	$C_{21}H_{18}O_{12}$	461.07139	461.07255	–2.52	13	8.448	▲	▲	2
**M96**	Kaempferol-3-*O*-glucuronide	$C_{21}H_{18}O_{12}$	461.07178	461.07255	–1.67	13	8.670	▲	▲	2
**M97**	Kaempferol-4'-*O*-glucuronide	$C_{21}H_{18}O_{12}$	461.07153	461.07255	–2.21	13	8.879	▲	▲	2
**M98**	Kaempferol-7-*O*-glucuronide	$C_{21}H_{18}O_{12}$	461.07141	461.07255	–2.47	13	9.126	▲	▲	2
**M99**	Naringin-5-*O*-glucuronide	$C_{21}H_{20}O_{11}$	447.09256	447.09328	–1.61	12	8.960	▲		2
**M100**	Naringin-7-*O*-glucuronide	$C_{21}H_{20}O_{11}$	447.09262	447.09328	–1.48	12	8.882	▲	▲	2
**M101**	Naringin-4'-*O*-glucuronide	$C_{21}H_{20}O_{11}$	447.09283	447.09328	–1.01	12	9.017	▲	▲	2
**M102**	Naringin isomer glucuronide	$C_{21}H_{20}O_{11}$	447.09265	447.09328	–1.41	12	9.126	▲	▲	3

**M103**	Apigenin-5-*O*-glucuronide	$C_{21}H_{18}O_{11}$	445.07663	445.07763	–2.25	13	8.108	▲	▲	2
**M104**	Apigenin-7-*O*-glucuronide	$C_{21}H_{18}O_{11}$	445.07791	445.07763	0.63	13	8.509	▲	▲	2
**M105**	Apigenin-4'-*O*-glucuronide	$C_{21}H_{18}O_{11}$	445.07675	445.07763	–1.98	13	8.355	▲	▲	2
**M106**	Apigenin glucuronide	$C_{21}H_{18}O_{11}$	445.07672	445.07763	–2.04	13	8.232	▲	▲	3
**M107**	Trihydroxylated dihydrogenated flavone glucuronide 1	$C_{21}H_{22}O_{10}$	433.11279	433.11402	–2.84	12	9.007		▲	3
**M108**	Trihydroxylated dihydrogenated flavone glucuronide 2	$C_{21}H_{22}O_{10}$	433.11325	433.11402	–1.78	12	9.884		▲	3
**M109**	Trihydroxylated dihydrogenated flavone glucuronide 3	$C_{21}H_{22}O_{10}$	433.11401	433.11402	–0.02	12	10.098	▲	▲	3
**M110**	Trihydroxylated dihydrogenated flavone glucuronide 4	$C_{21}H_{22}O_{10}$	433.11316	433.11402	–1.99	12	10.149	▲	▲	3
**M111**	Dehydroxylated naringin glucuronide 1	$C_{21}H_{20}O_{10}$	431.09702	431.09837	–3.13	12	7.459	▲	▲	3
**M112**	Dehydroxylated naringin glucuronide 2	$C_{21}H_{20}O_{10}$	431.09750	431.09837	–2.02	12	7.514	▲	▲	3
**M113**	Dehydroxylated naringin glucuronide 3	$C_{21}H_{20}O_{10}$	431.09744	431.09837	–2.16	12	7.946	▲	▲	3
**M114**	Dehydroxylated naringin glucuronide 4	$C_{21}H_{20}O_{10}$	431.09763	431.09837	–1.72	12	8.086	▲	▲	3

**M115**	Dehydroxylated naringin glucuronide 5	$C_{21}H_{20}O_{10}$	431.09757	431.09837	–1.86	12	11.151		▲	3
**M116**	Dehydroxylated apigenin glucuronide 1	$C_{21}H_{18}O_{10}$	429.08194	429.08272	–1.82	13	6.409	▲		3
**M117**	Dehydroxylated apigenin glucuronide 2	$C_{21}H_{18}O_{10}$	429.08136	429.08272	–3.17	13	6.780	▲		3
**M118**	Dehydroxylated apigenin glucuronide 3	$C_{21}H_{18}O_{10}$	429.08173	429.08272	–2.31	13	7.008		▲	3
**M119**	Dehydroxylated apigenin glucuronide 4	$C_{21}H_{18}O_{10}$	429.08191	429.08272	–1.89	13	7.099	▲	▲	3
**M120**	Dehydroxylated apigenin glucuronide 5	$C_{21}H_{18}O_{10}$	429.08212	429.08272	–1.40	13	7.855	▲	▲	3
**M128**	A, B-Rings dimethylated kaempferol glucuronide	$C_{23}H_{22}O_{12}$	489.10278	489.10385	–2.19	13	7.499		▲	2
**M133**	Hydrogenated kaempferol glucuronide 1	$C_{21}H_{20}O_{12}$	463.08707	463.08820	–2.44	12	6.241		▲	3
**M134**	Hydrogenated kaempferol glucuronide 2	$C_{21}H_{20}O_{12}$	463.08762	463.08820	–1.25	12	6.616		▲	3
**M136**	Methylated dehydroxylated apigenin glucuronide	$C_{22}H_{20}O_{10}$	443.09756	443.09837	–1.83	13	9.795		▲	3
**M146**	Apigenin diglucuronide	$C_{27}H_{26}O_{17}$	621.10858	621.10972	–1.84	15	6.221		▲	3
**M147**	A-Ring dehydroxylated apigenin glucuronide methylated rhamnose	$C_{28}H_{30}O_{14}$	589.15517	589.15623	–1.80	13	7.121		▲	2
**M148**	Phenol glucuronide 1	$C_{12}H_{14}O_{7}$	269.06685	269.06668	0.63	6	5.318		▲	3
**M149**	Phenol glucuronide 2	$C_{12}H_{14}O_{7}$	269.06686	269.06668	0.67	6	5.492		▲	3

**M150**	Hydroxystyrene glucuronide	$C_{14}H_{16}O_7$	295.08185	295.08233	–1.63	7	8.300		▲	2
**M151**	Methylated phenol glucuronide 1	$C_{13}H_{16}O_7$	283.08182	283.08233	–1.80	6	7.317		▲	3
**M152**	Methylated phenol glucuronide2	$C_{13}H_{16}O_7$	283.08179	283.08233	–1.91	6	6.603	▲		3
**M153**	Methylated phenol glucuronide 3	$C_{13}H_{16}O_7$	283.08200	283.08233	–1.17	6	6.933	▲		3
**M154**	Methylated phenol glucuronide 4	$C_{13}H_{16}O_7$	283.08218	283.08233	–0.53	6	7.241	▲		3
**M155**	Methylated phenol glucuronide 5	$C_{13}H_{16}O_7$	283.08211	283.08233	–0.78	6	7.401	▲		3
**M156**	Hydroxyethylbenzene glucuronide 1	$C_{14}H_{18}O_7$	297.09750	297.09798	–1.62	6	8.888	▲		3
**M157**	Hydroxyethylbenzene glucuronide 2	$C_{14}H_{18}O_7$	297.09732	297.09798	–2.22	6	8.947	▲	▲	3
**M158**	Hydroxybenzyl alcohol glucuronide 1	$C_{13}H_{16}O_8$	299.07684	299.07724	–1.34	6	6.278		▲	3
**M159**	Hydroxybenzyl alcohol glucuronide 2	$C_{13}H_{16}O_8$	299.07770	299.07724	1.54	6	6.931		▲	3
**M160**	Hydroxyphenylethanol glucuronide 1	$C_{14}H_{18}O_8$	313.09244	313.09289	–1.44	6	7.695		▲	3
**M161**	Hydroxyphenylethanol glucuronide 2	$C_{14}H_{18}O_8$	313.09268	313.09289	–0.67	6	8.452		▲	3
**M162**	Hydroxybenzaldehyde glucuronide 1	$C_{13}H_{14}O_8$	297.06085	297.06159	–2.49	7	5.816		▲	3
**M163**	Hydroxybenzaldehyde glucuronide 2	$C_{13}H_{14}O_8$	297.06140	297.06159	–0.64	7	5.976		▲	3

**M164**	Hydroxybenzaldehyde glucuronid 3	$C_{13}H_{14}O_8$	297.06137	297.06159	–0.74	7	6.425		▲	3
**M165**	Hydroxybenzaldehyde glucuronide 4	$C_{13}H_{14}O_8$	297.06132	297.06159	–0.91	7	6.478		▲	3
**M167**	Kaempferol glucuronyl sulfate 1	$C_{21}H_{18}O_{15}S$	541.02771	541.02936	–3.05	13	7.206		▲	3
**M168**	Kaempferol glucuronyl sulfate 2	$C_{21}H_{18}O_{15}S$	541.02808	541.02936	–2.37	13	7.587	▲		3
**M169**	Kaempferol glucuronyl sulfate 3	$C_{21}H_{18}O_{15}S$	541.02960	541.02936	0.44	13	6.960	▲		3
**M170**	Naringenin glucuronyl sulfate	$C_{21}H_{20}O_{14}S$	527.04919	527.05010	–1.73	12	7.685		▲	3
**M171**	Apigenin glucuronyl sulfate 1	$C_{21}H_{18}O_{14}S$	525.03345	525.03445	–1.90	13	6.634		▲	3
**M172**	Apigenin glucuronyl sulfate 2	$C_{21}H_{18}O_{14}S$	525.03333	525.03445	–2.13	13	6.685		▲	3
**M173**	Apigenin glucuronyl sulfate 3	$C_{21}H_{18}O_{14}S$	525.03351	525.03445	–1.79	13	5.334	▲		3
**M174**	Apigenin glucuronyl sulfate 4	$C_{21}H_{18}O_{14}S$	525.03331	525.03445	–2.17	13	5.442	▲		3
**M175**	Apigenin glucuronyl sulfate 5	$C_{21}H_{18}O_{14}S$	525.03321	525.03445	–2.36	13	5.834	▲		3
**M176**	Apigenin glucuronyl sulfate 6	$C_{21}H_{18}O_{14}S$	525.03356	525.03445	–1.70	13	6.209	▲		3
**M177**	Apigenin glucuronyl sulfate 7	$C_{21}H_{18}O_{14}S$	525.03366	525.03445	–1.50	13	6.900	▲		3
**M192**	Hydroxybenzyl alcohol glucuronyl sulfate	$C_{13}H_{16}O_{11}S$	379.03320	379.03406	–2.27	6	4.096		▲	3

Notec: No.: number; MF: molecular formula; Meas.: measured; Pred.: predicted; Diff: difference; DBE: double bone equivalents; t_R: retention time; P: plasma; U: urine; F: feces; CL: confidence levels; *: potential new compounds by retrieving information from SciFinder database; ▲: detected

2.1 Secondary mass spectrometry (MS^2) data of M10–M12, and M52–M55

A

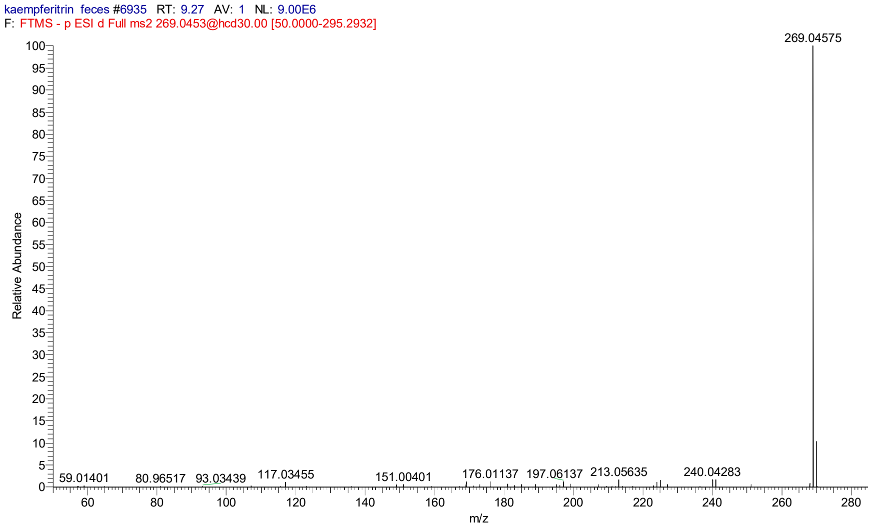

B

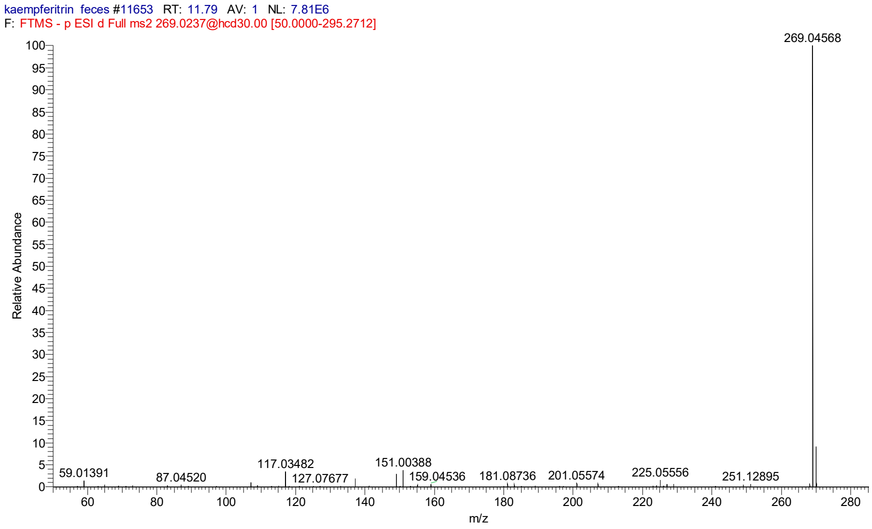

C

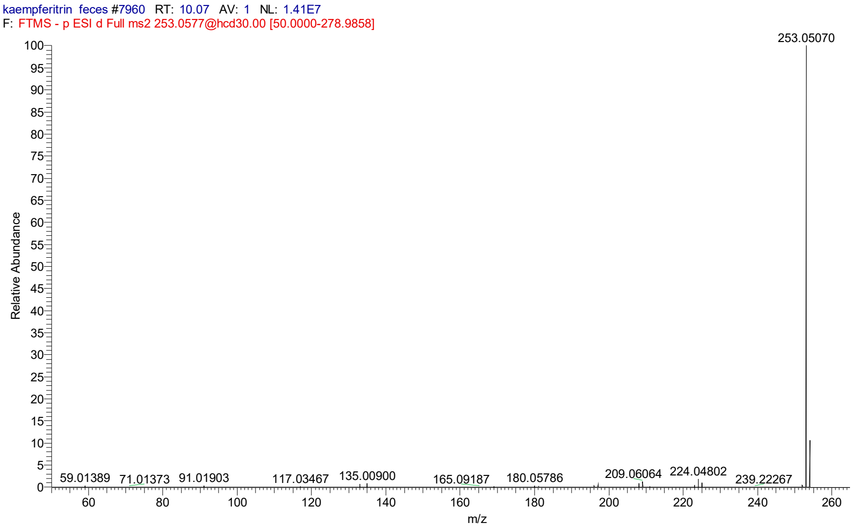

Figure S1 Secondary mass spectrometry (MS^2) data of **M10** (A), **M11** (B), and **M12** (C).

A

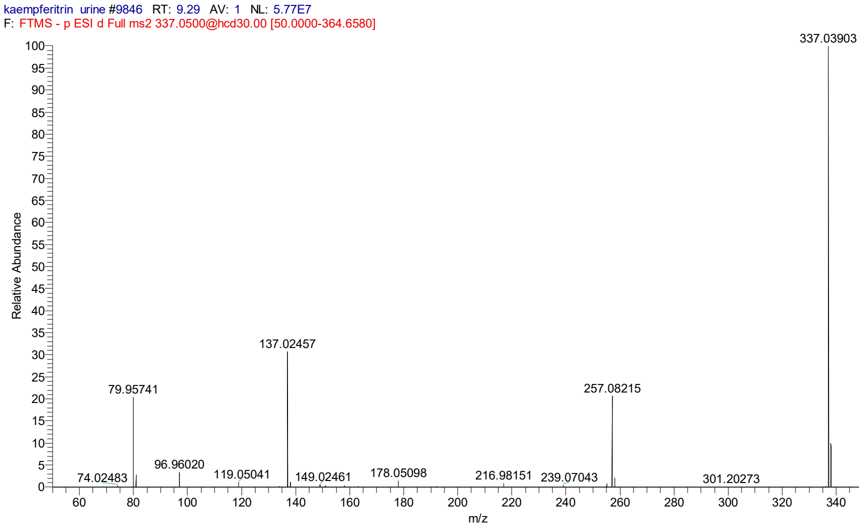
kaempferitrin urine #9846 RT: 9.29 AV: 1 NL: 5.77E7
F: FTMS - p ESI d Full ms2 337.0500@hcd30.00 [50.0000-364.6580]
337.03903
137.02457
79.95741
257.08215
74.02483
96.96020
119.05041
149.02461
178.05098
216.98151
239.07043
301.20273
Relative Abundance
m/z

B

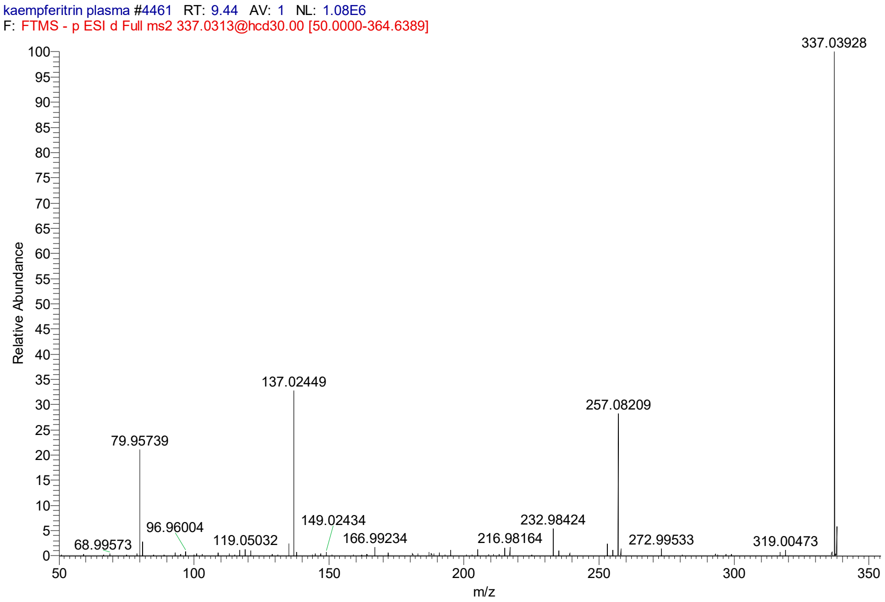
kaempferitrin plasma #4461 RT: 9.44 AV: 1 NL: 1.08E6
F: FTMS - p ESI d Full ms2 337.0313@hcd30.00 [50.0000-364.6389]
337.03928
137.02449
257.08209
79.95739
232.98424
149.02434
96.96004
166.99234
216.98164
68.99573
119.05032
272.99533
319.00473
Relative Abundance
m/z

C

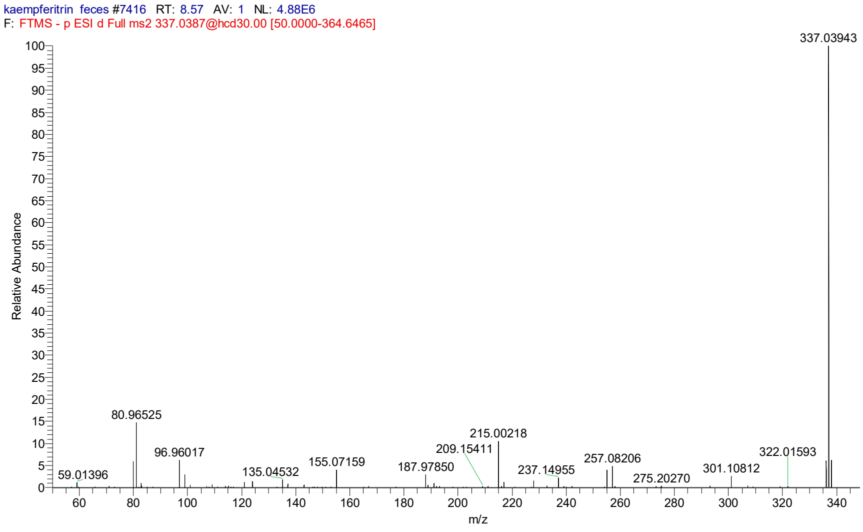
kaempferitrin feces #7416 RT: 8.57 AV: 1 NL: 4.88E6
F: FTMS - p ESI d Full ms2 337.0387@hcd30.00 [50.0000-364.6465]
337.03943
80.96525
215.00218
209.15411
322.01593
96.96017
257.08206
155.07159
187.97850
237.14955
301.10812
135.04532
275.20270
59.01396
Relative Abundance
m/z

D

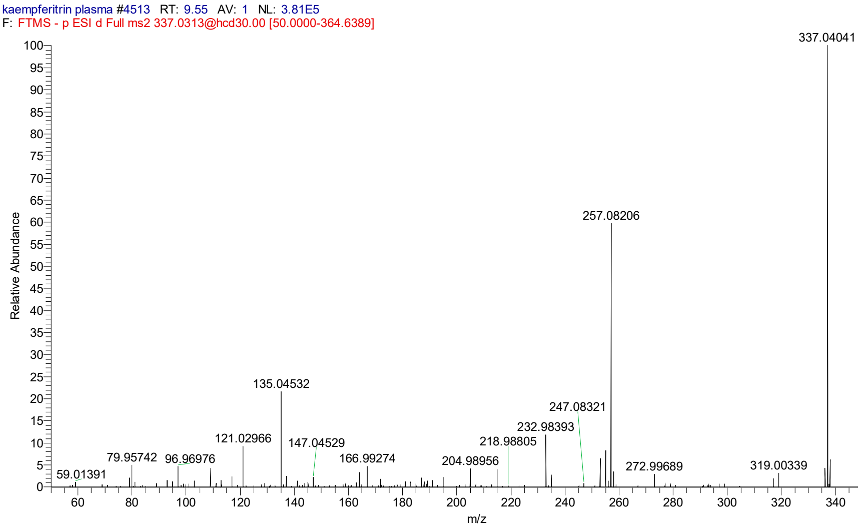

Figure S2 Secondary mass spectrometry (MS^2) data of **M53** (A), **M54** (B), **M52** (C), **and M55** (D).

2.2 Characterization of phase I metabolites (M2–M9 and M18–M25) with Figures S3–10

(1) B-Ring hydroxylated apigenin (M2)

The extracted ion chromatograms (EICs) of **M1** and **M2** are presented in **Figure S3**. **M1** was clearly identified as kaempferol by comparison with the standard reference. It is observed that *m/z* 285.04010 in the MS spectrum, and the fragment ions of *m/z* 257.04498, *m/z* 151.00383, *m/z* 137.02477, *m/z* 117.03368, and *m/z* 229.05029 in the MS^2 spectrum. The molecular formula (MF) of kaempferol was $C_{15}H_{10}O_6$, the molecular weight (MW) was 286 Da, the double bond equivalents (DBE) was 11, and the retention time (t_R) was 12.078 min. **M2** exhibited ($[M–H]^-$) at *m/z* 285.04041, which implies that the MF was $C_{15}H_{10}O_6$, MW was 286 Da, and DBE was 11, compared with kaempferol had no change. The fragment ions of *m/z* 257.04599, *m/z* 149.02453 ($C_8H_5O_3$, $^{0,2}A^-$), *m/z* 133.02971 ($C_8H_5O_2$, $^{0,2}A^-$), *m/z* 177.01892 ($C_9H_5O_4$, loss of B, A^-) can be seen by MS^2. Among them, *m/z* 177.01892 was the fragment obtained after the characteristic loss of the B-ring of apigenin, so the compound was identified as B-ring hydroxylated apigenin.

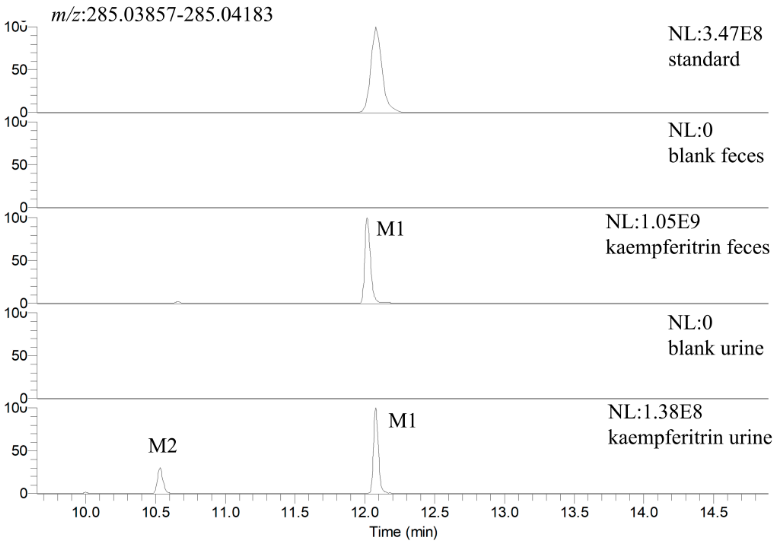

Figure S3 The EICs of **M1** and **M2** in the blank feces, kaempferitrin feces, blank urine, and kaempferitrin urine samples in negative ion mode.

(2) Dehydroxylated and dihydrogenated metabolites of kaempferol (M3–M6)

The EICs of **M3**–**M6** are shown in **Figure S4**. **M3**–**M6** presented $[M–H]^-$ at *m/z* 273.07678, *m/z* 273.07684, *m/z* 273.07693, and *m/z* 273.07648 in the MS spectra, and their MF was predicted to be $C_{15}H_{14}O_5$, their MW was 274 Da, and their DBE was 9. Compared with kaempferol, the MF was supplemented with 4 hydrogen atoms and decreased with 1 oxygen atom, the MW decreased by 12 Da, and the DBE was decreased by 2. Therefore, **M3**–**M6** were tentatively identified as the metabolites of dehydroxylated and dihydrogenated kaempferol.

M6, fragment ions of *m/z* 229.08702, *m/z* 167.03510, *m/z* 151.00365, *m/z* 119.05053, and *m/z* 125.02457 can be seen by MS spectrum. Therefore, **M6** was identified as apiforol. **M3**, fragment ions of *m/z* 255.06639, *m/z* 167.03511, *m/z* 163.04073, *m/z* 149.02466, and *m/z* 109.029676 can be seen by MS^2. **M4**, fragment ions of *m/z* 258.05411, *m/z* 151.04019, *m/z* 125.02410, and *m/z* 163.04041 can be seen by MS^2, and **M5**, fragment ions of *m/z* 255.06644, *m/z* 163.04019, *m/z* 136.01678, *m/z* 151.04007, and *m/z* 109.02961 can be seen by MS^2. Therefore, the compounds were identified as apiforol isomers.

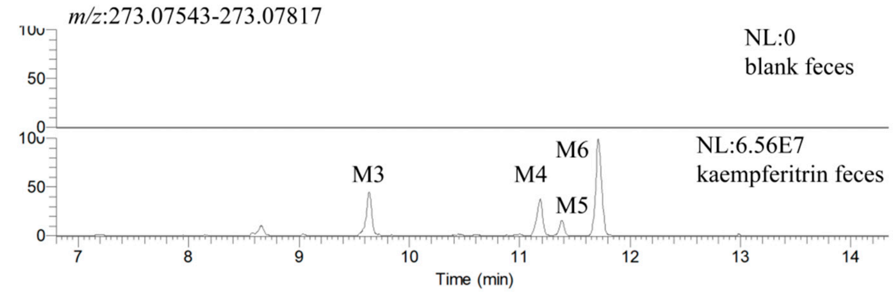

Figure S4 The EICs of **M3**–**M6** in the blank and kaempferitrin feces samples in negative ion mode.

(3) Dehydroxylated and hydrogenated metabolites of kaempferol (M7–M9)

The EICs of **M7**–**M9** are presented in **Figure S5**. In negative ion mode, the **M7**–**M9** presented at *m/z* 271.06107, *m/z* 271.06100, and *m/z* 271.06116 ($[M–H]^-$) in the MS spectra, MF was predicted to be $C_{15}H_{12}O_5$. Compared to kaempferol, MF was increased by 2 hydrogen atoms and decreased with 1 oxygen atom, DBE was 10, it was speculated that the C-ring has a reduction reaction. Therefore, **M7**–**M9** were tentatively identified as the dehydroxylated and hydrogenated metabolites of kaempferol. **M7** fragment ions of *m/z* 271.06145, *m/z* 151.00381 ($C_7H_3O_4$, $^{1,3}A^-$), *m/z* 119.05044 (C_8H_7O,$^{1,3}B^-$), *m/z* 177.01901 ($C_9H_5O_4$, loss B-ring, A^-), *m/z* 107.05025, and *m/z* 93.03465, of which *m/z* 177.01901 was the fragment of dihydrogenated flavone after the characteristic loss of the B-ring, so the compound was identified as naringenin.

The fragment ions of **M8** were 271.06143, *m/z* 165.01964, *m/z* 151.00394, *m/z* 137.02440, *m/z* 121.02946, and *m/z* 93.03452, which could be seen in the MS^2 spectrum. The fragment ions of **M9** were *m/z*271.06143, *m/z* 165.01947, *m/z* 151.00391, *m/z* 137.02463, *m/z* 119.05028, and *m/z* 133.02963, which could be seen in MS^2, identified the compound as naringenin isomer, maybe C-ring opening and C-4 carbonyl reduction.

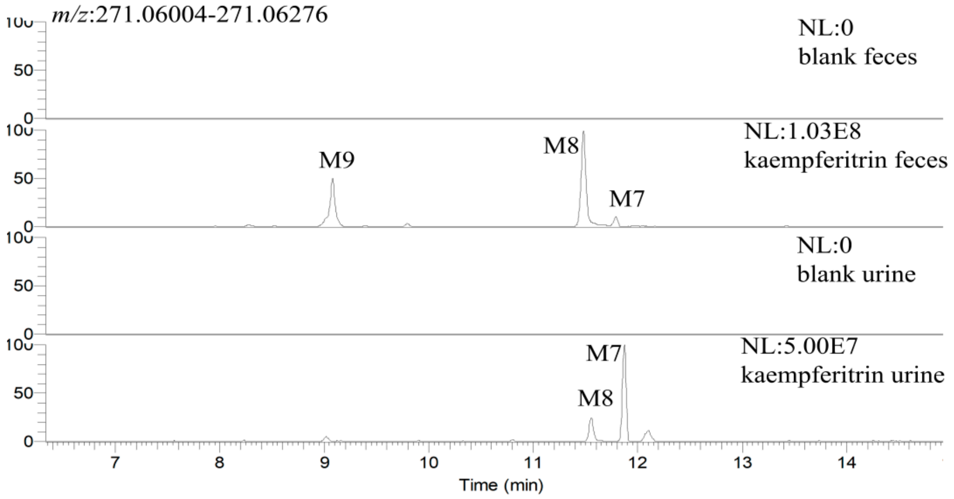

Figure S5 The EICs of **M7–M9** in the blank feces, kaempferitrin feces, blank urine, and kaempferitrin urine samples in negative ion mode.

(4) Hydroxylated metabolites of kaempferol (M18 and M19)

The EICs of **M18** and **M19** are presented in **Figure S6**. In the negative ion mode, the *m/z* 301.03525 and *m/z* 301.03522 ($[M–H]^-$) are presented in their MS spectra, MF was predicted to be $C_{15}H_{10}O_7$. Compared to kaempferol, MF was increased by 2 oxygen atoms, and DBE was 11. Therefore, **M18** and **M19** were tentatively identified as hydroxylated metabolites of kaempferol. The fragment ions of *m/z* 151.00383 ($C_7H_3O_4$, $^{1,3}A^-$) and *m/z* 178.99870 were seen at the MS^2 spectrum of **M18**, and **M18** was tentatively identified as quercetin. **M19** fragment ions of *m/z* 151.00412 ($C_7H_3O_4$, $^{0,3}A^-$) and *m/z* 178.99891 were seen at the MS^2. **M19** was identified as B-ring hydroxylated kaempferol.

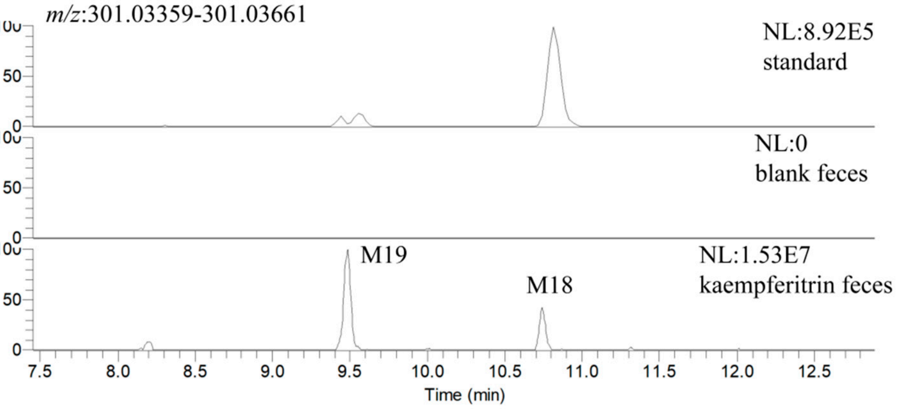

Figure S6 The EICs of **M18** and **M19** in the standard, blank feces, and kaempferitrin feces samples in negative ion mode.

(5) Dihydroxylated and hydrogenated metabolite of kaempferol (M20)

The EICs of **M20** are presented in **Figure S7**. In the negative ion mode, the presented at *m/z* 255.06622 ([M–H]$^-$) in the MS spectra, MF was predicted to be $C_{15}H_{12}O_4$, and DBE was 10. Compared to kaempferol, MF was increased by 2 hydrogen atoms and decreased by 2 oxygen atoms, and it was presumed to be a dihydroxylated and hydrogenated metabolite. The fragment ions of **M20** were *m/z* 149.02461 ($C_8H_5O_3$, $^{0,2}A^-$), *m/z* 135.00896 ($C_7H_3O_3$, $^{0,3}A^-$), and *m/z* 121.02946 ($C_7H_5O_2$, $^{0,2}B^-$), which could be seen by MS^2. Determined that dihydroxylated occurred in the A-ring, so the compound was identified as A-ring dehydroxylated naringenin, maybe 7, 4'-dihydroxylatedxyflavanone or 5, 4'-dihydroxylatedxyflavanone.

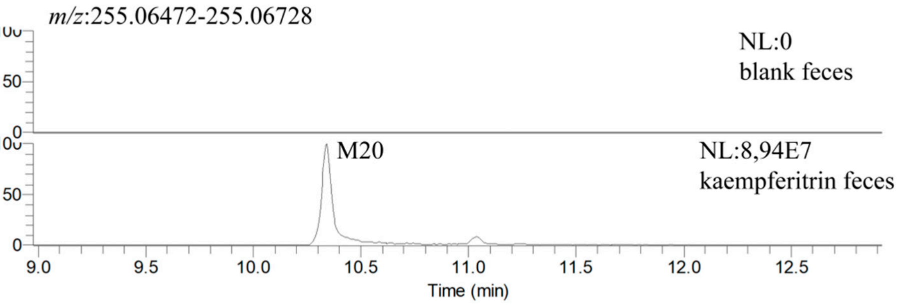

Figure S7 The EICs of **M20** in the blank and kaempferitrin feces samples in negative ion mode.

(6) Hydrogenated metabolites of kaempferol (M21–M23)

The EICs of **M21–M23** are presented in **Figure S8**. In the negative ion mode, they were presented at *m/z* 287.05585, *m/z* 287.05609, and *m/z* 287.05597 ([M–H]$^-$) in the MS spectra, MF was predicted to be $C_{15}H_{12}O_6$. Compared with kaempferol, MF was increased by 2 hydrogen atoms, DBE was 10, and it was speculated that the C-ring had a reduction reaction. **M21** showed the fragment ions of *m/z* 269.04742, *m/z* 243.06665, *m/z* 165.01907 ($C_8H_5O_4$, $^{1,2}A^-$), *m/z* 151.00383 ($C_7H_3O_4$, $^{1,3}A^-$), and *m/z* 125.02464 ($C_6H_5O_3$, $^{1,4}A^-$) could be seen by MS^2, the compound was identified as hydroxylated kaempferol. The fragment ions of **M22** and **M23** were *m/z* 269.0455, *m/z* 243.0666,

and *m/z* 125.0245; the compounds were identified as hydroxylated kaempferol isomers.

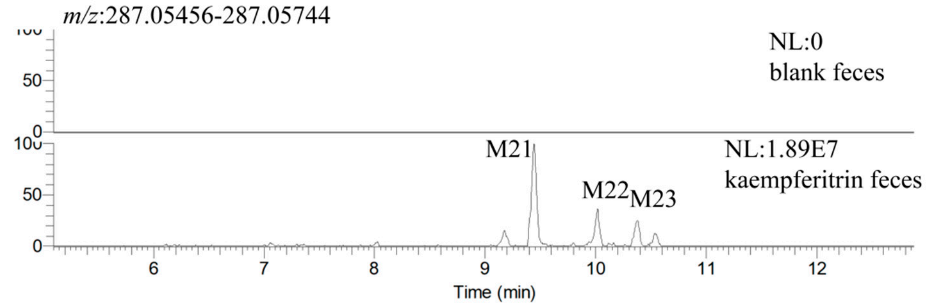

Figure S8 The EICs of **M21–M23** in the blank and kaempferitrin feces samples in negative ion mode.

(7) Dihydrogenated metabolite of kaempferol (M24)

The EICs of **M24** are presented in **Figure S9**. In the negative ion mode, the **M24** presented at *m/z* 289.07156 ($[M–H]^-$) in the MS spectra, MF was predicted to be $C_{15}H_{14}O_6$. Compared with kaempferol, MF was increased by 4 hydrogen atoms, and DBE was 9, it was speculated that the C-ring had undergone a reduction reaction. The fragment ions of *m/z* 271.06137, *m/z* 183.03011, *m/z* 164.01166, and *m/z* 125.02459 can be seen by the MS^2. The compound was identified as dihydrogenated kaempferol.

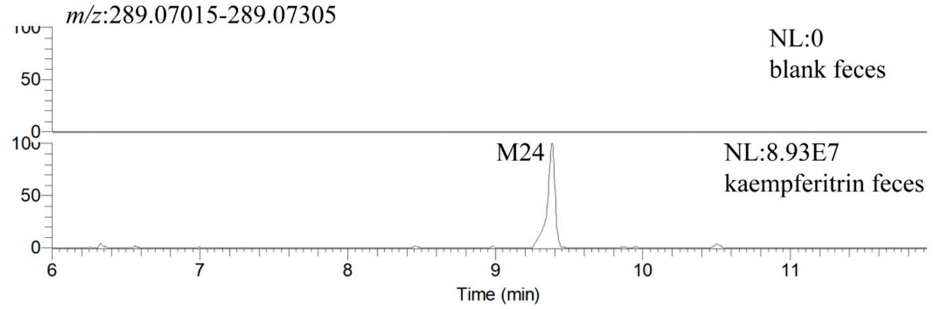

Figure S9 The EICs of **M24** in the blank feces and kaempferitrin feces samples in negative ion mode.

(8) Decarbonylated metabolite of kaempferol (M25)

The EICs of **M25** are presented in **Figure S10**. In the negative ion mode, the peak presented at *m/z* 257.04550 ($[M–H]^-$) in the MS spectra, MF was predicted to be $C_{14}H_{10}O_5$. Compared with kaempferol, MF was decreased by a unit of CO, DBE was 10, and it was presumed to be a

decarbonylated metabolite of kaempferol. The fragment ions of *m/z* 135.04532 ($C_8H_7O_2$, $^{0,2}A^-$), *m/z* 121.02946 ($C_7H_5O_2$, $^{0,2}B^-$), and *m/z* 109.02972 ($C_6H_5O_2$, $^{0,4}A^-$) can be seen by MS^2. The compound was identified as 4, 6-dihydroxylatedxy-2-(4-hydroxyphenyl)-3(2*H*)-benzofuranone.

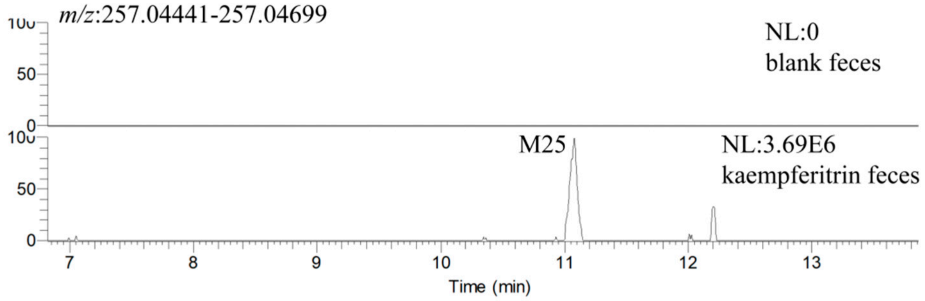

Figure S10 The EICs of **M25** in the blank and kaempferitrin feces samples in negative ion mode.

2.2 Characterization of apigenin glucoses (M26 and M27) with Figure S11

The EICs of **M26** and **M27** are presented in **Figure S11**. In the negative ion mode, the **M26** and **M27** presented at *m/z* 477.10318 and *m/z* 477.10303 ($[M+HCOO]^-$) in the MS spectra, MF was predicted to be $C_{22}H_{22}O_{12}$. The fragment ions of *m/z* 431.097, *m/z* 269.045, and *m/z* 255.008 can be seen by MS^2, in which the relative molecular mass difference of m*/z* 431.097 and *m/z* 269.045 was 162.052, precisely calculated as glucose. The compounds were identified as apigenin glucoses.

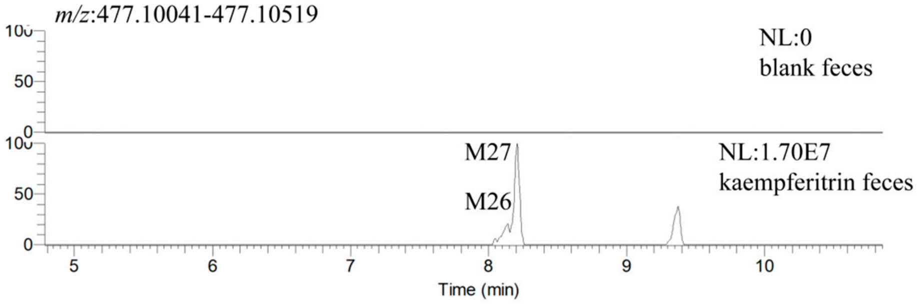

Figure S11 The EICs of **M26** and **M27** in the blank and kaempferitrin feces samples in negative ion mode.

2.3 Characterization of phase II metabolites: hydroxylated and methylated metabolites (M28–M35) with Figures S12–S18

(1) Methylated metabolites of kaempferol (M28 and M29)

The EICs of **M28** and **M29** are presented in **Figure S12**. In the negative ion mode, they are presented at *m/z* 299.05569 and *m/z* 299.05621 ($[M–H]^-$) in the MS spectra. MF was predicted to be $C_{16}H_{12}O_6$, compared with kaempferol, MF was increased by a unit of CH_2, and DBE was unchanged, and it was presumed to be a methylated metabolite of kaempferol. **M29** fragment ions of *m/z* 253.09296, *m/z* 211.07646, *m/z* 179.03522, *m/z* 135.04536 ($C_8H_7O_2$, $^{0,2}A^-$), *m/z* 117.03475 (C_8H_5O, $^{1,3}B^-$). The relative molecular mass difference between *m/z* 299.05621 and *m/z* 284.03302 was calculated as methylation. **M28** fragment ions of *m/z* 284.03302 and *m/z* 123.04543 can be seen by MS^2. Presumably, methylation occurs at the A-ring. On the basis of CLogP, **M29** was identified as 7-*O*-methylated kaempferol (CLogP: 2.74425), and **M28** was identified as 5-*O*-methylated kaempferol (CLogP: 1.84425).

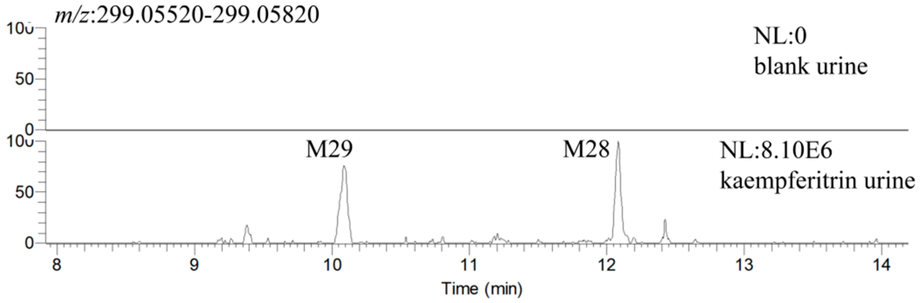

Figure S12 The EICs of **M28** and **M29** in the blank and kaempferitrin urine samples in negative ion mode.

(2) Methylated and dihydroxylated metabolite of kaempferol (M30)

The EICs of **M30** are presented in **Figure S13**. In the negative ion mode, the **M30** presented at *m/z* 301.07138 ($[M–H]^-$) in the MS spectra, MF was predicted to be $C_{16}H_{14}O_6$, and DBE was 10. Compared with kaempferol, a unit of CH_4 was increased in MF, and DBE was decreased by 1. It was speculated that the C-ring reduction and methylation reaction occurred. The fragment ions of *m/z* 257.08170, *m/z* 283.06180, *m/z* 211.07657, *m/z* 119.05037 (C_8H_7O, $^{1,3}B^-$), *m/z* 152.01123 ($C_7H_4O_4$•, $^{1,3}A^-$), *m/z* 137.02464 ($C_7H_5O_3$, $^{0,3}A^-$), *m/z* 181.05051, *m/z* 109.02967 ($C_6H_5O_2$, $^{0,4}A^-$), *m/z* 107.05038 (C_7H_7O, $^{1,2}B^-$) and *m/z* 93.03463 could be seen by MS^2, methylation was presumed to occur in the B-ring, and the compound was identified as 4'-*O*- methylated hydrogenated kaempferol.

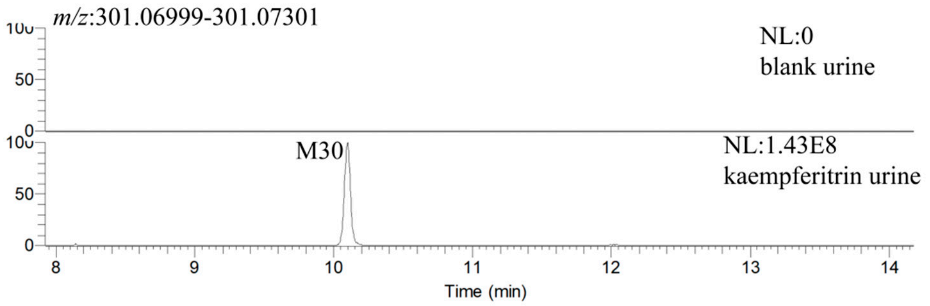

Figure S13 The EICs of **M30** in the blank and kaempferitrin urine samples in negative ion mode.

(3) Dimethylated and hydroxylated metabolite of kaempferol (M31)

The EICs of **M31** are presented in **Figure S14**. In the negative ion mode, the **M31** presented at *m/z* 329.06644 ($[M–H]^–$) in the MS spectra, and MF was predicted to be $C_{17}H_{14}O_7$, compared with kaempferol, a unit of C_2H_4O was increased in MF, and DBE was unchanged. It was speculated that hydroxylation and dimethylation reactions occurred. The fragment ions of *m/z* 314.04355, *m/z* 299.01990, *m/z* 285.04111, *m/z* 271.02460, and *m/z* 201.09520 can be seen by the MS^2. The compound was identified as dimethylated quercetin.

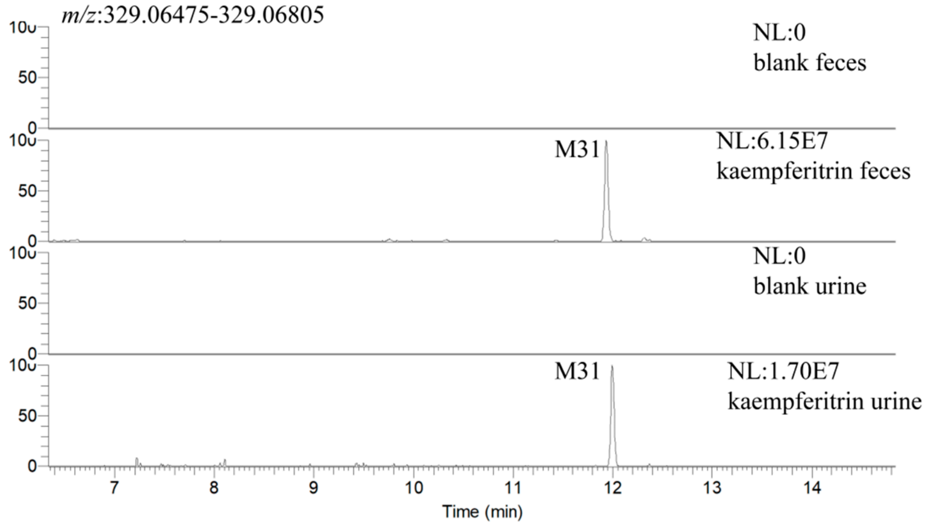

Figure S14 The EICs of **M31** in the blank feces, kaempferitrin feces, blank urine, and kaempferitrin urine samples in negative ion mode.

(4) Methylated and hydroxylated metabolite of kaempferol (M32)

The EICs of **M32** are presented in **Figure S15**. In the negative ion mode, the **M32** presented at *m/z* 315.05099 ($[M–H]^–$) in the MS spectra, MF was predicted to be $C_{16}H_{12}O_7$, compared with

kaempferol, a unit of CH_2O was increased in MF, and DBE was unchanged. It was speculated that hydroxylation and methylation reactions occurred. The fragment ions of *m/z* 300.02780, *m/z* 149.06100 ($C_9H_9O_2$, $^{0,2}A^-$), *m/z* 119.05023 (C_8H_7O, $^{1,3}B^-$) can be seen by the MS^2. Based on the structure of **M32**, methylation was assumed to occur in the A-ring. The compound was identified as A-ring methylated quercetin.

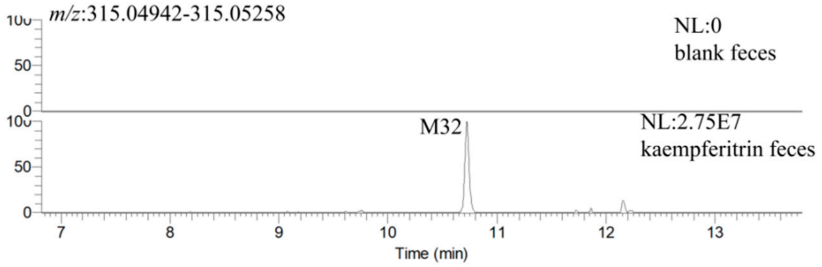

Figure S15 The EICs of **M32** in the blank and kaempferitrin feces samples in negative ion mode.

(5) Methylated, hydroxylated, and hydrogenated metabolite of kaempferol (M33)

The EICs of **M33** are presented in **Figure S16**. In the negative ion mode, the **M33** presented at *m/z* 317.06662 ($[M–H]^-$) in the MS spectra, MF was predicted to be $C_{16}H_{14}O_7$, compared with kaempferol, a unit of CH_4O was increased in MF, and DBE was decreased by 1. It was speculated that hydroxylation, methylation, and hydrogenation reactions occurred. The fragment ions of *m/z* 299.05640, *m/z* 151.00362, *m/z* 149.02457 ($C_8H_5O_3$, $^{0,2}A^-$), *m/z* 193.01424, *m/z* 167.03516, and *m/z* 119.05023 (C_8H_7O, $^{1,3}B^-$) can be seen by MS^2. *m/z* 193.01412 speculated that methylation occurred in the A-ring, and identified the compound as A-ring methylated hydrogenated quercetin.

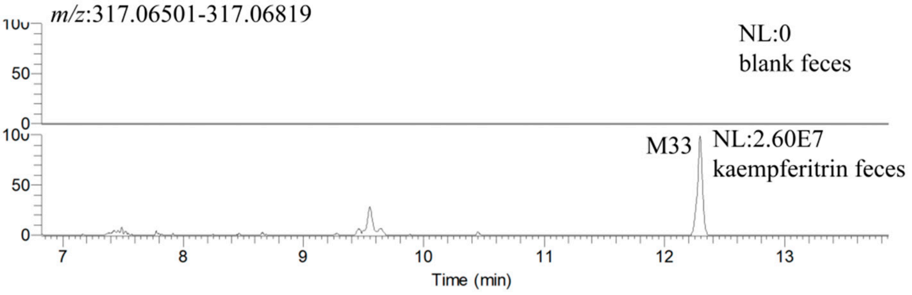

Figure S16 The EICs of **M33** in the blank and kaempferitrin feces samples in negative ion mode.

(6) Dihydroxylated methylated and hydrogenated metabolite of kaempferol (M34)

The EICs of **M34** are presented in **Figure S17**. In the negative ion mode, the **M34** presented at *m/z* 333.06152 ($[M–H]^-$) in the MS spectra, MF was predicted to be $C_{16}H_{14}O_8$, compared with kaempferol, a unit of CH_4O_2 was increased in MF, and DBE was decreased by 1. It was speculated that dihydroxylation, methylation, and hydrogenation reactions occurred. The fragment ions of *m/z* 315.05151, *m/z* 297.04099, *m/z* 271.06100, *m/z* 151.00391 ($C_7H_3O_4$, $^{1,3}A^-$), *m/z* 121.02969 ($C_7H_5O_2$, $^{0,2}B^-$), *m/z* 169.01433, and *m/z* 165.01949 could be seen by MS^2. *m/z* 169.01433 and *m/z* 165.01949, speculated that methylation and hydroxylation occurred in the A-ring. The compound was identified as A-ring methylated hydroxylated hydrogenated quercetin.

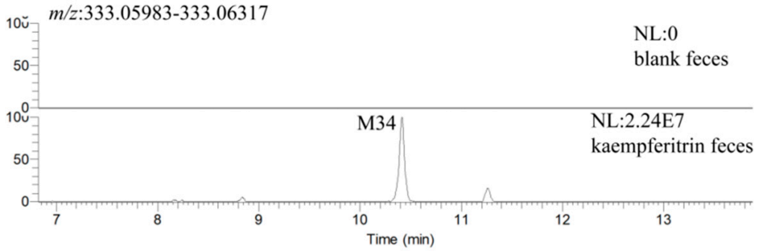

Figure S17 The EICs of **M34** in the blank and kaempferitrin feces samples in negative ion mode.

(7) Methylated and dehydroxylated metabolite of kaempferol (M35)

The EICs of **M35** are presented in **Figure S18**. In the negative ion mode, the **M35** presented at *m/z* 283.06100 ($[M–H]^-$) in the MS spectra, MF was predicted to be $C_{16}H_{12}O_5$, DBE was 11, compared with kaempferol, MF was reduced by 1 oxygen atom and increased by a unit of CH_2, DBE was unchanged. It was speculated that dehydroxylation and methylation occurred. The fragment ions of *m/z* 268.03799, *m/z* 240.04306 (–CO), *m/z* 211.03999, *m/z* 148.01707, *m/z* 135.00891 ($C_7H_3O_3$, $^{0,3}A^-$), and *m/z* 107.05064 (C_7H_7O, $^{1,2}B^-$) could be seen by MS^2. Speculated that methylation occurred in the A-ring. The compound was identified as A-ring methylated apigenin.

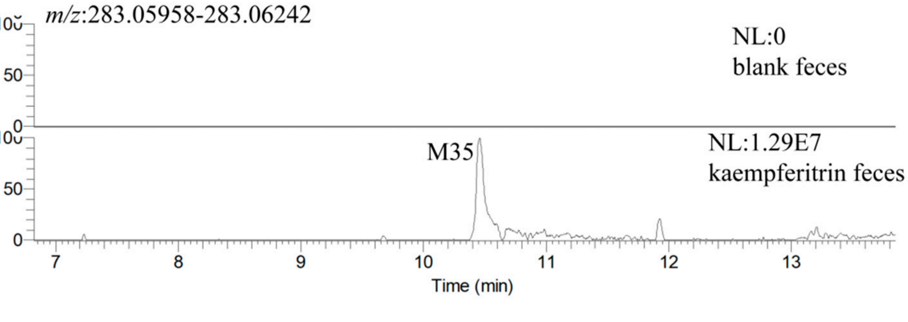

Figure S18 The EICs of **M35** in the blank and kaempferitrin feces samples in negative ion mode.

3.4. Characterization of phase II metabolites: sulfated metabolites (M36–M49, and M57–M92) with Figures S19-35

(1) Methylated apigenin sulfates (M36–M38)

The EICs of **M36**–**M38** are presented in **Figure S19**. In the negative ion mode, the presented at *m/z* 363.01724, *m/z* 363.01685, and *m/z* 363.01758 ($[M–H]^-$) in the MS spectra, MF was predicted to be $C_{16}H_{12}O_8S$. **M36** showed the fragment ions of *m/z* 283.06143, *m/z* 268.03796, *m/z* 201.02208, and *m/z* 113.02456 can be seen by MS^2. Among them, *m/z* 79.95730 was the characteristic fragment ion of sulfation, and *m/z* 201.02208 suppositions that sulfation occurs in the B-ring. The compound was identified as A-ring methylated apigenin-4'-*O*-sulfate. In the negative ion mode, the **M37** and **M38** fragment ions of *m/z* 283.0613, *m/z* 214.9939, and *m/z* 268.0379 can be seen in MS^2. The difference in molecular weight between *m/z* 363.0171 and *m/z* 283.0613 was 79.9558, indicating that the compounds were sulfates. **M37** and **M38** were identified as methylated apigenin sulfates.

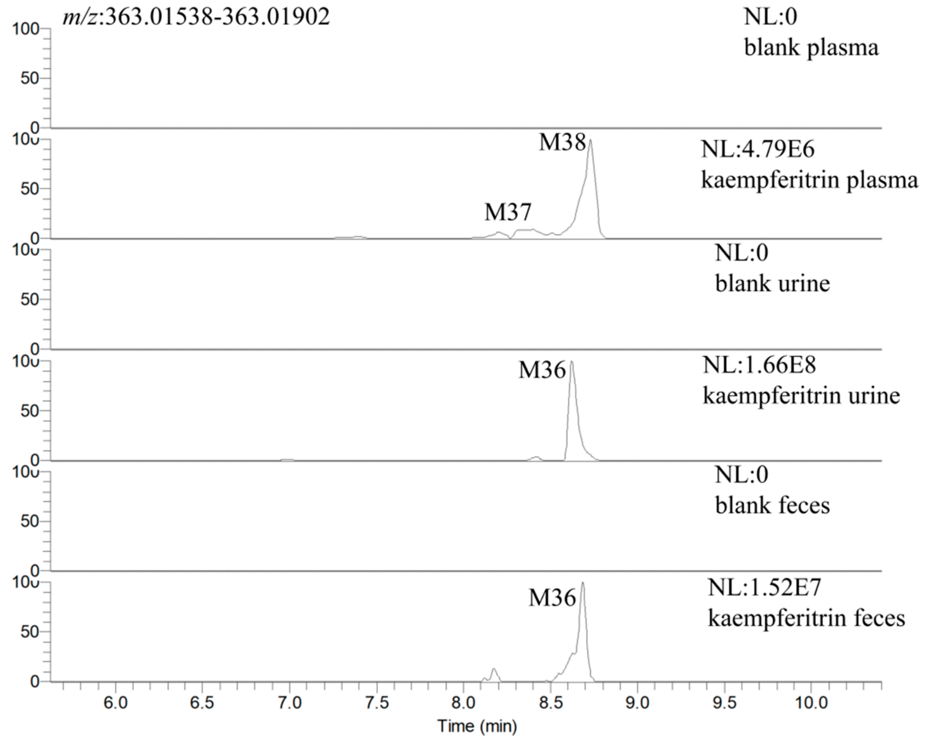

Figure S19 The EICs of **M36–M38** in the blank plasma, kaempferitrin plasma, blank urine, kaempferitrin urine, blank feces, and kaempferitrin feces samples in negative ion mode.

(2) Kaempferol sulfates (M39 and M40)

The EICs of **M39** and **M40** are presented in **Figure S20**. In the negative ion mode, the presented at *m/z* 364.99634 and *m/z* 364.99603 ($[M–H]^-$) in the MS spectra, and MF was predicted to be $C_{15}H_{10}O_9S$. The fragment ions of *m/z* 285.0407, *m/z* 257.0452, *m/z* 151.0038 ($C_7H_3O_4$, $^{1,3}A^-$), and *m/z* 96.9602 can be seen by MS^2, in which *m/z* 96.9602 was the characteristic fragment ion of sulfation. The compounds were identified as kaempferol sulfates.

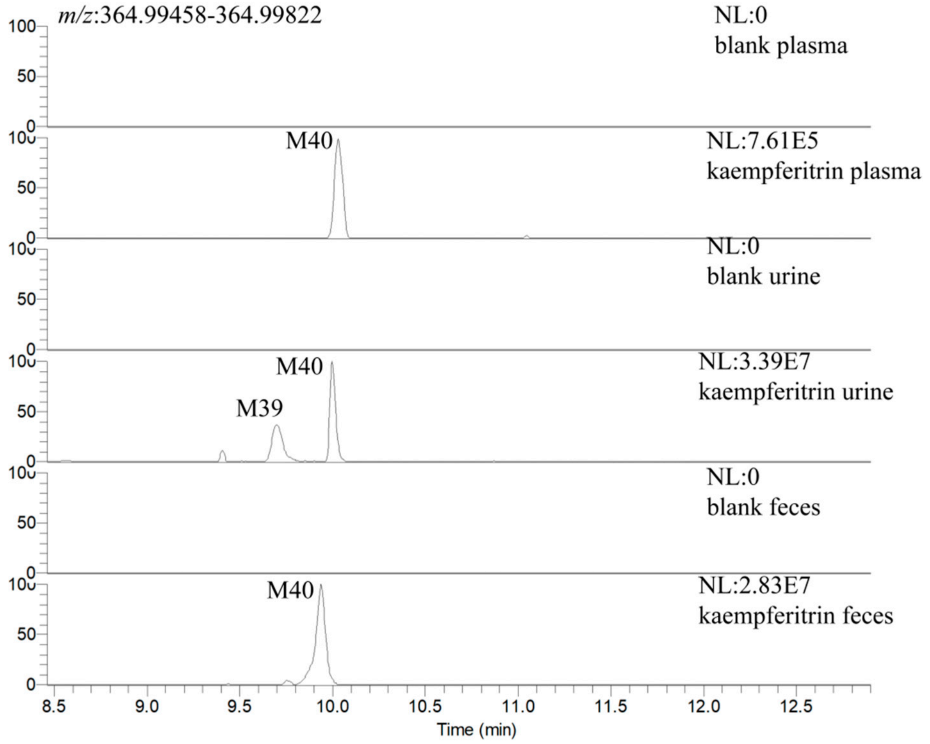

Figure S20 The EICs of **M39** and **M40** in the blank plasma, kaempferitrin plasma, blank urine, kaempferitrin urine, blank feces, and kaempferitrin feces samples in negative ion mode.

(3) Naringenin sulfates (M41–M44)

The EICs of **M41**–**M44** are presented in **Figure S21**. In the negative ion mode, the presented at *m/z* 351.01761, *m/z* 351.01709, *m/z* 351.01727, and *m/z* 351.01764 ($[M–H]^-$) in the MS spectra, and MF was predicted to be $C_{15}H_{12}O_8S$. **M41** and **M44** fragment ions of *m/z* 271.0615, *m/z* 151.0038 ($C_7H_3O_4$, $^{1,3}A^-$), *m/z* 165.0195 ($C_8H_5O_4$, $^{1,2}A^-$), *m/z* 121.0296 ($C_7H_5O_2$, $^{0,2}B^-$), and *m/z* 93.0343, in which *m/z* 79.9574 was a characteristic fragment ion of sulfation, comparison based on retention time and CLogP, **M44** was identified as naringenin-7-*O*-sulfate (CLogP: 0.821621), and **M41** was

identified as naringenin-5-*O*-sulfate (CLogP: 0.361621).

In the MS^2 spectrum of **M43**, the fragment ions of *m/z* 271.06152, *m/z* 139.04021 ($C_7H_7O_3$, $^{0,3}A^-$), *m/z* 151.00385 ($C_7H_3O_4$, $^{1,3}A^-$), *m/z* 119.05037 (C_8H_7O, $^{1,3}B^-$), *m/z* 93.03459, and *m/z*199.00745 (*m/z* 119.05037+SO_3) were observed, Among these fragment ions, the *m/z* 79.95744 was the characteristic fragment ion of sulfation, and the compound was identified as naringenin-4'-*O*-sulfate. In the MS^2 spectrum of **M42**, the fragment ions of *m/z* 271.06152, *m/z* 139.04021 ($C_7H_7O_3$, $^{0,3}A^-$), *m/z* 151.00385 ($C_7H_3O_4$, $^{1,3}A^-$), *m/z* 119.05037, *m/z* 183.03020, *m/z* 96.96024, and *m/z* 79.95744 were observed, in which *m/z* 96.96024, and *m/z* 79.95744 were the characteristic fragment ion of sulfation, and the compound was identified as naringenin isomer sulfate.

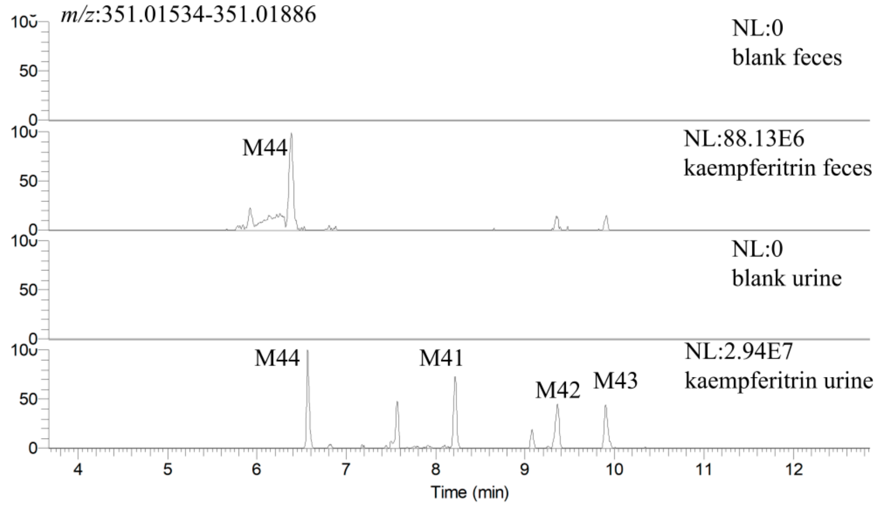

Figure S21 The EICs of **M41–M44** in the blank feces, kaempferitrin feces, blank urine, and kaempferitrin urine samples in negative ion mode.

(4) Apigenin sulfates (M45–M49)

The EICs of **M45–M49** are presented in **Figure S22**. In the negative ion mode, the presented at *m/z* 349.00110, *m/z* 349.00116, *m/z* 349.00137, *m/z* 349.00112 and *m/z* 349.00121 ($[M–H]^-$) in the MS spectra, and MF was predicted to be $C_{15}H_{10}O_8S$. The fragment ions of *m/z* 269.0460, *m/z* 225.0547, *m/z* 151.0037 ($C_7H_3O_4$, $^{1,3}A^-$), *m/z* 117.0351(C_8H_5O, $^{1,3}B^-$), *m/z* 96.9601, and *m/z* 79.9569 could be seen by MS^2, in which *m/z* 96.9601 and *m/z* 79.9569 were characteristic fragment ions of

sulfation. The compounds were identified as apigenin sulfates.

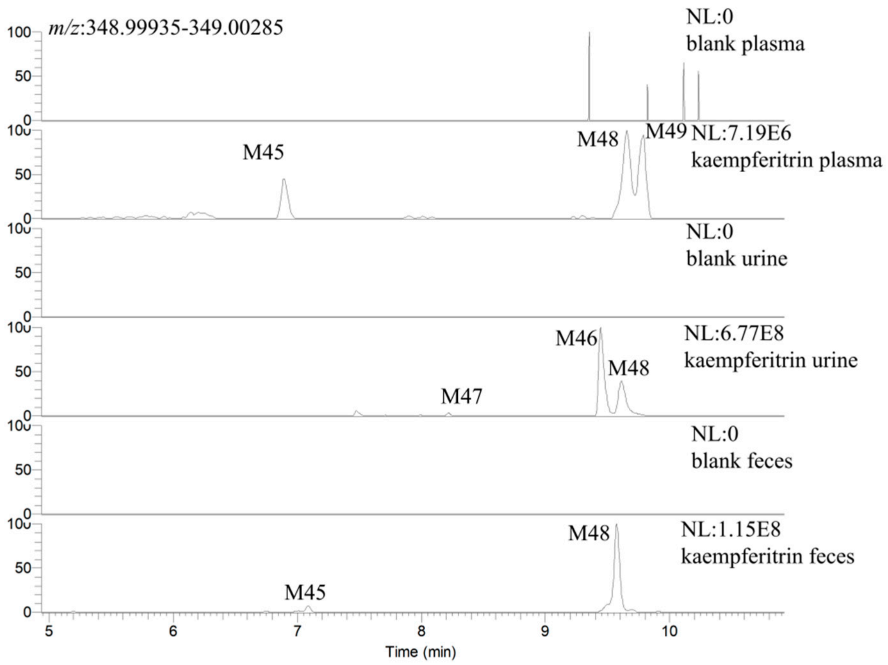

Figure S22 The EICs of **M45**–**M49** in the blank plasma, kaempferitrin plasma, blank urine, kaempferitrin urine, blank feces, and kaempferitrin feces samples in negative ion mode.

(5) Dihydroxylated apigenin sulfates (M57–M61)

The EICs of **M57**–**M61** are presented in **Figure S23**. In the negative ion mode, the presented at *m/z* 335.02246, *m/z* 335.02274, *m/z* 335.02271, *m/z* 335.02298, and *m/z* 335.02287 ($[M–H]^-$) in the MS spectra, MF was predicted to be $C_{15}H_{12}O_7S$. In the MS^2 spectrum of **M58**, the fragment ions of *m/z* 255.06572, *m/z* 149.02465 ($C_8H_5O_3$, $^{0,2}A^-$), *m/z* 162.03382 (B ring loss of dihydroxylated flavone), *m/z* 96.96963, *m/z*79.95904, *m/z* 134.03766, and *m/z* 199.00712(*m/z* 199.05025+SO_3) were observed. Among these fragment ions, the *m/z* 79.95904 was the sulfation characteristic fragment, *m/z* 199.00712 hypothesized that sulfation occurred in the B-ring and identified the compound as dihydroxylated flavanones-4'-*O*-sulfate.

In the negative ion mode, In the MS^2 spectrum of **M57** and **M59**–**M61**, the fragment ions of *m/z* 255.0664, *m/z* 149.0246 ($C_8H_5O_3$, $^{0,2}A^-$), *m/z* 96.9696 and *m/z* 79.9590 were observed, in which *m/z* 79.9590 was the characteristic fragment of sulfation. The compounds were identified as

dihydroxylated flavanone sulfates.

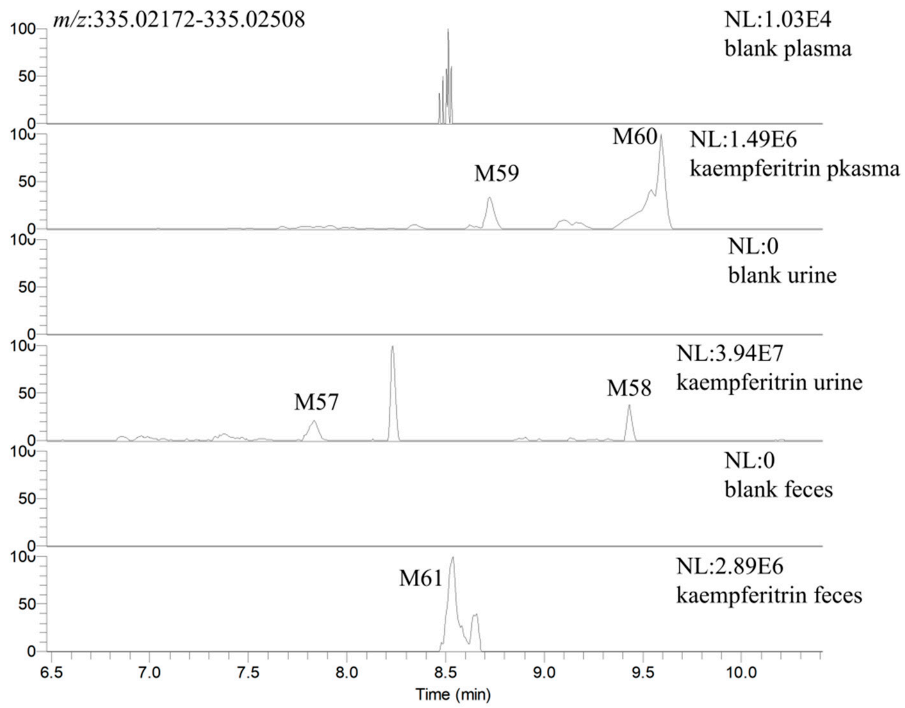

Figure S23 The EICs of **M57–M61** in the blank plasma, kaempferitrin plasma, blank urine, kaempferitrin urine, blank feces, and kaempferitrin feces samples in negative ion mode.

(6) Dehydroxylated apigenin sulfates (M62–M66)

The EICs of **M62–M66** are presented in **Figure S24**. In the negative ion mode, the presented at *m/z* 333.00708, *m/z* 333.00770, *m/z* 333.00772, *m/z* 333.00721, and *m/z* 333.00699 ($[M–H]^–$) in the MS spectra, and MF was predicted to be $C_{15}H_{10}O_7S$. The fragment ions of *m/z* 253.0507, *m/z* 135.0087 ($C_7H_3O_3$, $^{0,3}A^–$), *m/z* 117.0346 (C_8H_5O, $^{1,3}B^–$) and *m/z* 79.9575 could be seen by MS^2, and *m/z* 79.9575 was characteristic fragment ion of sulfation, and the compounds were identified as A-ring dehydroxylated apigenin sulfates.

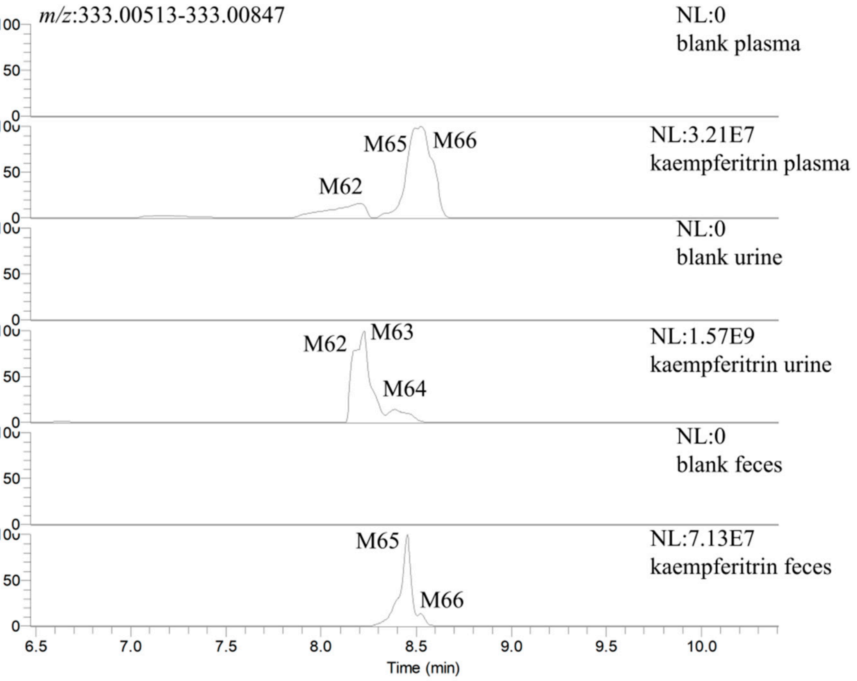

Figure S24. The EICs of **M62–M66** in the blank plasma, kaempferitrin plasma, blank urine, kaempferitrin urine, blank feces, and kaempferitrin feces samples in negative ion mode.

(7) Dihydroxylated flavane sulfates (M67–M69)

The EICs of **M67–M69** are presented in **Figure S25**. In the negative ion mode, the presented at *m/z* 321.04330, *m/z* 321.04337, and *m/z* 321.04345 ($[M–H]^-$) in the MS spectra, MF was predicted to be $C_{15}H_{14}O_6S$, and the MS^2 showed the fragment ions of *m/z* 241.0871, *m/z* 135.0453 ($C_8H_7O_2$, $^{0,2}A^-$), *m/z* 121.0296 ($C_7H_5O_2$, $^{0,2}A^-$), *m/z* 119.0504 (C_8H_7O, $^{1,3}B^-$), *m/z* 93.0347, and *m/z* 79.9574, of which *m/z* 79.9574 was the characteristic fragment ion of sulfation. The compounds were identified as dihydroxylated flavane sulfates. Entering $C_{15}H_{14}O_6S$ into Scifinder retrieved 234 compounds with the same formula, and the presumed compounds were not found and were presumed to be new compounds.

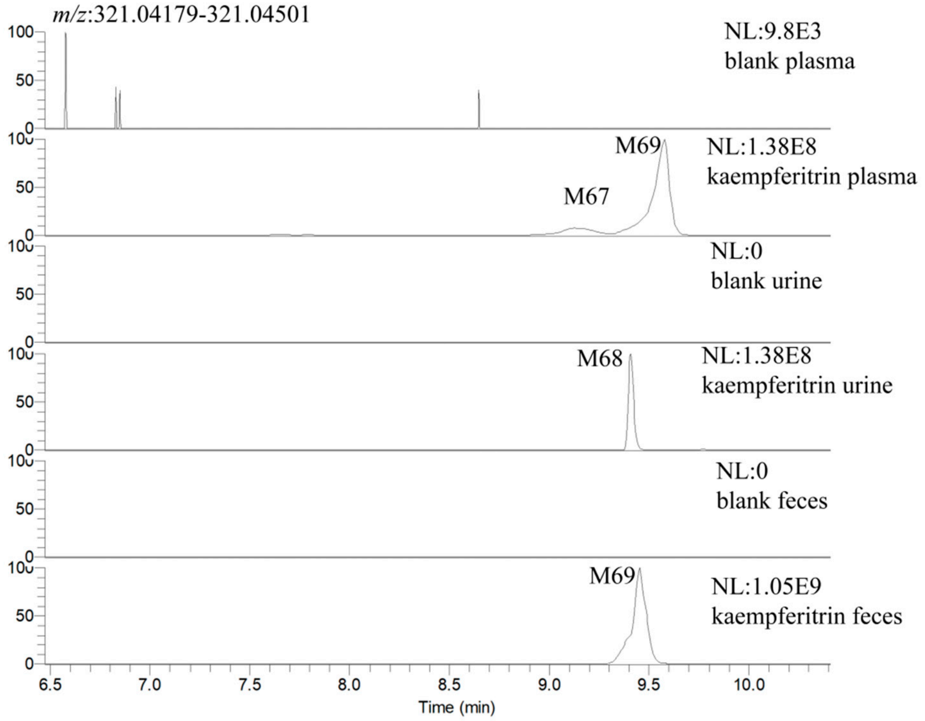

Figure S25 The EICs of **M67–M69** in the blank plasma, kaempferitrin plasma, blank urine, kaempferitrin urine, blank feces, and kaempferitrin feces samples in negative ion mode.

(8) Dimethylated kaempferol sulfate (M70)

The EICs of **M70** are presented in **Figure S26**. In the negative ion mode, the presented at *m/z* 393.02817 ([M–H]$^-$) in the MS spectra, MF was predicted to be $C_{17}H_{14}O_9S$, and the MS^2 visualized the fragment ions of *m/z* 313.07230, *m/z* 298.04868, *m/z* 189.05608, *m/z* 165.05586 ($C_9H_9O_3$, $^{0,2}A^-$), *m/z* 123.04538 ($C_7H_7O_2$, $^{0,3}A^-$), and *m/z* 147.04552, of which the difference in the relative molecular mass between *m/z* 393.02817 and *m/z* 313.07230 was 79.95587, compared with kaempferol, the MF increased a unit of C_2H_4, DBE did not change, it was presumed that dimethylation occurs, the compound was identified as dimethylated kaempferol sulfate.

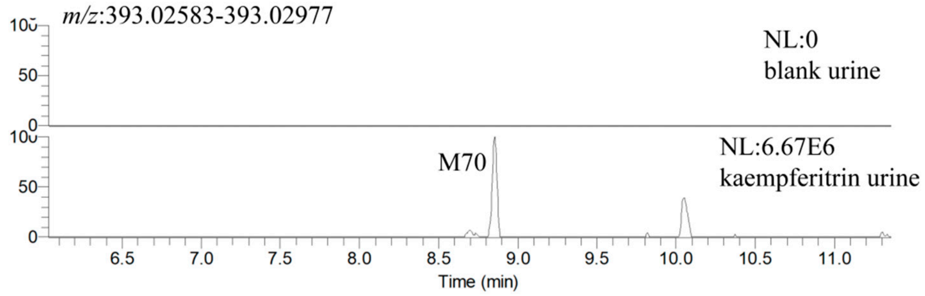

Figure S26 The EICs of **M70** in the blank and kaempferitrin urine samples in negative ion mode.

(9) Hydrogenated kaempferol sulfate (M71)

The EICs of **M71** are presented in **Figure S27**. In the negative ion mode, the presented at *m/z* 367.01160 ($[M–H]^-$) in the MS spectra, MF was predicted to be $C_{15}H_{12}O_9S$, and the MS^2 showed the fragment ions of *m/z* 287.05643, *m/z* 259.06140, *m/z* 243.06654, *m/z* 139.04019 ($C_7H_7O_3$, $^{0,3}A^-$), *m/z* 165.01939 ($C_8H_5O_4$, $^{1,2}A^-$), *m/z* 152.01144 ($C_7H_4O_4$•, $^{1,3}A^-$), *m/z* 125.02457 ($C_6H_5O_3$, $^{1,4}A^-$), *m/z* 96.96022, and *m/z* 79.95739, of which *m/z* 96.96022, *m/z* 79.95739 were characteristic fragment ions of sulfation, which were presumed to be metabolites of sulfation, compared with kaempferol, MF increased by 2 hydrogen atoms, DBE decreased by 1, it was presumed that C-ring reduction occurs, and the compound was identified as hydrogenated kaempferol sulfate.

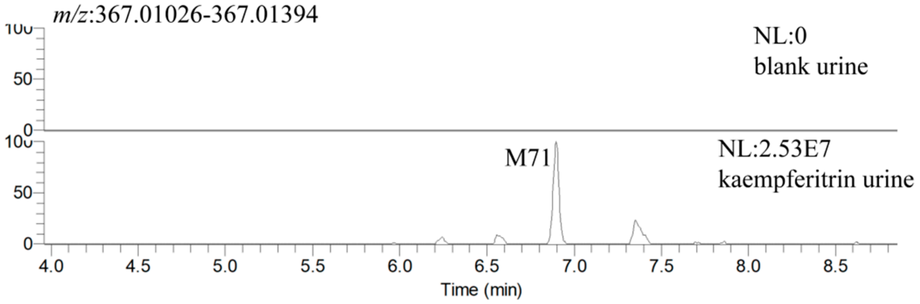

Figure S27 The EICs of **M71** in the blank and kaempferitrin urine samples in negative ion mode.

(10) Methylated dihydrogenated kaempferol sulfate (M72)

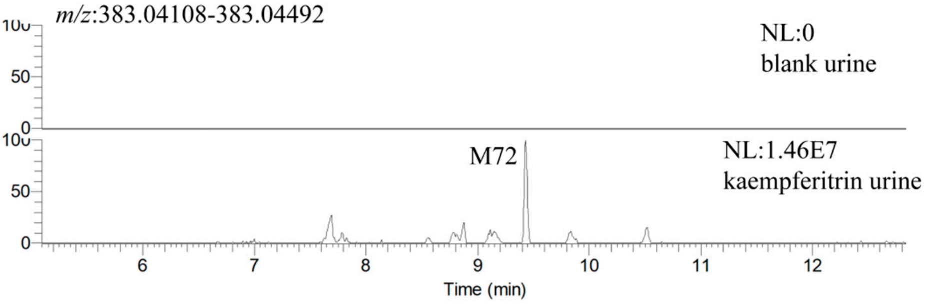

Figure S28 The EICs of **M72** in the blank and kaempferitrin urine samples in negative ion mode.

(11) Methylated dihydrogenated apigenin sulfates (M73–M75)

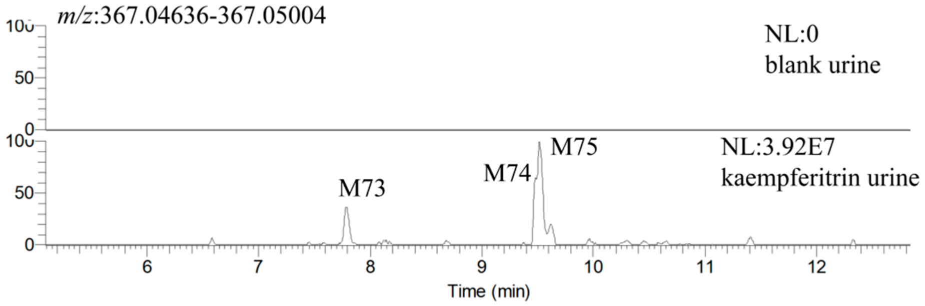

Figure S29 The EICs of **M73–M75** in the blank and kaempferitrin urine samples in negative ion mode.

(12) A, C-Rings of apigenin cracking sulfates (M76–M79)

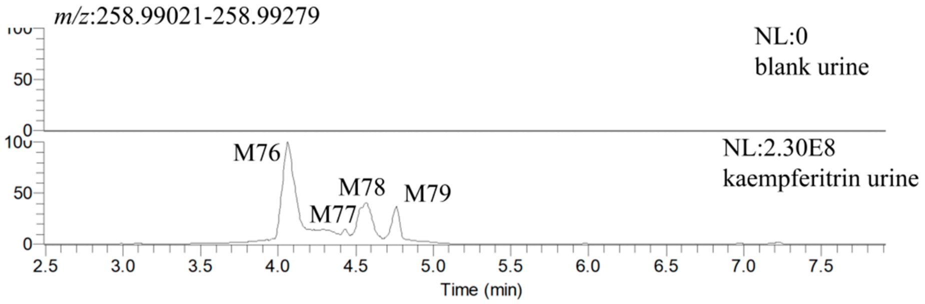

Figure S30 The EICs of **M76–M79** in the blank and kaempferitrin urine samples in negative ion mode.

(13) A, B-Rings of apigenin disulfates (M80 and M81)

The EICs of **M80** and **M81** are presented in **Figure S31**. In the negative ion mode, the presented at *m/z* 428.95834 and *m/z* 428.95844 ($[M–H]^-$) in the MS spectra, MF was predicted to be $C_{15}H_{10}O_{11}S_2$, and the MS^2 showed the fragment ions of *m/z* 349.0025, *m/z* 269.0458, *m/z* 253.0505, *m/z* 228.0100, *m/z* 173.9976, *m/z* 79.9574, and *m/z* 96.9602, of which *m/z* 79.9574 and *m/z* 96.9602 were the characteristic fragment ions for sulfation, and *m/z* 173.9976 was presumed to be sulfated in the B-ring, so the compounds were identified as the A, B-rings of apigenin disulfates.

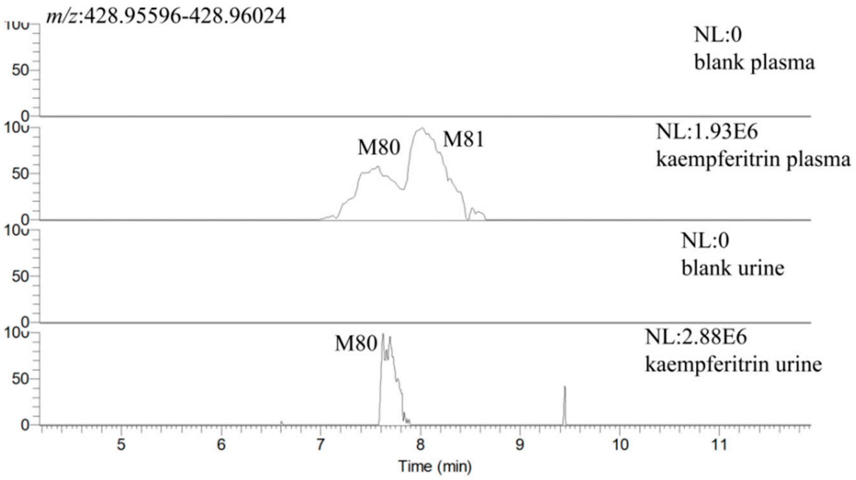

Figure S31 The EICs of **M80** and **M81** in the blank plasma, kaempferitrin plasma, blank urine, and kaempferitrin urine samples in negative ion mode.

(14) Trihydroxylated dihydrogenated flavone disulfates (M82–M84)

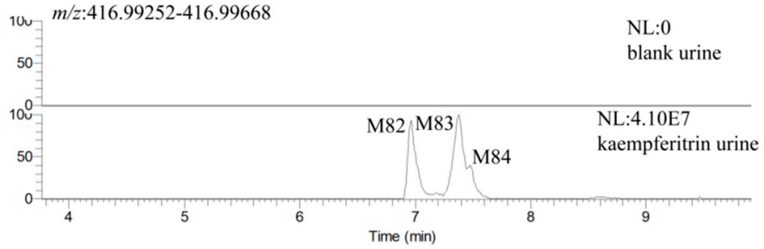

Figure S32 The EICs of **M82–M84** in the blank and kaempferitrin urine samples in negative ion mode.

(15) Dehydroxylated apigenin A, B-rings disulfates (M85–M89)

The EICs of **M85–M89** are presented in **Figure S33**. In the negative ion mode, the *m/z* 412.96323, *m/z* 412.96338, *m/z* 412.96359, *m/z* 412.96328, and *m/z* 412.96327 ($[M–H]^-$) was visible at the MS spectra, MF was predicted to be $C_{15}H_{10}O_{10}S_2$, and the MS^2 showed the fragment ions of *m/z* 333.0076, *m/z* 253.0506, *m/z* 205.9788 (*m/z* 125.0246 + SO_3), *m/z* 187.0068 (*m/z* 107.0504 + SO_3), *m/z* 96.9600, and *m/z* 79.9573, of which *m/z* 96.9600, *m/z* 79.9573 were the characteristic fragment ions for sulfation, *m/z* 205.9788 was presumed to be sulfated in the A-ring, *m/z* 187.0068 was presumed to be sulfated in the B-ring, and the compounds were identified as dehydroxylated apigenin A, B-rings disulfates.

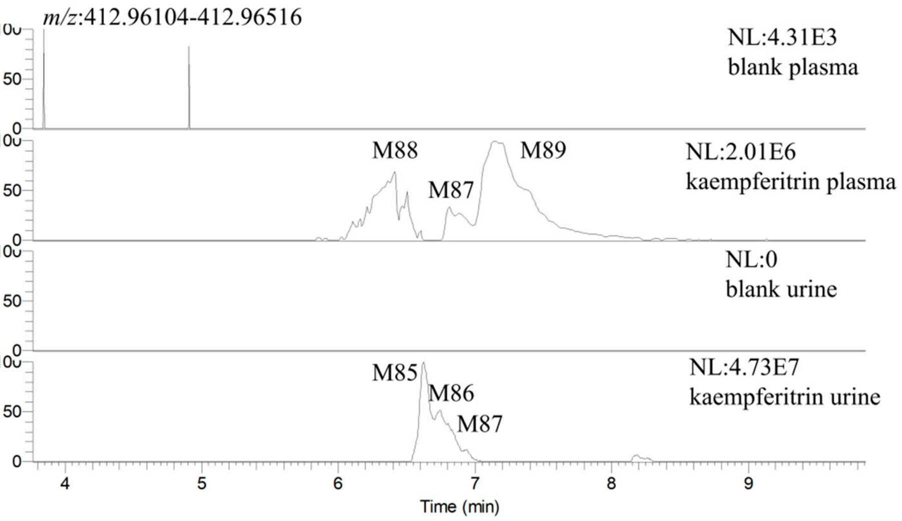

Figure S33 The EICs of **M85–M89** in the blank plasma, kaempferitrin plasma, blank urine, and kaempferitrin urine samples in negative ion mode.

(16) Dihydroxylated flavane disulfates (M90 and M91)

The EICs of **M90** and **M91** are presented in **Figure S34**. In the negative ion mode, the *m/z* 400.99976 and *m/z* 401.00009 ($[M–H]^-$) were visible at the MS spectra, and MF was predicted to be $C_{15}H_{14}O_9S_2$ and the MS^2 showed the fragment ions of *m/z* 321.0419, *m/z* 241.0870, *m/z* 135.0452 ($C_8H_7O_2$, $^{0,2}A^-$), *m/z* 121.0296 ($C_7H_5O_2$, $^{0,2}A^-$), *m/z* 119.0505 (C_8H_7O, $^{1,3}B^-$), and *m/z* 79.9574, of which *m/z* 79.9574 was a sulfated fragmentation ion, and the compounds were identified as dihydroxylated flavane disulfates. Entering $C_{15}H_{14}O_9S_2$ into Scifinder retrieved 13 compounds with the same formula, and the presumed compounds were not found and were presumed to be new compounds.

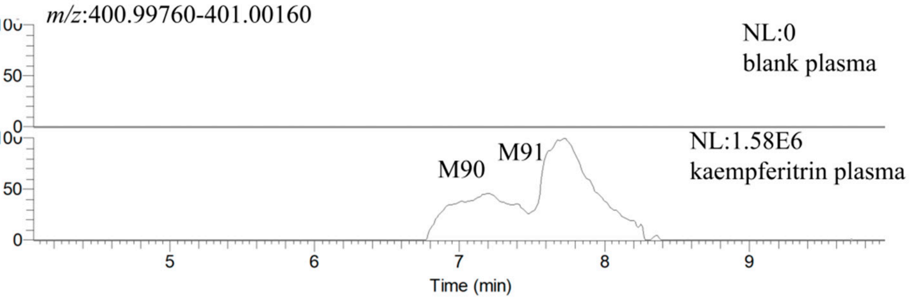

Figure S34 The EICs of **M90** and **M91** in the blank and kaempferitrin plasma samples in negative ion mode.

(17) Ethyl-phenol disulfate (M92)

The EICs of **M92** are presented in **Figure S35**. In the negative ion mode, the *m/z* 280.97919 ($[M–H]^{-}$) was visible at the MS spectra, the MF was predicted to be $C_8H_{10}O_7S_2$, with *m/z* 280.97919 as the parent ion, and the MS^2 showed the fragment ions of *m/z* 201.02274 (*m/z* 121.06599 + SO_3), *m/z* 121.06599 (C_8H_9O, $^{1,3}A^{-}$), and *m/z* 79.95739, of which the *m/z* 79.95739 was the characteristic fragment ion for sulfation, and the compound was identified to be ethyl-phenol disulfate.

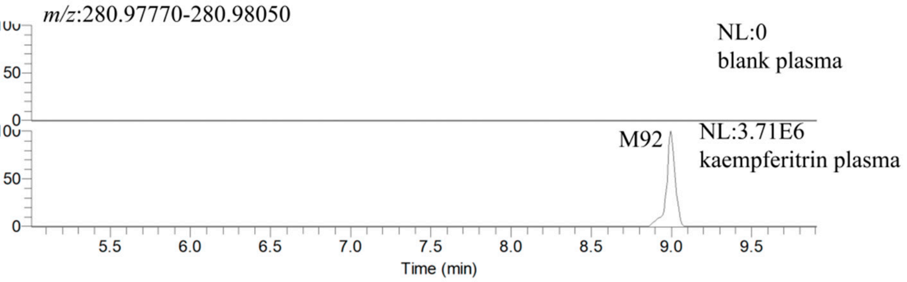

Figure S35 The EICs of **M92** in the blank and kaempferitrin plasma samples in negative ion mode.

2.5 Characterization of phase II metabolites: glucuronidated metabolites (M93–M165) with Figures S36–S62

(1) Methylated apigenin glucuronides (M93 and M94)

The EICs of **M93** and **M94** are presented in **Figure S36**. In the negative ion mode, the *m/z* 459.09210 and *m/z* 459.09250 ($[M–H]^{-}$) were visible at the MS spectra, MF was predicted to be $C_{22}H_{20}O_{11}$, and the MS^2 showed the fragment ions of *m/z* 283.0614, *m/z* 268.0379, *m/z* 240.0429, *m/z* 175.0249, *m/z* 113.0245, *m/z* 85.0295, and *m/z* 59.0319, of which *m/z* 175.0249 was a characteristic fragment ion of glucuronidation, which was presumed to be a metabolite of glucuronidation, and the compounds were identified as methylated apigenin glucuronides.

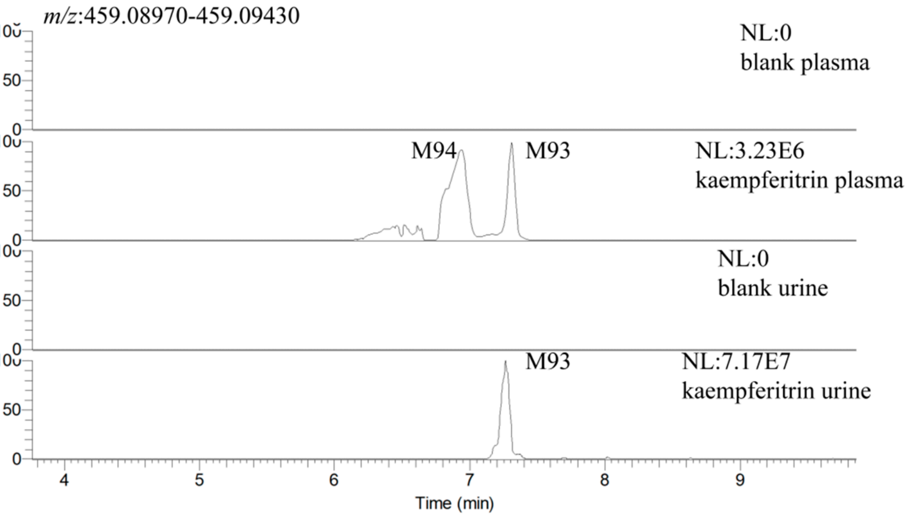

Figure S36 The EICs of **M93** and **M94** in the blank plasma, kaempferitrin plasma, blank urine, and kaempferitrin urine samples in negative ion mode.

(2) Kaempferol glucuronides (M95–M98)

The EICs of **M95**–**M98** are presented in **Figure S37**. In the negative ion mode, the *m/z* 461.07139, *m/z* 461.07178, *m/z* 461.07153, and *m/z* 461.07141 ($[M–H]^–$) were visible at the MS spectra, MF was predicted to be $C_{21}H_{18}O_{12}$, and the MS^2 showed the fragment ions of *m/z* 285.0407, *m/z* 257.0461, *m/z* 151.0038 ($C_7H_3O_4$, $^{1,3}A^–$), *m/z* 121.0661 (C_8H_9O, $^{1,3}B^–$), *m/z* 175.0249, *m/z* 113.0245, and *m/z* 85.0295, of which *m/z* 175.0249 was a glucuronidation characteristic fragmentation ion, and the compounds were identified as kaempferol glucuronides. Based on CLogP, the compounds were identified as **M95**: kaempferol-5-*O*-glucuronide (CLogP: –0.17049), **M96**: kaempferol-3-*O*-glucuronide (CLogP: –0.218557), **M97**: kaempferol-4'-*O*-glucuronide (CLogP: 0.135692), **M98**: kaempferol-7-*O*-glucuronide (CLogP: 0.182951).

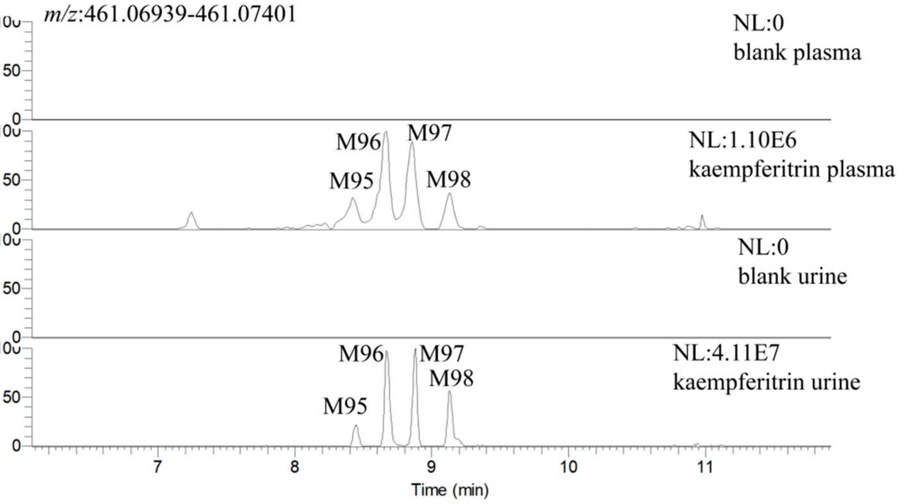

Figure S37 The EICs of **M95–M98** in the blank plasma, kaempferitrin plasma, blank urine, and kaempferitrin urine samples in negative ion mode.

(3) Naringin glucuronides (M99–M102)

The EICs of **M99–M102** are presented in **Figure S38**. In the negative ion mode, the *m/z* 447.09256, *m/z* 447.09262, *m/z* 447.09283 and *m/z* 447.09265 ($[M–H]^–$) were visible at the MS spectra, MF was predicted to be $C_{21}H_{20}O_{11}$, and the MS^2 visualized the fragment ions of *m/z* 271.0622, *m/z* 175.0250, *m/z* 151.0039 ($C_7H_3O_4$, $^{1,3}A^–$), *m/z* 119.0506 (C_8H_7O, $^{1,3}B^–$), *m/z* 113.0246, and *m/z* 85.0296, of which *m/z* 175.0250 was a glucuronidation characteristic fragmentation ion, and the compounds were identified as naringin glucuronides. Based on CLogP the compounds were identified as **M99**: naringenin-5-*O*-glucuronide (CLogP: –0.536774), **M100**: naringenin-7-*O*-glucuronide (CLogP: 0.406323), **M101**: naringenin-4'-*O*-glucuronide (CLogP: 0.469553), **M102**: naringenin isomer glucuronide.

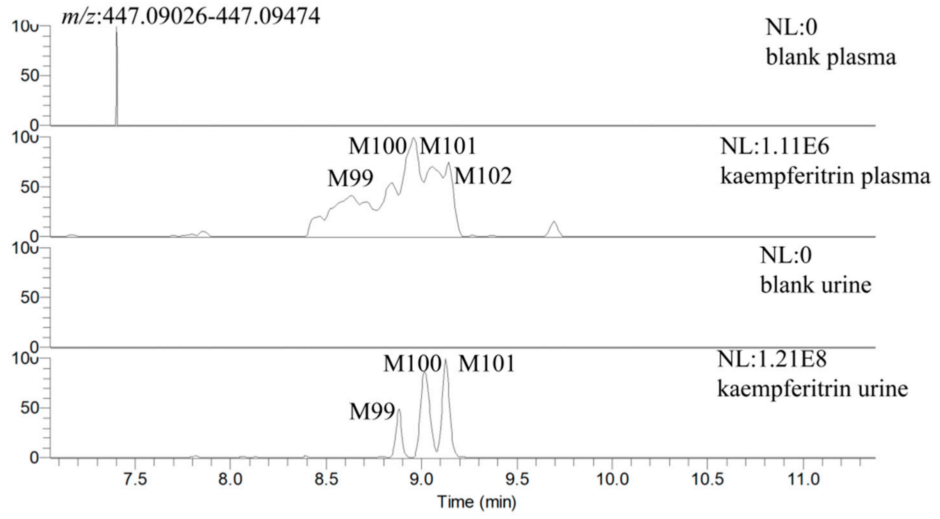

Figure S38 The EICs of **M99–M102** in the blank plasma, kaempferitrin plasma, blank urine, and kaempferitrin urine samples in negative ion mode.

(4) Apigenin glucuronides (M103–M106)

The EICs of **M103–M106** are presented in **Figure S39**. In negative ion mode, the *m/z* 445.07663, *m/z* 445.07791, *m/z* 445.07675, and *m/z* 445.07672 ($[M–H]^-$) were visible in the MS spectra, MF was predicted as $C_{21}H_{18}O_{11}$, and MS^2 showed *m/z* 269.0458, *m/z* 225.0561, *m/z* 201.0231, *m/z* 175.0250, *m/z* 113.0244, and *m/z* 85.0295. The *m/z* 175.0248 was a characteristic fragment ion of glucuronidation, and compounds were identified as apigenin glucuronides. **M103**: apigenin-5-*O*-glucuronide (CLogP: 0.0300615), **M104**: apigenin-7-*O*-glucuronide (CLogP: 0.930062), **M105**: apigenin-4'-*O*-glucuronide (CLogP: 0.924951), **M106**: apigenin isomer glucuronide.

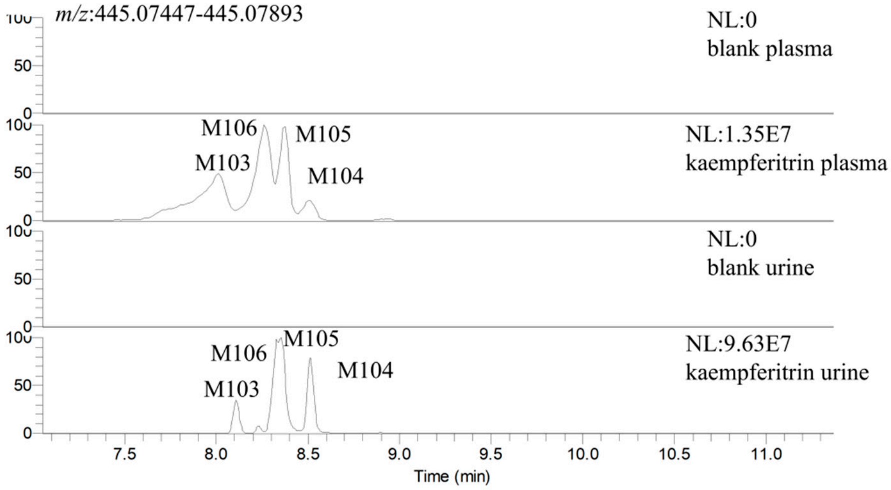

Figure S39 The EICs of **M103–M106** in the blank plasma, kaempferitrin plasma, blank urine, and kaempferitrin urine samples in negative ion mode.

(5) Trihydroxylated dihydrogenated flavone glucuronides (M107–M110)

The EICs of **M107–M110** are presented in **Figure S40**. In the negative ion mode, the *m/z* 433.11279, *m/z* 433.11325, *m/z* 433.11401, *m/z* 433.11316, and *m/z* 433.11307 ($[M–H]^-$) were visible in the MS spectra, MF was predicted as $C_{21}H_{22}O_{10}$, and MS^2 showed *m/z* 257.0822, *m/z* 175.0250, *m/z* 151.0403 ($C_8H_7O_3$, $^{0,2}A^-$), *m/z* 119.0504 (C_8H_7O, $^{1,3}B^-$), *m/z* 137.0246 ($C_7H_5O_3$, $^{0,3}A^-$), *m/z* 113.0245, and *m/z* 85.0295, of which *m/z* 175.0250 was the characteristic fragmentation ion of glucuronidation. Compared to kaempferol, removing the MF of $C_6H_8O_6$, increased MF by 4 hydrogen atoms and decreased by 2 oxygen atoms, with a DBE of 9. It was presumed that C-ring reduction and dehydroxylation occurred, and the compounds were identified as trihydroxylated dihydrogenated flavone glucuronides.

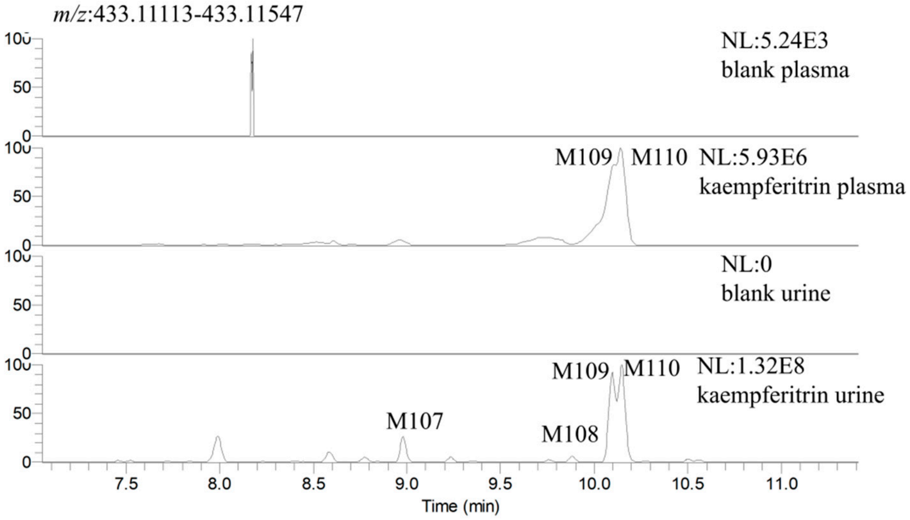

Figure S40 The EICs of **M107–M110** in the blank plasma, kaempferitrin plasma, blank urine, and kaempferitrin urine samples in negative ion mode.

(6) Dehydroxylated naringin glucuronides (M111–M115)

The EICs of **M111–M115** are presented in **Figure S41**. In the negative ion mode, the *m/z* 431.09702, *m/z* 431.09750, *m/z* 431.09744, *m/z* 431.09763, and *m/z* 431.09757 ($[M–H]^–$) were visible in the MS spectra, MF was predicted to be $C_{21}H_{20}O_{10}$, and MS^2 visualized the *m/z* 255.0665, *m/z* 175.0249, *m/z* 149.0246 ($C_8H_5O_3$, $^{0,2}A^–$), *m/z* 113.0245, *m/z* 135.0088 ($C_7H_3O_3$, $^{0,2}A^–$), *m/z* 119.0505 (C_8H_7O, $^{1,3}B^–$), *m/z* 85.0295, and *m/z* 59.0319. The *m/z* 175.0249 was the characteristic fragment ion of glucuronidation. The MF removal of $C_6H_8O_6$, the reduction of MF by 2 OH, compared with kaempferol, and the DBE was 10, which was presumed to be dehydroxylated and hydrogenated, the compounds were identified as dehydroxylated naringin glucuronides.

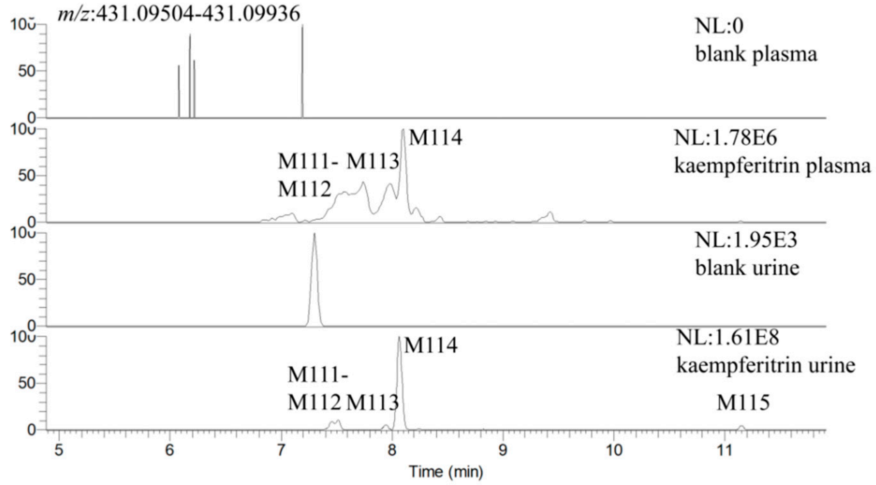

Figure S41 The EICs of **M111–M115** in the blank plasma, kaempferitrin plasma, blank urine, and kaempferitrin urine samples in negative ion mode.

(7) Dehydroxylated apigenin glucuronides (M116–M120)

The EICs of **M116–M120** are presented in **Figure S42**. In negative ion mode, the *m/z* 429.08194, *m/z* 429.08136, *m/z* 429.08173, *m/z* 429.08191, and *m/z* 429.08212 ($[M–H]^-$) were visible at the MS spectra, MF was predicted to be $C_{21}H_{18}O_{10}$, and the MS^2 showed the fragment ions of *m/z* 253.0507, *m/z* 175.0249, *m/z* 113.0245, *m/z* 85.0295, and *m/z* 59.0319, of which *m/z* 175.0249 was a characteristic fragment ion of glucuronidation, which were presumed to be the metabolite of glucuronidations, and the compounds were identified as dehydroxylated apigenin glucuronides.

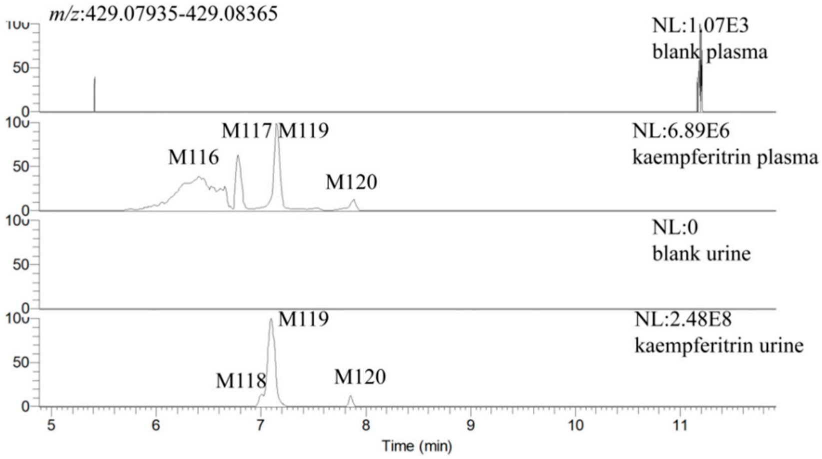

Figure S42 The EICs of **M116–M120** in the blank plasma, kaempferitrin plasma, blank urine, and kaempferitrin urine samples in negative ion mode.

(8) Dihydroxylated flavane glucuronides (M121–M127)

The EICs of **M121–M127** are presented in **Figure S43**. In the negative ion mode, the *m/z* 417.11819, *m/z* 417.11807, *m/z* 417.11801, *m/z* 417.11816, *m/z* 417.11810, *m/z* 417.11819, and *m/z* 417.11821 ($[M–H]^-$) were visible at the MS spectra, MF was predicted to be $C_{21}H_{22}O_9$, and the MS^2 showed the fragment ions of *m/z* 241.0871, *m/z* 175.0249, *m/z* 121.0296 ($C_7H_5O_2$, $^{0,2}B^-$), *m/z* 135.0453 ($C_8H_7O_2$, $^{0,2}A^-$), *m/z* 113.0245, and *m/z* 85.0295, of which *m/z* 175.0249 was a characteristic fragment ion for glucuronidation, and the A-ring and C-ring were presumed to be

dehydroxylated, and the compounds were identified as dihydroxylated flavane glucuronides. Inputting $C_{21}H_{22}O_9$ into Scifinder produced 429 compounds with the same formula, but not the presumed compounds, which were presumed to be new compounds.

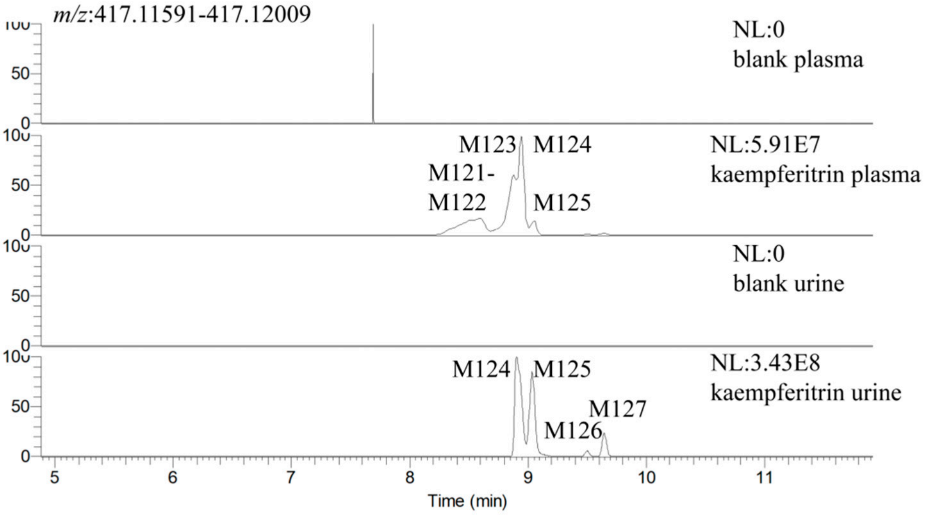

Figure S43 The EICs of **M121–M127** in the blank plasma, kaempferitrin plasma, blank urine, and kaempferitrin urine samples in negative ion mode.

(9) Dimethylated kaempferol glucuronide (M128)

The EICs of **M128** are presented in **Figure S44**. In the negative ion mode, the *m/z* 489.10278 ($[M–H]^–$) was visible at the MS spectra, MF was predicted to be $C_{23}H_{22}O_{12}$, with *m/z* 489.1029 as the parent ion, and showed the fragment ions of *m/z* 313.07197, *m/z* 298.04849, *m/z* 283.02502, *m/z* 270.05350, *m/z* 255.02979, *m/z* 175.02489, *m/z* 113.02455, and *m/z* 85.02953, of which *m/z* 175.02489 was the glucuronidation characteristic fragmentation ions, 313.07197, *m/z* 298.04849, and *m/z* 283.02502 differed by 15.0234. Compared with kaempferol, MF increased by a unit of C_2H_4, DBE was unchanged, presumably two methylations took place, and the compound was identified as A, B-rings dimethylated kaempferol glucuronide.

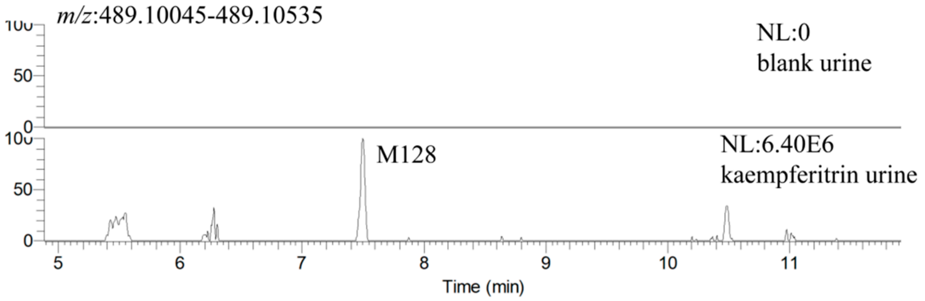

Figure S44 The EICs of **M128** in the blank and kaempferitrin urine samples in negative ion mode.

(10) Methylated hydrogenated kaempferol glucuronide (M129)

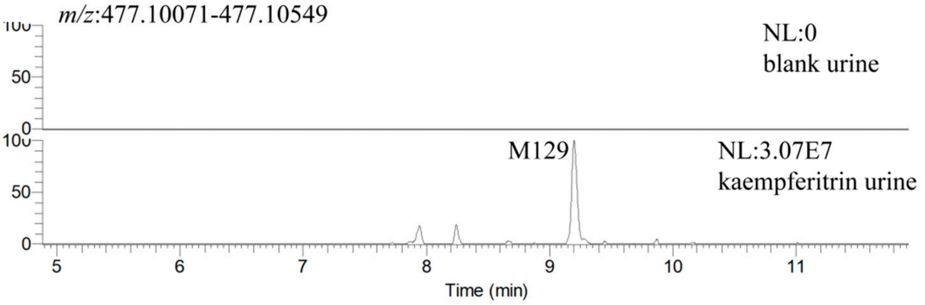

Figure S45 The EICs of **M129** in the blank and kaempferitrin urine samples in negative ion mode.

(10) Methylated dedihydroxylated dihydrogenated kaempferol glucuronides (M130–M132)

The EICs of **M130–M132** are presented in **Figure S46**. In the negative ion mode, the *m/z* 447.12900, *m/z* 447.12930 and *m/z* 447.12894 ($[M–H]^-$) were visible at the MS spectra, MF was predicted to be $C_{22}H_{24}O_{10}$, and the MS^2 showed the fragment ions of *m/z* 271.0934, *m/z* 175.0250, *m/z* 149.0609 ($C_9H_9O_2$, $^{0,2}A^-$), *m/z* 121.0296 ($C_7H_5O_2$, $^{0,2}B^-$), *m/z* 135.0454 ($C_8H_7O_2 – CH_2$, $^{0,2}A^-$), *m/z* 113.0245, and *m/z* 85.0295, where *m/z* 175.0249 was a characteristic fragment ion for glucuronidation, compared with kaempferol, MF decreased by 2 oxygen atoms and increased by a unit of CH_6, DBE was 9, presumably the C-ring was reduced in its entirety, and the methylation was presumed to have occurred in the A-ring, and the compounds were identified as methylated dedihydroxylated dihydrogenated kaempferol glucuronides. When $C_{22}H_{24}O_{10}$ was entered into Scifinder, 328 compounds with the same formula were found, but there were no presumed compounds, so it was presumed that they might be new compounds.

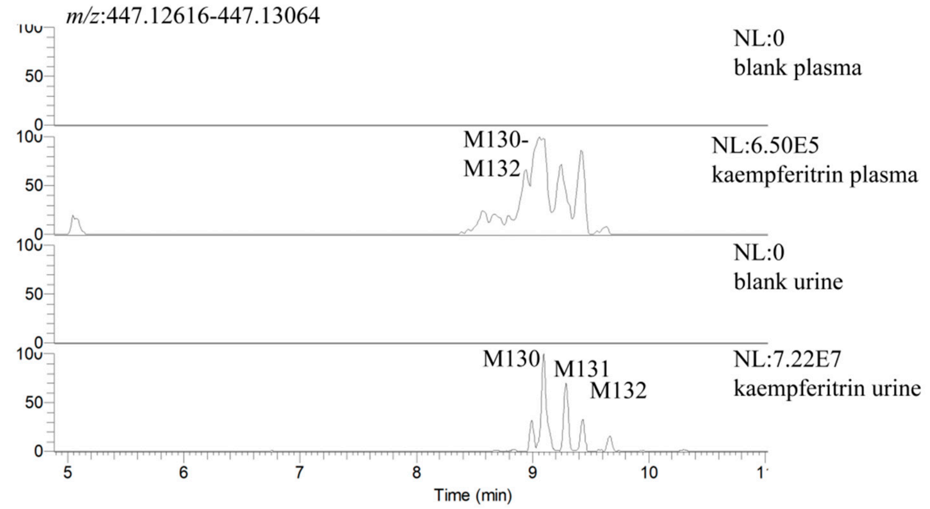

Figure S46 The EICs of **M130–M132** in the blank plasma, kaempferitrin plasma, blank urine, and kaempferitrin urine samples in negative ion mode.

(12) Hydrogenated kaempferol glucuronides (M133 and M134)

The EICs of **M133** and **M134** are presented in **Figure S47**. In the negative ion mode, the *m/z* 463.08707 and *m/z* 463.08762 ($[M–H]^–$) were visible at the MS spectra, MF was predicted to be $C_{21}H_{20}O_{12}$, and the MS^2 showed the fragment ions of *m/z* 287.0564, *m/z* 259.0614, *m/z* 243.0664, *m/z* 201.0555, *m/z* 165.0193 ($C_8H_5O_4$, $^{1,2}A^–$), *m/z* 152.0116 ($C_7H_4O_4$•, $^{1,3}A^–$), *m/z* 125.0246 ($C_6H_5O_3$, $^{1,4}A^–$), *m/z* 107.0503 (C_7H_7O, $^{1,2}A^–$), *m/z* 113.0245, and *m/z* 85.0295, in which *m/z* 287.0564 and *m/z* 463.0871 differed by a relative molecular mass of 176.0307 for the characteristic fragments of glucuronidation, and the compounds were identified as hydrogenated kaempferol glucuronides.

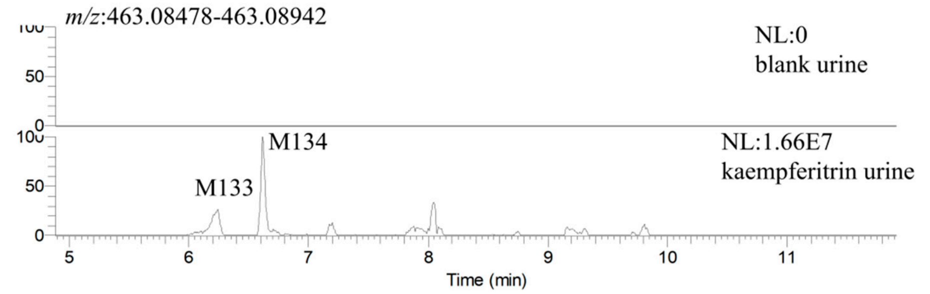

Figure S47 The EICs of **M133** and **M134** in the blank and kaempferitrin urine samples in negative ion mode.

(13) Methylated dehydroxylated naringenin glucuronide (M135)

The EICs of **M135** are presented in **Figure S48**. In negative ion mode, the *m/z* 445.11316 ([M–H]⁻) was visible at the MS spectra, MF was predicted to be $C_{22}H_{22}O_{10}$, and the MS^2 showed the fragment ions of *m/z* 269.08194, *m/z* 254.05867, *m/z* 175.02493, *m/z* 113.02455, and *m/z* 85.02953, of which *m/z* 175.02493 was the characteristic fragment for glucuronidation, removal of $C_6H_8O_6$, the MF was increased of a unit of CH_4, decreased of 2 oxygen atoms in MF compared to kaempferol, DBE was 10, presumably dehydroxylation, C-ring reduction and methylation, the compound was identified as a methylated dehydroxylated naringenin glucuronide. Entering $C_{22}H_{22}O_{10}$ into Scifinder retrieved 452 compounds with the same formula, and the presumed compound was not found and was presumed to be a new compound.

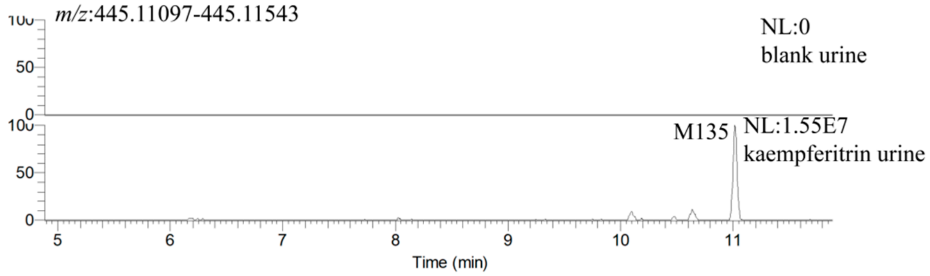

Figure S48 The EICs of **M135** in the blank and kaempferitrin urine samples in negative ion mode.

(14) Methylated dehydroxylated apigenin glucuronide (M136)

The EICs of **M136** are presented in **Figure S49**. In negative ion mode, the *m/z* 443.09756 ([M–H]⁻) was visible at the MS spectra, MF was predicted to be $C_{22}H_{20}O_{10}$, and the MS^2 showed the fragment ions of *m/z* 267.06650, *m/z* 252.04294, *m/z* 175.02492, *m/z* 113.02456, and *m/z* 85.02955, of which *m/z* 175.02492 was the characteristic fragment ion of glucuronidation, removal of $C_6H_8O_6$, compared with kaempferol, MF increased a unit of CH_2, decreased 2 oxygen atom, DBE was unchanged, it was presumed that dedihydroxylation, methylation and hydrogenation occurred, the compound was identified as a methylated dehydroxylated apigenin glucuronide.

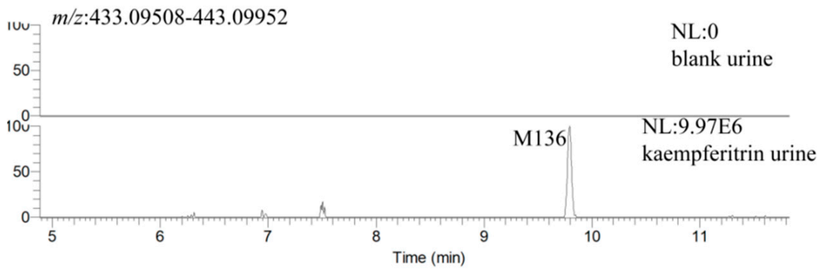

Figure S49 The EICs of **M136** in the blank and kaempferitrin urine samples in negative ion mode.

(15) Dihydroxylated dihydrogenated flavane glucuronide (M137–M142)

The EICs of **M137**–**M142** are presented in **Figure S50**. In the negative ion mode, the *m/z* 419.13397, *m/z* 419.13403, *m/z* 419.13397, *m/z* 419.13336, *m/z* 419.13370, and *m/z* 419.13310 ([M–H]$^-$) were visible at the MS spectra, MF was predicted to be $C_{21}H_{24}O_9$, and the MS^2 showed the fragment ions of *m/z* 243.1029, *m/z* 175.0250, *m/z* 123.0454 ($C_7H_7O_2$, $^{0,3}A^-$), *m/z* 113.0245, *m/z* 85.0295, and *m/z* 59.0138, of which *m/z* 175.0249 was a neutral fragment of glucuronidation, and the compounds were identified as dihydroxylated dihydrogenated flavane glucuronides. When $C_{21}H_{24}O_9$ was entered into Scifinder, 357 compounds with the same formula were found, but there was no presumed compounds, which were presumed to be new compounds.

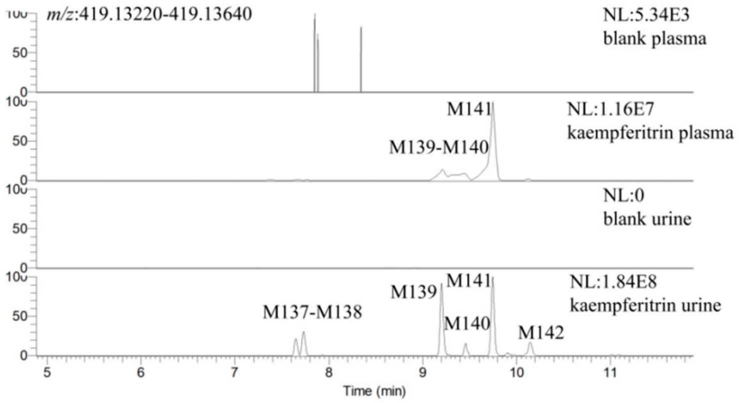

Figure S50 The EICs of **M137**–**M142** in the blank plasma, kaempferitrin plasma, blank urine, and kaempferitrin urine samples in negative ion mode.

(16) Dimethylatedated apigenin glucuronide (M143)

The EICs of **M143** are presented in **Figure S51** In the negative ion mode, the *m/z* 473.10779 ([M–H]$^-$ was visible at the MS spectra, MF was predicted to be $C_{23}H_{22}O_{11}$, the MS^2 showed the

fragment ions of *m/z* 297.07718, *m/z* 282.05362, *m/z* 267.03009, *m/z* 175.02495, *m/z* 113.02457 and *m/z* 85.02956, of which *m/z* 175.02495 was a glucuronidation characteristic fragment ion, *m/z* 297.07718, *m/z* 282.05362, *m/z* 267.03009 the difference of 15.02356 and 15.02353 was calculated precisely as methylated fragments and the compound was identified as dimethylated apigenin glucuronide. Entering $C_{23}H_{22}O_{11}$ into Scifinder retrieved 40 compounds with the same formula, and the presumed compound was not found and was presumed to be a new compound.

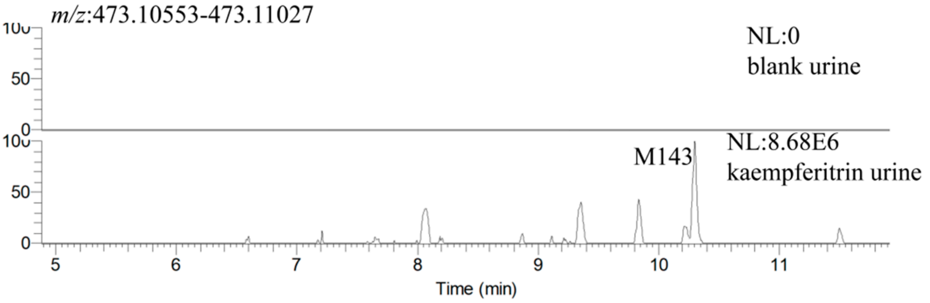

Figure S51 The EICs of **M143** in the blank and kaempferitrin urine samples in negative ion mode.

(17) Methylated dihydrogenated apigenin glucuronide (M144)

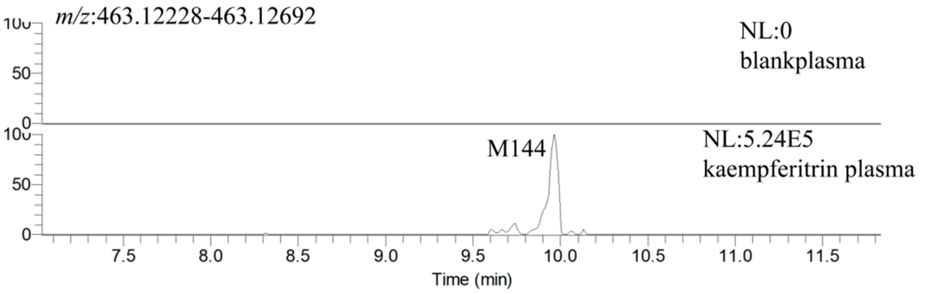

Figure S52 The EICs of **M144** in the blank and kaempferitrin plasma samples in negative ion mode.

(18) Dimethylated quercetin glucuronide (M145)

The EICs of **M145** are presented in **Figure S53**. In the negative ion mode, the *m/z* 505.09735 ($[M–H]^-$) was visible at the MS spectra, MF was predicted to be $C_{23}H_{22}O_{13}$, and the MS^2 showed the fragment ions of *m/z* 329.06693, *m/z* 314.04349, *m/z* 299.02005, *m/z* 285.03964, *m/z* 271.02466, *m/z* 175.02492, *m/z* 113.02454, of which *m/z* 175.02492 was a glucuronidation characteristic fragment ion, compared with kaempferol, the MF increased a unit of C_2H_4O, the DBE was 11, presumably two methylations and one hydroxylation occurred, and the compound was identified as dimethylated

quercetin glucuronide.

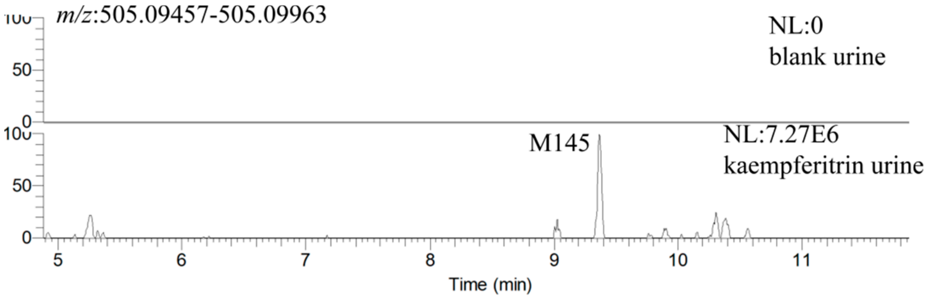

Figure S53 The EICs of **M145** in the blank and kaempferitrin urine samples in negative ion mode.

(19) Apigenin diglucuronide (M146)

The EICs of **M146** are presented in **Figure S54**. In the negative ion mode, the *m/z* 621.10858 ($[M–H]^-$) was visible at the MS spectra, MF was predicted to be $C_{27}H_{26}O_{17}$, and the MS^2 showed the fragment ions of *m/z* 445.07834, *m/z* 269.04578, *m/z* 201.05608, *m/z* 175.02507, *m/z* 107.05035 (C_7H_7O, $^{1,2}A^-$), *m/z* 113.02458 and *m/z* 85.02953, of which *m/z* 175.02507 was the characteristic fragment ion for glucuronidation, and the compound was identified as apigenin diglucuronide.

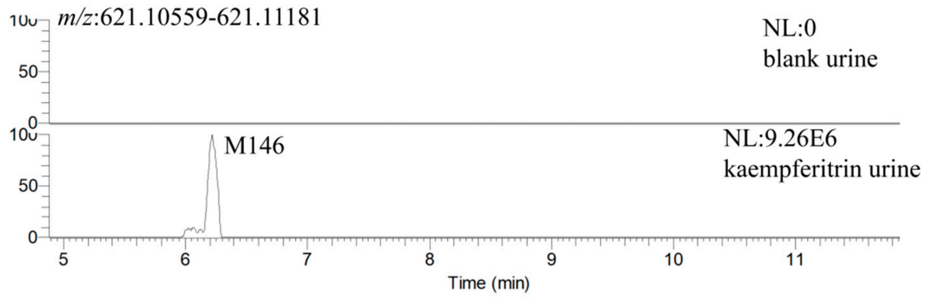

Figure S54 The EICs of **M146** in the blank and kaempferitrin urine samples in negative ion mode.

(20) Dehydroxylated apigenin glucuronide methylated rhamnose (M147)

The EICs of **M147** are presented in **Figure S55**. In the negative ion mode, the *m/z* 589.15517 ($[M–H]^-$) was visible at the MS spectra, MF was predicted to be $C_{28}H_{30}O_{14}$, and the MS^2 showed the fragment ions of *m/z* 429.08292, *m/z* 253.05074, *m/z* 175.02480, *m/z* 113.02451 and *m/z* 85.02950, of which *m/z* 175.02480 was the characteristic fragment ion of glucuronidation, *m/z* 589.15517 and *m/z* 429.08292 difference in relative molecular mass of 160.07225, precision calculations for the methylated rhamnose, the compound was identified as A-ring dehydroxylated apigenin glucuronide

methylated rhamnose.

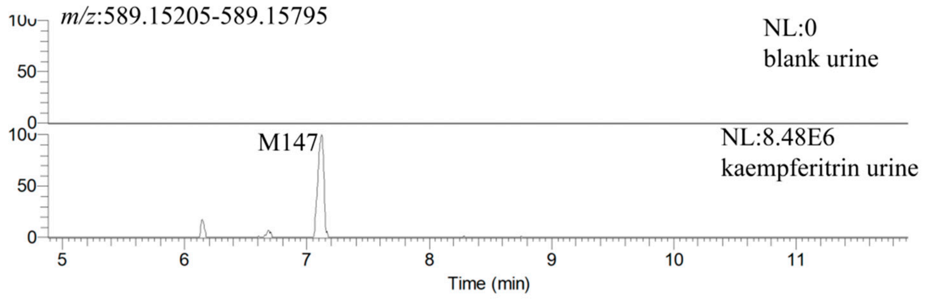

Figure S55 The EICs of **M147** in the blank and kaempferitrin urine samples in negative ion mode.

(21) Phenol glucuronides (M148 and M149)

The EICs of **M148** and **M149** are presented in **Figure S56**. In the negative ion mode, the *m/z* 269.06685 and *m/z* 269.06686 ($[M–H]^{–}$) were visible at the MS spectra, MF was predicted to be $C_{12}H_{14}O_7$, and the MS^2 showed the fragment ions of *m/z* 175.0249, *m/z* 93.0346, *m/z* 113.0245 and *m/z* 85.0295. Among them, *m/z* 175.0249 was the characteristic fragment ion for glucuronidation, and the compounds were identified as phenol glucuronides.

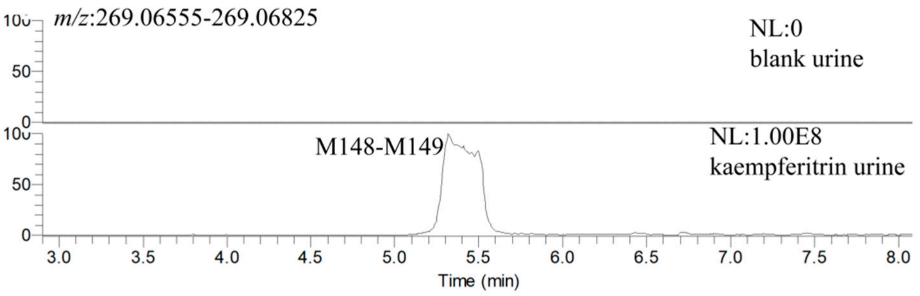

Figure S56 The EICs of **M148** and **M149** in the blank and kaempferitrin urine samples in negative ion mode.

(22) Hydroxystyrene glucuronide (M150)

The EICs of **M150** are presented in **Figure S57**. In negative ion mode, the *m/z* 295.08185 ($[M–H]^{–}$) was visible at the MS spectra, MF was predicted to be $C_{14}H_{16}O_7$, and the MS^2 showed the fragment ions of *m/z* 175.02499, *m/z* 119.05043, *m/z* 113.02459 and *m/z* 85.02956, of which *m/z* 175.02499 was a characteristic fragment ion for glucuronidation. The compound was identified as hydroxystyrene glucuronide.

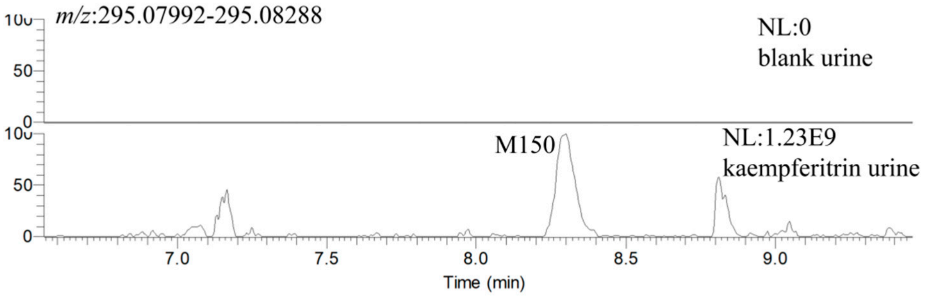

Figure S57 The EICs of **M150** in the blank and kaempferitrin urine samples in negative ion mode.

(23) Methylated phenol glucuronides (M151–M155)

The EICs of **M151–M155** are presented in **Figure S58**. In negative ion mode, the *m/z* 283.08182, *m/z* 283.08179, *m/z* 283.08200, *m/z* 283.08218 and *m/z* 283.08211 ($[M–H]^-$) were visible at the MS spectra, and MF was predicted to be $C_{13}H_{16}O_7$ and the MS^2 showed the fragment ions of *m/z* 175.0249, *m/z* 107.0503 (C_7H_7O, $^{1,2}A^-$), *m/z* 113.0245, and *m/z* 85.0295, of which *m/z* 175.0249 was the characteristic fragment ion for glucuronidation, and the compounds were identified as methylated phenol glucuronides.

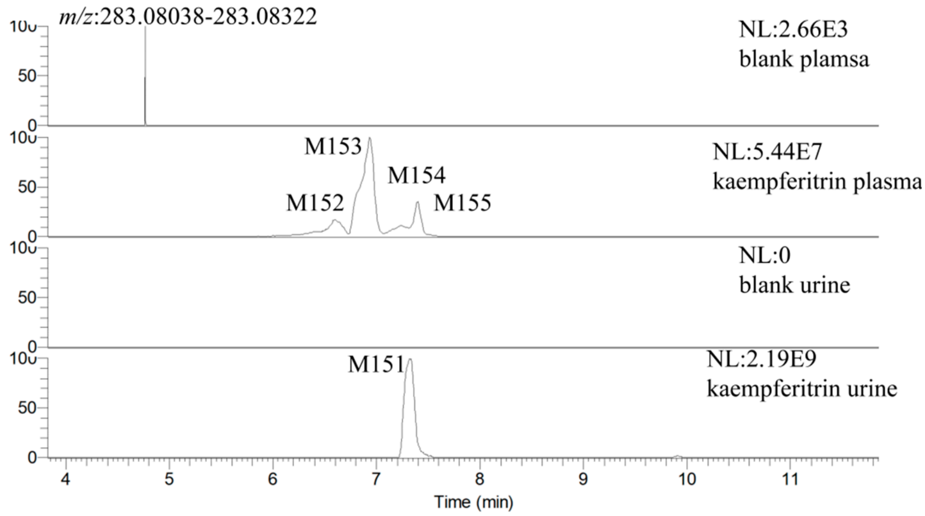

Figure S58 The EICs of **M151-M155** in the blank plasma, kaempferitrin plasma, blank urine, and kaempferitrin urine samples in negative ion mode.

(24) Hydroxyethylbenzene glucuronides (M156 and M157)

The EICs of **M156** and **M157** are presented in **Figure S59**. In the negative ion mode, the *m/z* 297.09750 and *m/z* 297.09732 ($[M–H]^-$) were visible in the MS spectra, the MF was predicted to be

$C_{14}H_{18}O_7$, and the MS^2 showed the fragment ions of *m/z* 175.0250, *m/z* 121.0660 (C_8H_9O, $^{1,3}A^-$), *m/z* 113.0246, *m/z* 85.0295, and *m/z* 59.0319, of which the *m/z* 175.0249 was the characteristic fragment ion of glucuronidation, which were presumed to be the metabolite of glucuronidations. Therefore, the compounds were identified as hydroxyethylbenzene glucuronides.

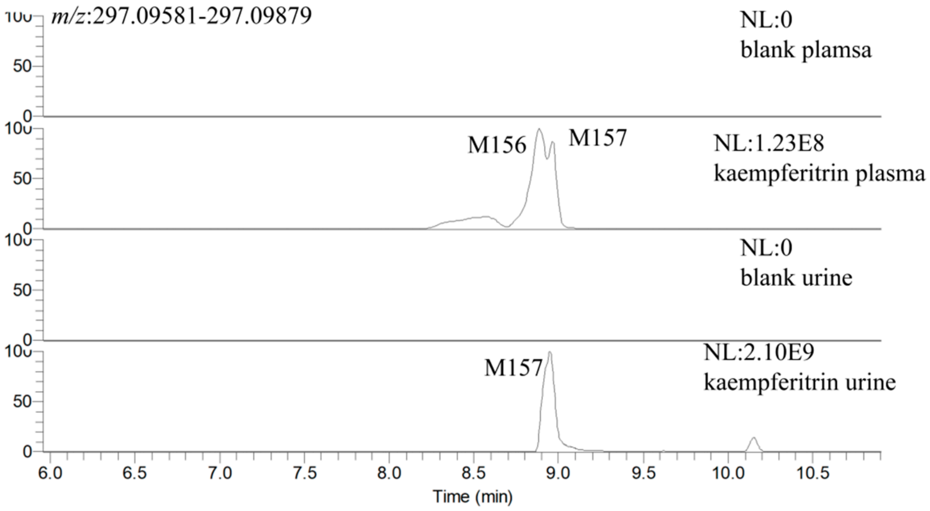

Figure S59 The EICs of **M156** and **M157** in the blank plasma, kaempferitrin plasma, blank urine, and kaempferitrin urine samples in negative ion mode.

(25) Hydroxybenzyl alcohol glucuronides (M158 and M159)

The EICs of **M158** and **M159** are presented in **Figure S60**. In the negative ion mode, the *m/z* 299.07684 and *m/z* 299.07770 ($[M–H]^-$) were visible at the MS spectra, and MF was predicted to be $C_{13}H_{16}O_8$. In the MS^2 spectra, the fragment ions of *m/z* 175.0250, *m/z* 123.0453 ($C_7H_7O_2$, $^{0,3}A^-$), *m/z* 113.0245, *m/z* 85.0295, and *m/z* 59.0319 were observed, of which the *m/z* 175.0250 was a characteristic fragment ion of glucuronidation. Therefore, it was presumed to be the metabolite of glucuronidations, and the compounds were identified as hydroxybenzyl alcohol glucuronides.

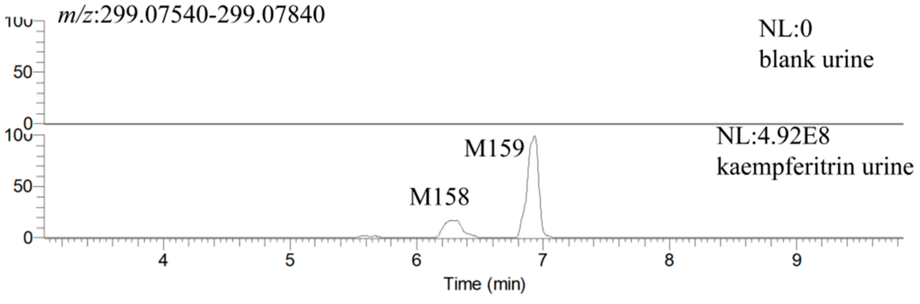

Figure S60 The EICs of **M158** and **M159** in the blank and kaempferitrin urine samples in negative ion mode.

(26) Hydroxyphenylethanol glucuronides (M160 and M161)

The EICs of **M160** and **M161** are presented in **Figure S61**. In the negative ion mode, the of *m/z* 313.09244 and *m/z* 313.09268 ($[M–H]^–$) were visible at the MS spectra, MF was predicted to be $C_{14}H_{18}O_8$, and the MS^2 showed the fragment ions of *m/z* 175.0245, *m/z* 137.0609, *m/z* 113.0246, and *m/z* 85.0296. The compounds were identified as hydroxyphenylethanol glucuronides.

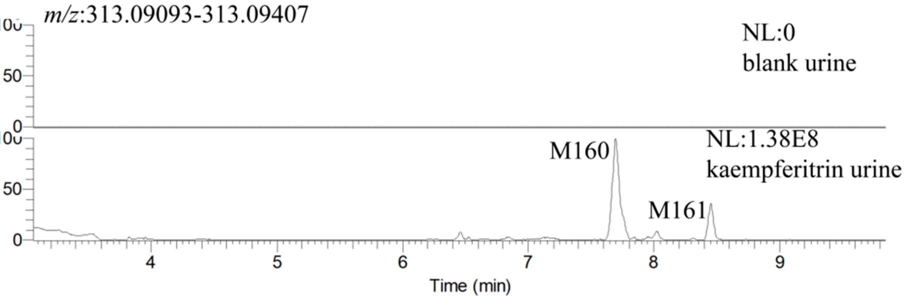

Figure S61 The EICs of **M160** and **M161** in the blank and kaempferitrin urine samples in negative ion mode.

(27) Hydroxybenzaldehyde glucuronides (M162–M165)

The EICs of **M162**–**M165** are presented in **Figure S62**. In the negative ion mode, the *m/z* 297.06085, *m/z* 297.06140, *m/z* 297.06137, and *m/z* 297.06132 ($[M–H]^–$) were visible at the MS spectra, MF was predicted to be $C_{13}H_{14}O_8$, and the MS^2 showed the fragment ions of *m/z* 175.0249, *m/z* 121.0660 (C_8H_9O, $^{1,3}A^–$), *m/z* 113.0245, *m/z* 85.0295, and *m/z* 59.0139. Among these fragment ions, *m/z* 175.0249 was the characteristic fragment ion for glucuronidation. Inputting $C_{14}H_{18}O_7$ into Scifinder, there were 733 compounds with the same formula as its general formula, which were identified as hydroxybenzaldehyde glucuronides.

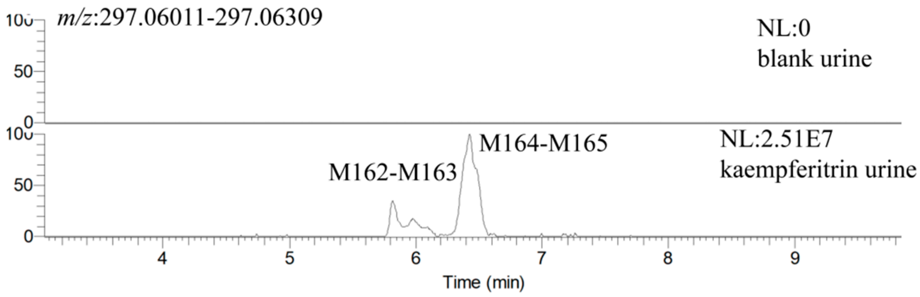

Figure S62 The EICs of **M162–M165** in the blank and kaempferitrin urine samples in negative ion mode.

2.6 Characterization of phase II metabolites: sulfated and glucuronidated metabolites (M166–M192) with Figures S63–S71

(1) Methylated apigenin glucuronyl sulfate (M166)

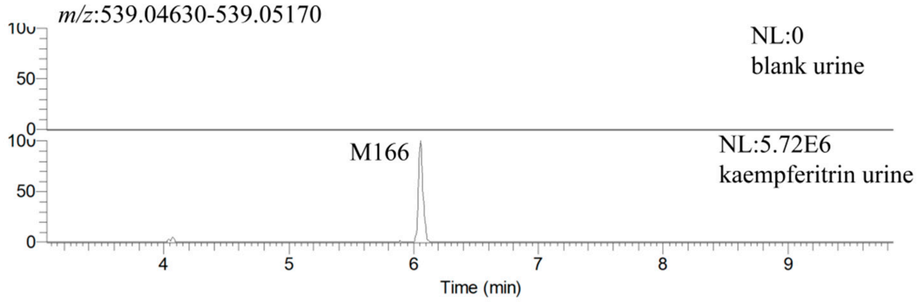

Figure S63 The EICs of **M166** in the blank and kaempferitrin urine samples in negative ion mode.

(2) Kaempferol glucuronyl sulfates (M167–M169)

The EICs of **M167–M169** are presented in **Figure S64**. In the negative ion mode, the *m/z* 541.02771, *m/z* 541.02808 and *m/z* 541.02960 ($[M–H]^-$) were visible at the MS spectra, MF was predicted to be $C_{21}H_{18}O_{15}S$, and the MS^2 showed the fragment ions of *m/z* 285.0406, *m/z* 461.0731, *m/z* 175.0249, *m/z* 229.0509, and *m/z* 257.0458, of which *m/z* 79.9573 was a sulfated characteristic fragment ion and *m/z* 175.0249 was a glucuronidated fragment ion, the compounds were identified as kaempferol glucuronyl sulfates.

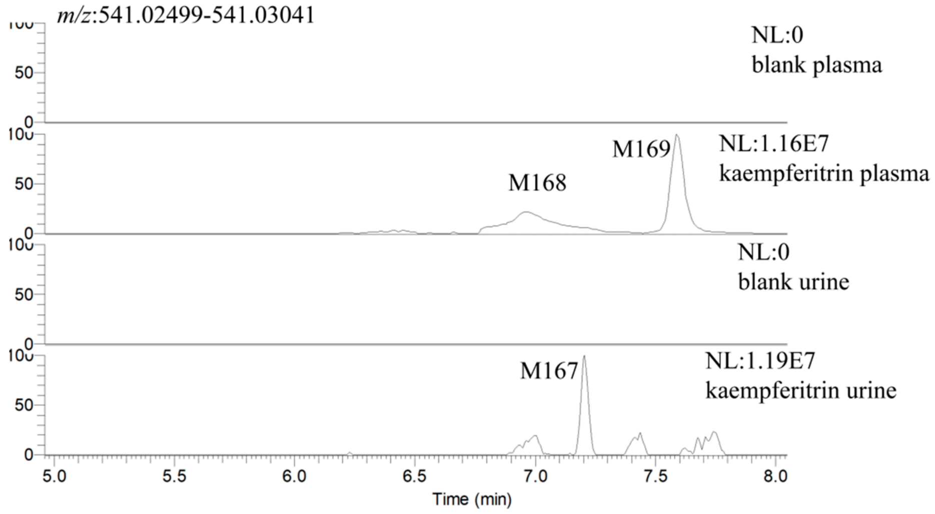

Figure S64 The EICs of **M167–M169** in the blank plasma, kaempferitrin plasma, blank urine, and kaempferitrin urine samples in negative ion mode.

(3) Naringenin glucuronyl sulfate (M170)

The EICs of **M170** are presented in **Figure S65**. In the negative ion mode, the *m/z* 527.04919 ([M–H]⁻) was visible at the MS spectra, MF was predicted to be $C_{21}H_{20}O_{14}S$, and the MS^2 showed the fragment ions of *m/z* 447.09381, *m/z* 351.01755, *m/z* 271.06149, *m/z* 175.02493, *m/z* 151.00386 ($C_7H_3O_4$, $^{1,3}A^-$), *m/z* 119.05033 (C_8H_7O, $^{1,3}B^-$), *m/z* 113.02457 and *m/z* 85.02954, of which *m/z* 175.02493 was a glucuronidation characteristic fragment ion, *m/z* 527.0491 differed from *m/z* 447.09381, *m/z* 351.01755 differed from *m/z* 271.06149 with a relative molecular mass of 96.07626 and 79.95606 as a sulfation characteristic fragment. The compound was identified as naringenin glucuronyl sulfate.

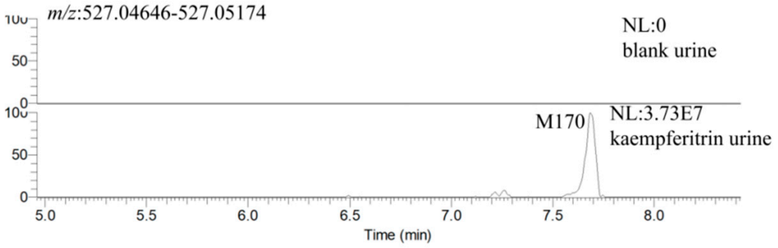

Figure S65 The EICs of **M170** in the blank and kaempferitrin urine samples in negative ion mode.

(4) Apigenin glucuronyl sulfates (M171–M177)

The EICs of **M171–M177** are presented in **Figure S66**. In the negative ion mode, the *m/z* 525.03345, *m/z* 525.03333, *m/z* 525.03351, *m/z* 525.03331, *m/z* 525.03321, *m/z* 525.03356 and *m/z* 525.03366 ($[M–H]^–$) were visible at the MS spectra, MF was predicted to be $C_{21}H_{18}O_{14}S$, and the MS^2 showed the fragment ions of *m/z* 445.0778, *m/z* 349.0024, *m/z* 269.0457, *m/z* 253.0511, *m/z* 187.0073, *m/z* 113.0246, and *m/z* 59.0138, of which the relative molecular mass of the difference between *m/z* 349.0024 and *m/z* 525.0334 was 176.0310, precisely calculated as a glucuronidated metabolite, and the relative mass of the difference between *m/z* 349.0022 and *m/z* 269.0457 molecular mass of 79.9565 was the characteristic fragment of sulfation, the compounds were identified as apigenin glucuronyl sulfates.

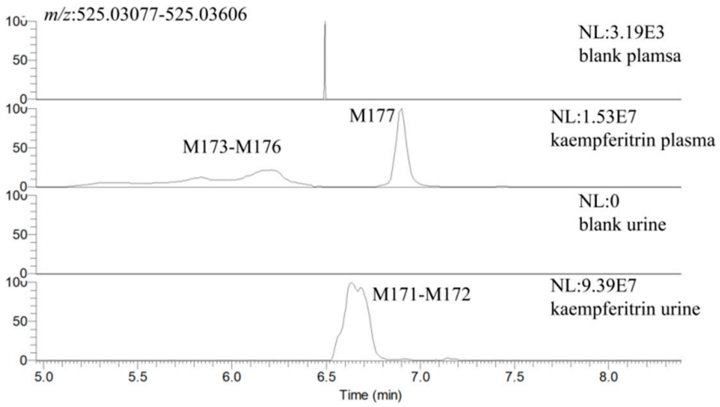

Figure S66 The EICs of **M171-M177** in the blank plasma, kaempferitrin plasma, blank urine, and kaempferitrin urine samples in negative ion mode.

(5) Trihydroxylated dihydrogenated flavone glucuronyl sulfates (M178–M183)

The EICs of **M178–M183** are presented in **Figure S67**. In the negative ion mode, the *m/z* 513.07007, *m/z* 513.07000, *m/z* 513.07007, *m/z* 513.06995, *m/z* 513.06909, and *m/z* 513.07007 ($[M–H]^–$) were visible at the MS spectra, MF was predicted to be $C_{21}H_{22}O_{13}S$, and the MS^2 visualized the fragment ions of *m/z* 433.1143, *m/z* 337.0390, *m/z* 257.0822, *m/z* 175.0247, *m/z* 207.9935, *m/z* 137.0246 ($C_7H_5O_3$, $^{0,3}A^–$), *m/z* 113.0245, *m/z* 79.9574, and *m/z* 96.9601, of which *m/z* 175.0248 was the glucuronidation characteristic fragment ion, *m/z* 79.9574 was the sulfation characteristic fragment ion, identified the compounds as trihydroxylated dihydrogenated flavone glucuronyl sulfates, $C_{21}H_{22}O_{13}S$ was input into Scifinder, where 5 compounds were found with the same general

formula, but there were no presumed compounds; hence, it was presumed that it may be a new compound. In the MS^2 spectrum of **M180**, the fragment ion of *m/z* 433.11032, *m/z* 337.03735, *m/z* 257.08240, *m/z* 175.02483, *m/z* 135.04526 ($C_8H_7O_2$, $^{0,2}A^-$), *m/z* 113.02457, *m/z* 79.95745, and *m/z* 96.96021 were observed, of which *m/z* 175.02483 was a glucuronidation characteristic fragment ion, *m/z* 79.95745 was a sulfation characteristic fragment ion, identified the compound as trihydroxylated dihydrogenated flavone glucuronyl sulfate, $C_{21}H_{22}O_{13}S$ was entered into Scifinder, where 5 compounds were found with the same general formula, but there were no presumed compounds, presumed that they may be new compounds.

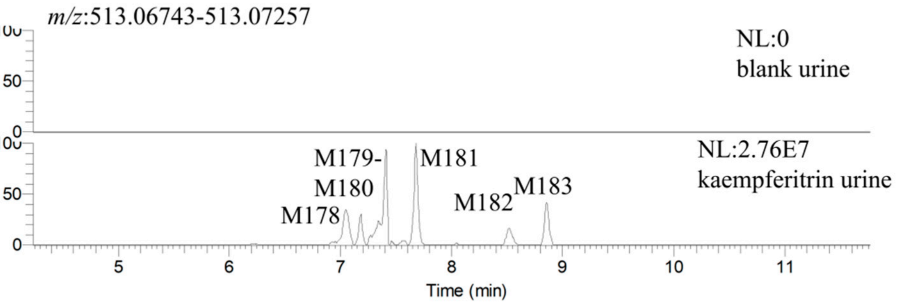

Figure S67 The EICs of **M178–M183** in the blank and kaempferitrin urine samples in negative ion mode.

(6) Dehydroxylated apigenin glucuronyl sulfates (M184–M186)

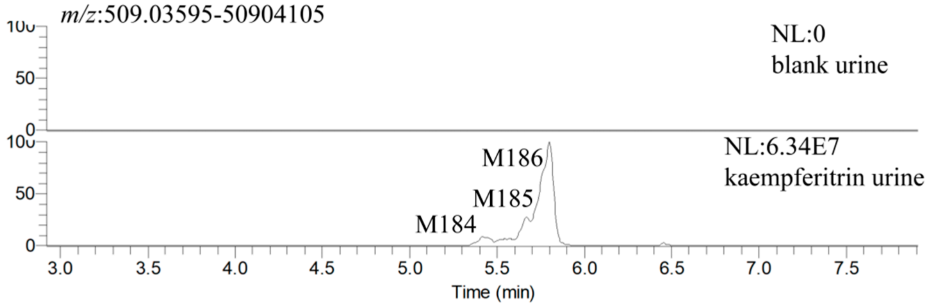

Figure S68 The EICs of **M184–M186** in the blank and kaempferitrin urine samples in negative ion mode.

(7) Dihydroxylated flavane glucuronyl sulfates (M187–M190)

The EICs of **M187–M190** are presented in **Figure S69**. In the negative ion mode, the *m/z*

497.07629, *m/z* 497.07455, *m/z* 497.07489, and *m/z* 497.07413 ($[M–H]^-$) were visible in the MS spectra, MF was predicted as $C_{21}H_{22}O_{12}S$, and the MS^2 visualized *m/z* 417.1195, *m/z* 321.0441, *m/z* 241.0872, *m/z* 175.0250, *m/z* 200.9863, *m/z* 121.0296, *m/z* 135.0454, *m/z* 119.0505, *m/z* 113.0245, and *m/z* 85.0295, of which *m/z* 175.0246 was a characteristic fragment ion for glucuronidation, *m/z* 321.0441, *m/z* 241.0871 difference relative molecular mass of 79.9570 was a characteristic fragment for sulfation, *m/z* 200.9863 was presumed that sulfation occurs in the B-ring, *m/z* 207.9943 was presumed to have occurred in the A-ring, removing SO_3 and $C_6H_8O_6$, with a reduction of 3 oxygen atoms and an increase of 4 hydrogen atoms in the MF compared to kaempferol, with a DBE of 9. It was presumed that all of the C-rings had been reduced and that the A- and C-rings were dehydroxylated, and the compounds were identified as dihydroxylated flavane glucuronyl sulfates. Entering $C_{21}H_{22}O_{12}S$ into Scifinder retrieved 8 compounds with the same formula, and the presumed compounds were not found; hence, it was presumed to be a new compound.

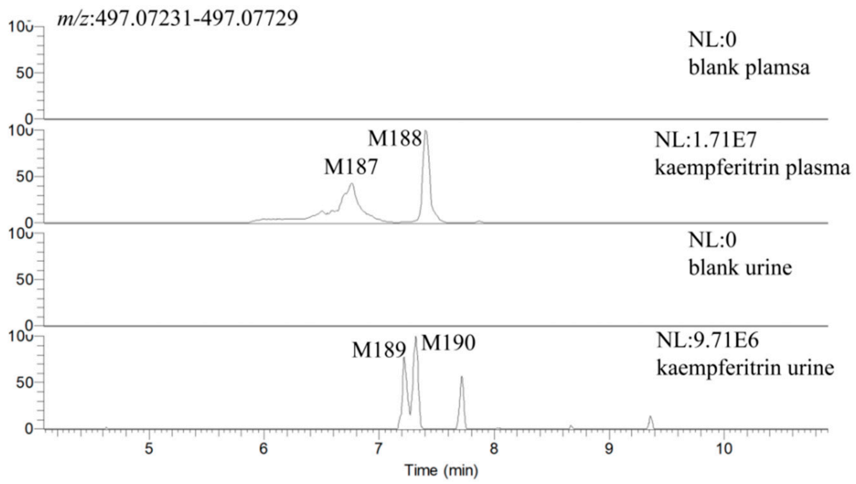

Figure S69 The EICs of **M187-M190** in the blank and kaempferitrin plasma samples in negative ion mode.

(8) Methylated dehydroxylated apiferol glucuronyl sulfate (M191)

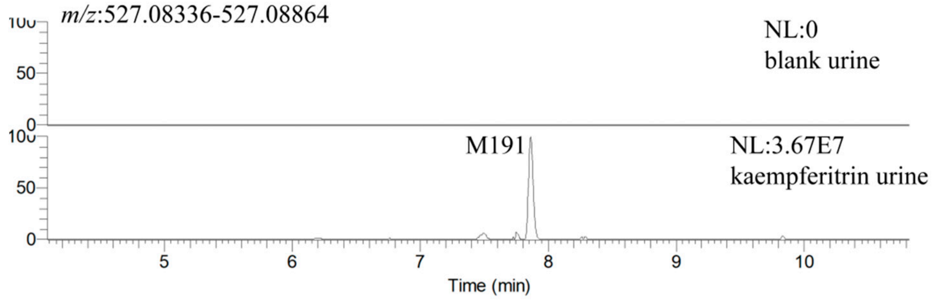

Figure S70 The EICs of **M191** in the blank and kaempferitrin urine samples in negative ion mode.

(9) Hydroxybenzyl alcohol glucuronyl sulfate (M192)

The EICs of **M192** are presented in **Figure S71**. In the negative ion mode, the *m/z* 379.03320 ($[M–H]^-$) was visible at the MS spectra, MF was predicted to be $C_{13}H_{16}O_{11}S$, and the MS^2 showed the fragment ions of *m/z* 299.07727, *m/z* 175.02490, *m/z* 203.00204, *m/z* 123.04527 ($C_7H_7O_2$, $^{0,3}A^-$), *m/z* 113.02454, *m/z* 96.96021, and *m/z* 79.95737, of which *m/z* 175.02490 was a glucuronidation fragmentation ion, *m/z* 96.96021, *m/z* 79.95737 were sulfate fragmentation ions. The compound was identified as hydroxybenzyl alcohol glucuronyl sulfate.

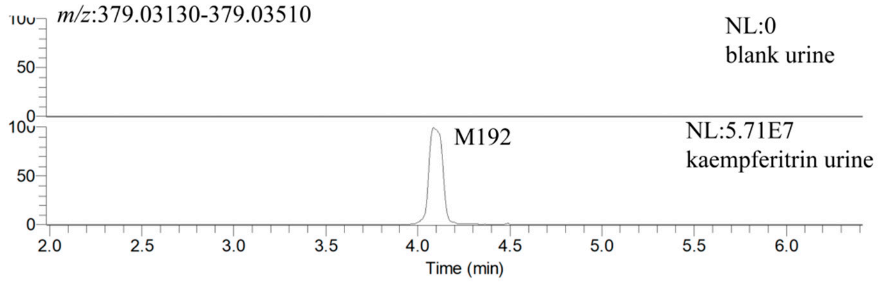

Figure S71 The EICs of **M192** in the blank and kaempferitrin urine samples in negative ion mode.

3. Network pharmacology of KAE and its metabolites

Table S9

Core target information of kaempferitrin (KAE) and its metabolites.

Name	Betweenness	Closeness	Degree
**AKT1**	553.3353	0.785714	56

**TNF**	496.8635	0.77	54
**EGFR**	285.5099	0.740385	50
**PTGS2**	451.7288	0.733333	49
**BCL2**	220.1149	0.719626	47
**MMP9**	150.9023	0.681416	43
**ESR1**	206.372	0.6875	42
**PPARG**	246.2191	0.6875	42
**SRC**	253.741	0.681416	41
**KDR**	58.75987	0.631148	34
**MMP2**	64.98004	0.620968	32
**IL2**	63.05775	0.616	31
**GSK3B**	74.74313	0.611111	30
**KIT**	40.14695	0.601563	29
**BRAF**	25.38442	0.592308	27
**SERPINE1**	80.38269	0.596899	27
**PIK3CA**	35.59544	0.587786	27
**MET**	42.61287	0.601563	26
**PIK3R1**	24.72778	0.557971	25
**PRKACA**	89.27113	0.592308	25
**MPO**	54.30838	0.578947	24

**AR**	24.0721	0.592308	24
**ABCG2**	94.93353	0.587786	23
**APP**	65.64438	0.578947	23
**PLG**	50.57792	0.578947	23
**ABCB**	47.77517	0.583333	22
**PPARA**	53.01554	0.583333	22
**TERT**	18.67318	0.553957	21

Table S10

Results of the biological process (BP) category terms from GO enrichment analysis.

No.	Name	Count	P Value	Genes
**1**	negative regulation of apoptotic process	12	2.60E-11	GSK3B, SRC, BCL2, KDR, AKT1, BRAF, PIK3R1, MPO, TNF, MMP9, EGFR, IL2
**2**	negative regulation of gene expression	10	1.90E-10	GSK3B, APP, TERT, PIK3CA, SRC, KDR, AKT1, PPARG, ESR1, TNF
**3**	positive regulation of peptidyl-serine phosphorylation	7	5.78E-10	APP, BCL2, AKT1, BRAF, PTGS2, TNF, EGFR
**4**	phosphorylation	11	5.54E-09	GSK3B, PIK3CA, SRC, KIT, KDR, AKT1, BRAF, PIK3R1, PRKACA, MET, EGFR
**5**	positive regulation of smooth muscle cell proliferation	6	9.79E-09	PIK3CA, AKT1, PIK3R1, PTGS2, TNF, EGFR
**6**	positive regulation of protein phosphorylation	7	2.97E-07	APP, KDR, AKT1, BRAF, TNF, MMP9, EGFR
**7**	positive regulation of vascular associated smooth muscle cell proliferation	5	7.77E-07	TERT, SRC, MMP2, TNF, MMP9
**8**	response to xenobiotic stimulus	7	1.01E-06	ABCB, SRC, MMP2,

				BCL2, PPARG, PTGS2, TNF
**9**	epidermal growth factor receptor signaling pathway	5	1.04E-06	PIK3CA, SRC, AKT1, BRAF, EGFR
**10**	positive regulation of miRNA transcription	5	1.29E-06	AR, TERT, PPARG, TNF, EGFR
**11**	protein autophosphorylation	6	1.62E-06	GSK3B, SRC, KIT, KDR, AKT1, EGFR
**12**	positive regulation of transcription by RNA polymerase II	11	3.27E-06	APP, AR, AKT1, PPARG, PIK3R1, PPARA, ESR1, TNF, MET, EGFR, IL2
**13**	positive regulation of phosphatidylinositol 3-kinase/protein kinase B signal transduction	6	5.61E-06	PIK3CA, SRC, KIT, KDR, TNF, EGFR
**14**	negative regulation of intrinsic apoptotic signaling pathway	4	6.54E-06	SRC, BCL2, AKT1, MMP9
**15**	negative regulation of miRNA transcription	4	7.31E-06	PPARG, PPARA, ESR1, TNF
**16**	positive regulation of DNA-binding transcription factor activity	5	7.79E-06	KIT, AKT1, PPARG, ESR1, TNF
**17**	cellular response to insulin stimulus	5	7.79E-06	PIK3CA, SRC, AKT1, PPARG, PIK3R1
**18**	insulin-like growth factor receptor signaling pathway	4	1.10E-05	AR, PIK3CA, AKT1, PIK3R1

**19**	positive regulation of ERK1 and ERK2 cascade	6	1.29E-05	APP, SRC, KDR, BRAF, TNF, EGFR
**20**	negative regulation of extrinsic apoptotic signaling pathway in absence of ligand	4	1.72E-05	TERT, BCL2, AKT1, TNF
**21**	negative regulation of endothelial cell apoptotic process	4	1.87E-05	TERT, SERPINE1, KDR, BRAF
**22**	ovarian follicle development	4	2.02E-05	MMP2, KIT, BCL2, KDR
**23**	signal transduction	10	2.47E-05	AR, SRC, KIT, AKT1, BRAF, PPARG, PIK3R1, ESR1, MET, EGFR
**24**	positive regulation of nitric oxide biosynthetic process	4	2.91E-05	AKT1, PTGS2, ESR1, TNF
**25**	cellular response to hypoxia	5	3.97E-05	TERT, SRC, BCL2, PPARG, PTGS2
**26**	epithelial cell proliferation	4	4.29E-05	AR, KIT, KDR, EGFR
**27**	negative regulation of signaling receptor activity	3	5.02E-05	PPARG, PPARA, TNF
**28**	negative regulation of neuron apoptotic process	5	5.04E-05	TERT, PIK3CA, BCL2, KDR, BRAF
**29**	cellular response to reactive oxygen species	4	5.11E-05	MMP2, AKT1, MMP9, EGFR
**30**	positive regulation of gene expression	7	5.34E-05	GSK3B, APP, AR, AKT1,

				BRAF, PPARG, TNF
**31**	response to mechanical stimulus	4	6.36E-05	SRC, MMP2, PPARG, MPO
**32**	positive regulation of protein localization to plasma membrane	4	6.36E-05	AKT1, PIK3R1, TNF, EGFR
**33**	positive regulation of apoptotic process	6	6.74E-05	SRC, BCL2, PPARG, PTGS2, TNF, MMP9
**34**	insulin receptor signaling pathway	4	1.23E-04	GSK3B, PIK3CA, AKT1, PIK3R1
**35**	protein phosphorylation	6	1.40E-04	GSK3B, APP, SRC, AKT1, BRAF, PRKACA
**36**	phosphatidylinositol 3-kinase/protein kinase B signal transduction	4	1.62E-04	PIK3CA, AKT1, PIK3R1, TNF
**37**	cellular response to fluid shear stress	3	1.62E-04	SRC, MMP2, PTGS2
**38**	multicellular organism development	4	1.81E-04	KIT, KDR, MET, EGFR
**39**	positive regulation of nitric-oxide synthase activity	3	1.87E-04	TERT, AKT1, ESR1
**40**	positive regulation of cell growth	4	2.32E-04	BCL2, AKT1, EGFR, IL2
**41**	astrocyte activation	3	2.72E-04	APP, TNF, EGFR
**42**	negative regulation of anoikis	3	3.04E-04	PIK3CA, SRC, BCL2
**43**	positive regulation of DNA-templated transcription	7	3.37E-04	AR, AKT1, PPARG, PPARA, ESR1, TNF, EGFR
**44**	angiogenesis	5	3.90E-04	PIK3CA, MMP2,

				SERPINE1, KDR, PTGS2
**45**	positive regulation of inflammatory response	4	4.47E-04	APP, SERPINE1, TNF, IL2
**46**	cell surface receptor protein tyrosine kinase signaling pathway	4	4.59E-04	SRC, KDR, MET, EGFR
**47**	male gonad development	4	4.71E-04	AR, KIT, BCL2, ESR1
**48**	positive regulation of cell migration	5	5.91E-04	MMP2, KIT, KDR, AKT1, EGFR
**49**	response to oxidative stress	4	6.20E-04	APP, AKT1, PTGS2, MPO
**50**	cellular response to cadmium ion	3	0.001043	AKT1, MMP9, EGFR
**51**	extrinsic apoptotic signaling pathway in absence of ligand	3	0.001104	GSK3B, BCL2, IL2
**52**	positive regulation of glucose import	3	0.001166	TERT, AKT1, PIK3R1
**53**	cytokine-mediated signaling pathway	4	0.00121	KIT, AKT1, PIK3R1, PRKACA
**54**	cellular response to estradiol stimulus	3	0.001229	MMP2, ESR1, EGFR
**55**	extrinsic apoptotic signaling pathway via death domain receptors	3	0.001362	BCL2, PIK3R1, TNF
**56**	T cell differentiation	3	0.00143	KIT, PIK3R1, IL2
**57**	negative regulation of cysteine-type endopeptidase activity involved in apoptotic process	3	0.001501	SRC, AKT1, TNF

**58**	T cell costimulation	3	0.001573	PIK3CA, SRC, AKT1
**59**	extracellular matrix organization	4	0.001659	APP, MMP2, TNF, MMP9
**60**	cellular response to amyloid-beta	3	0.001721	GSK3B, APP, TNF
**61**	embryo implantation	3	0.001721	MMP2, PTGS2, MMP9
**62**	extracellular matrix disassembly	3	0.001798	MMP2, PLG, MMP9
**63**	positive regulation of MAPK cascade	4	0.002103	AR, KIT, KDR, TNF
**64**	peptidyl-tyrosine phosphorylation	3	0.002121	SRC, KDR, EGFR
**65**	response to activity	3	0.002121	PIK3CA, MMP2, TNF
**66**	visual learning	3	0.002121	APP, KIT, BRAF
**67**	response to glucocorticoid	3	0.002121	BCL2, PTGS2, TNF
**68**	positive regulation of blood vessel endothelial cell migration	3	0.002121	KDR, AKT1, PLG
**69**	cellular response to lipopolysaccharide	4	0.0022	SRC, SERPINE1, TNF, MMP9
**70**	ephrin receptor signaling pathway	3	0.002205	SRC, MMP2, MMP9
**71**	intrinsic apoptotic signaling pathway in response to DNA damage	3	0.002205	BCL2, PIK3R1, TNF
**72**	positive regulation of G1/S transition of mitotic cell cycle	3	0.00238	TERT, AKT1, EGFR
**73**	positive regulation of MAP kinase activity	3	0.00256	KIT, TNF, EGFR
**74**	positive regulation of kinase activity	3	0.00256	KDR, MET, EGFR

**75**	response to estrogen	3	0.002653	MMP2, PPARG, ESR1
**76**	response to gold nanoparticle	2	0.002735	MPO, TNF
**77**	substrate adhesion-dependent cell spreading	3	0.003038	SRC, BRAF, PIK3R1
**78**	cell–cell adhesion	4	0.003076	SRC, KIT, BCL2, EGFR
**79**	negative regulation of autophagy	3	0.003139	BCL2, AKT1, MET
**80**	cellular response to amino acid stimulus	3	0.003449	MMP2, TNF, EGFR
**81**	glucose metabolic process	3	0.003556	PIK3CA, AKT1, TNF
**82**	regulation of circadian rhythm	3	0.003773	GSK3B, PPARG, PPARA
**83**	positive regulation of non-canonical NF-kappaB signal transduction	3	0.003773	APP, TNF, EGFR
**84**	positive regulation of tyrosine phosphorylation of STAT protein	3	0.003773	KIT, TNF, IL2
**85**	circadian rhythm	3	0.003884	GSK3B, TNF, EGFR
**86**	learning or memory	3	0.003884	APP, SRC, EGFR
**87**	transcription by RNA polymerase II	4	0.003949	AR, PIK3R1, ESR1, IL2
**88**	negative regulation of osteoblast differentiation	3	0.003997	GSK3B, PPARG, TNF
**89**	response to UV-A	2	0.004099	AKT1, EGFR
**90**	cellular response to retinoic acid	3	0.004111	GSK3B, PPARG, TNF
**91**	excitatory postsynaptic potential	3	0.004111	GSK3B, AKT1, MET

**92**	positive regulation of cell population proliferation	5	0.004694	AR, BCL2, KDR, EGFR, IL2
**93**	activation of cysteine-type endopeptidase activity involved in apoptotic process	3	0.005205	BCL2, PPARG, TNF
**94**	morphogenesis of an epithelial fold	2	0.005462	AR, EGFR
**95**	cell migration	4	0.006489	PIK3CA, KDR, MET, MMP9
**96**	positive regulation of fatty acid metabolic process	2	0.006823	PPARG, PPARA
**97**	positive regulation of protein transport	2	0.006823	SRC, TNF
**98**	positive regulation of fever generation	2	0.006823	PTGS2, TNF
**99**	interleukin-18-mediated signaling pathway	2	0.008182	AKT1, PIK3R1
**100**	response to fructose	2	0.008182	PTGS2, TNF
**101**	positive regulation of I-kappaB phosphorylation	2	0.008182	AKT1, TNF
**102**	xenobiotic transport across blood–brain barrier	2	0.008182	ABCB, ABCG2
**103**	lymphoid progenitor cell differentiation	2	0.008182	KIT, BCL2
**104**	negative regulation of sequestering of triglyceride	2	0.008182	PPARG, PPARA

**105**	negative regulation of leukocyte cell–cell adhesion	2	0.008182	AKT1, PPARA
**106**	negative regulation of mitochondrial depolarization	2	0.008182	SRC, BCL2
**107**	positive regulation of fatty acid oxidation	2	0.008182	PPARG, PPARA
**108**	response to ethanol	3	0.009527	PPARA, TNF, IL2
**109**	positive regulation of phosphate metabolic process	2	0.00954	KIT, KDR
**110**	myeloid progenitor cell differentiation	2	0.00954	KIT, BRAF
**111**	prostate gland epithelium morphogenesis	2	0.010895	AR, MMP2
**112**	negative regulation of vascular wound healing	2	0.010895	SERPINE1, TNF
**113**	positive regulation of glucose metabolic process	2	0.012249	SRC, AKT1
**114**	MAPK cascade	3	0.01279	AR, BRAF, EGFR
**115**	cell differentiation	5	0.012926	GSK3B, SRC, AKT1, PPARG, PPARA
**116**	negative regulation of developmental process	2	0.013601	GSK3B, KIT
**117**	regulation of neuroinflammatory response	2	0.013601	PTGS2, MMP9

**118**	positive regulation of podosome assembly	2	0.013601	SRC, TNF
**119**	tongue development	2	0.013601	KIT, EGFR
**120**	export across plasma membrane	2	0.013601	ABCB, ABCG2
**121**	transepithelial transport	2	0.013601	ABCB, ABCG2
**122**	negative regulation of lipid storage	2	0.013601	PPARG, TNF
**123**	negative regulation of cholesterol storage	2	0.014951	PPARG, PPARA
**124**	cellular response to UV-A	2	0.014951	MMP2, MMP9
**125**	endothelial cell apoptotic process	2	0.014951	BRAF, TNF
**126**	negative regulation of B cell apoptotic process	2	0.014951	BCL2, IL2
**127**	anoikis	2	0.0163	PIK3CA, AKT1
**128**	nuclear receptor-mediated steroid hormone signaling pathway	2	0.0163	AR, ESR1
**129**	negative regulation of inflammatory response	3	0.017125	PPARG, PPARA, IL2
**130**	negative regulation of fibrinolysis	2	0.017647	SERPINE1, PLG
**131**	positive regulation of protein modification process	2	0.017647	KIT, KDR
**132**	tissue remodeling	2	0.018992	MMP2, PLG
**133**	peptidyl-tyrosine autophosphorylation	2	0.018992	KDR, EGFR

**134**	positive regulation of cyclin-dependent protein serine/threonine kinase activity	2	0.018992	AKT1, EGFR
**135**	negative regulation of macrophage derived foam cell differentiation	2	0.018992	PPARG, PPARA
**136**	positive regulation of endothelial cell chemotaxis	2	0.018992	KDR, MET
**137**	peptidyl-serine phosphorylation	3	0.01938	GSK3B, AKT1, PRKACA
**138**	negative regulation of macroautophagy	2	0.020335	PIK3CA, AKT1
**139**	digestive tract morphogenesis	2	0.020335	BCL2, EGFR
**140**	intracellular signal transduction	4	0.020644	GSK3B, SRC, KIT, AKT1
**141**	positive regulation of angiogenesis	3	0.02079	TERT, SERPINE1, KDR
**142**	negative regulation of cell population proliferation	4	0.020895	APP, AR, PLG, PTGS2
**143**	response to amyloid-beta	2	0.021676	MMP2, MMP9
**144**	replicative senescence	2	0.021676	TERT, SERPINE1
**145**	embryonic hemopoiesis	2	0.023016	KIT, KDR
**146**	response to hypoxia	3	0.024241	MMP2, PPARA, TNF
**147**	cellular response to prostaglandin E stimulus	2	0.024354	AKT1, PPARG
**148**	uterus development	2	0.024354	SRC, ESR1

**149**	mammary gland alveolus development	2	0.024354	AR, ESR1
**150**	positive regulation of DNA binding	2	0.024354	PPARG, MMP9
**151**	vascular endothelial growth factor signaling pathway	2	0.02569	PIK3CA, KDR
**152**	positive regulation of lipid biosynthetic process	2	0.02569	AKT1, PPARA
**153**	negative regulation of cell migration	3	0.026841	GSK3B, SERPINE1, BCL2
**154**	positive regulation of bone resorption	2	0.027024	SRC, EGFR
**155**	fibrinolysis	2	0.027024	SERPINE1, PLG
**156**	steroid hormone receptor signaling pathway	2	0.027024	PPARA, ESR1
**157**	labyrinthine layer blood vessel development	2	0.027024	AKT1, PLG
**158**	positive regulation of glial cell proliferation	2	0.028357	TNF, EGFR
**159**	positive regulation of protein export from nucleus	2	0.028357	GSK3B, PRKACA
**160**	stress fiber assembly	2	0.028357	SRC, BRAF
**161**	cellular response to estrogen stimulus	2	0.028357	AR, ESR1
**162**	positive regulation of gluconeogenesis	2	0.028357	PPARA, PRKACA
**163**	cell migration involved in sprouting angiogenesis	2	0.028357	KDR, AKT1

**164**	positive regulation of smooth muscle cell migration	2	0.028357	SRC, BCL2
**165**	melanocyte differentiation	2	0.029687	KIT, BCL2
**166**	estrogen receptor signaling pathway	2	0.029687	AR, ESR1
**167**	positive regulation of protein localization to cell surface	2	0.029687	AKT1, TNF
**168**	regulation of protein localization to plasma membrane	2	0.031016	AR, PIK3R1
**169**	regulation of peptidyl-tyrosine phosphorylation	2	0.031016	APP, EGFR
**170**	regulation of multicellular organism growth	2	0.032344	APP, PIK3CA
**171**	response to cadmium ion	2	0.032344	TERT, KIT
**172**	response to food	2	0.033669	AKT1, MPO
**173**	positive regulation of phosphorylation	2	0.033669	AR, EGFR
**174**	heart development	3	0.033808	MMP2, PPARG, PPARA
**175**	positive regulation of protein metabolic process	2	0.034993	APP, AKT1
**176**	DNA biosynthetic process	2	0.037636	TERT, SRC
**177**	microglial cell activation	2	0.038954	APP, TNF
**178**	focal adhesion assembly	2	0.038954	SRC, BCL2
**179**	proteolysis	4	0.039065	APP, MMP2, PLG, MMP9

**180**	positive regulation of lamellipodium assembly	2	0.040271	PIK3CA, PIK3R1
**181**	negative regulation of extrinsic apoptotic signaling pathway via death domain receptors	2	0.040271	GSK3B, SERPINE1
**182**	negative regulation of transcription by RNA polymerase II	5	0.040453	AR, PPARG, PPARA, ESR1, TNF
**183**	vascular endothelial growth factor receptor signaling pathway	2	0.041586	SRC, KDR
**184**	negative regulation of mitotic cell cycle	2	0.041586	TNF, EGFR
**185**	response to electrical stimulus	2	0.041586	SRC, MMP2
**186**	liver regeneration	2	0.041586	TNF, EGFR
**187**	positive regulation of vasoconstriction	2	0.041586	PTGS2, EGFR
**188**	negative regulation of apoptotic signaling pathway	2	0.042899	BCL2, TNF
**189**	positive regulation of synaptic transmission, glutamatergic	2	0.044211	PTGS2, EGFR
**190**	cellular response to vascular endothelial growth factor stimulus	2	0.044211	KDR, AKT1
**191**	cellular response to nerve growth factor stimulus	2	0.045521	AKT1, BRAF
**192**	endodermal cell differentiation	2	0.045521	MMP2, MMP9

**193**	intracellular receptor signaling pathway	2	0.045521	AR, PPARA
**194**	negative regulation of stress fiber assembly	2	0.045521	PIK3R1, MET
**195**	positive regulation of cell migration involved in sprouting angiogenesis	2	0.048136	KDR, PTGS2
**196**	negative regulation of protein-containing complex assembly	2	0.04944	GSK3B, SRC
**197**	response to interleukin-1	2	0.04944	APP, SRC
**198**	regulation of neuron projection development	2	0.04944	GSK3B, AKT1
**199**	response to hydrogen peroxide	2	0.04944	MMP2, BCL2
**200**	positive regulation of calcium-mediated signaling	2	0.050743	APP, PRKACA
**201**	negative regulation of extrinsic apoptotic signaling pathway	2	0.052045	AR, SRC
**202**	positive regulation of protein localization to nucleus	2	0.053344	SRC, AKT1
**203**	T cell differentiation in thymus	2	0.053344	BCL2, BRAF
**204**	collagen catabolic process	2	0.054642	MMP2, MMP9
**205**	negative regulation of blood vessel endothelial cell migration	2	0.054642	PPARG, TNF
**206**	response to nicotine	2	0.055938	MMP2, BCL2

**207**	semaphorin-plexin signaling pathway	2	0.055938	KDR, MET
**208**	positive regulation of protein import into nucleus	2	0.055938	PIK3R1, PTGS2
**209**	positive regulation of stem cell proliferation	2	0.055938	TERT, KDR
**210**	positive regulation of receptor signaling pathway via JAK-STAT	2	0.057232	KIT, TNF
**211**	positive regulation of chemokine production	2	0.057232	APP, TNF
**212**	vasodilation	2	0.059816	TNF, EGFR
**213**	cellular response to epidermal growth factor stimulus	2	0.059816	AKT1, EGFR
**214**	response to nutrient levels	2	0.059816	SRC, TNF
**215**	hormone-mediated signaling pathway	2	0.059816	PPARG, PPARA
**216**	osteoclast differentiation	2	0.059816	PIK3R1, TNF
**217**	positive regulation of B cell proliferation	2	0.061105	BCL2, IL2
**218**	stem cell differentiation	2	0.061105	KIT, ESR1
**219**	positive regulation of protein binding	2	0.062393	GSK3B, TERT
**220**	thymus development	2	0.063679	BCL2, BRAF
**221**	stem cell proliferation	2	0.064963	ABCB, KDR
**222**	forebrain development	2	0.066246	APP, SRC

**223**	negative regulation of endopeptidase activity	2	0.067526	SERPINE1, AKT1
**224**	positive regulation of Notch signaling pathway	2	0.067526	SRC, KIT
**225**	G2/M transition of mitotic cell cycle	2	0.067526	APP, ABCB
**226**	phosphatidylinositol phosphate biosynthetic process	2	0.068806	PIK3CA, PIK3R1
**227**	positive regulation of protein-containing complex assembly	2	0.068806	GSK3B, TNF
**228**	extrinsic apoptotic signaling pathway	2	0.070083	GSK3B, TNF
**229**	positive regulation of fibroblast proliferation	2	0.071359	ESR1, EGFR
**230**	somatic stem cell population maintenance	2	0.072633	KIT, BRAF
**231**	cognition	2	0.072633	APP, TNF
**232**	peptidyl-threonine phosphorylation	2	0.072633	GSK3B, AKT1
**233**	positive regulation of TORC1 signaling	2	0.073905	SRC, AKT1
**234**	positive regulation of fat cell differentiation	2	0.073905	AKT1, PPARG
**235**	cell fate commitment	2	0.075176	KDR, PPARG
**236**	learning	2	0.077712	APP, PTGS2

**237**	cellular response to heat	2	0.078978	PRKACA, PTGS2
**238**	positive regulation of cell differentiation	2	0.078978	GSK3B, AR
**239**	response to nutrient	2	0.078978	PPARG, PPARA
**240**	platelet activation	2	0.082765	PIK3CA, SRC
**241**	humoral immune response	2	0.082765	BCL2, TNF
**242**	positive regulation of interleukin-8 production	2	0.084024	SERPINE1, TNF
**243**	positive regulation of neuron apoptotic process	2	0.085281	GSK3B, TNF
**244**	positive regulation of interleukin-1 beta production	2	0.085281	APP, TNF
**245**	positive regulation of endothelial cell migration	2	0.086537	KDR, AKT1
**246**	cellular response to xenobiotic stimulus	2	0.089043	BRAF, EGFR
**247**	positive regulation of cytokine production	2	0.089043	SRC, TNF
**248**	phagocytosis	2	0.09279	PIK3CA, MET
**249**	positive regulation of endothelial cell proliferation	2	0.094036	KDR, AKT1

Table S11

Results of the cellular component (CC) category terms from GO enrichment analysis.

NO.	Name	Count	PValue	Genes
**1**	receptor complex	7	3.58E-07	APP, SRC, KIT, KDR, PPARG, MET, EGFR
**2**	plasma membrane	21	4.00E-07	GSK3B, APP, ABCB, SRC, MMP2, SERPINE1, BRAF, PLG, PIK3R1, ESR1, TNF, EGFR, AR, TERT, PIK3CA, KIT, KDR, AKT1, PRKACA, MET, ABCG2
**3**	membrane raft	6	6.76E-06	APP, SRC, KDR, TNF, EGFR, ABCG2
**4**	cell surface	7	1.59E-04	APP, ABCB, KIT, PLG, TNF, MET, EGFR
**5**	perinuclear region of cytoplasm	7	3.52E-04	APP, PIK3CA, SRC, PPARG, PIK3R1, PRKACA, EGFR
**6**	extracellular space	10	7.10E-04	APP, MMP2, KIT, SERPINE1, PLG, MPO, TNF, MMP9, EGFR, IL2
**7**	extracellular region	10	9.02E-04	APP, MMP2, SERPINE1, KDR, PLG, MPO, TNF, MET, MMP9, IL2
**8**	protein-containing complex	6	0.001646	AR, BCL2, AKT1, PTGS2, ESR1, EGFR
**9**	cell–cell junction	4	0.001929	APP, KIT, AKT1, PIK3R1
**10**	cytoplasm	16	0.001982	GSK3B, APP, ABCB, SRC, BRAF, PIK3R1, PTGS2, ESR1, EGFR, AR, TERT, PIK3CA, BCL2, AKT1, PPARG,

				PRKACA
**11**	glutamatergic synapse	5	0.002172	GSK3B, SRC, AKT1, PLG, PRKACA
**12**	platelet alpha granule lumen	3	0.003478	APP, SERPINE1, PLG
**13**	nucleus	16	0.004232	GSK3B, APP, MMP2, BRAF, PIK3R1, MPO, ESR1, EGFR, AR, TERT, KDR, BCL2, AKT1, PPARG, PPARA, PRKACA
**14**	external side of apical plasma membrane	2	0.007732	ABCB, ABCG2
**15**	mitochondrion	7	0.010362	GSK3B, SRC, MMP2, BCL2, AKT1, BRAF, PRKACA
**16**	phosphatidylinositol 3-kinase complex, class IA	2	0.011576	PIK3CA, PIK3R1
**17**	postsynapse	3	0.014606	GSK3B, AKT1, MET
**18**	collagen-containing extracellular matrix	4	0.015288	MMP2, SERPINE1, PLG, MMP9
**19**	external side of plasma membrane	4	0.017462	KIT, KDR, PLG, TNF
**20**	extracellular exosome	8	0.021708	APP, ABCB, SRC, SERPINE1, PLG, PRKACA, MPO, MMP9
**21**	nucleoplasm	11	0.02404	GSK3B, AR, TERT, SRC, AKT1, PPARG, PPARA, PRKACA, MPO, ESR1, ABCG2
**22**	cell junction	3	0.024164	SRC, KDR, EGFR

**23**	phosphatidylinositol 3-kinase complex	2	0.029328	PIK3CA, PIK3R1
**24**	cytosol	13	0.03881	GSK3B, APP, SRC, BRAF, PIK3R1, ESR1, AR, TERT, PIK3CA, BCL2, AKT1, PPARG, PRKACA
**25**	endosome	3	0.068497	APP, KDR, EGFR
**26**	basal plasma membrane	2	0.089072	MET, EGFR
**27**	caveola	2	0.092612	SRC, PTGS2
**28**	membrane	11	0.094105	APP, AR, ABCB, KIT, BCL2, AKT1, PIK3R1, PRKACA, ESR1, MET, EGFR
**29**	apical plasma membrane	3	0.097855	ABCB, EGFR, ABCG2

Table S12

Results of the molecular function (MF) category terms from GO enrichment analysis.

No.	Name	Count	PValue	Genes
**1**	enzyme binding	9	2.84E-08	APP, AR, SRC, AKT1, PLG, PPARG, PTGS2, ESR1, EGFR
**2**	protein tyrosine kinase activity	6	3.03E-07	SRC, KIT, KDR, BRAF, MET, EGFR
**3**	transcription coactivator binding	5	5.66E-07	AR, TERT, PPARG, PPARA, ESR1
**4**	protease binding	6	5.68E-07	GSK3B, KIT, SERPINE1, BCL2, PLG, TNF
**5**	nuclear receptor activity	5	9.15E-07	AR, PPARG, PIK3R1, PPARA, ESR1
**6**	identical protein binding	13	1.31E-06	APP, BRAF, ESR1, TNF, MMP9, EGFR, TERT, KDR, BCL2, AKT1, PPARG, MET, ABCG2
**7**	ATP binding	12	3.18E-06	GSK3B, ABCB, PIK3CA, SRC, KIT, KDR, AKT1, BRAF, PRKACA, MET, EGFR, ABCG2
**8**	protein binding	28	1.37E-05	APP, GSK3B, ABCB, SRC, SERPINE1, PLG, PIK3R1, PTGS2, MPO, TNF, EGFR, TERT, KDR, AKT1, PRKACA, MMP2, BRAF, MMP9, ESR1, IL2, AR, PIK3CA, KIT, BCL2, PPARG, PPARA, MET, ABCG2

**9**	transmembrane receptor protein tyrosine kinase activity	4	2.16E-05	SRC, KIT, KDR, EGFR
**10**	protein kinase activity	6	3.66E-05	GSK3B, SRC, AKT1, BRAF, PRKACA, MET
**11**	kinase activity	5	1.82E-04	GSK3B, PIK3CA, AKT1, PIK3R1, EGFR
**12**	ATPase binding	4	2.23E-04	AR, SRC, ESR1, EGFR
**13**	protein phosphatase binding	4	2.56E-04	PPARG, PIK3R1, MET, EGFR
**14**	ubiquitin protein ligase binding	5	8.65E-04	GSK3B, ABCB, BCL2, PRKACA, EGFR
**15**	protein serine/threonine/tyrosine kinase activity	3	9.11E-04	GSK3B, AKT1, PRKACA
**16**	growth factor binding	3	0.001352	SRC, KIT, KDR
**17**	nuclear estrogen receptor binding	3	0.001567	SRC, PPARG, ESR1
**18**	protein serine kinase activity	5	0.00158	GSK3B, PIK3CA, AKT1, BRAF, PRKACA
**19**	signaling receptor binding	5	0.001628	APP, AR, SRC, SERPINE1, PLG
**20**	protein serine/threonine kinase activity	5	0.001848	GSK3B, PIK3CA, AKT1, BRAF, PRKACA
**21**	protein homodimerization activity	6	0.003462	TERT, KIT, BCL2, AKT1, PTGS2, ABCG2

**22**	DNA binding	7	0.004562	APP, AR, TERT, PPARG, PIK3R1, PPARA, ESR1
**23**	endopeptidase activity	3	0.004909	MMP2, PLG, MMP9
**24**	protein kinase binding	5	0.004933	GSK3B, AKT1, PRKACA, ESR1, EGFR
**25**	chromatin binding	5	0.005699	AR, PPARG, MPO, ESR1, EGFR
**26**	nitric-oxide synthase regulator activity	2	0.006975	AKT1, ESR1
**27**	sequence-specific DNA binding	4	0.007166	BCL2, PPARG, PPARA, ESR1
**28**	zinc ion binding	6	0.007337	AR, MMP2, PPARG, PPARA, ESR1, MMP9
**29**	beta-catenin binding	3	0.010292	GSK3B, AR, ESR1
**30**	kinase activator activity	2	0.015282	PIK3R1, IL2
**31**	estrogen response element binding	2	0.01666	AR, ESR1
**32**	insulin receptor substrate binding	2	0.018037	PIK3CA, PIK3R1
**33**	ABC-type xenobiotic transporter activity	2	0.019411	ABCB, ABCG2
**34**	efflux transmembrane transporter activity	2	0.019411	ABCB, ABCG2
**35**	heme binding	3	0.019715	SRC, PTGS2, MPO

**36**	integrin binding	3	0.021414	SRC, KDR, EGFR
**37**	DNA-binding transcription factor binding	3	0.023942	BCL2, PPARG, PPARA
**38**	RNA polymerase II-specific DNA-binding transcription factor binding	3	0.027131	GSK3B, AR, PPARA
**39**	MAP kinase kinase kinase activity	2	0.02898	BRAF, EGFR
**40**	insulin receptor binding	2	0.030339	SRC, PIK3R1
**41**	DNA-binding transcription activator activity, RNA polymerase II-specific	4	0.030746	AR, PPARG, PPARA, ESR1
**42**	serine-type endopeptidase activity	3	0.031056	MMP2, PLG, MMP9
**43**	nuclear steroid receptor activity	2	0.031697	PPARA, ESR1
**44**	xenobiotic transmembrane transporter activity	2	0.034407	ABCB, ABCG2
**45**	calmodulin binding	3	0.036106	AKT1, ESR1, EGFR
**46**	protein domain specific binding	3	0.03672	PLG, PPARA, PRKACA
**47**	DNA-binding transcription factor activity	4	0.037998	AR, PPARG, PPARA, ESR1

**48**	steroid binding	2	0.042494	AR, ESR1
**49**	peroxidase activity	2	0.042494	PTGS2, MPO
**50**	14-3-3 protein binding	2	0.043835	AKT1, ESR1
**51**	transcription cis-regulatory region binding	3	0.045397	AR, PPARG, TNF
**52**	ATPase-coupled transmembrane transporter activity	2	0.051846	ABCB, ABCG2
**53**	transcription coregulator binding	2	0.051846	PPARG, ESR1
**54**	SH2 domain binding	2	0.05715	SRC, KIT
**55**	cadherin binding	3	0.079326	SRC, KDR, EGFR
**56**	metallopeptidase activity	2	0.083246	MMP2, MMP9
**57**	scaffold protein binding	2	0.093488	SRC, BRAF

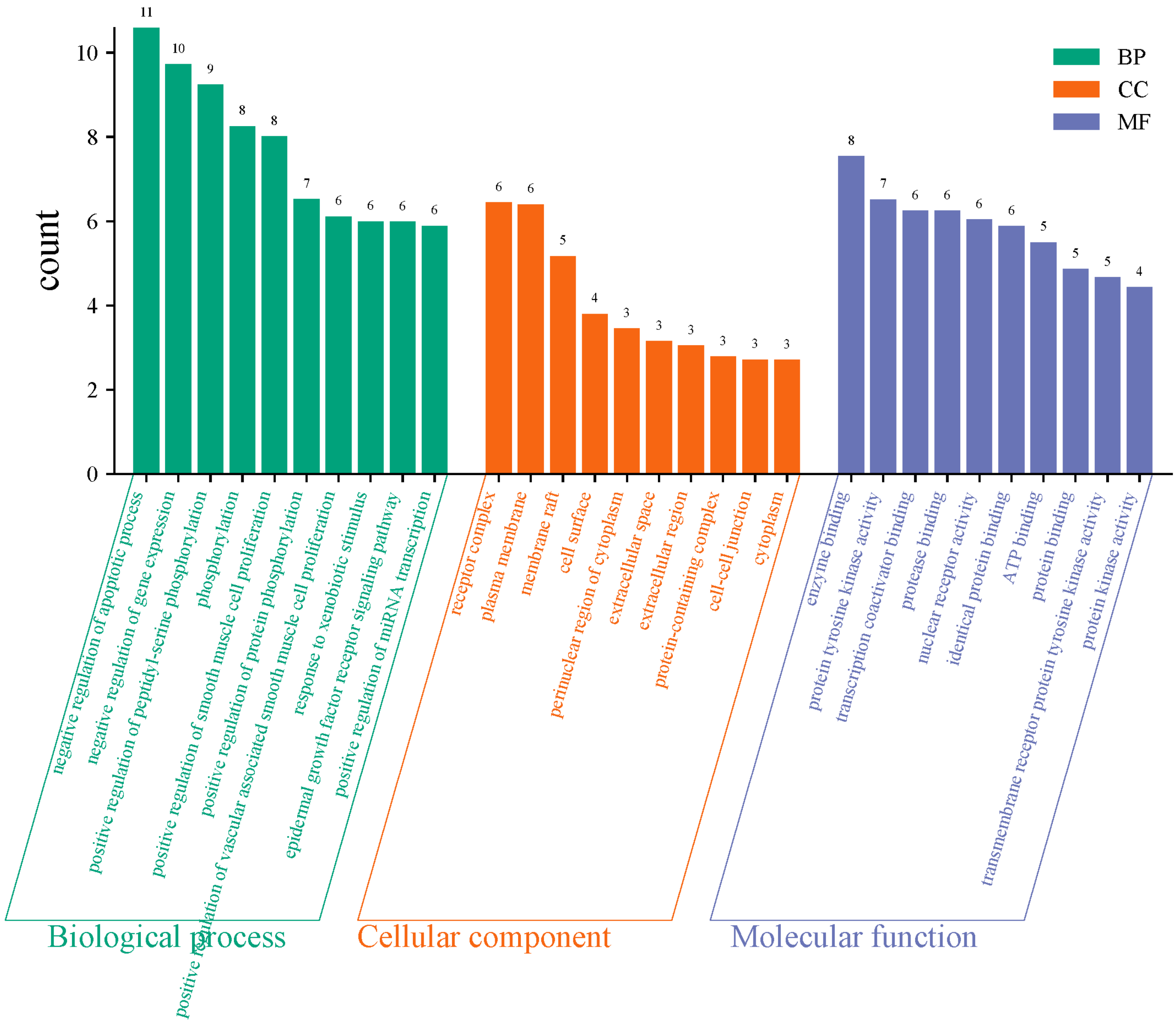

Figure S72 Results of GO enrichment analysis. (GO: Gene Ontology).

Table S13

Results of the pathways from KEGG enrichment analysis.

No.	Pathway ID	Pathway name	Count	PValue	FDR
**1**	hsa04151	PI3K-Akt signaling pathway	10	7.14E-07	2.89E-06
**2**	hsa05213	Endometrial cancer	6	8.04E-07	3.12E-06
**3**	hsa04370	VEGF signaling pathway	6	8.75E-07	3.26E-06
**4**	hsa04919	Thyroid hormone signaling pathway	7	1.43E-06	5.10E-06
**5**	hsa05221	Acute myeloid leukemia	6	1.64E-06	5.66E-06
**6**	hsa05230	Central carbon metabolism in cancer	6	2.04E-06	6.54E-06
**7**	hsa04917	Prolactin signaling pathway	6	2.04E-06	6.54E-06
**8**	hsa05218	Melanoma	6	2.34E-06	7.03E-06
**9**	hsa05223	Non-small cell lung cancer	6	2.34E-06	7.03E-06
**10**	hsa05135	Yersinia infection	7	2.93E-06	8.53E-06
**11**	hsa05165	Human papillomavirus infection	9	4.42E-06	1.25E-05
**12**	hsa04014	Ras signaling pathway	8	5.26E-06	1.44E-05
**13**	hsa04932	Non-alcoholic fatty liver disease	7	6.20E-06	1.65E-05
**14**	hsa05219	Bladder cancer	5	6.47E-06	1.67E-05
**15**	hsa05131	Shigellosis	8	7.09E-06	1.78E-05
**16**	hsa05142	Chagas disease	6	1.28E-05	3.14E-05
**17**	hsa04931	Insulin resistance	6	1.69E-05	3.93E-05

**18**	hsa04066	HIF-1 signaling pathway	6	1.69E-05	3.93E-05
**19**	hsa04062	Chemokine signaling pathway	7	2.02E-05	4.59E-05
**20**	hsa04010	MAPK signaling pathway	8	2.39E-05	5.30E-05
**21**	hsa04668	TNF signaling pathway	6	2.59E-05	5.60E-05
**22**	hsa04722	Neurotrophin signaling pathway	6	2.70E-05	5.70E-05
**23**	hsa04923	Regulation of lipolysis in adipocytes	5	2.80E-05	5.76E-05
**24**	hsa05415	Diabetic cardiomyopathy	7	2.85E-05	5.76E-05
**25**	hsa04660	T cell receptor signaling pathway	6	2.92E-05	5.78E-05
**26**	hsa05206	MicroRNAs in cancer	8	3.08E-05	5.97E-05
**27**	hsa05166	Human T-cell leukemia virus 1 infection	7	4.57E-05	8.68E-05
**28**	hsa05208	Chemical carcinogenesis - reactive oxygen species	7	4.93E-05	9.17E-05
**29**	hsa04910	Insulin signaling pathway	6	5.28E-05	9.63E-05
**30**	hsa05162	Measles	6	5.46E-05	9.68E-05
**31**	hsa05211	Renal cell carcinoma	5	5.51E-05	9.68E-05
**32**	hsa05214	Glioma	5	7.62E-05	1.31E-04
**33**	hsa05212	Pancreatic cancer	5	8.02E-05	1.36E-04
**34**	hsa04150	mTOR signaling pathway	6	1.01E-04	1.67E-04
**35**	hsa05010	Alzheimer disease	8	1.29E-04	2.11E-04
**36**	hsa04630	JAK-STAT signaling pathway	6	1.34E-04	2.15E-04

**37**	hsa04211	Longevity regulating pathway	5	1.47E-04	2.32E-04
**38**	hsa05222	Small cell lung cancer	5	1.67E-04	2.59E-04
**39**	hsa05167	Kaposi sarcoma-associated herpesvirus infection	6	2.76E-04	4.21E-04
**40**	hsa04914	Progesterone-mediated oocyte maturation	5	3.31E-04	4.96E-04
**41**	hsa04725	Cholinergic synapse	5	3.79E-04	5.59E-04
**42**	hsa04935	Growth hormone synthesis, secretion and action	5	4.74E-04	6.78E-04
**43**	hsa04071	Sphingolipid signaling pathway	5	4.74E-04	6.78E-04
**44**	hsa04611	Platelet activation	5	5.20E-04	7.32E-04
**45**	hsa04024	cAMP signaling pathway	6	5.33E-04	7.39E-04
**46**	hsa04810	Regulation of actin cytoskeleton	6	5.77E-04	7.89E-04
**47**	hsa04068	FoxO signaling pathway	5	6.39E-04	8.61E-04
**48**	hsa04210	Apoptosis	5	7.15E-04	9.49E-04
**49**	hsa04380	Osteoclast differentiation	5	8.40E-04	0.001101
**50**	hsa04213	Longevity regulating pathway - multiple species	4	8.52E-04	0.001101
**51**	hsa04072	Phospholipase D signaling pathway	5	0.001006	0.001282
**52**	hsa04664	Fc epsilon RI signaling pathway	4	0.001164	0.001463
**53**	hsa01524	Platinum drug resistance	4	0.001482	0.001838

**54**	hsa05220	Chronic myeloid leukemia	4	0.001599	0.001938
**55**	hsa04140	Autophagy - animal	5	0.001605	0.001938
**56**	hsa05100	Bacterial invasion of epithelial cells	4	0.00166	0.001979
**57**	hsa05164	Influenza A	5	0.001712	0.002015
**58**	hsa04360	Axon guidance	5	0.002192	0.002548
**59**	hsa05235	PD-L1 expression and PD-1 checkpoint pathway in cancer	4	0.002501	0.002872
**60**	hsa04613	Neutrophil extracellular trap formation	5	0.002559	0.002892
**61**	hsa04662	B cell receptor signaling pathway	4	0.002581	0.002892
**62**	hsa04912	GnRH signaling pathway	4	0.002746	0.00304
**63**	hsa04657	IL-17 signaling pathway	4	0.002917	0.003192
**64**	hsa05169	Epstein–Barr virus infection	5	0.003131	0.003385
**65**	hsa05231	Choline metabolism in cancer	4	0.003279	0.003465
**66**	hsa04750	Inflammatory mediator regulation of TRP channels	4	0.003279	0.003465
**67**	hsa05146	Amoebiasis	4	0.003667	0.003832
**68**	hsa05170	Human immunodeficiency virus 1 infection	5	0.003721	0.003846
**69**	hsa04620	Toll-like receptor signaling pathway	4	0.004301	0.004395
**70**	hsa04726	Serotonergic synapse	4	0.004997	0.004997
**71**	hsa04928	Parathyroid hormone synthesis,	4	0.004997	0.004997

		secretion and action			
**72**	hsa04670	Leukocyte transendothelial migration	4	0.005119	0.005119
**73**	hsa05171	Coronavirus disease - COVID-19	5	0.005523	0.005523
**74**	hsa04152	AMPK signaling pathway	4	0.005891	0.005891
**75**	hsa04650	Natural killer cell mediated cytotoxicity	4	0.007024	0.007024
**76**	hsa04930	Type II diabetes mellitus	3	0.008924	0.008924
**77**	hsa04936	Alcoholic liver disease	4	0.0093	0.0093
**78**	hsa04550	Signaling pathways regulating pluripotency of stem cells	4	0.0093	0.0093
**79**	hsa05020	Prion disease	5	0.009494	0.009494
**80**	hsa04973	Carbohydrate digestion and absorption	3	0.010844	0.010844
**81**	hsa04921	Oxytocin signaling pathway	4	0.011163	0.011163
**82**	hsa04934	Cushing syndrome	4	0.01136	0.01136
**83**	hsa04218	Cellular senescence	4	0.011761	0.011761
**84**	hsa04340	Hedgehog signaling pathway	3	0.0125	0.0125
**85**	hsa05022	Pathways of neurodegeneration - multiple diseases	6	0.014176	0.014176
**86**	hsa04929	GnRH secretion	3	0.016602	0.016602
**87**	hsa05152	Tuberculosis	4	0.016961	0.016961
**88**	hsa04920	Adipocytokine signaling pathway	3	0.019096	0.019096
**89**	hsa05168	Herpes simplex virus 1 infection	6	0.019284	0.019284

**90**	hsa05120	Epithelial cell signaling in Helicobacter pylori infection	3	0.019613	0.019613
**91**	hsa05202	Transcriptional misregulation in cancer	4	0.020389	0.020389
**92**	hsa05203	Viral carcinogenesis	4	0.023871	0.023871
**93**	hsa04976	Bile secretion	3	0.03051	0.03051
**94**	hsa04540	Gap junction	3	0.03177	0.03177
**95**	hsa04520	Adherens junction	3	0.032408	0.032408
**96**	hsa04666	Fc gamma R-mediated phagocytosis	3	0.035673	0.035673
**97**	hsa04916	Melanogenesis	3	0.037691	0.037691
**98**	hsa05132	Salmonella infection	4	0.04005	0.04005
**99**	hsa04064	NF-kappa B signaling pathway	3	0.04045	0.04045
**100**	hsa04020	Calcium signaling pathway	4	0.04126	0.04126
**101**	hsa04922	Glucagon signaling pathway	3	0.041857	0.041857
**102**	hsa05145	Toxoplasmosis	3	0.044727	0.044727
**103**	hsa04728	Dopaminergic synapse	3	0.060928	0.060928
**104**	hsa04371	Apelin signaling pathway	3	0.067562	0.067562
**105**	hsa05017	Spinocerebellar ataxia	3	0.070966	0.070966
**106**	hsa04261	Adrenergic signaling in cardiomyocytes	3	0.07972	0.07972
**107**	hsa01523	Antifolate resistance	2	0.087822	0.087822

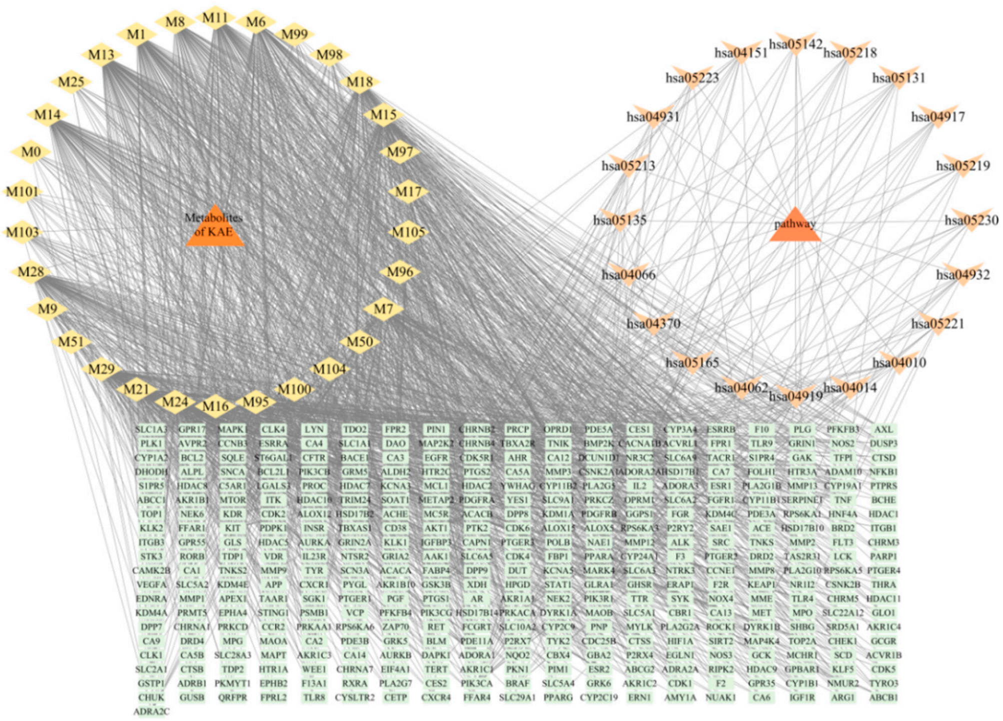

Figure S73 The compound-target-pathway (C-T-P) network (Green nodes represent the targets; orange nodes represent the pathways, yellow nodes represent the compounds).

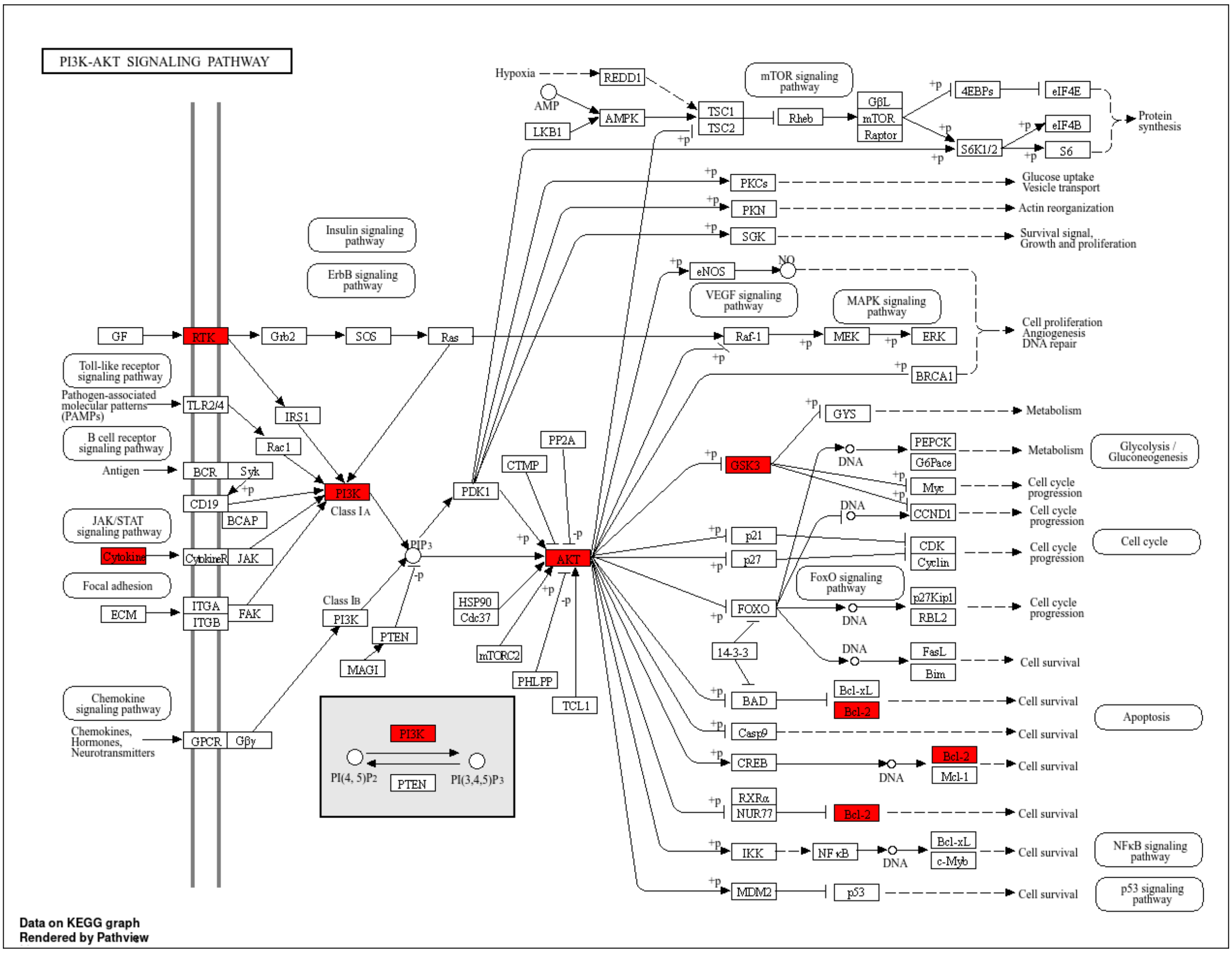

Figure S74 Distribution of key targets in the PI3K/AKT signaling pathway. (Red rectangle stands for the key targets).

4. Molecular docking validation analysis

Table S14

Details of each ligand–protein interaction molecular docking results.

Interacting group	Amino acid residue	Distance	Interaction type
AKT1-M96	LYS179	3.1	Hydrogen Bond
	GLU228	2.8	Hydrogen Bond
	ALA230	2.7	Hydrogen Bond
	GLU278	3.3	Hydrogen Bond
	THR291	2.9	Hydrogen Bond
	ASP292	2.5	Hydrogen Bond
SRC-M100	LYS295	4.5	Pi-cation
	GLU310	2.5	Hydrogen Bond
	MET341	2.8, 3.3, 2.8	Hydrogen Bond
	SER345	3.2	Hydrogen Bond
	ASP348	2.5, 2.5, 2.6	Hydrogen Bond
	ASH404	2.9	Hydrogen Bond
ESR1-M13	GLU353	2.5	Hydrogen Bond

	ARG394	3.2	Hydrogen Bond
	PHE404	5.1	Pi-Pi
	GLY521	2.7	Hydrogen Bond
	HIS524	3.1	Hydrogen Bond
MMP9-M58	GLN227	3.0	Hydrogen Bond
	PRO246	2.7	Hydrogen Bond
	ZN301		Salt bridge
EGFR-M16	LEU718	2.7	Hydrogen Bond
	LYS745	2.8	Hydrogen Bond
	GLN791	2.7	Hydrogen Bond
	ASN842	2.8	Hydrogen Bond
	ASP855	2.9	Hydrogen Bond

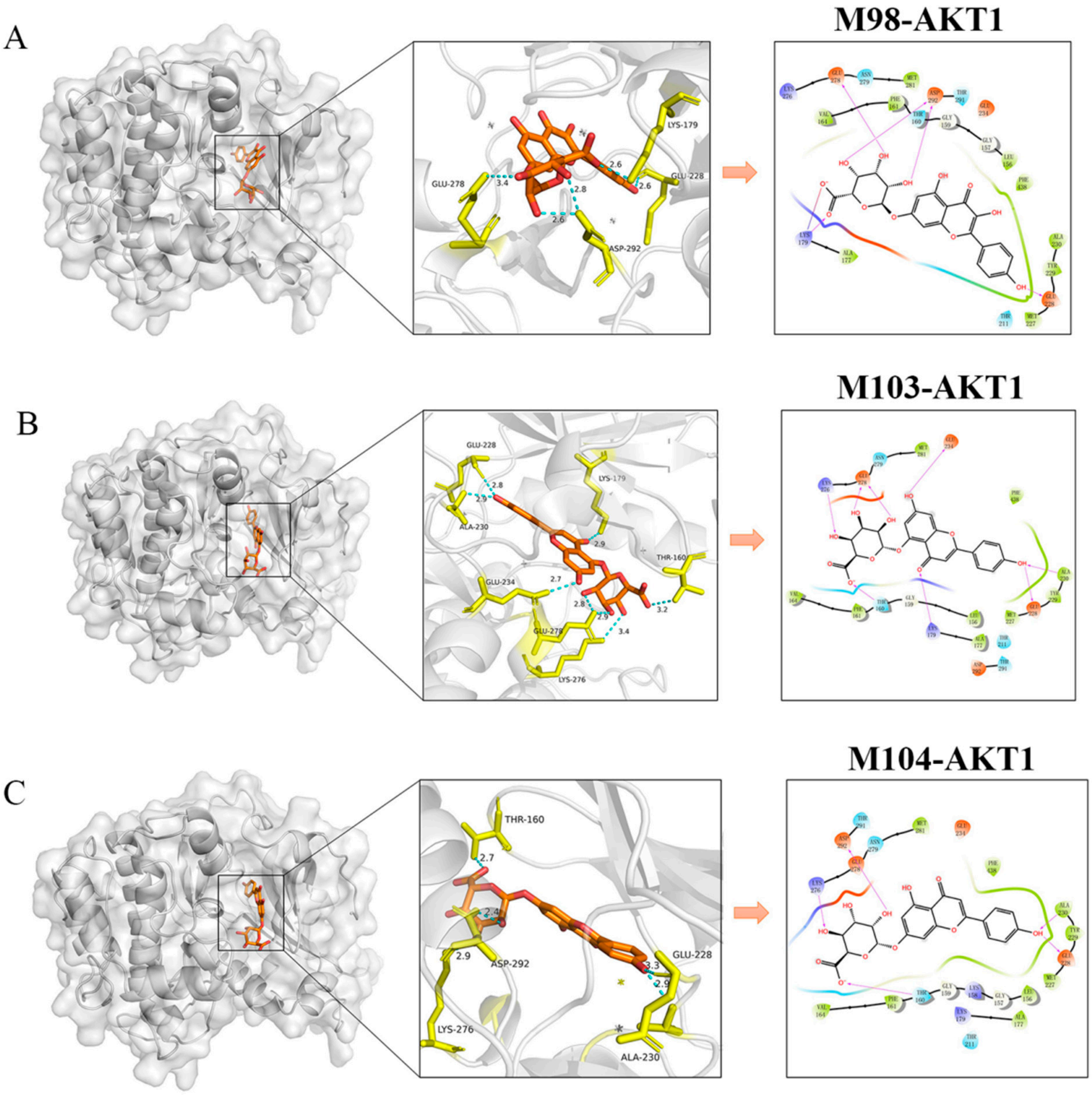

Figure S75 Molecular docking results of **M98** (A), **M103** (B), and **M104** (C) with AKT1.

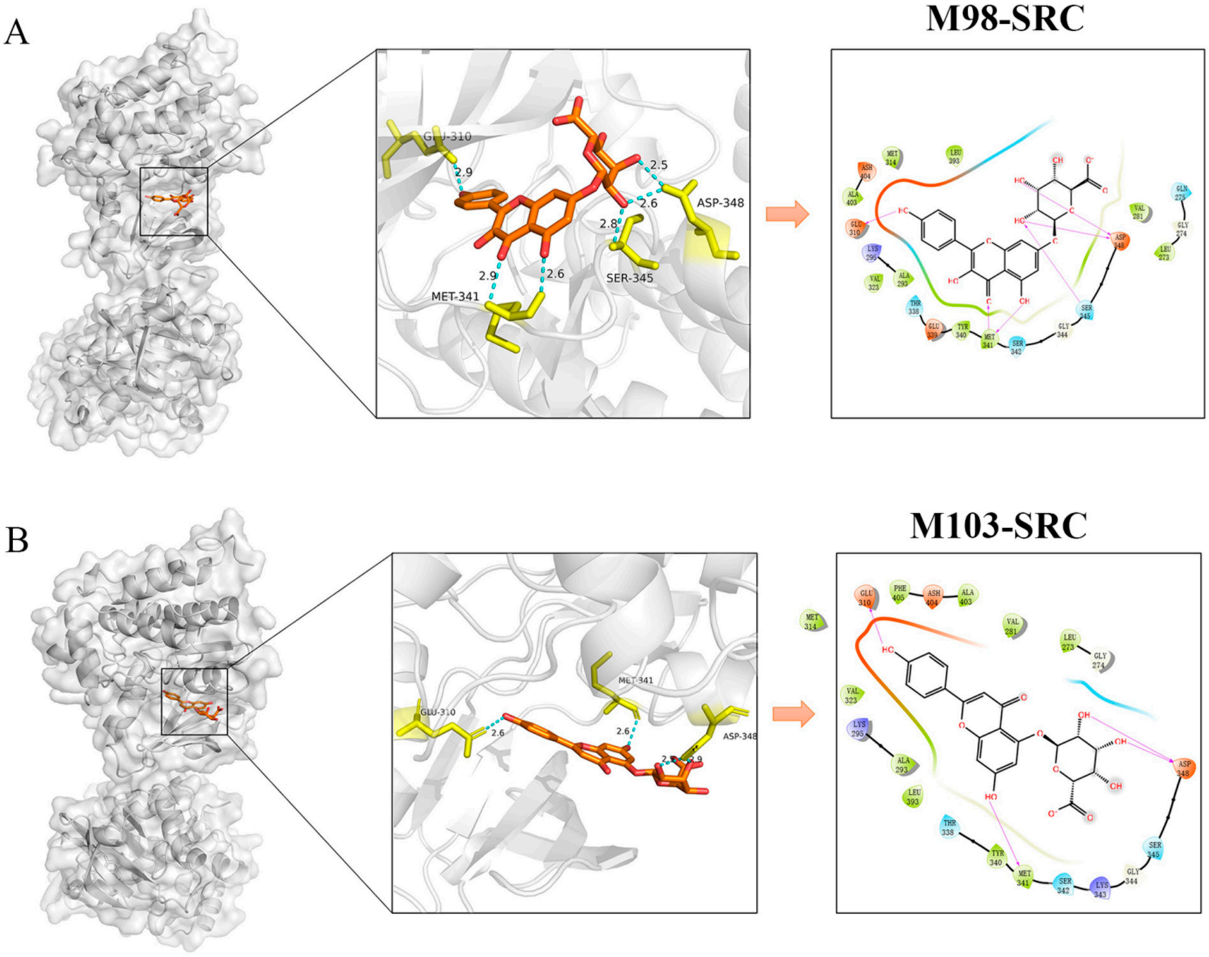

Figure S76 Molecular docking results of **M98** (A) and **M103** (B) with SRC.

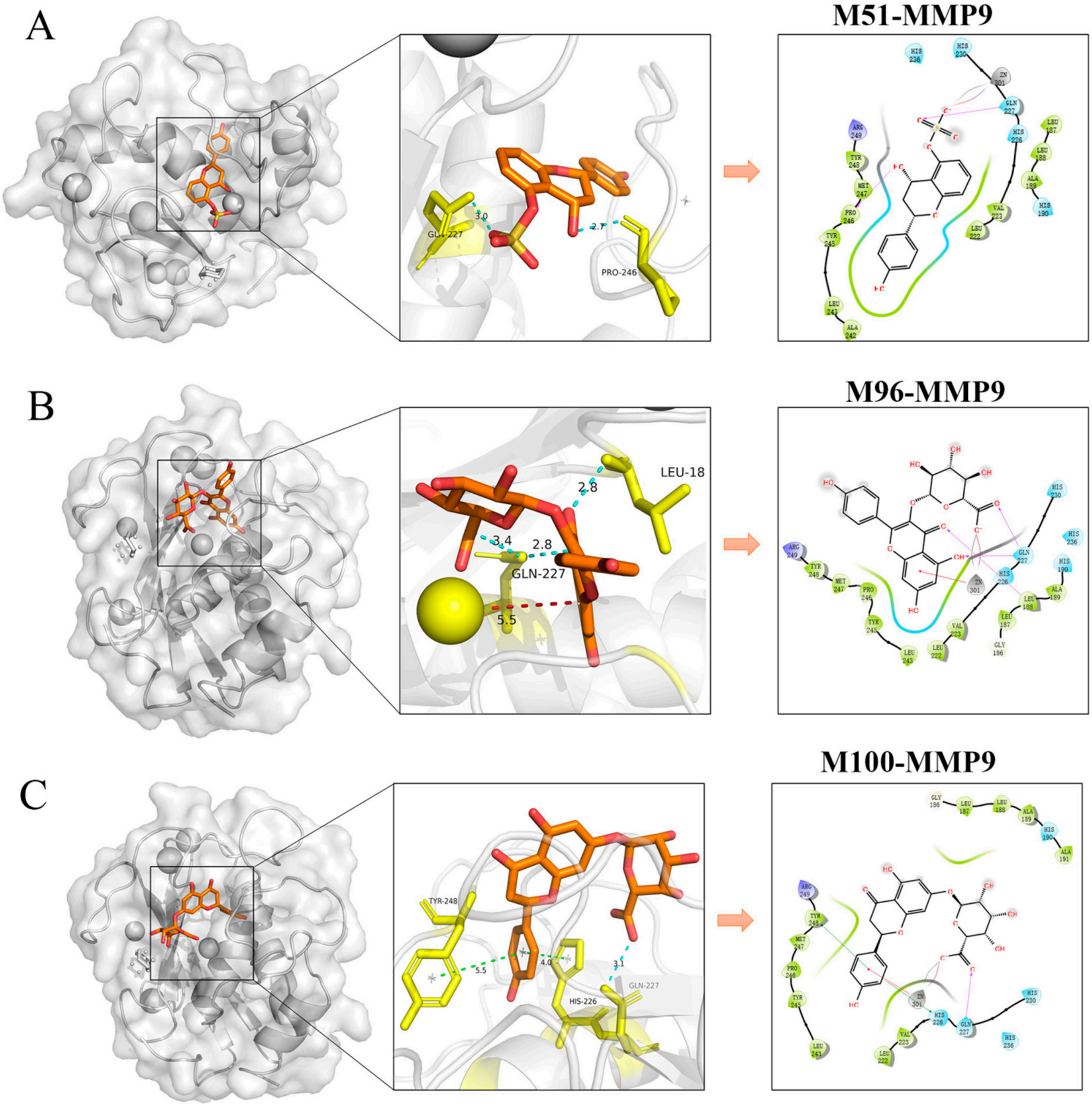

Figure S77 Molecular docking results of **M51** (A), **M96** (B), and **M100** (C) with MMP9.

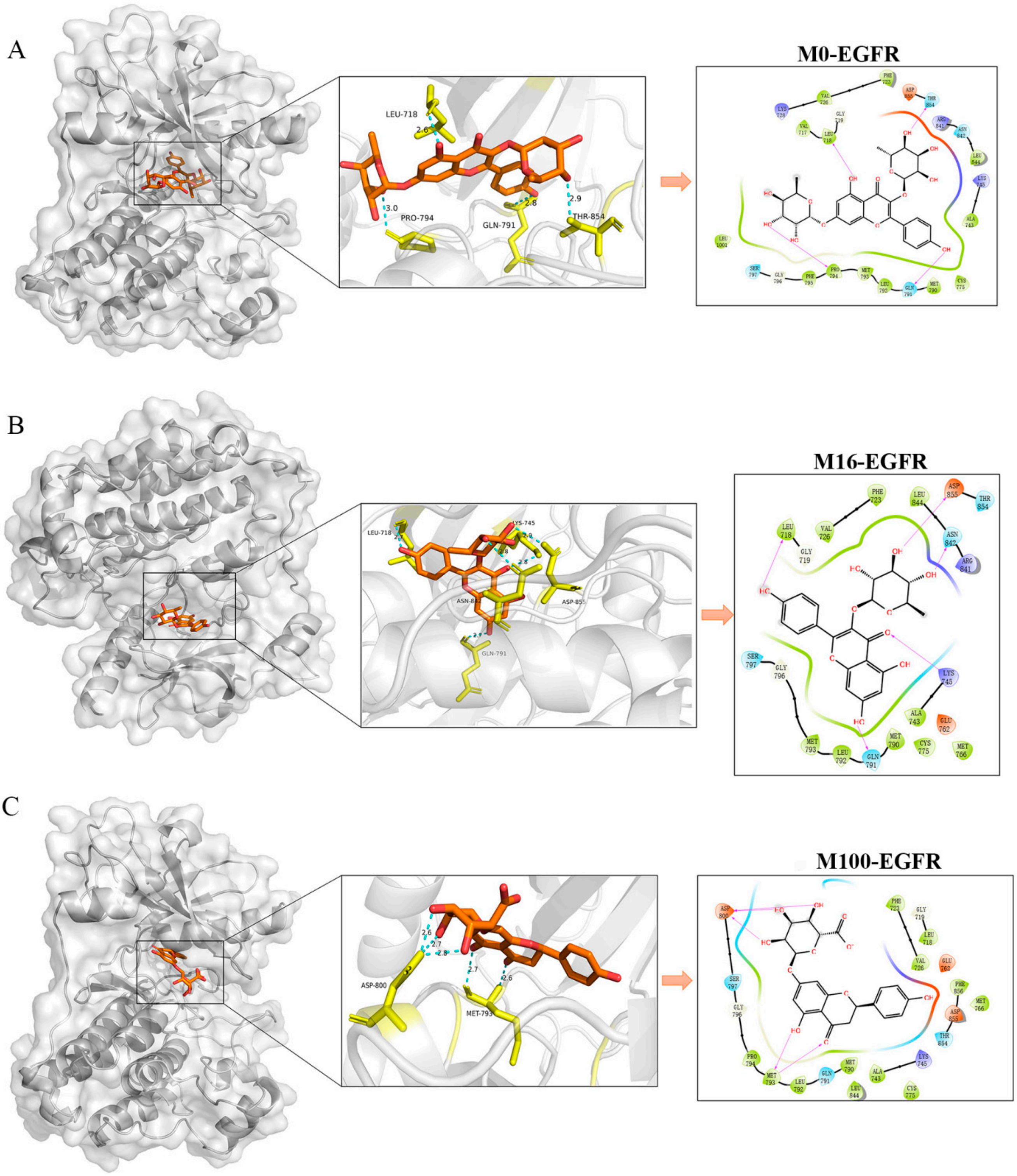

Figure S78 Molecular docking results of **M0** (A), **M16** (B), and **M100** (C) with EGFR.

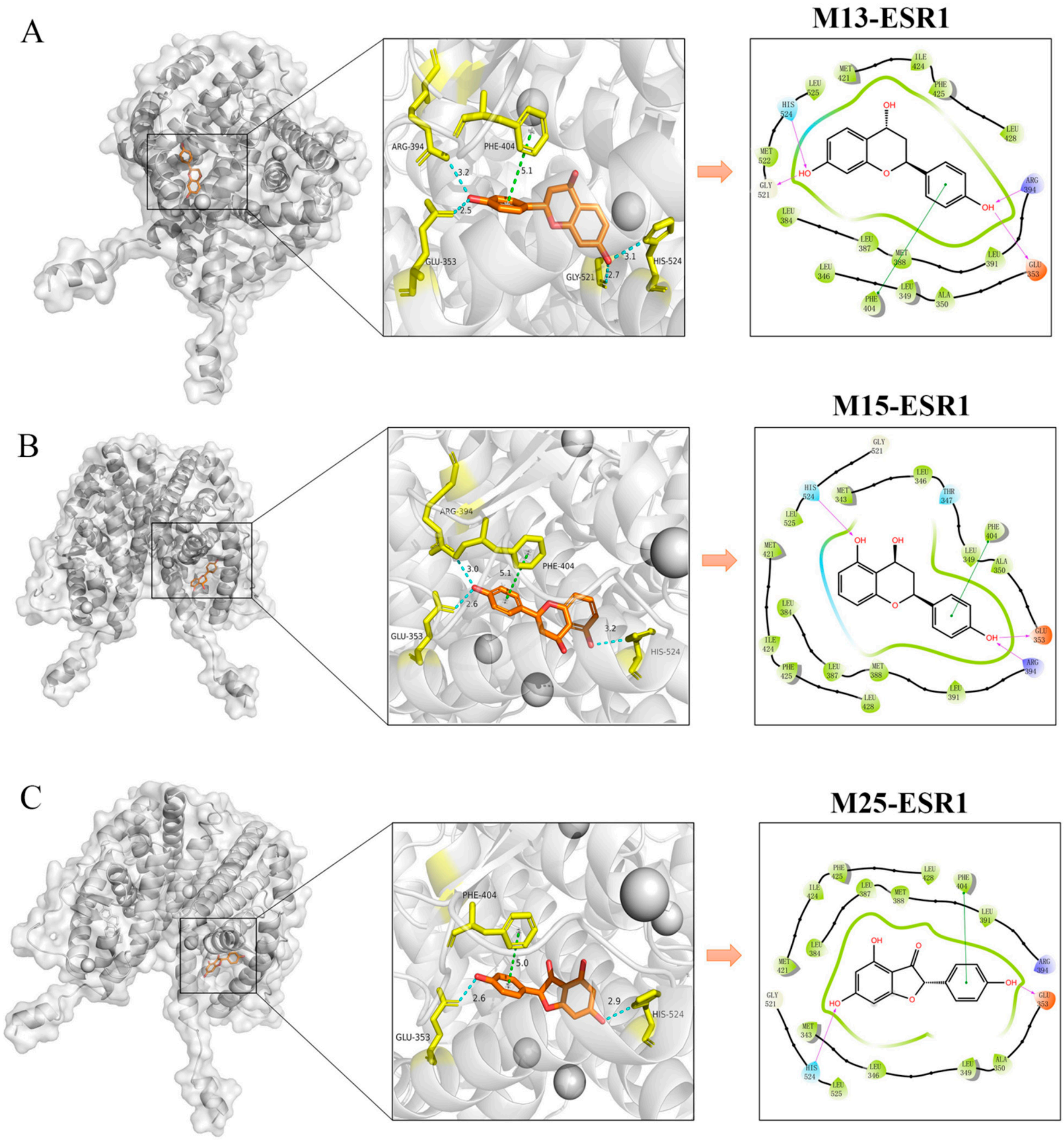

Figure S79 Molecular docking results of **M13** (A), **M15** (B), and **M25** (C) with ESR1.

Molecular docking using AutoDock Vina

The molecular docking experiment was conducted using AutoDock Vina version 1.1.2, with structural preprocessing and parameter settings performed via AutoDockTools version 1.5.7.

(1) Receptor protein preparation

The crystal structure of the target protein was obtained from the protein data bank (PDB) database (https://www.rcsb.org/). Using AutoDockTools, crystalline water molecules, co-crystallized ligands, and heteroatoms were removed. Hydrogen atoms were added in accordance with a physiological pH of 7.4 to correct the protonation states of amino acids, and the final structure was saved in protein data bank quantum chemistry/torsion (PDBQT) format.

(2) Ligand preparation

The ligand structure was drawn using ChemDraw and subsequently converted into pdb. format through Chem 3D. Redundant atoms were eliminated using AutoDockTools; hydrogen atoms were added, and the protonation state adjusted accordingly. Rotatable bonds were defined to maintain flexible conformations, Gasteiger charges were calculated, and the ligand was saved in PDBQT format.

(3) Grid box settings

A docking grid box was generated utilizing AutoGrid4, positioning its centroid at the active pocket's center to ensure comprehensive coverage of both the entire binding site and adjacent flexible regions.

(4) Interface parameter settings and scoring functions

Employing a flexible ligand-rigid receptor docking mode, search thoroughness (exhaustiveness) was set to 10; each ligand produced nine distinct conformations for evaluation. The binding energy calculations utilized the optimized potentials for liquid simulations (OPLS) force field, while conformations were ranked based on their binding free energy values. AutoDock Vina facilitates calculation of "binding free energy scores" when ligands interact with receptors. This score reflects the binding affinity within ligand-receptor complexes and serves as a tool for screening optimal binding conformations. The molecular docking scoring function employed by AutoDock Vina is detailed as follows:

$$\mathrm{score}_i = \frac{\mathrm{inter}_i + \mathrm{intra}_i - \mathrm{intra}_{\mathrm{best}}}{1 + 0.05846 \times n_{\mathrm{rot}}}$$

Inter_i: ligand-receptor interaction energy of the i-th conformation; Intra_i: intramolecular interaction energy of the ligand in the i-th conformation; $\mathrm{Intra}_{\mathrm{best}}$: interaction energy corresponding to the optimal (lowest energy) conformation within a ligand molecule; n_{rot}: number of rotatable bonds of a ligand.

(5) Results

The docking scores of KAE, 27 metabolites, and aspirin are shown in Figure S80. The core conclusions are consistent, and both tools demonstrate that KAE (**M0**), phase I metabolites (**M1**, **M6**, **M7**, **M8**, **M9**, **M11**, **M13**, **M14**, **M15**, **M16**, **M17**, **M18**, **M21**, **M24**, and **M25**), and phase II metabolites (**M28**, **M29**, **M51**, **M95**, **M96**, **M97**, **M98**, **M99**, **M100**, **M103**, **M104**, and **M105**) can bind to multiple antithrombotic targets though the binding ability varies (the docking scores meet the strong binding criteria).

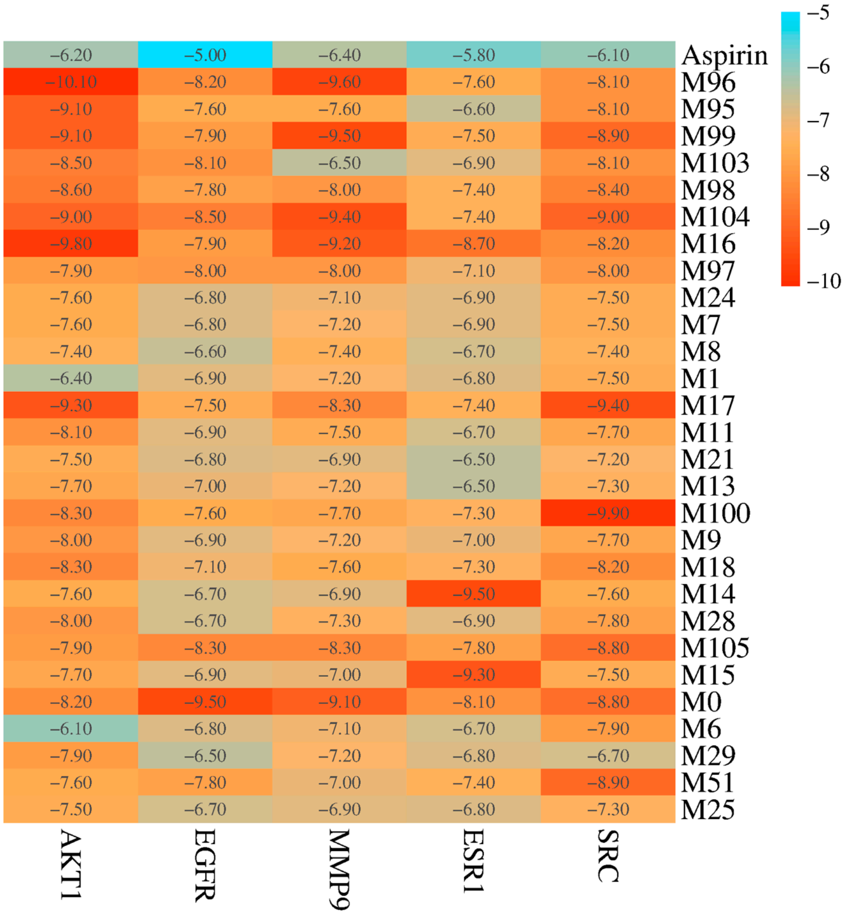

Figure S80. Heat map of molecular docking scoring (AutoDock Vina).

Based on the binding energy scores, the top 6 components identified by each of the two molecular docking methods were selected, and their intersection was determined (Table S15); the components in this intersection were further visualized for analysis. Specifically, AKT1 exhibited primary binding interactions with M95, M96, M98, M99, and M104; EGFR primarily bound to M0, M96, and M104; MMP9 showed main binding affinity for M96, M99, and M104; ESR1 was mainly associated with M14, M15, and M16; and SRC primarily interacted with M17, M100, and M104. The results show that the outcomes of the two molecular docking methods are consistent.

For the same "compound–target" system, the RMSD values calculated by the two methods were both ≤ 2 Å (a commonly used threshold in the molecular docking field), with a numerical difference of ≤ 0.5 Å. This indicates that the ligand binding conformations predicted by the two methods were highly consistent, and the docking results exhibited good stability, supporting the reliability of the docking conclusions for this system.

In conclusion, the results from the two molecular docking methods collectively suggest that KAE (M0) and its 27 metabolites may serve as the key components mediating the antithrombotic activity of KAE.

Table S15

Intersection of the top 6 “effective forms” scored by two molecular docking methods

**Schrödinger Suite 2021**				**AutoDock Vina**			
**Protein**	**Ligand**	**Binding energy (Kcal/mol)**	**RMSD value (Å)**	**Protein**	**Ligand**	**Binding energy (Kcal/mol)**	**RMSD value (Å)**
AKT1	M95	-9.28	1.035	AKT1	M95	-9.1	1.201
	M96	-9.32	0.964		M96	-10.1	1.202

	M98	-8.74	1.026		M98	-8.6	1.201
	M99	-9.25	0.987		M99	-9.1	1.196
Youxiao	M104	-8.57	1.354		M104	-9.0	1.201
	M0	-9.65	1.427		M0	-9.5	1.205
EGFR	M96	-8.81	1.562	EGFR	M96	-8.2	1.208
	M104	-8.52	1.468		M104	-8.5	1.206
	M96	-9.72	0.974		M96	-9.6	1.201
MMP9	M99	-9.63	1.158	MMP9	M99	-9.5	1.207
	M104	-9.42	0.736		M104	-9.4	0.462
	M14	-10.41	1.205		M14	-9.5	1.112
ESR1	M15	-9.09	1.237	ESR1	M15	-9.3	1.109
	M16	-9.03	0.867		M16	-8.7	1.103
	M17	-10.88	1.529		M17	-9.4	1.199
SRC	M100	-10.90	1.263	SRC	M100	-9.9	1.205
	M104	-10.32	1.035		M104	-9.0	1.201
